# Supplementary material for: Late-life restoration of mitochondrial function reverses cardiac dysfunction in old mice
Source: eLife. 2020 Jul 10;9:e55513. doi: 10.7554/eLife.55513 (PMC7377906; doi:10.7554/eLife.55513)
Supplement: Figure 2—source data 1. [file elife-55513-fig2-data1.zip › MitoSOX images/MitoSOX image description.docx]

**Images for MitoSOX/MitoTracker Green measurement**

The LIF files in this folder can be opened with Fiji software. Each LIF file contains images of one to multiple cardiomyocytes isolated from a control (saline) or a SS-31-treated mouse as indicated in the image name. For each image, MitoTracker Green signal was captured in Channel 1 and MitoSOX signal was captured in Channel 2. The intensity ratio of MitoSOX/MitoTracker Green (ie. Channel 2/Channel 1) was calculated for each cell.
