# Supplementary material for: Late-life restoration of mitochondrial function reverses cardiac dysfunction in old mice
Source: eLife. 2020 Jul 10;9:e55513. doi: 10.7554/eLife.55513 (PMC7377906; doi:10.7554/eLife.55513)
Supplement: Figure 2—source data 2. [file elife-55513-fig2-data2.zip › MitoPY1/MitoPY1 image description.docx]

**Images for MitoPY1/MitoTracker Deep Red measurement**

The LIF files in this folder can be opened with Fiji software. Each LIF file contains images of one to multiple cardiomyocytes isolated from a control (saline) or a SS-31-treated mouse as indicated in the image name. For each image, MitoPY1 signal was captured in Channel 1 and MitoTracker Deep Red signal was captured in Channel 2. The intensity ratio of MitoPY1/ MitoTracker Deep Red (ie. Channel 1/Channel 2) was calculated for each cell.
