# Supplementary figures and images for: Late-life restoration of mitochondrial function reverses cardiac dysfunction in old mice (part 2 of 3)
Source: eLife. 2020 Jul 10;9:e55513. doi: 10.7554/eLife.55513 (PMC7377906; doi:10.7554/eLife.55513)

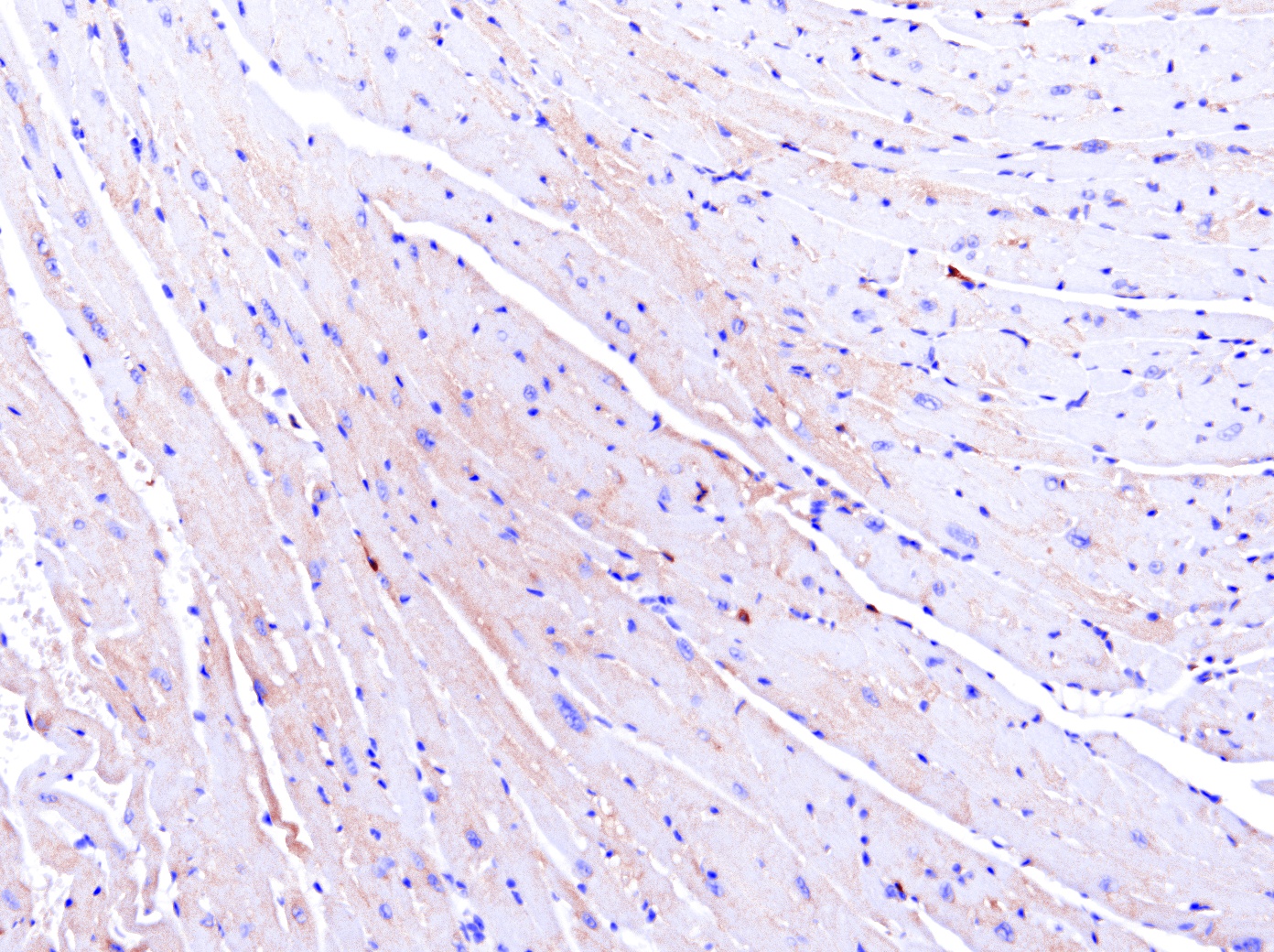

Supplement: Figure 4—source data 1. [file elife-55513-fig4-data1.zip › p16_images_for_eLife/p16_images_Ann_Chiao_for_eLife/Old Controls/OCL_9/Composite_7.3_d.jpg]

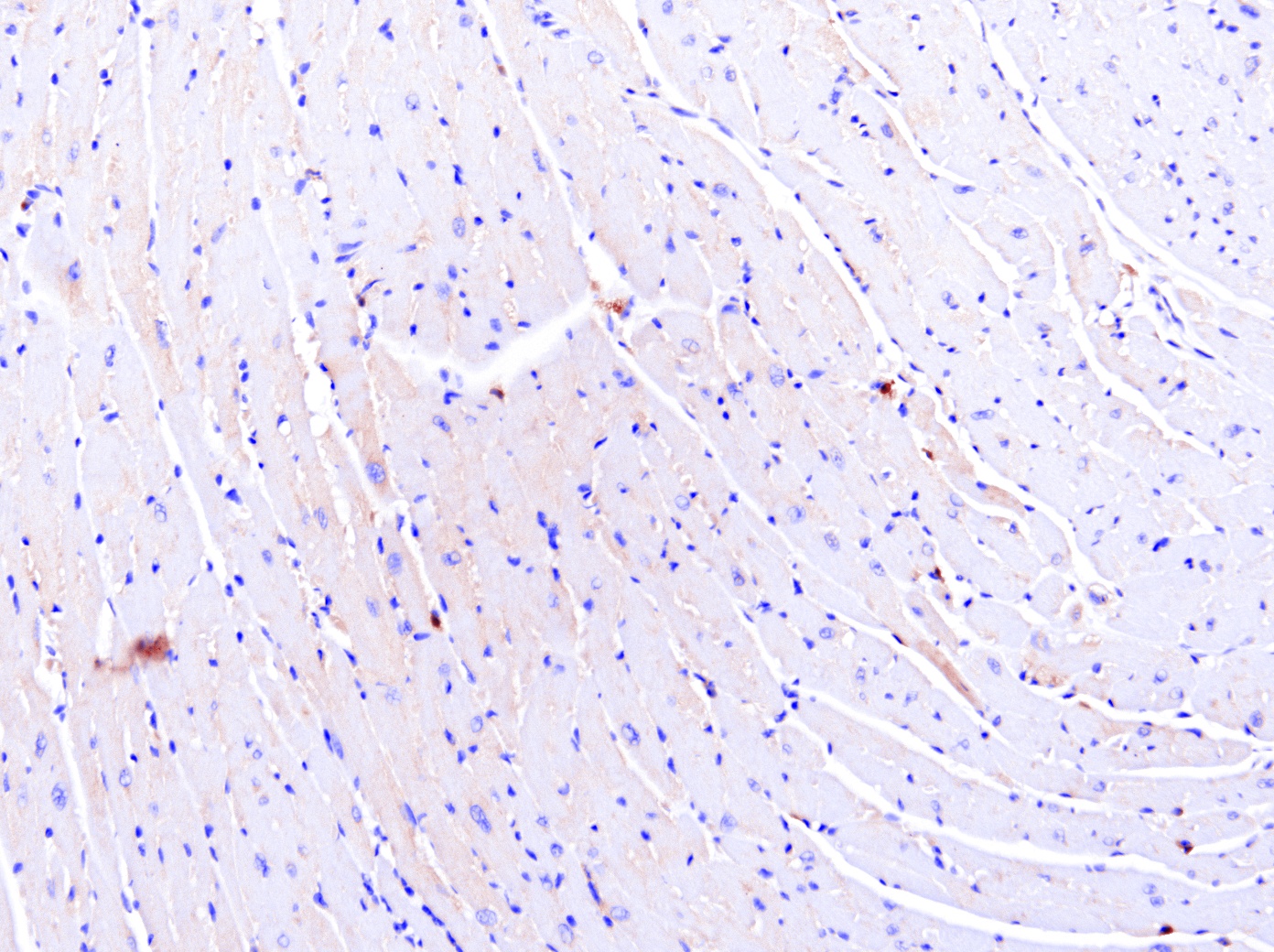

Supplement: Figure 4—source data 1. [file elife-55513-fig4-data1.zip › p16_images_for_eLife/p16_images_Ann_Chiao_for_eLife/Old Controls/OCL_9/Composite_7.3_e.jpg]

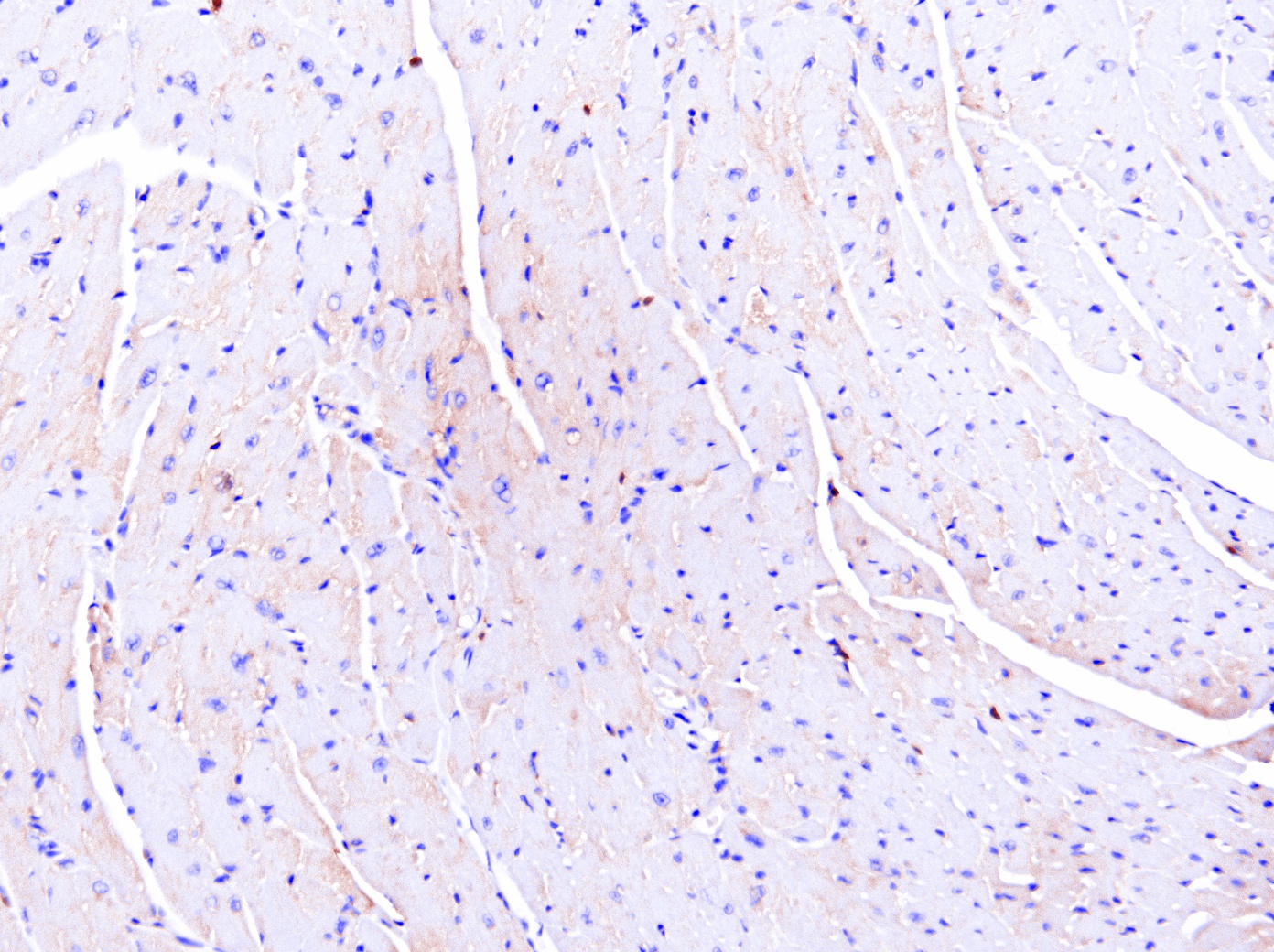

Supplement: Figure 4—source data 1. [file elife-55513-fig4-data1.zip › p16_images_for_eLife/p16_images_Ann_Chiao_for_eLife/Old Controls/OCL_9/Composite_7.3_f.jpg]

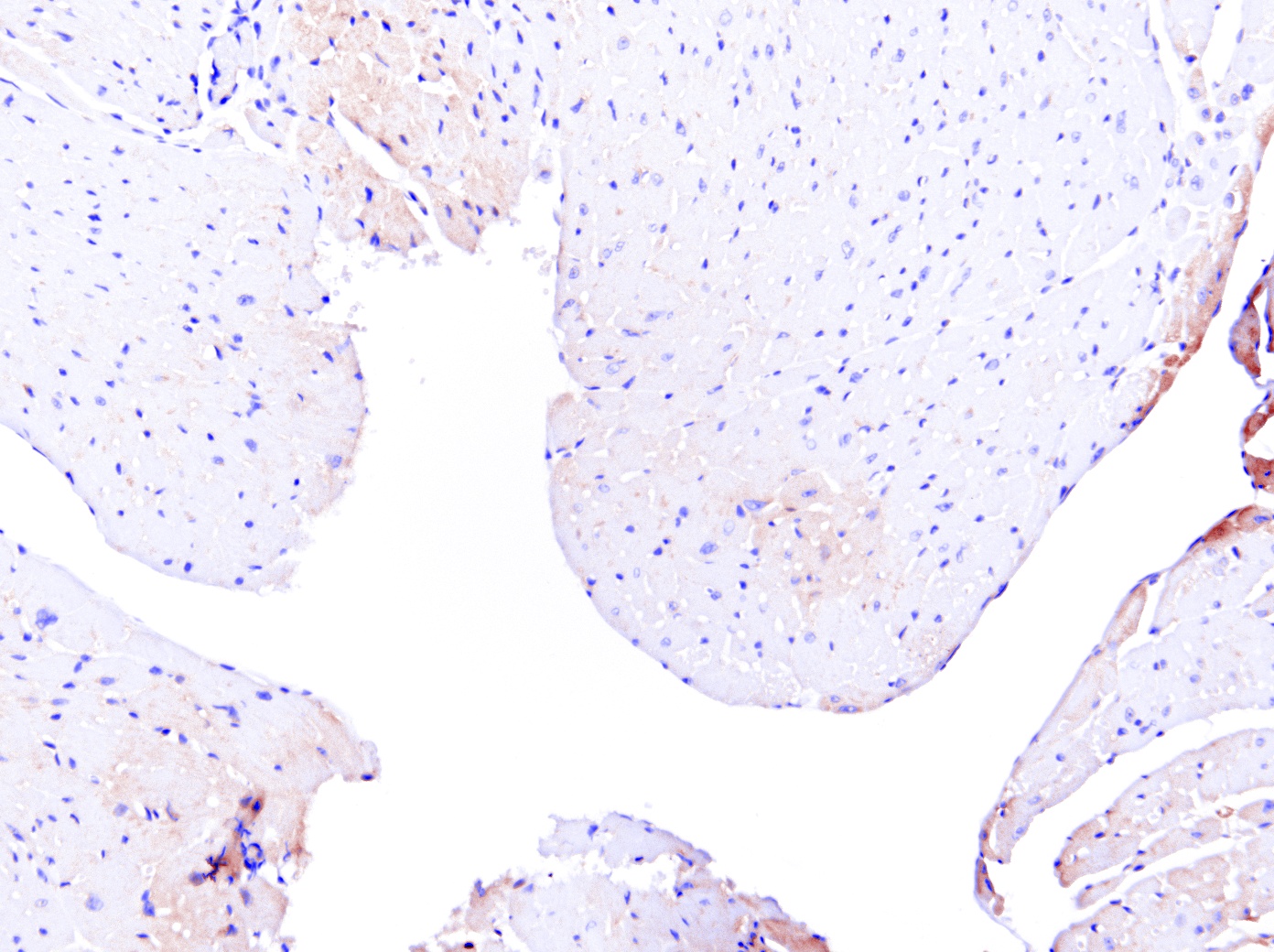

Supplement: Figure 4—source data 1. [file elife-55513-fig4-data1.zip › p16_images_for_eLife/p16_images_Ann_Chiao_for_eLife/Old Controls/OCL_9/Composite_7.3_g.jpg]

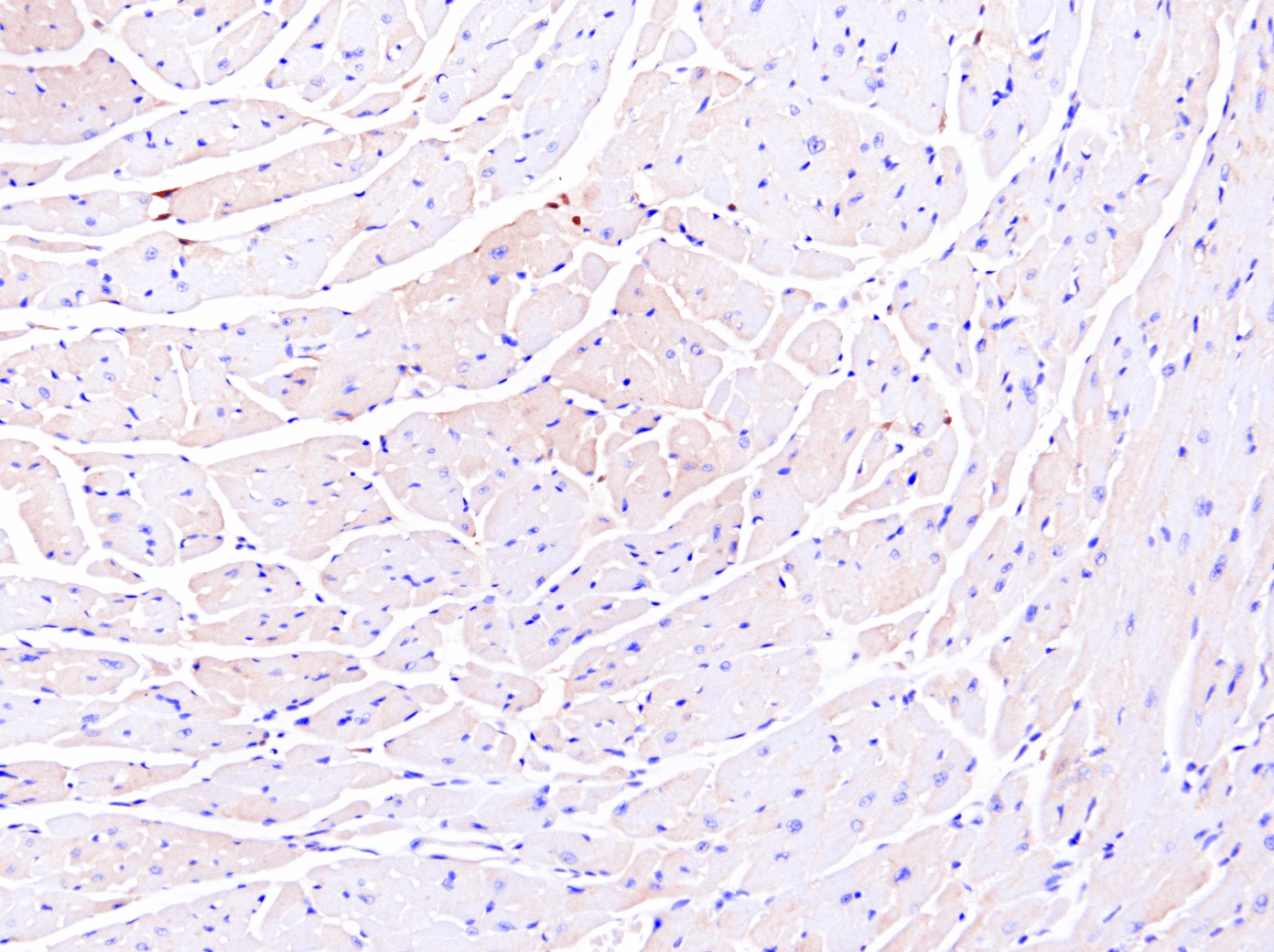

Supplement: Figure 4—source data 1. [file elife-55513-fig4-data1.zip › p16_images_for_eLife/p16_images_Ann_Chiao_for_eLife/Old Controls/OCL_9/Composite_7.3_h.jpg]

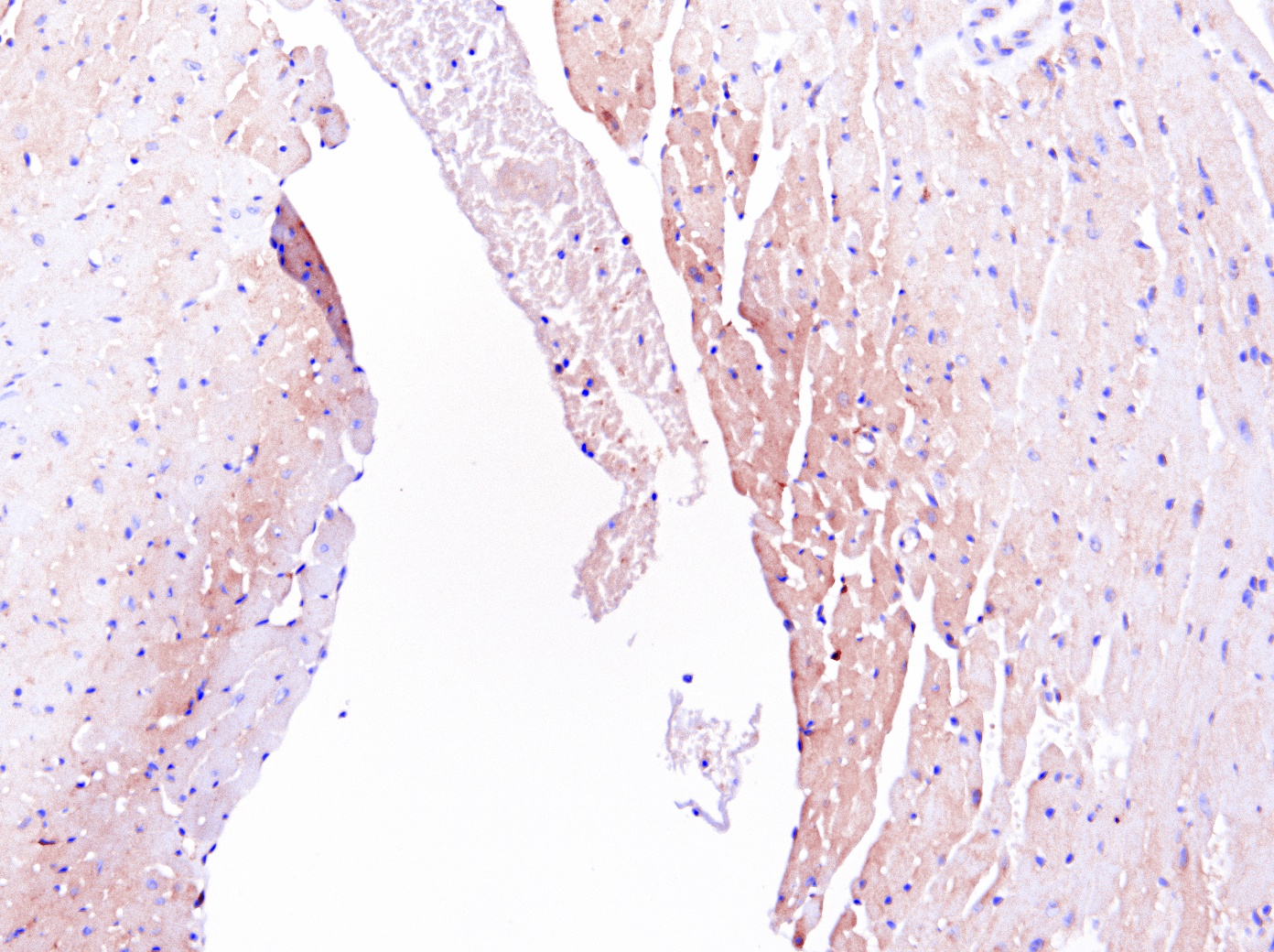

Supplement: Figure 4—source data 1. [file elife-55513-fig4-data1.zip › p16_images_for_eLife/p16_images_Ann_Chiao_for_eLife/Old Controls/OCL_9/Composite_7.3_i.jpg]

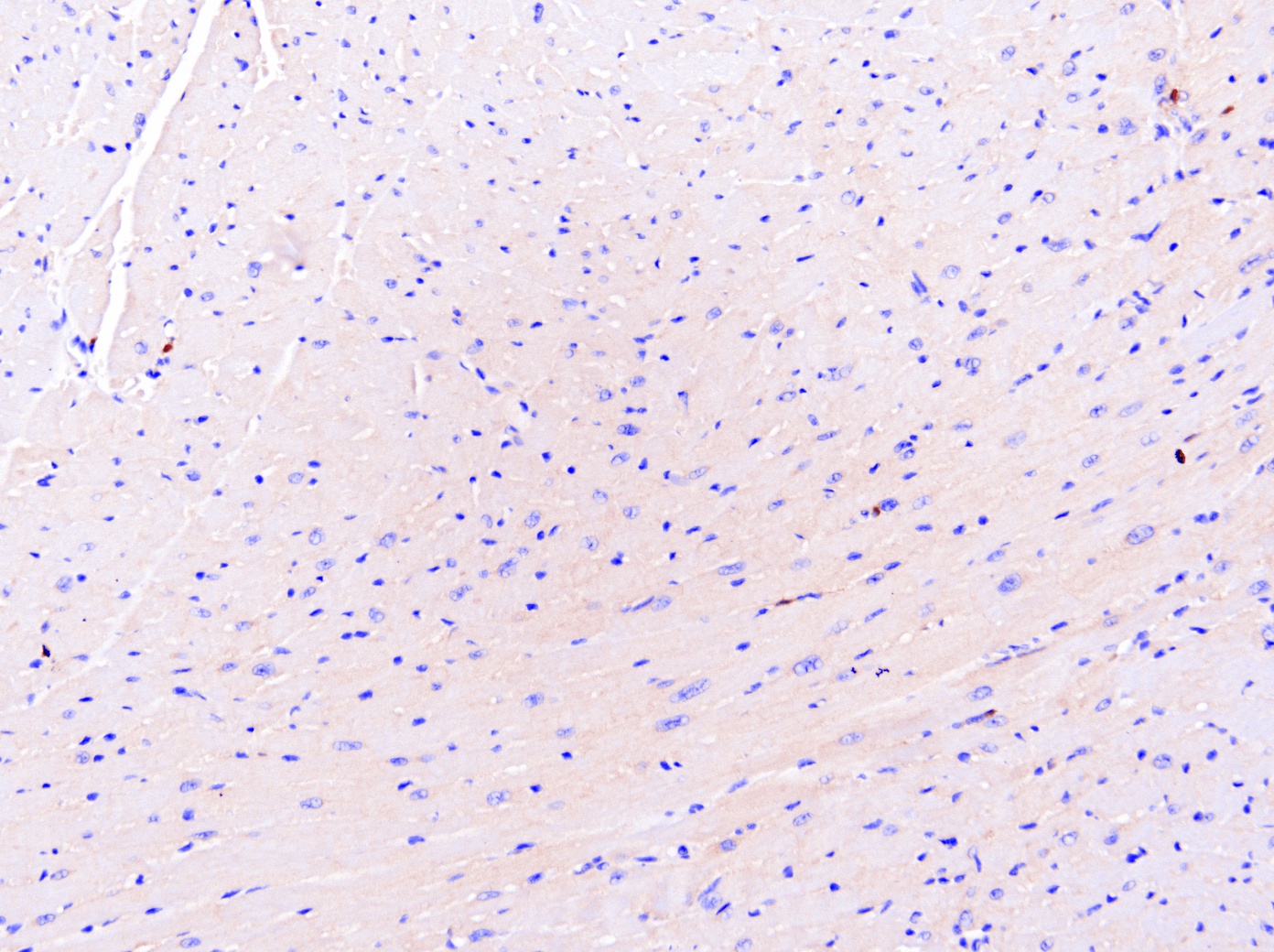

Supplement: Figure 4—source data 1. [file elife-55513-fig4-data1.zip › p16_images_for_eLife/p16_images_Ann_Chiao_for_eLife/Old Controls/OCL_9/Composite_7.3_j.jpg]

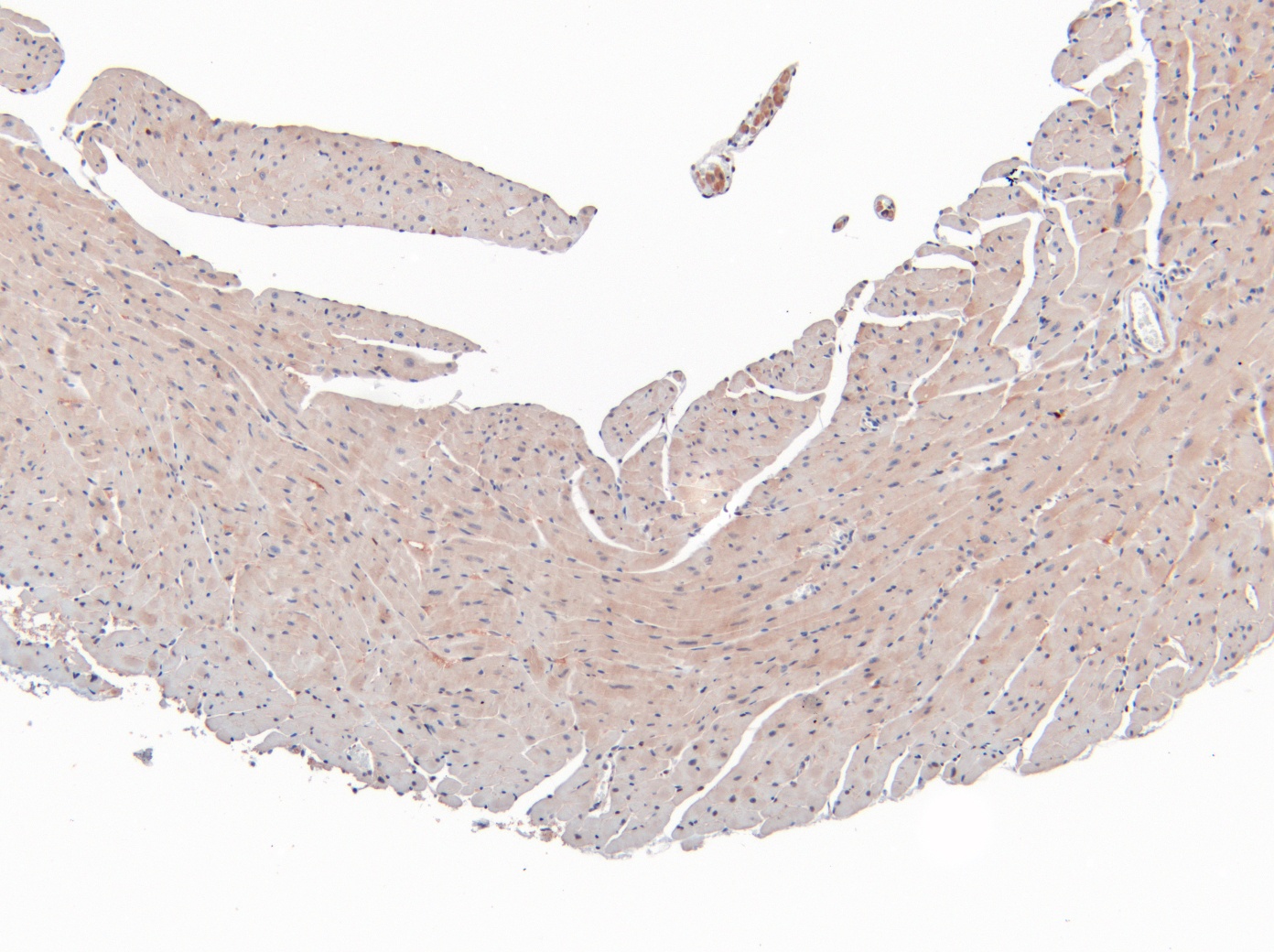

Supplement: Figure 4—source data 1. [file elife-55513-fig4-data1.zip › p16_images_for_eLife/p16_images_Ann_Chiao_for_eLife/Old SS-31 Treated/OSS_1/MS_1_p16_10x_a_RGB.jpg]

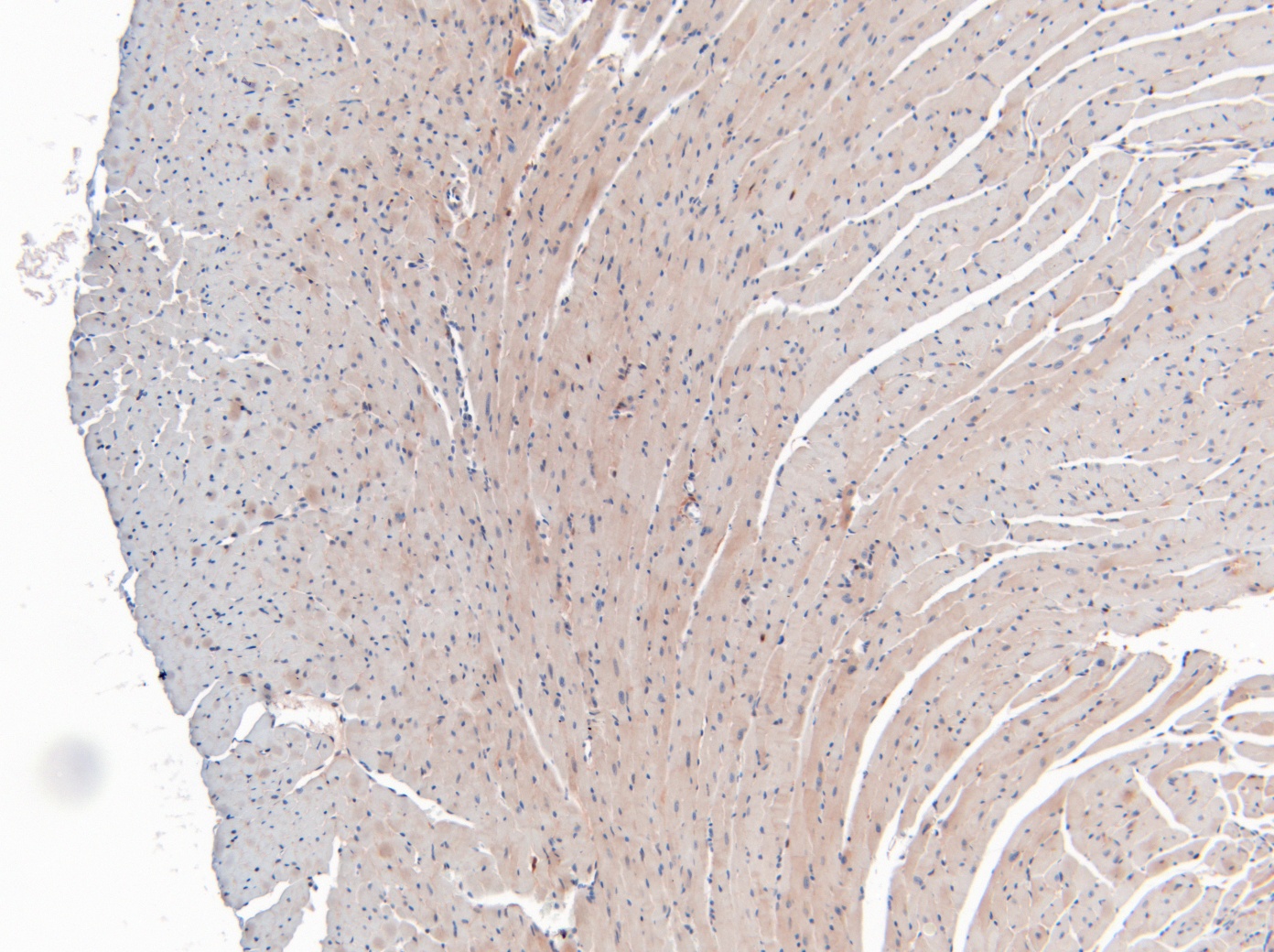

Supplement: Figure 4—source data 1. [file elife-55513-fig4-data1.zip › p16_images_for_eLife/p16_images_Ann_Chiao_for_eLife/Old SS-31 Treated/OSS_1/MS_1_p16_10x_c_RGB.jpg]

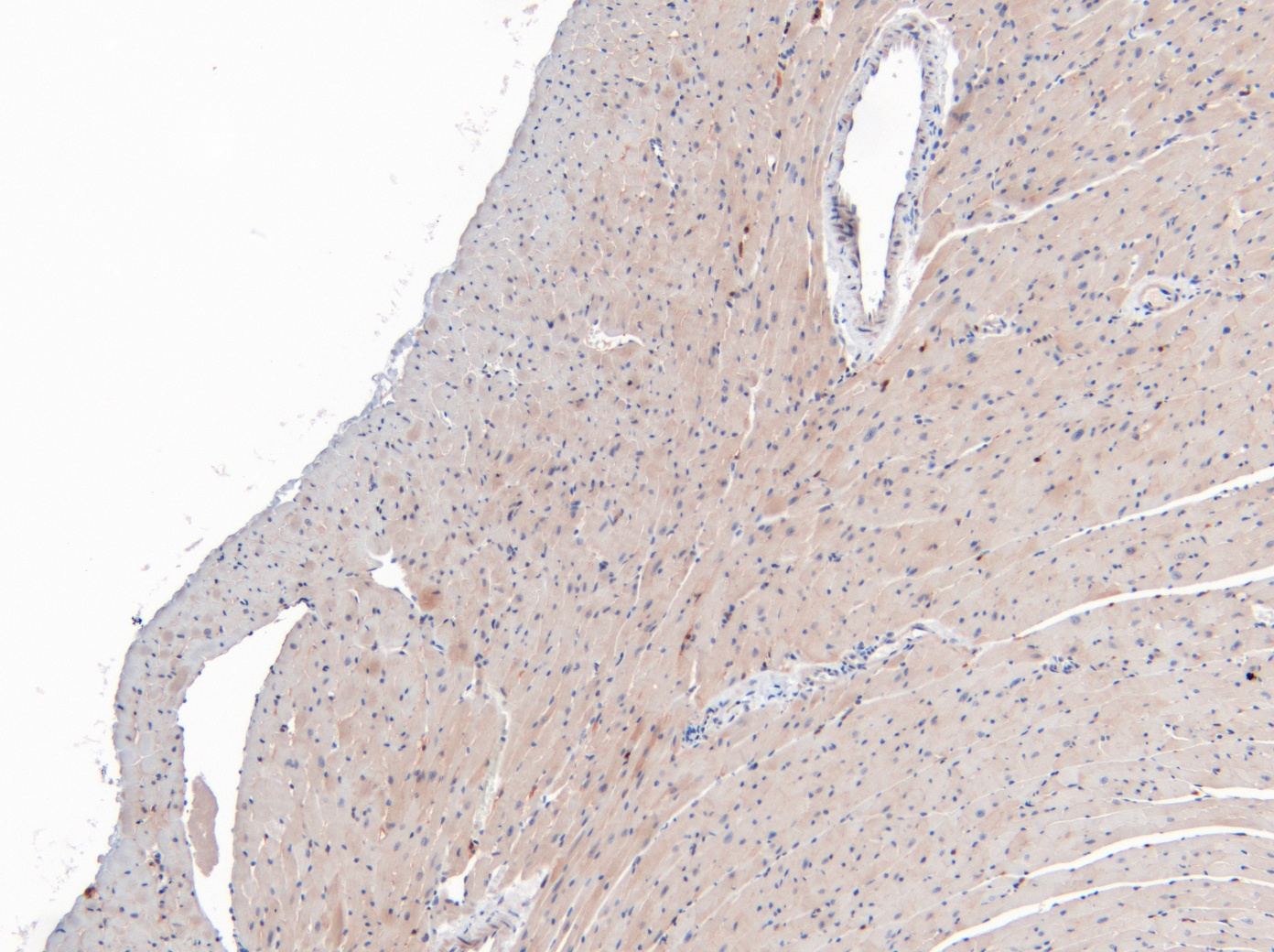

Supplement: Figure 4—source data 1. [file elife-55513-fig4-data1.zip › p16_images_for_eLife/p16_images_Ann_Chiao_for_eLife/Old SS-31 Treated/OSS_1/MS_1_p16_10x_d_RGB.jpg]

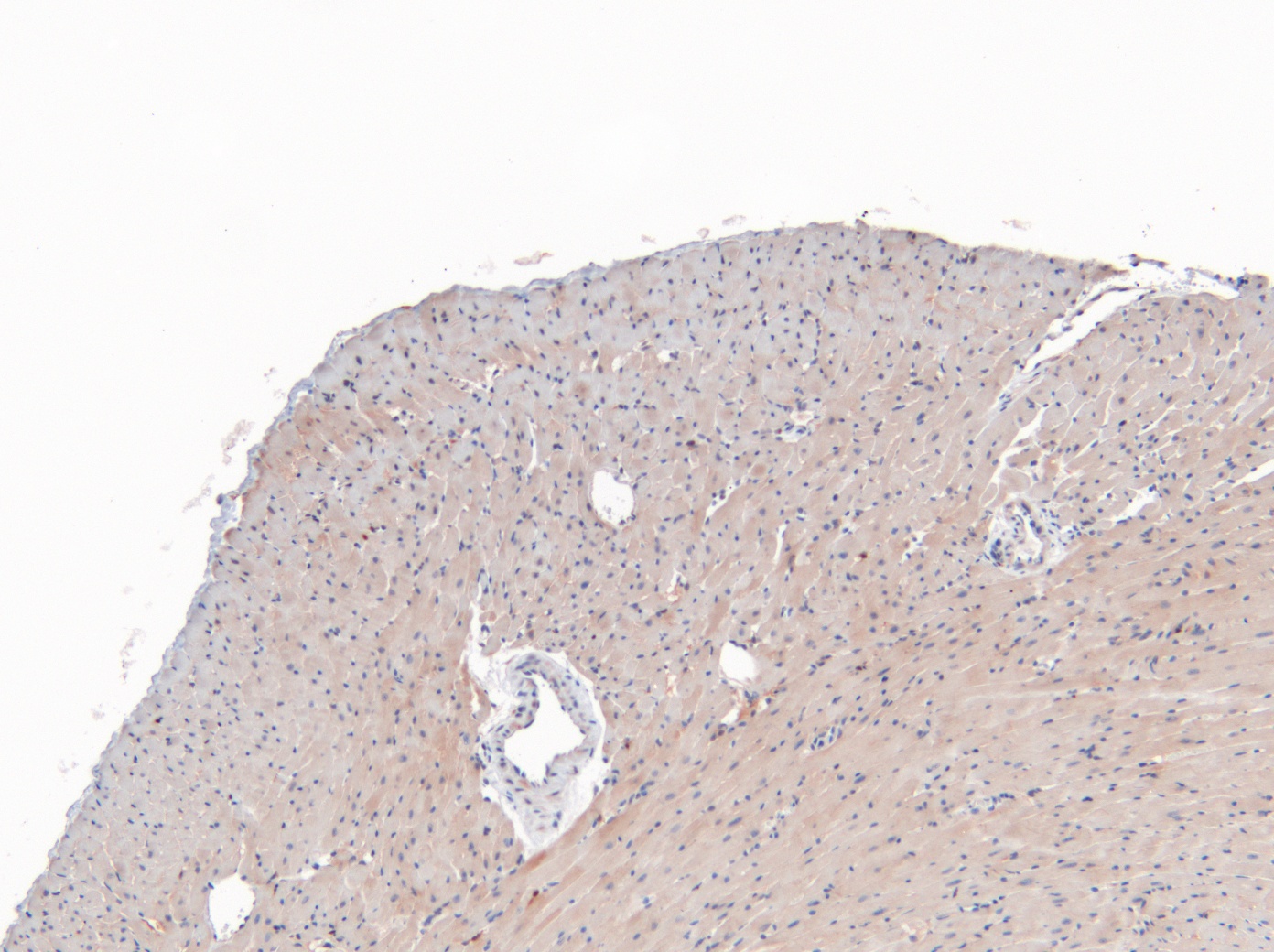

Supplement: Figure 4—source data 1. [file elife-55513-fig4-data1.zip › p16_images_for_eLife/p16_images_Ann_Chiao_for_eLife/Old SS-31 Treated/OSS_1/MS_1_p16_10x_e_RGB.jpg]

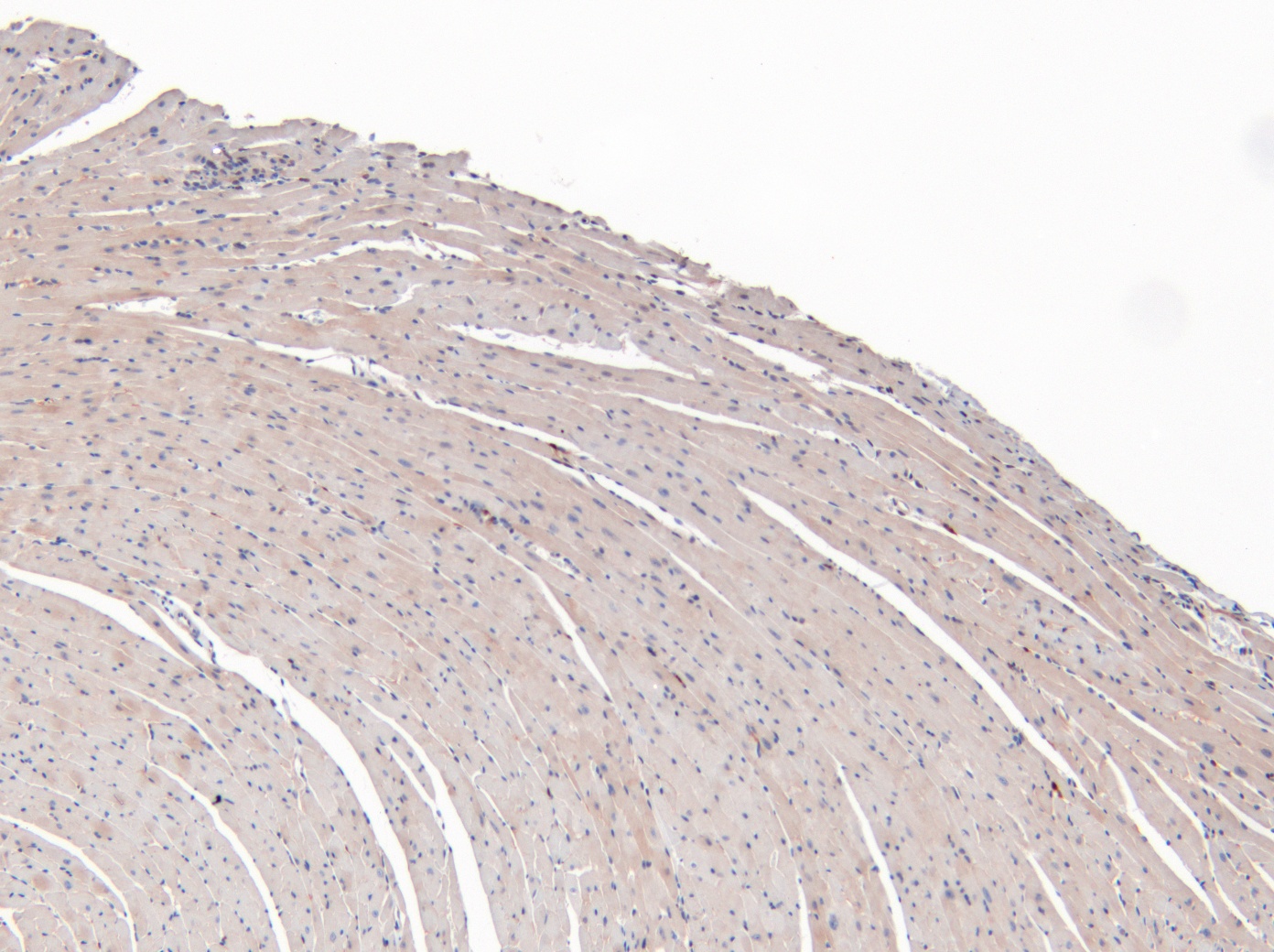

Supplement: Figure 4—source data 1. [file elife-55513-fig4-data1.zip › p16_images_for_eLife/p16_images_Ann_Chiao_for_eLife/Old SS-31 Treated/OSS_1/MS_1_p16_10x_f_RGB.jpg]

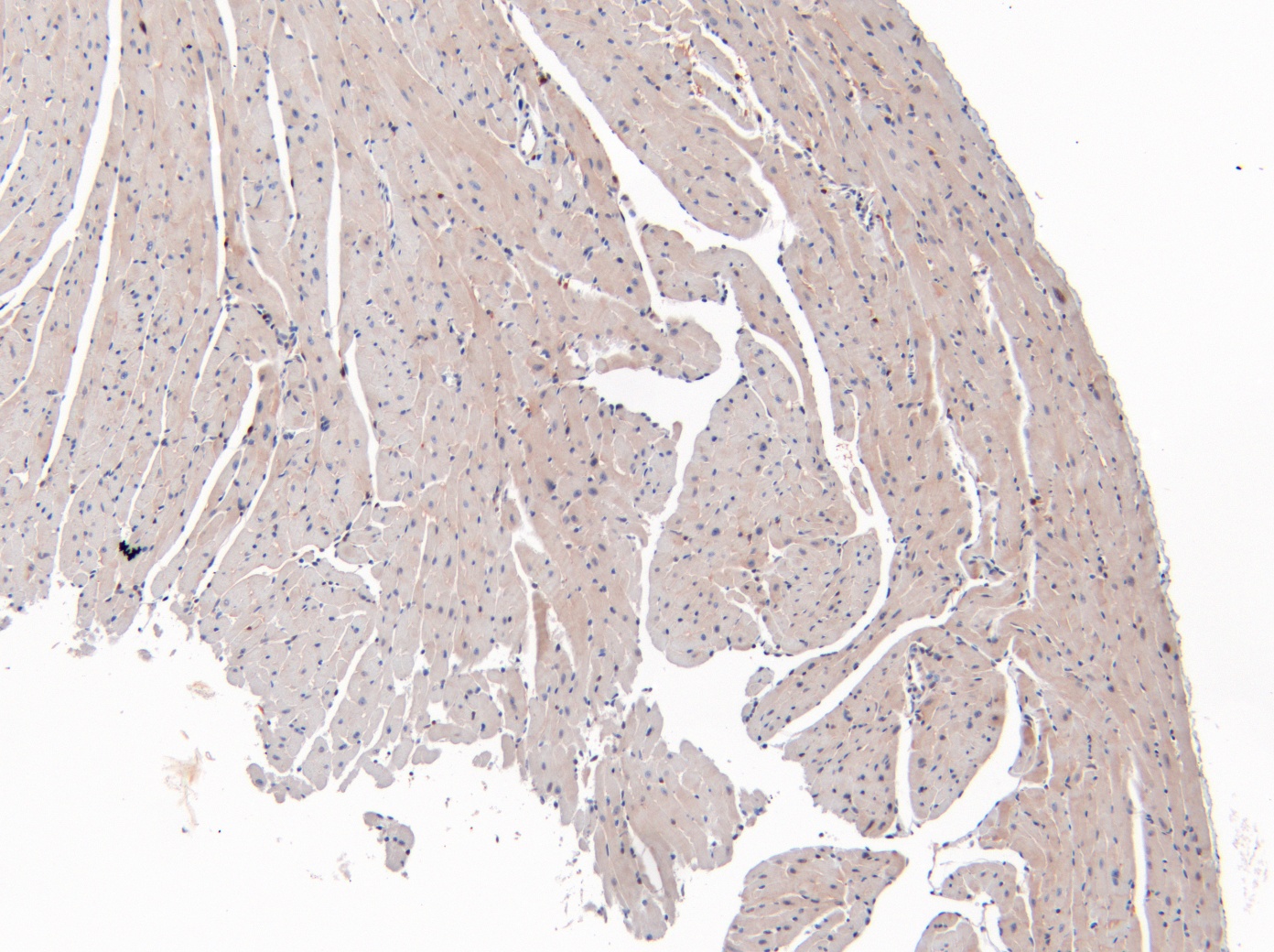

Supplement: Figure 4—source data 1. [file elife-55513-fig4-data1.zip › p16_images_for_eLife/p16_images_Ann_Chiao_for_eLife/Old SS-31 Treated/OSS_1/MS_1_p16_10x_g_RGB.jpg]

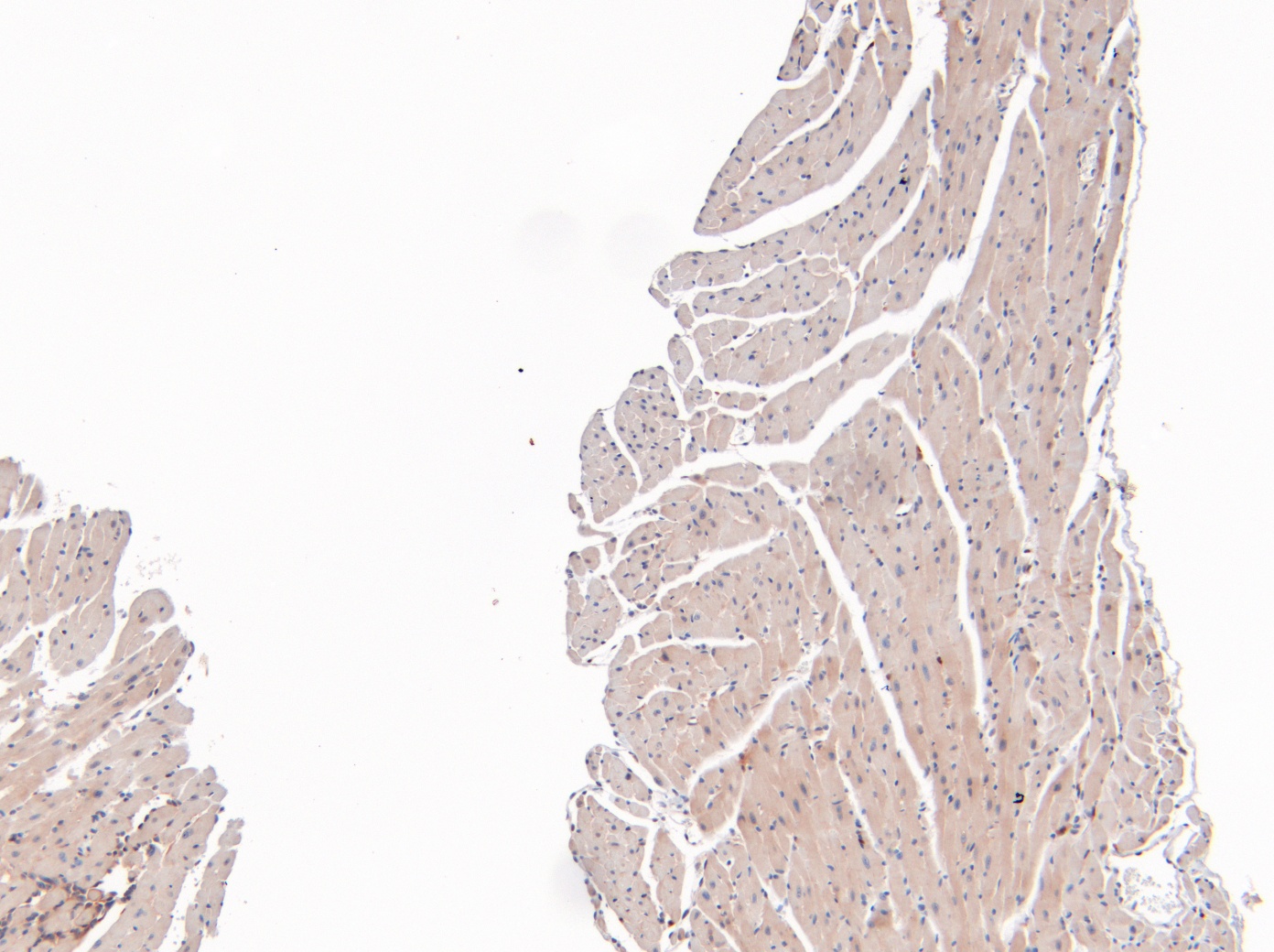

Supplement: Figure 4—source data 1. [file elife-55513-fig4-data1.zip › p16_images_for_eLife/p16_images_Ann_Chiao_for_eLife/Old SS-31 Treated/OSS_1/MS_1_p16_10x_h_RGB.jpg]

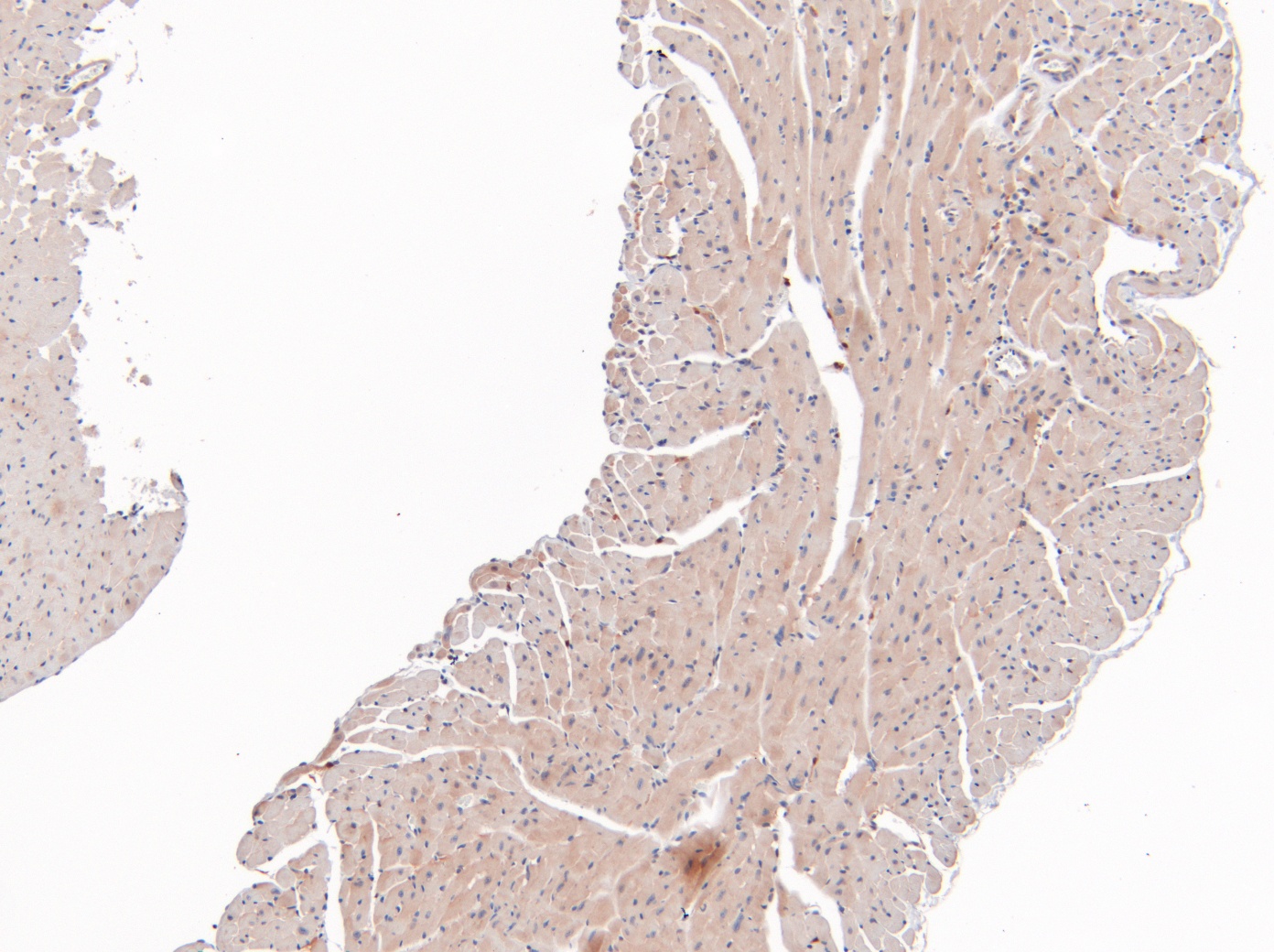

Supplement: Figure 4—source data 1. [file elife-55513-fig4-data1.zip › p16_images_for_eLife/p16_images_Ann_Chiao_for_eLife/Old SS-31 Treated/OSS_1/MS_1_p16_10x_i_RGB.jpg]

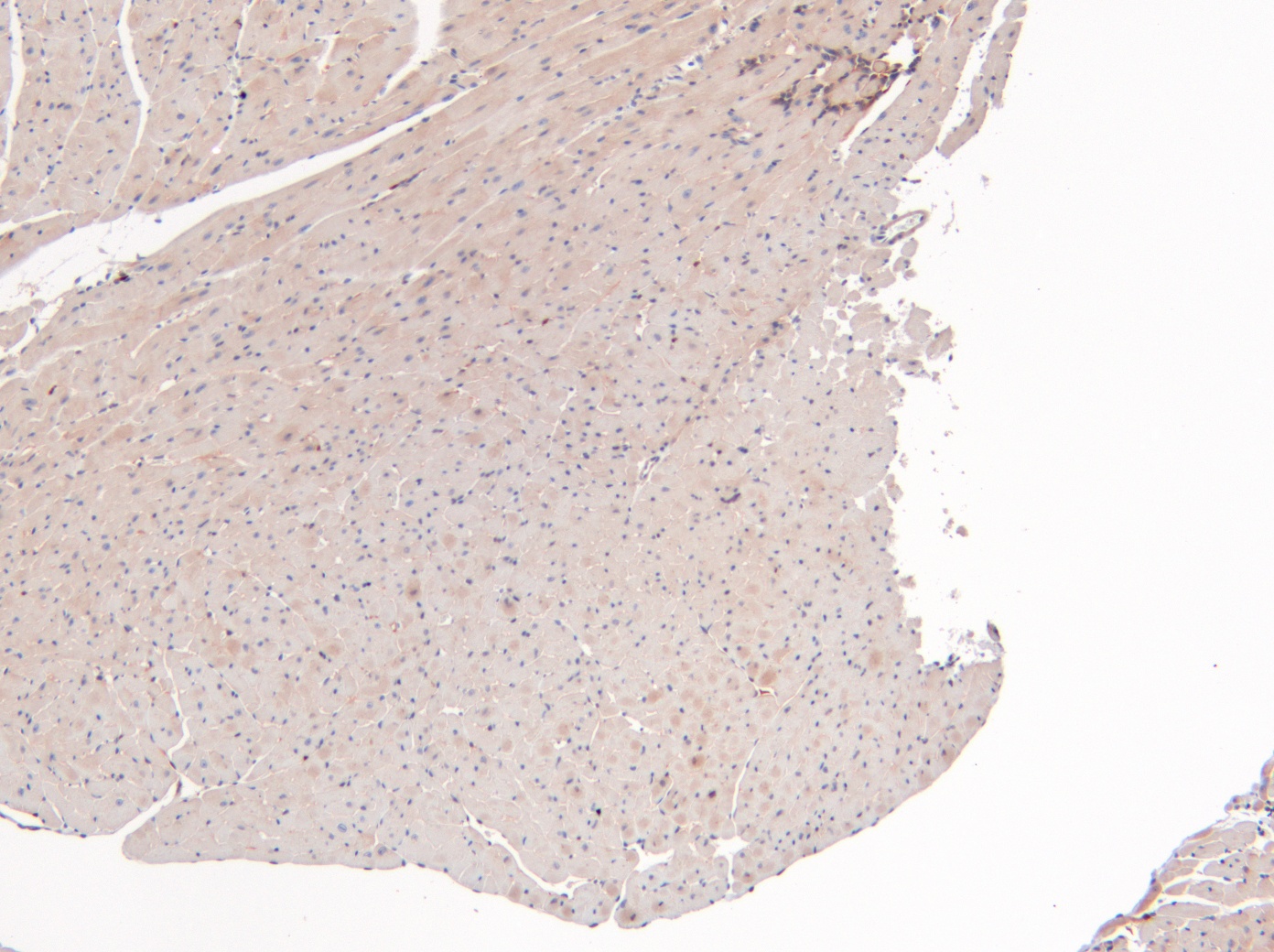

Supplement: Figure 4—source data 1. [file elife-55513-fig4-data1.zip › p16_images_for_eLife/p16_images_Ann_Chiao_for_eLife/Old SS-31 Treated/OSS_1/MS_1_p16_10x_j_RGB.jpg]

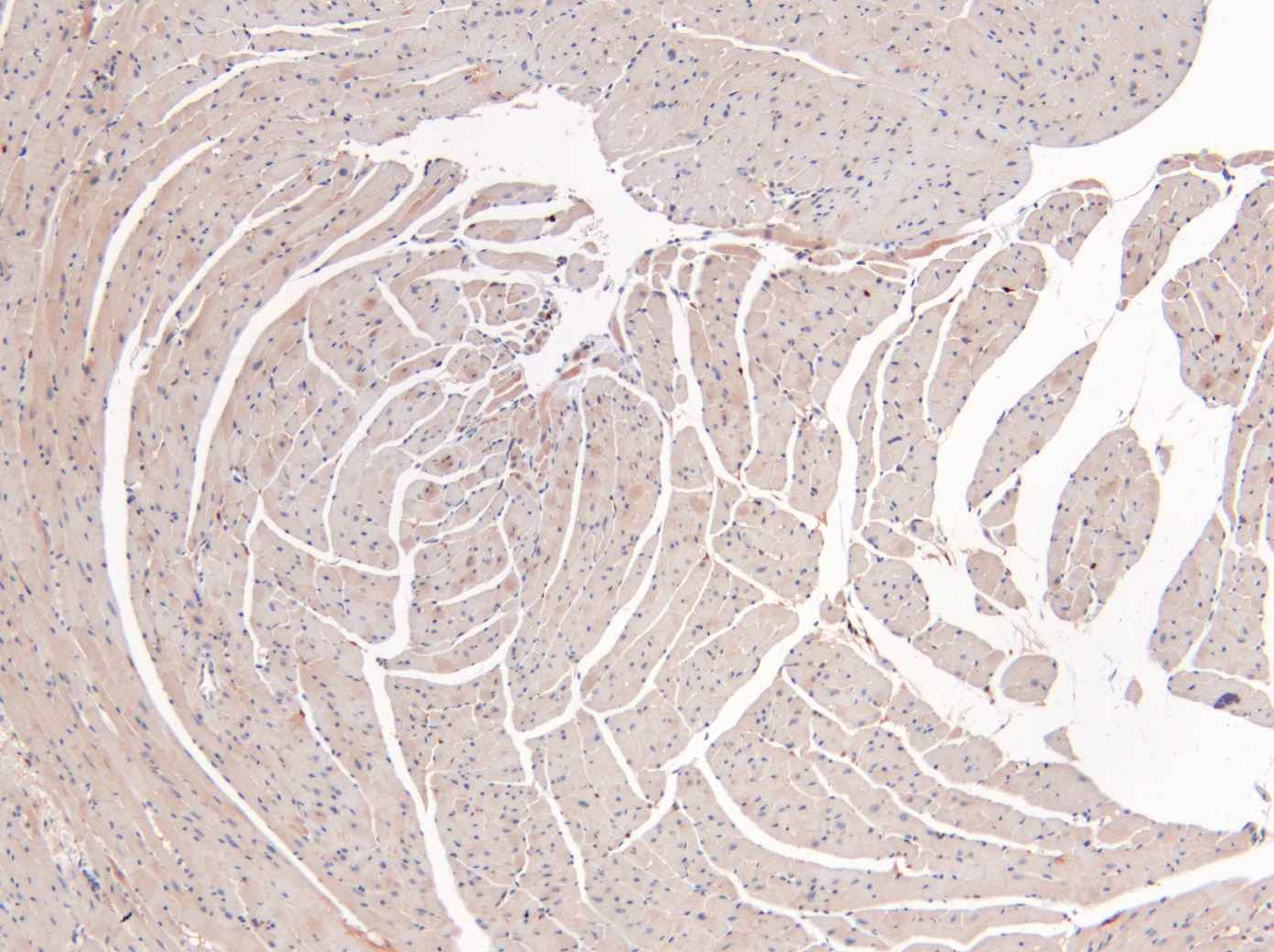

Supplement: Figure 4—source data 1. [file elife-55513-fig4-data1.zip › p16_images_for_eLife/p16_images_Ann_Chiao_for_eLife/Old SS-31 Treated/OSS_1/MS_1_p16_10x_k_RGB.jpg]

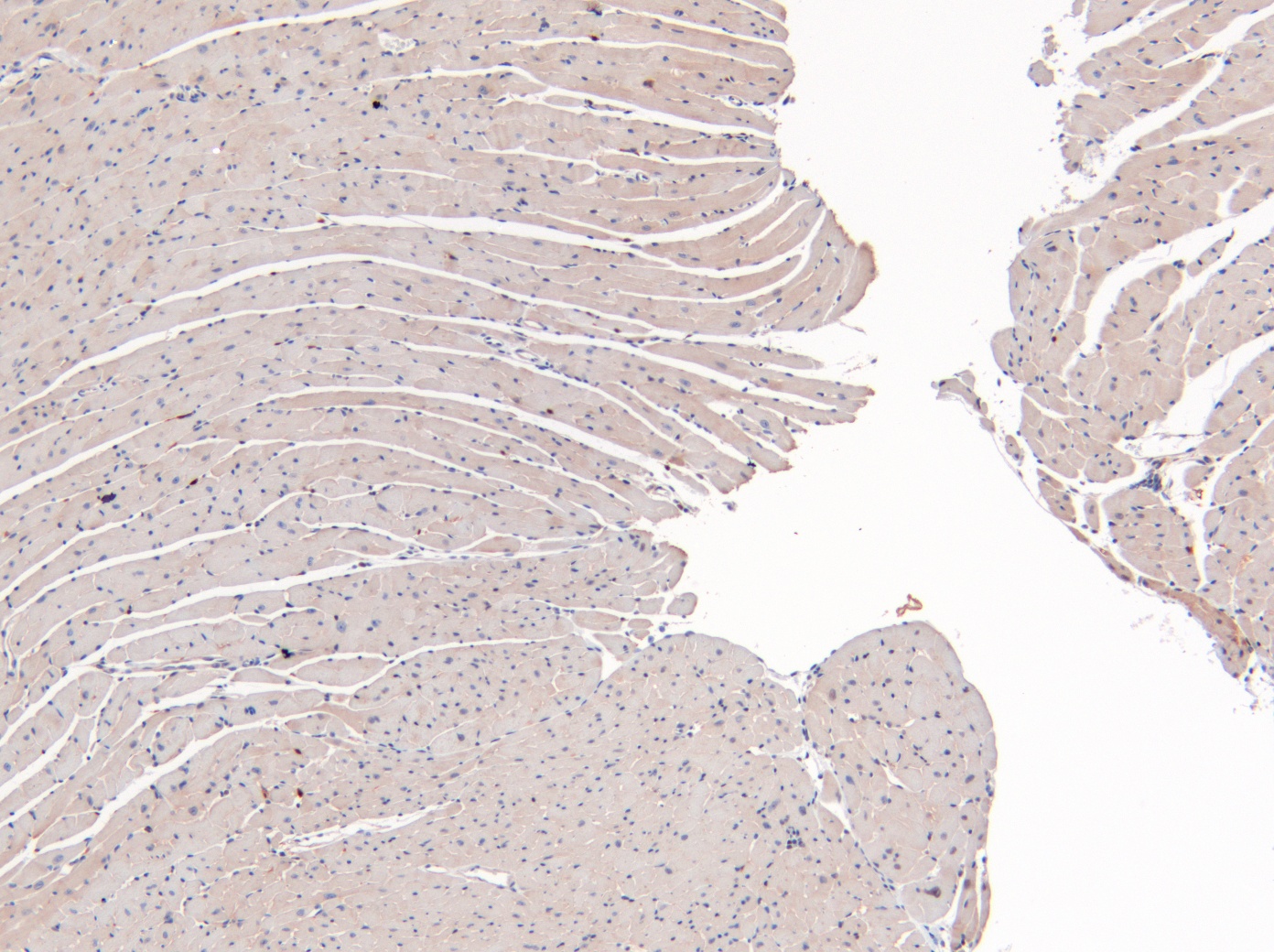

Supplement: Figure 4—source data 1. [file elife-55513-fig4-data1.zip › p16_images_for_eLife/p16_images_Ann_Chiao_for_eLife/Old SS-31 Treated/OSS_1/MS_1_p16_10x_l_RGB.jpg]

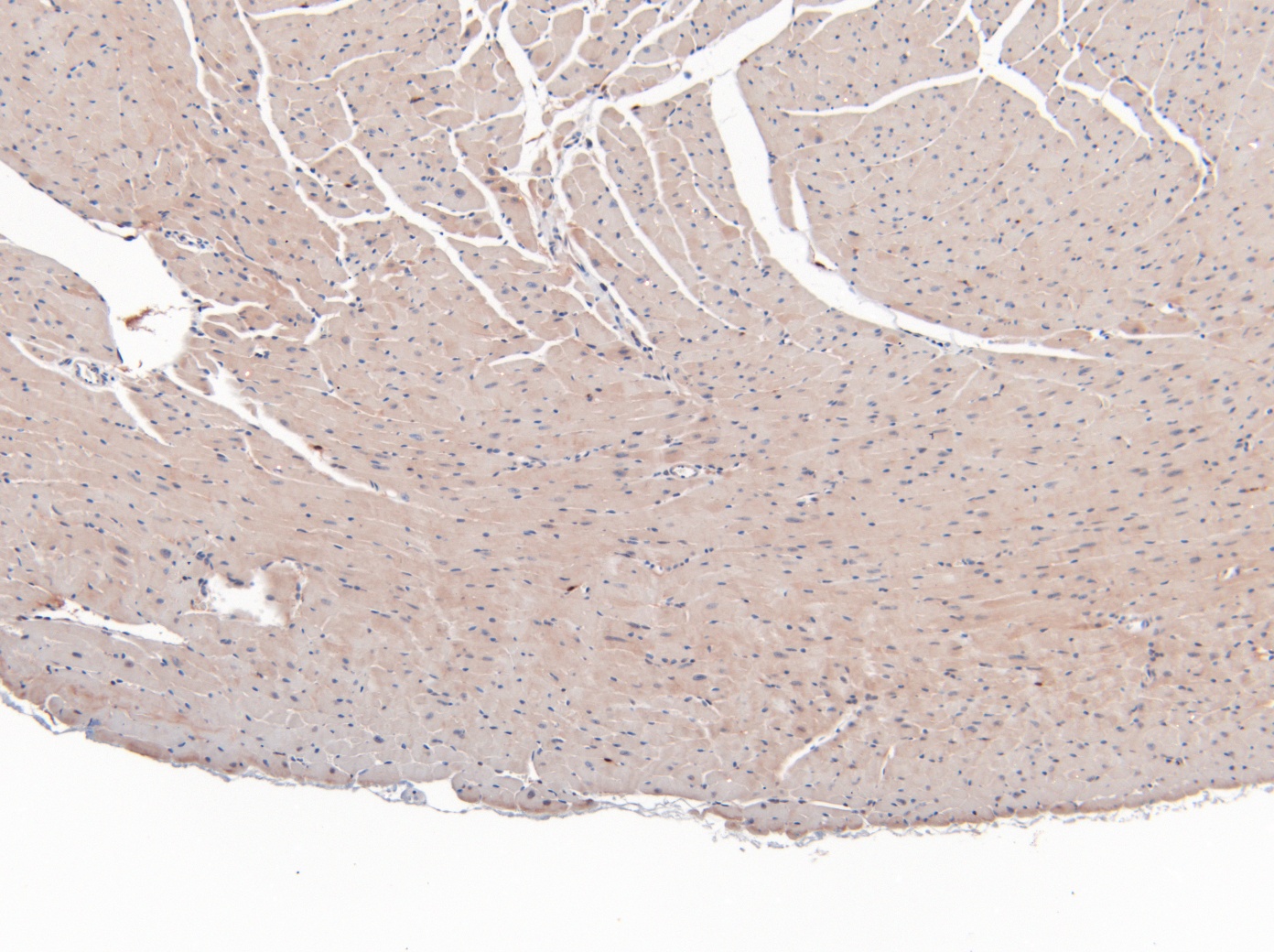

Supplement: Figure 4—source data 1. [file elife-55513-fig4-data1.zip › p16_images_for_eLife/p16_images_Ann_Chiao_for_eLife/Old SS-31 Treated/OSS_2/MS_3_p16_10x_a_RGB.jpg]

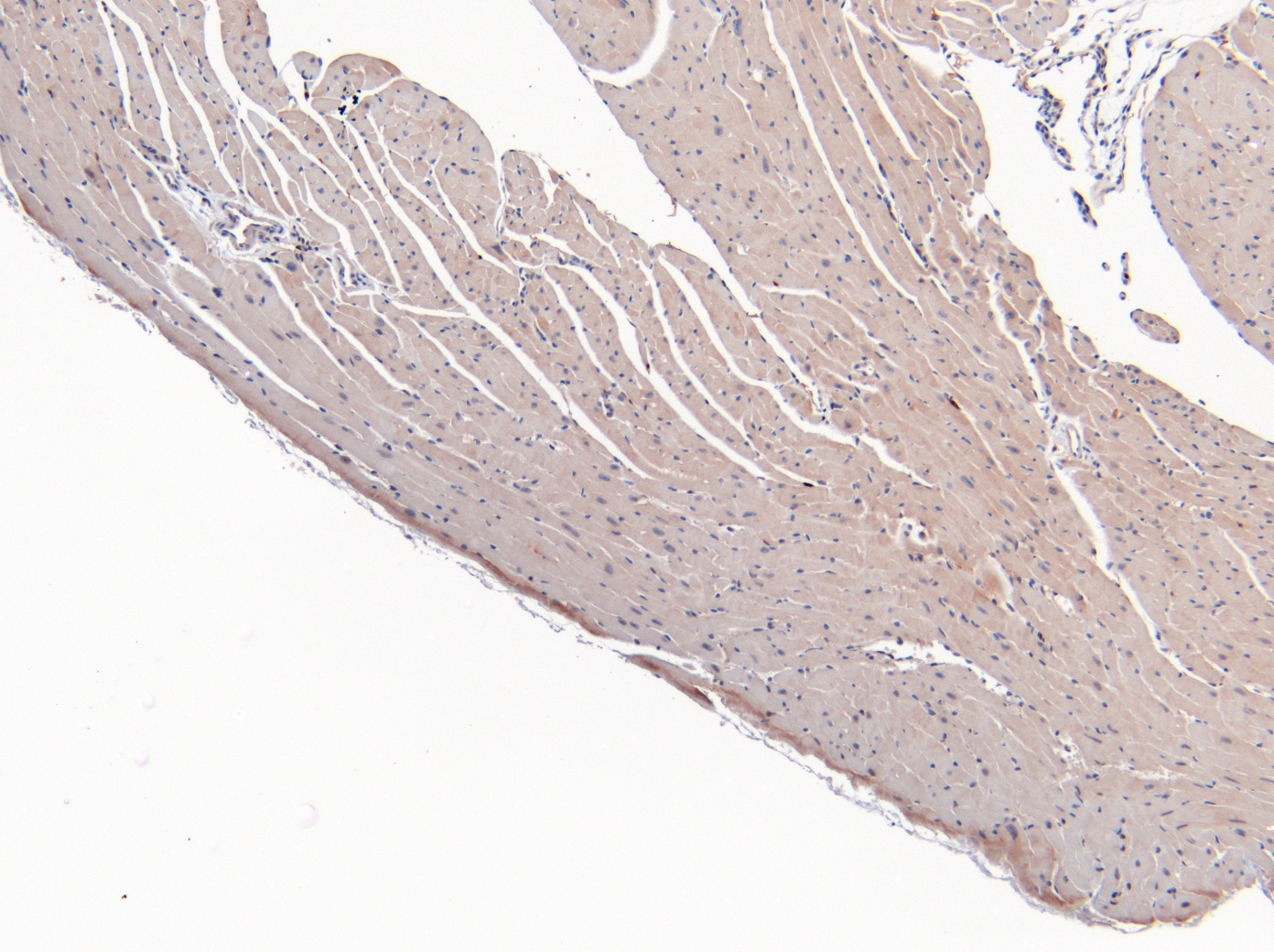

Supplement: Figure 4—source data 1. [file elife-55513-fig4-data1.zip › p16_images_for_eLife/p16_images_Ann_Chiao_for_eLife/Old SS-31 Treated/OSS_2/MS_3_p16_10x_b_RGB.jpg]

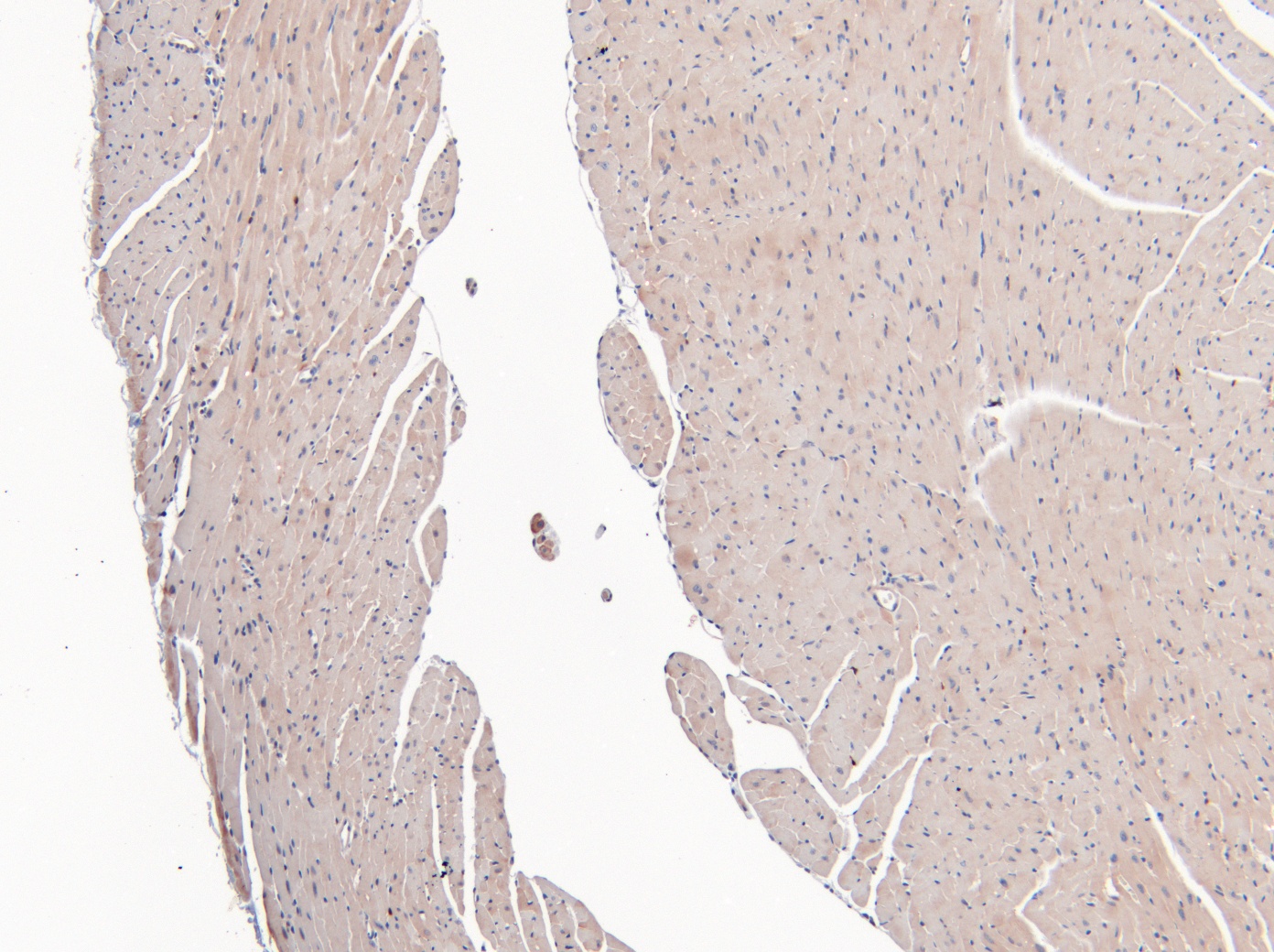

Supplement: Figure 4—source data 1. [file elife-55513-fig4-data1.zip › p16_images_for_eLife/p16_images_Ann_Chiao_for_eLife/Old SS-31 Treated/OSS_2/MS_3_p16_10x_c_RGB.jpg]

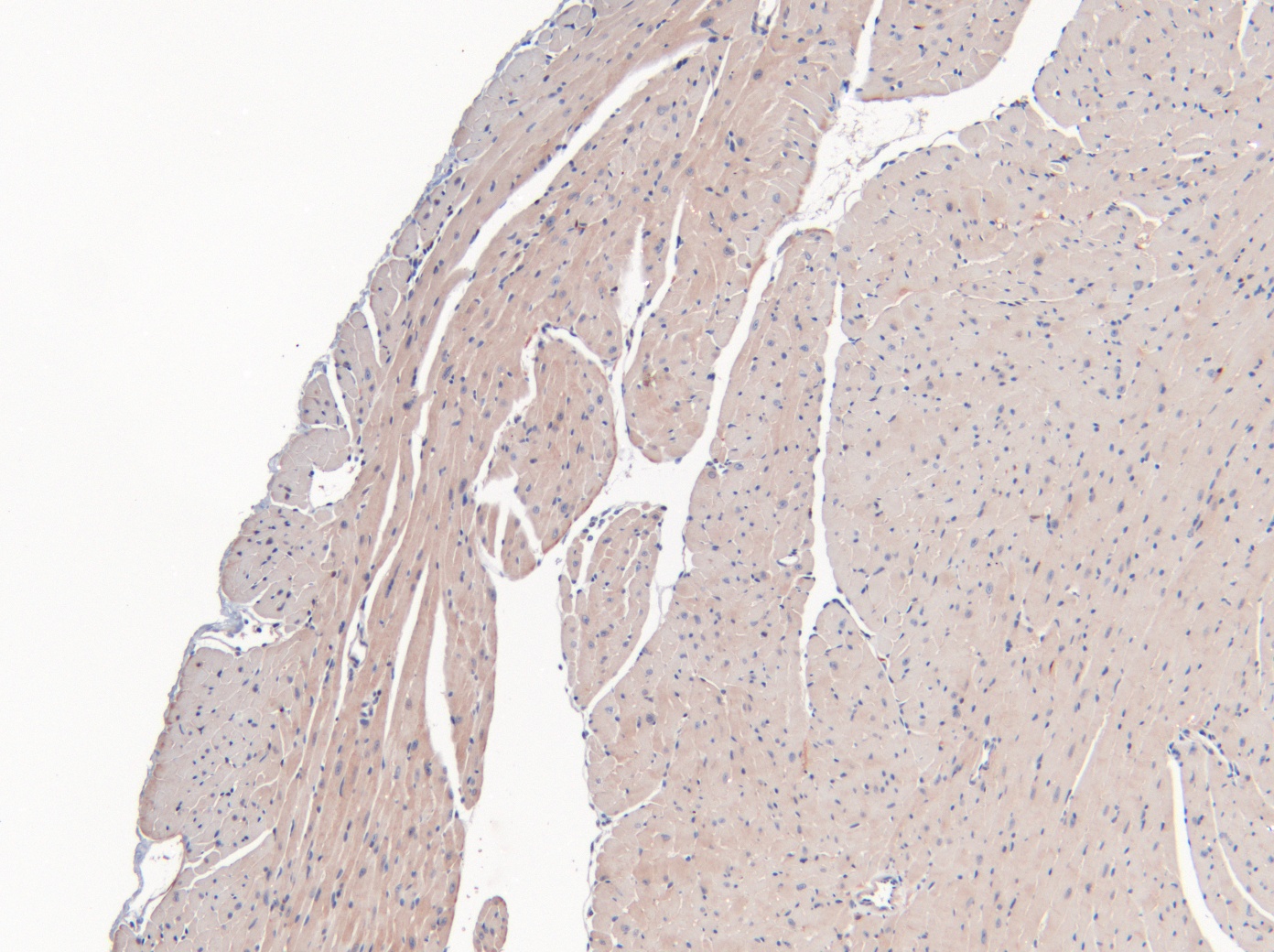

Supplement: Figure 4—source data 1. [file elife-55513-fig4-data1.zip › p16_images_for_eLife/p16_images_Ann_Chiao_for_eLife/Old SS-31 Treated/OSS_2/MS_3_p16_10x_d_RGB.jpg]

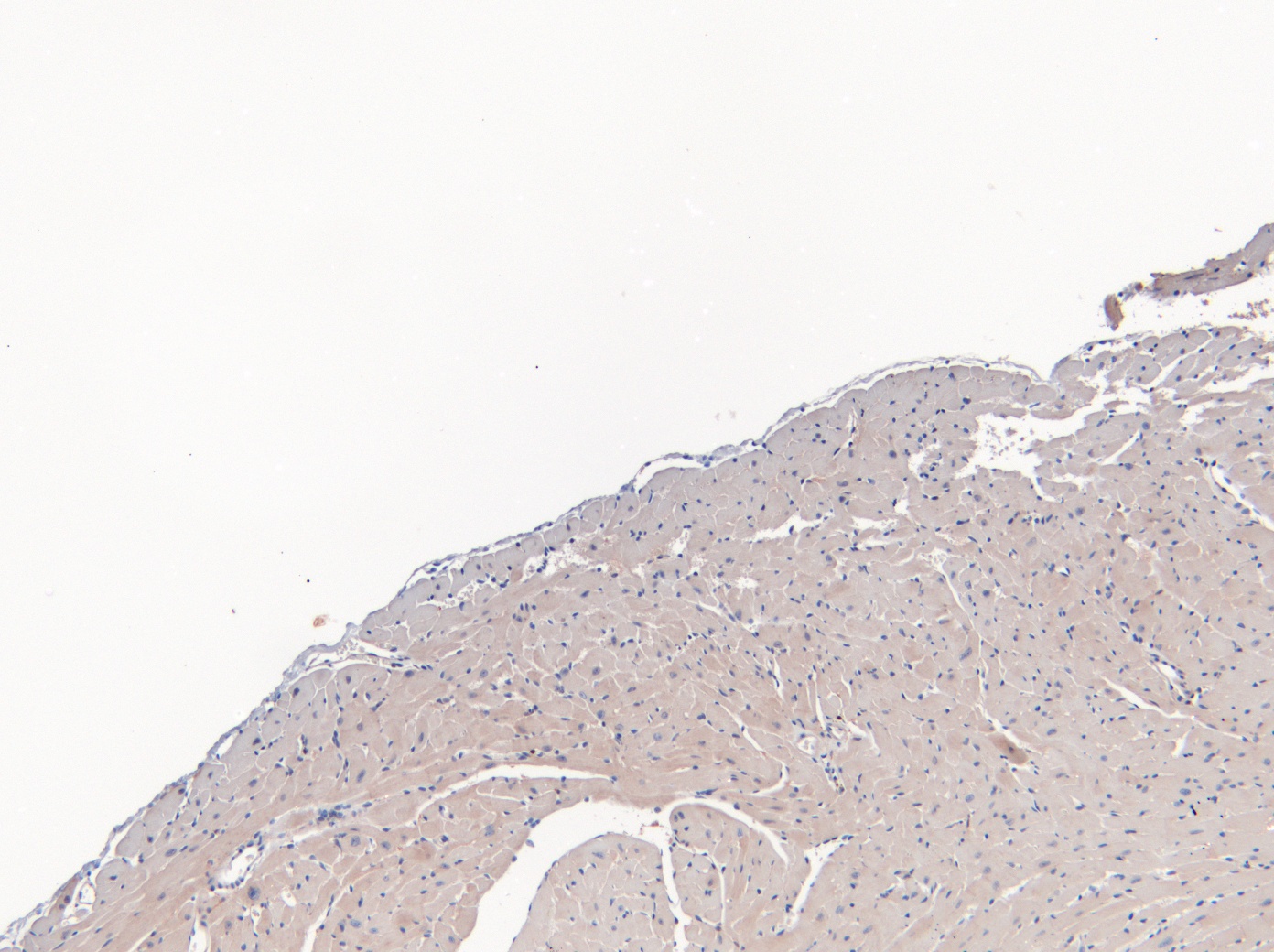

Supplement: Figure 4—source data 1. [file elife-55513-fig4-data1.zip › p16_images_for_eLife/p16_images_Ann_Chiao_for_eLife/Old SS-31 Treated/OSS_2/MS_3_p16_10x_e_RGB.jpg]

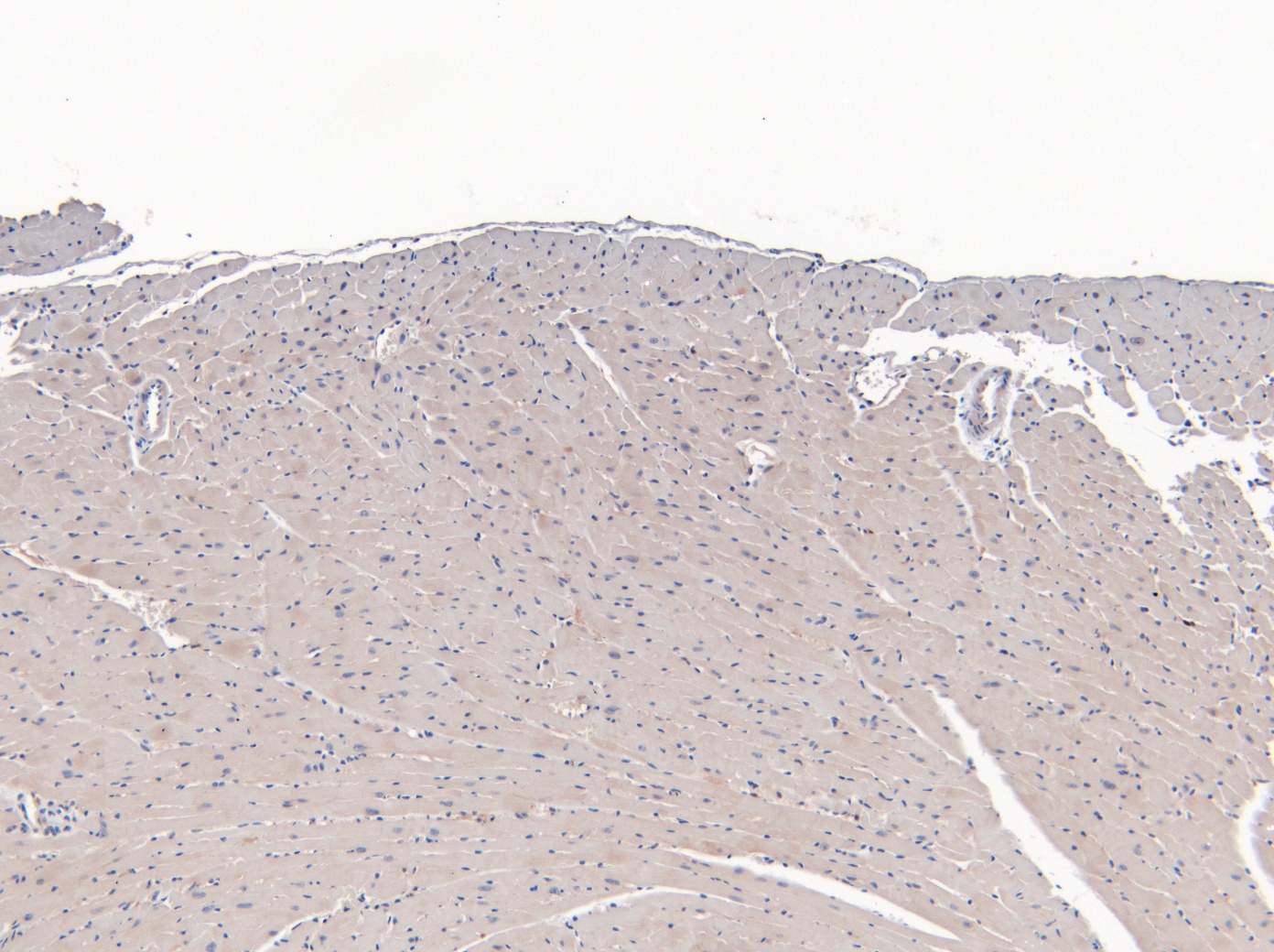

Supplement: Figure 4—source data 1. [file elife-55513-fig4-data1.zip › p16_images_for_eLife/p16_images_Ann_Chiao_for_eLife/Old SS-31 Treated/OSS_2/MS_3_p16_10x_f_RGB.jpg]

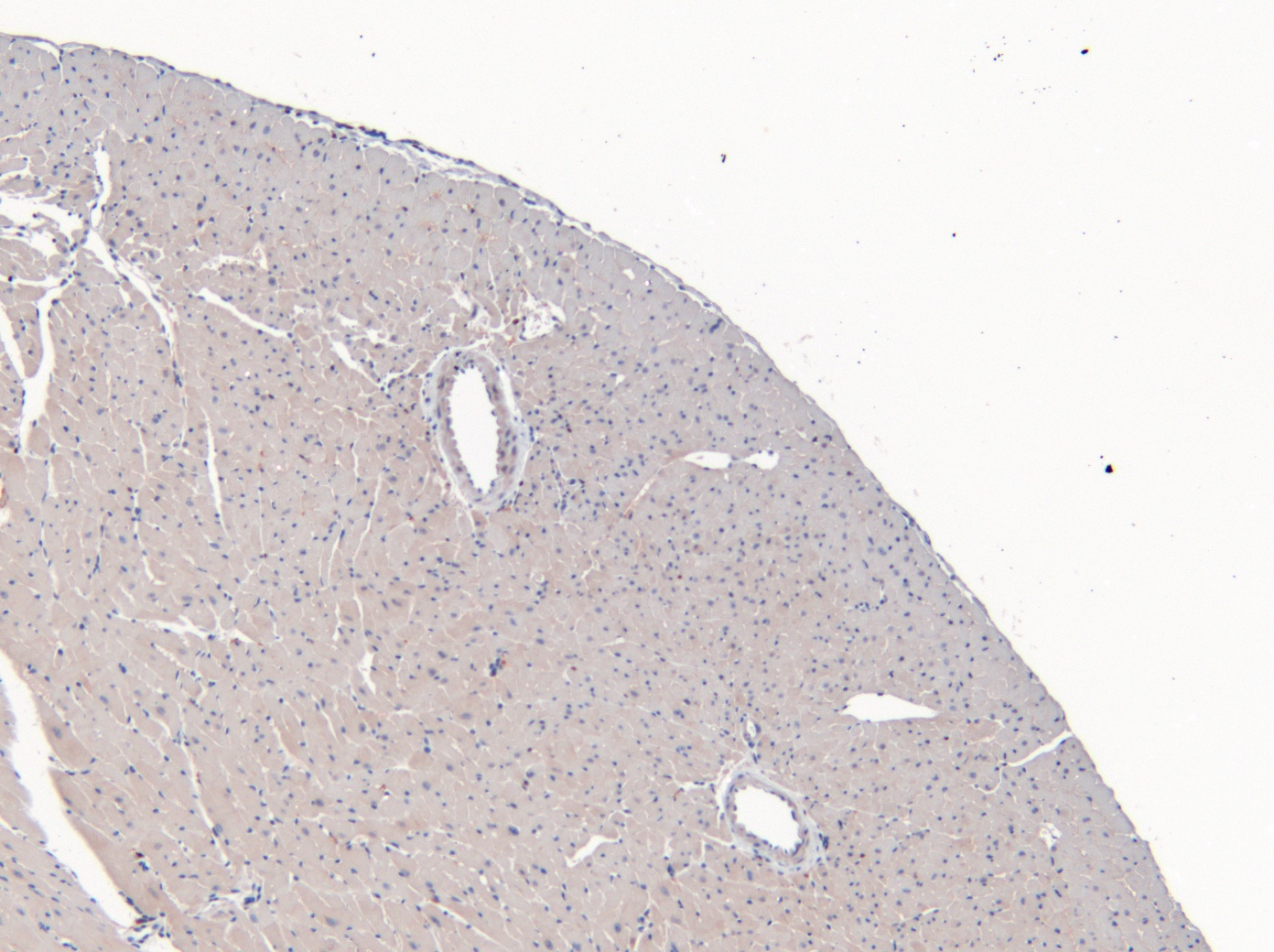

Supplement: Figure 4—source data 1. [file elife-55513-fig4-data1.zip › p16_images_for_eLife/p16_images_Ann_Chiao_for_eLife/Old SS-31 Treated/OSS_2/MS_3_p16_10x_g_RGB.jpg]

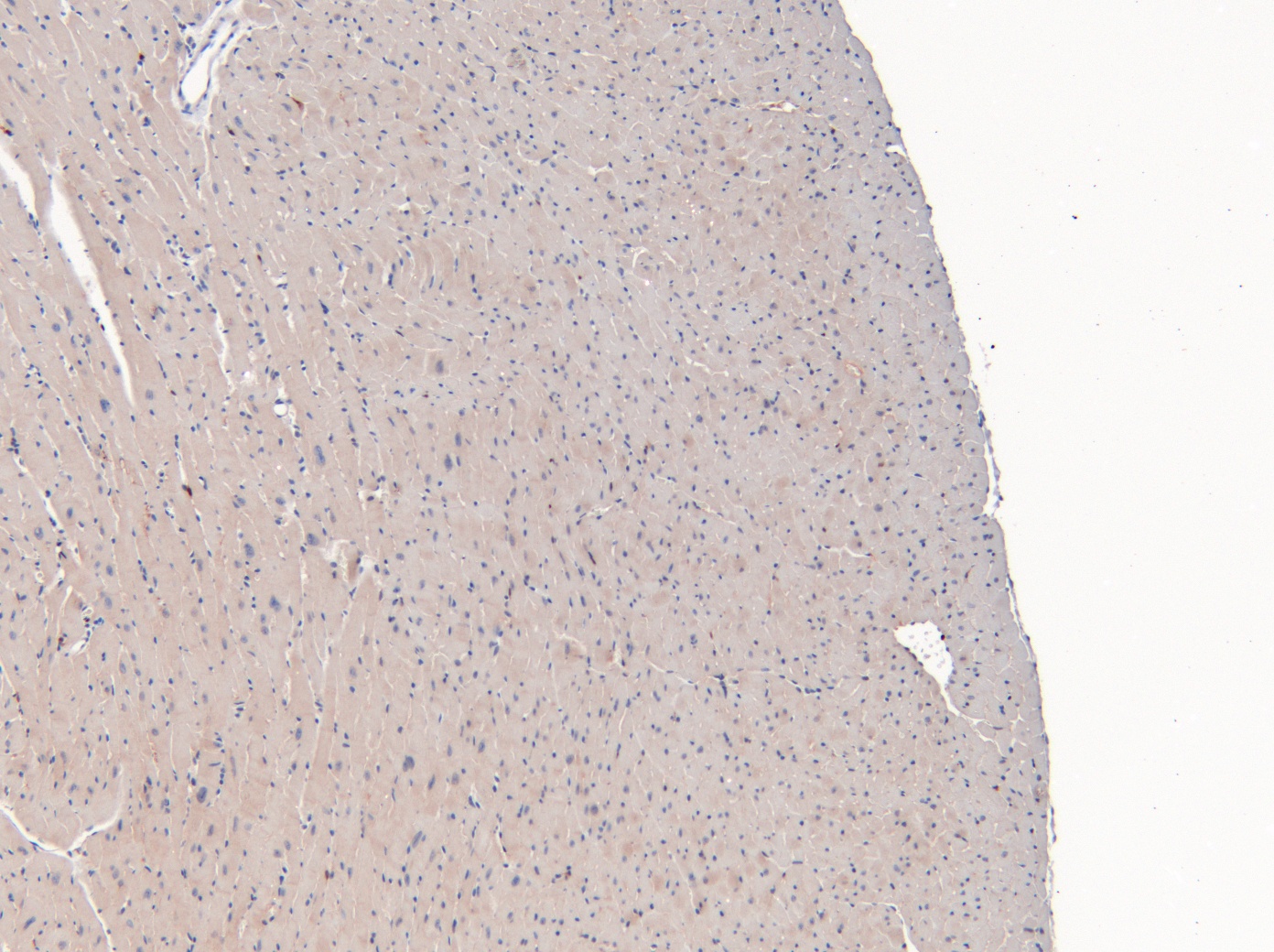

Supplement: Figure 4—source data 1. [file elife-55513-fig4-data1.zip › p16_images_for_eLife/p16_images_Ann_Chiao_for_eLife/Old SS-31 Treated/OSS_2/MS_3_p16_10x_h_RGB.jpg]

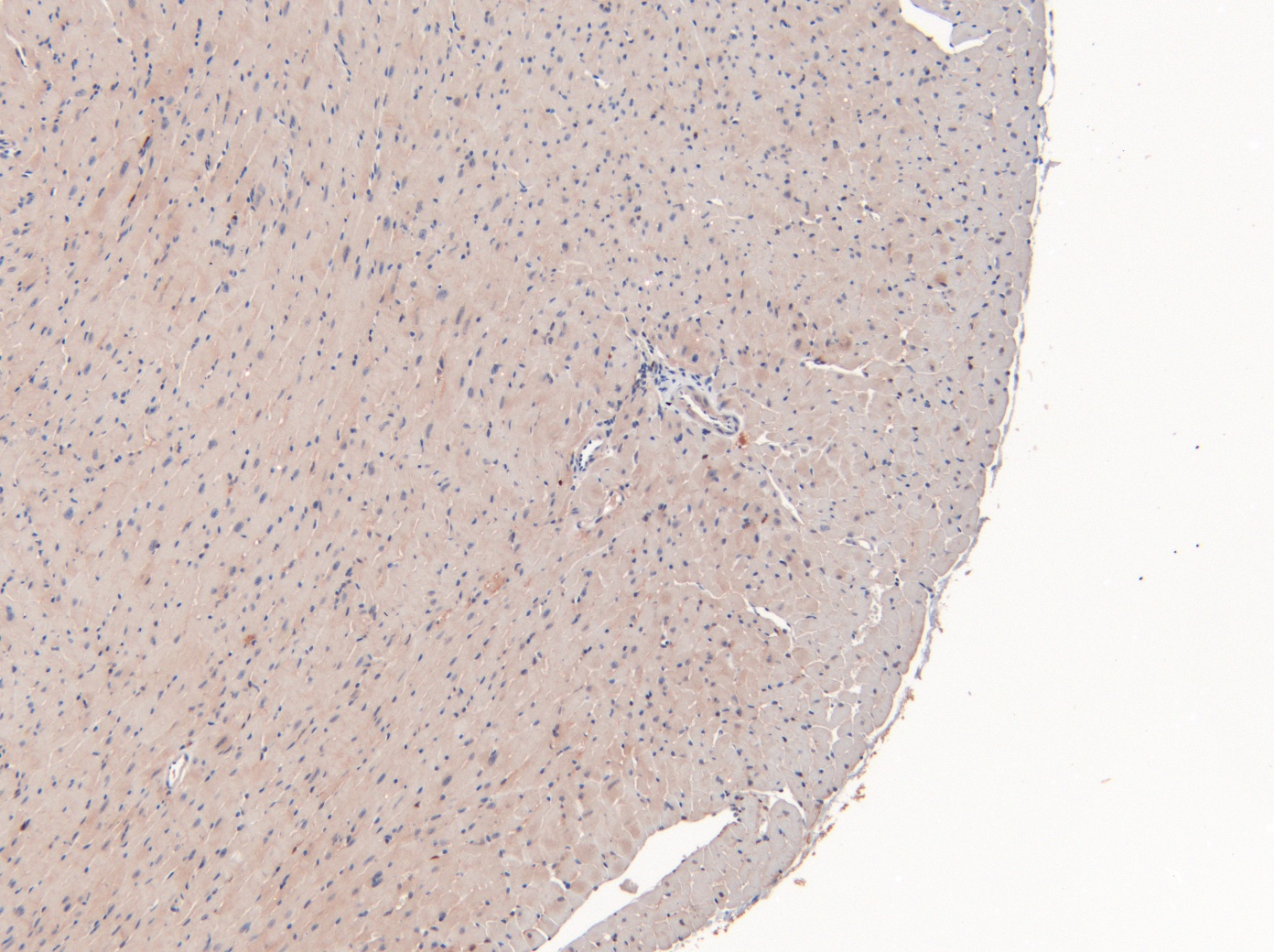

Supplement: Figure 4—source data 1. [file elife-55513-fig4-data1.zip › p16_images_for_eLife/p16_images_Ann_Chiao_for_eLife/Old SS-31 Treated/OSS_2/MS_3_p16_10x_i_RGB.jpg]

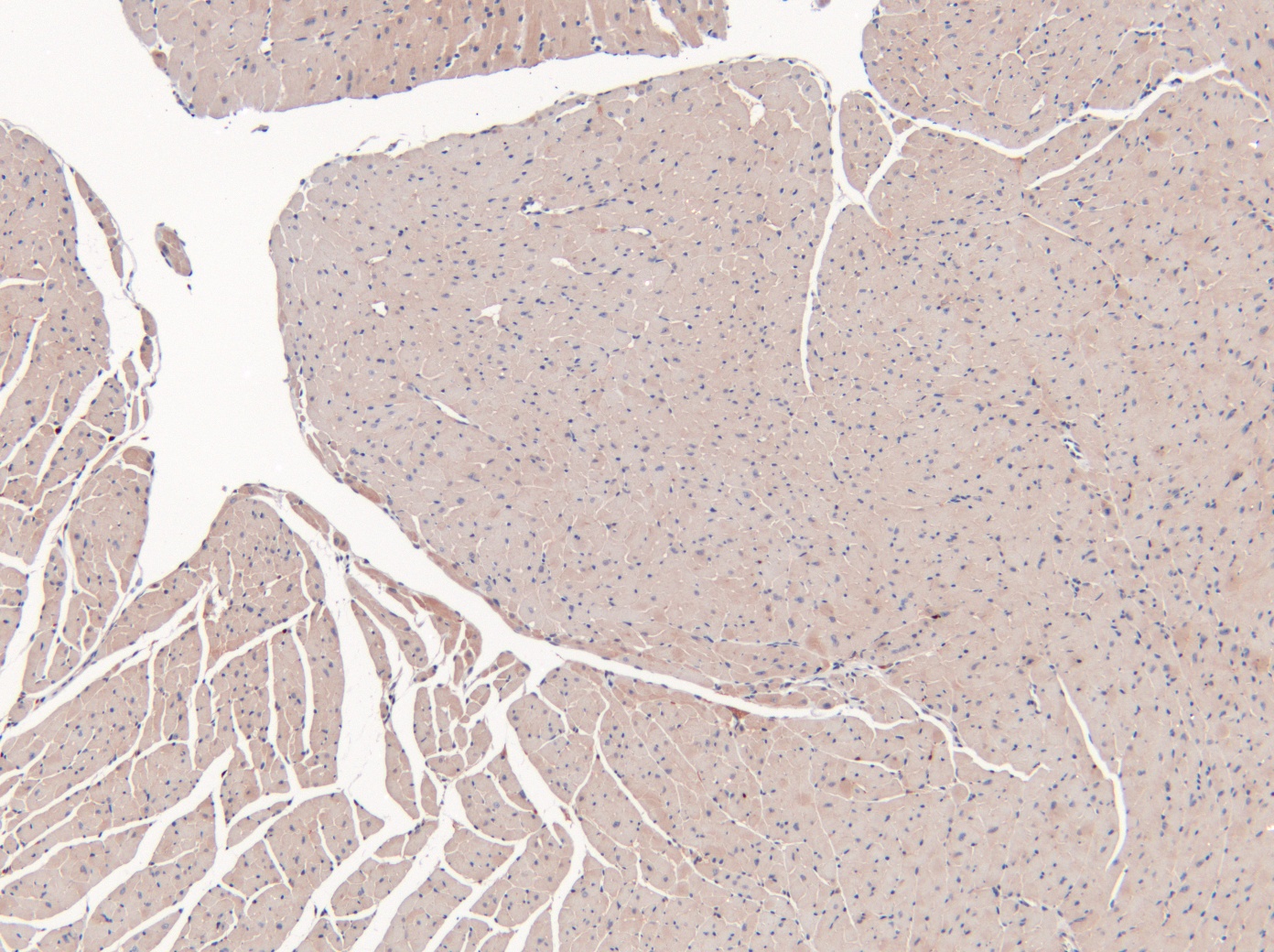

Supplement: Figure 4—source data 1. [file elife-55513-fig4-data1.zip › p16_images_for_eLife/p16_images_Ann_Chiao_for_eLife/Old SS-31 Treated/OSS_2/MS_3_p16_10x_j_RGB.jpg]

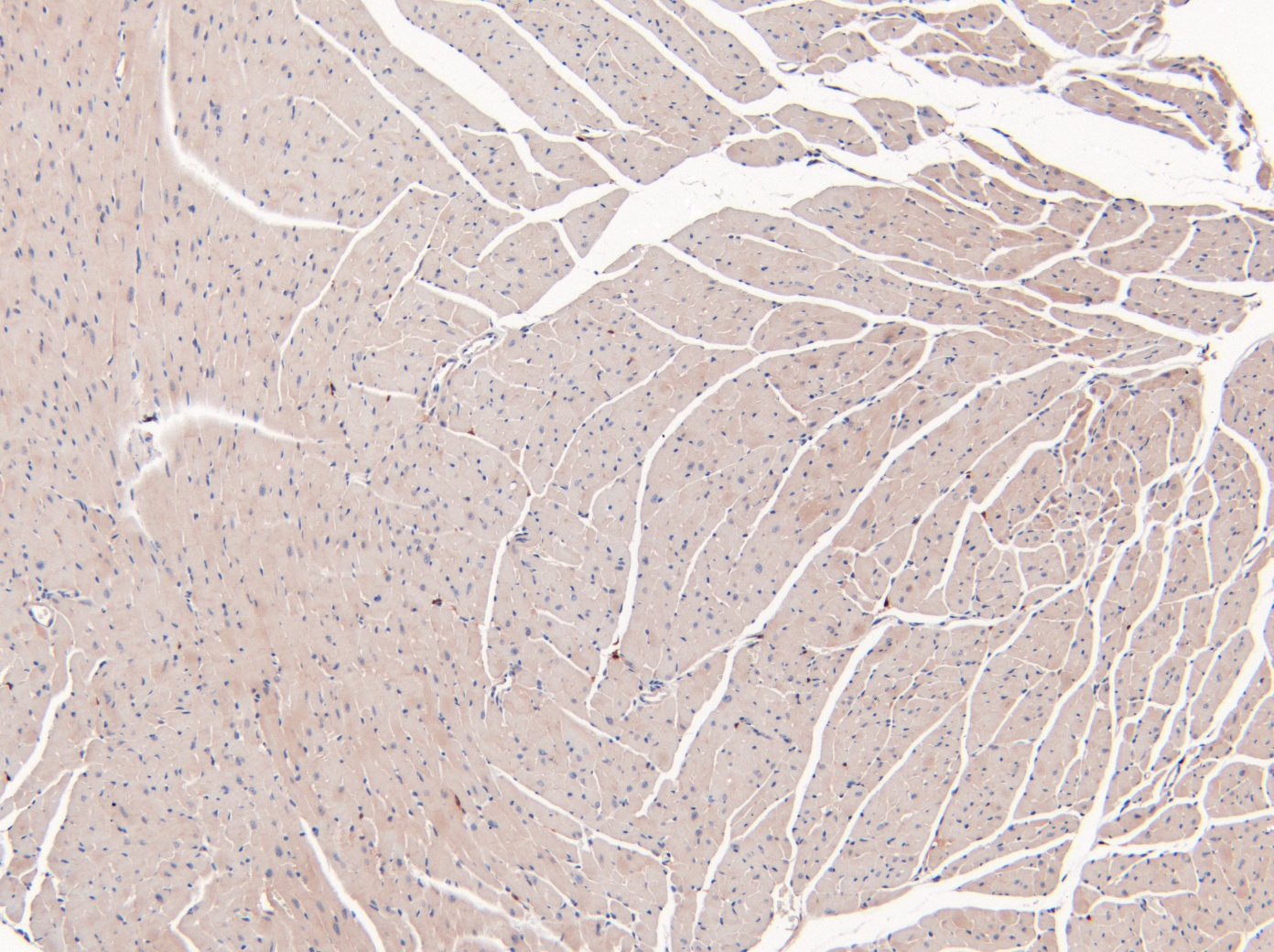

Supplement: Figure 4—source data 1. [file elife-55513-fig4-data1.zip › p16_images_for_eLife/p16_images_Ann_Chiao_for_eLife/Old SS-31 Treated/OSS_2/MS_3_p16_10x_k_RGB.jpg]

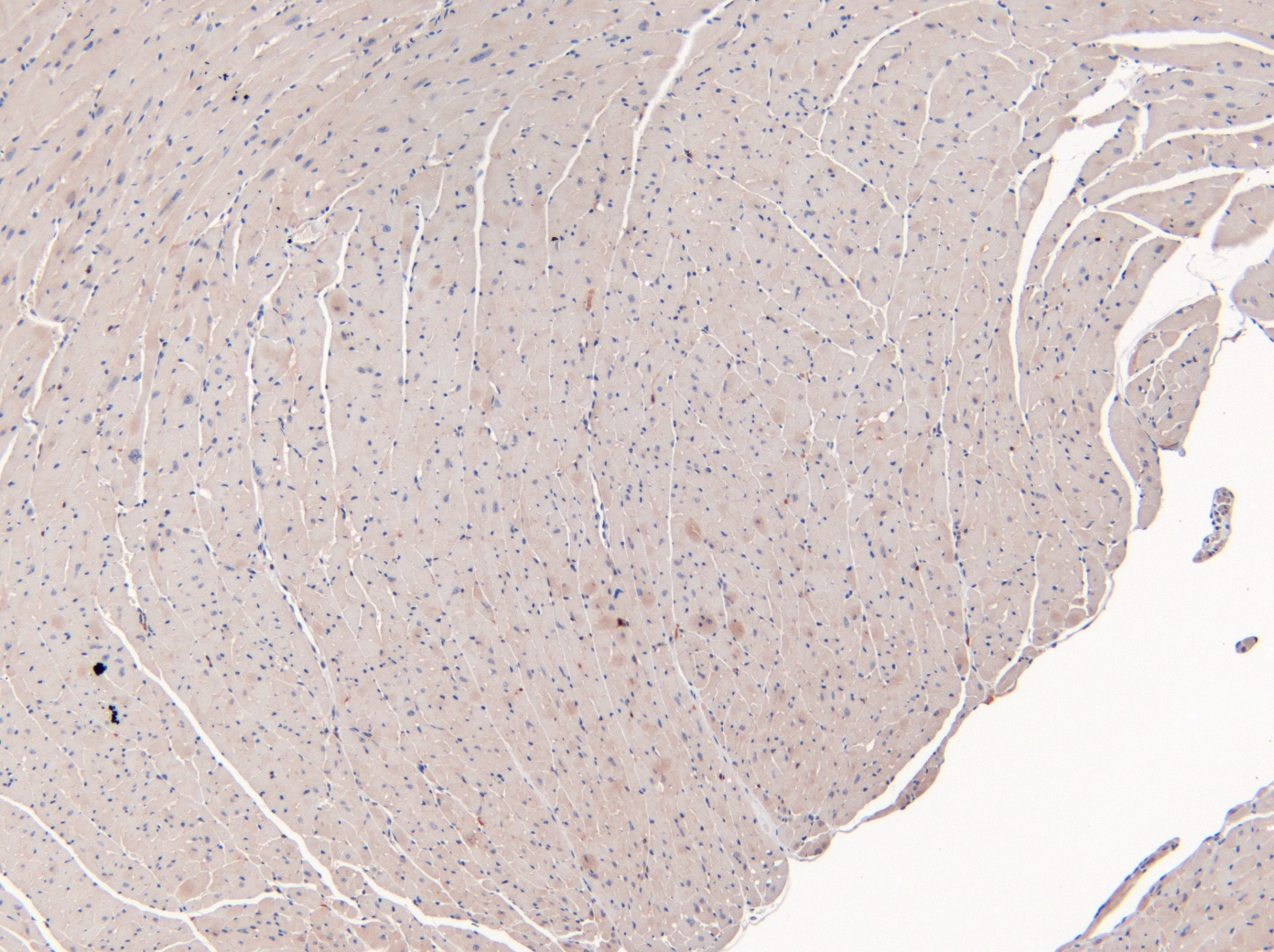

Supplement: Figure 4—source data 1. [file elife-55513-fig4-data1.zip › p16_images_for_eLife/p16_images_Ann_Chiao_for_eLife/Old SS-31 Treated/OSS_2/MS_3_p16_10x_l_RGB.jpg]

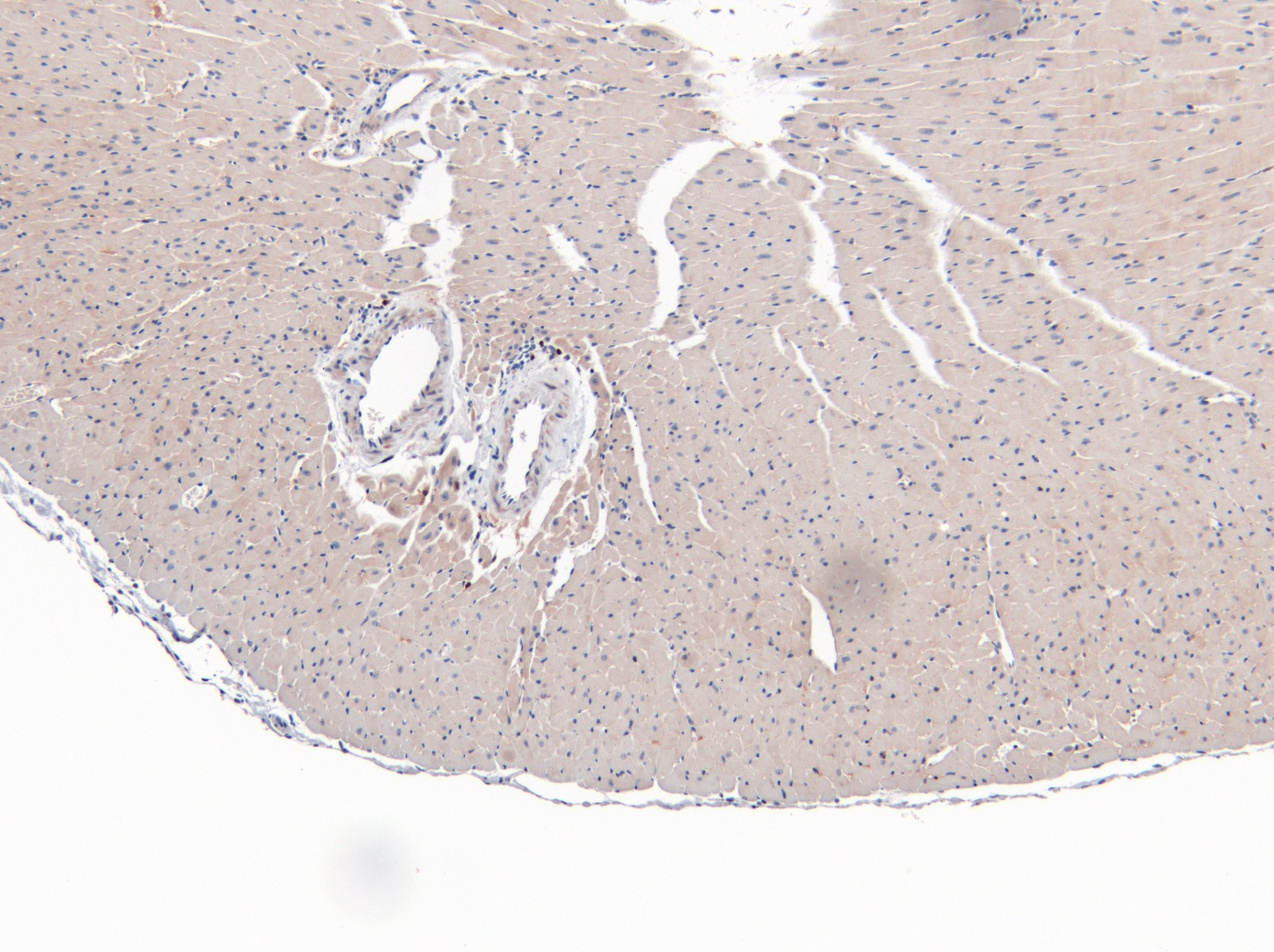

Supplement: Figure 4—source data 1. [file elife-55513-fig4-data1.zip › p16_images_for_eLife/p16_images_Ann_Chiao_for_eLife/Old SS-31 Treated/OSS_3/MS_6_p16_10x_a_RGB.jpg]

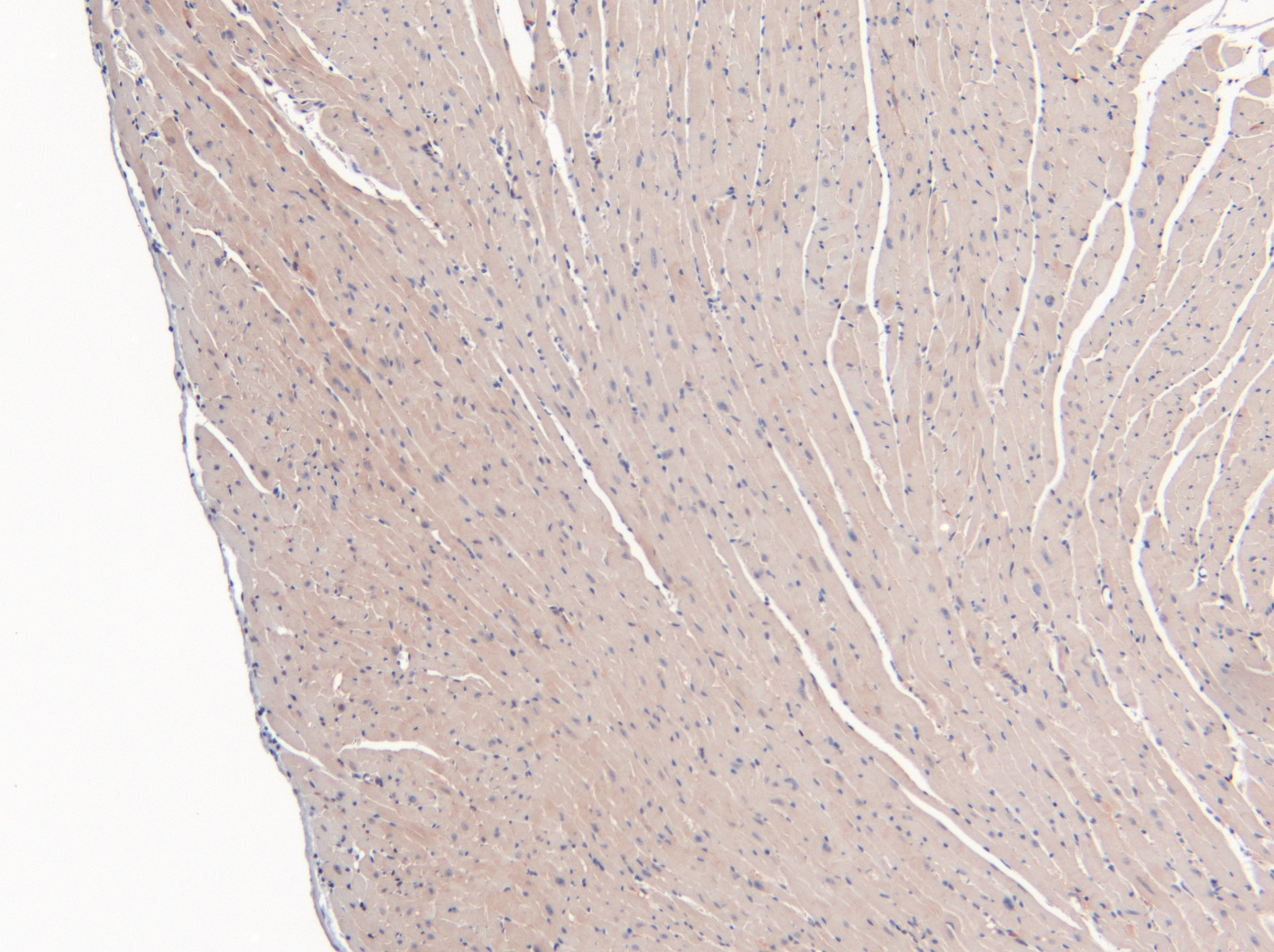

Supplement: Figure 4—source data 1. [file elife-55513-fig4-data1.zip › p16_images_for_eLife/p16_images_Ann_Chiao_for_eLife/Old SS-31 Treated/OSS_3/MS_6_p16_10x_b_RGB.jpg]

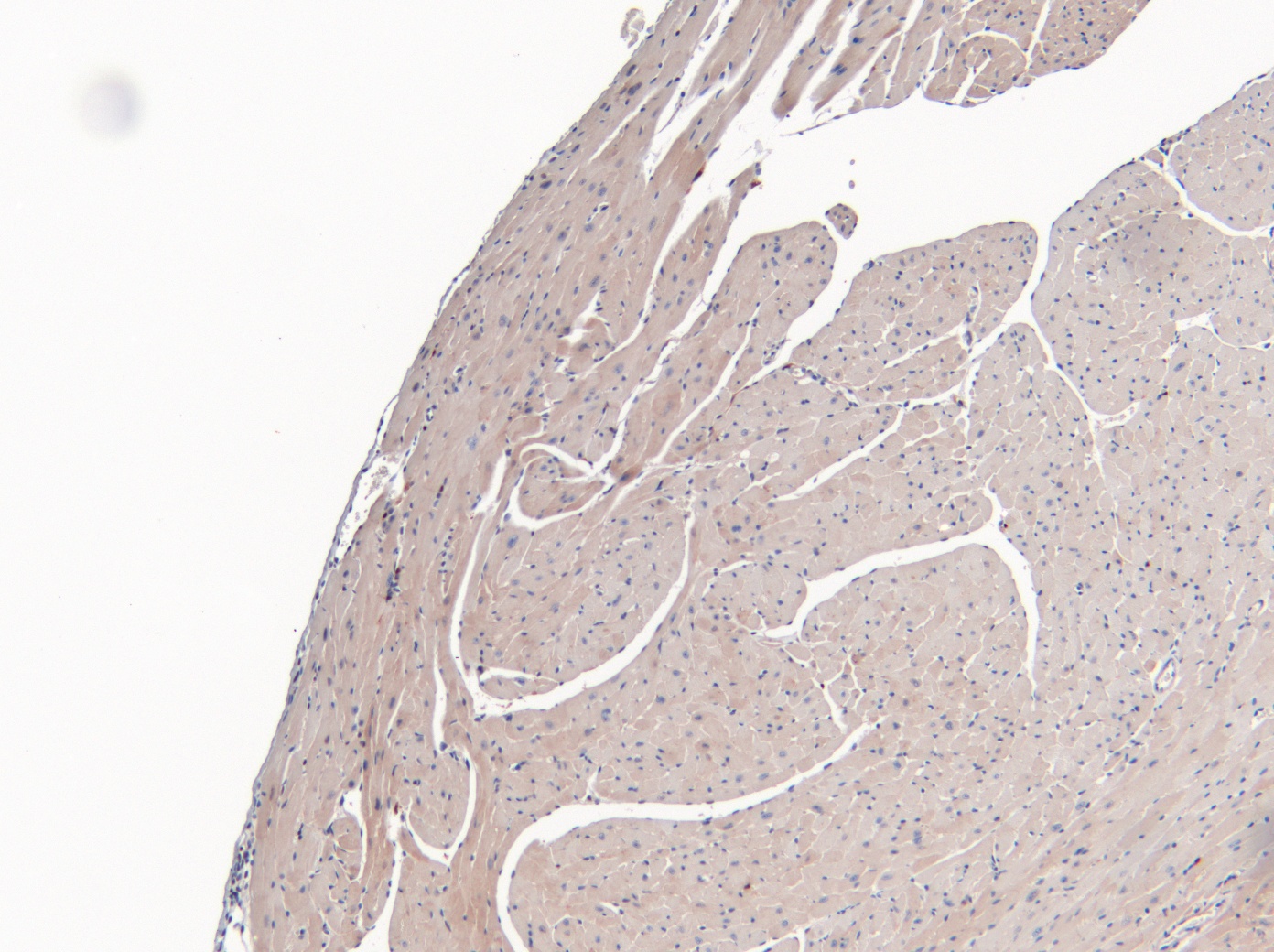

Supplement: Figure 4—source data 1. [file elife-55513-fig4-data1.zip › p16_images_for_eLife/p16_images_Ann_Chiao_for_eLife/Old SS-31 Treated/OSS_3/MS_6_p16_10x_c_RGB.jpg]

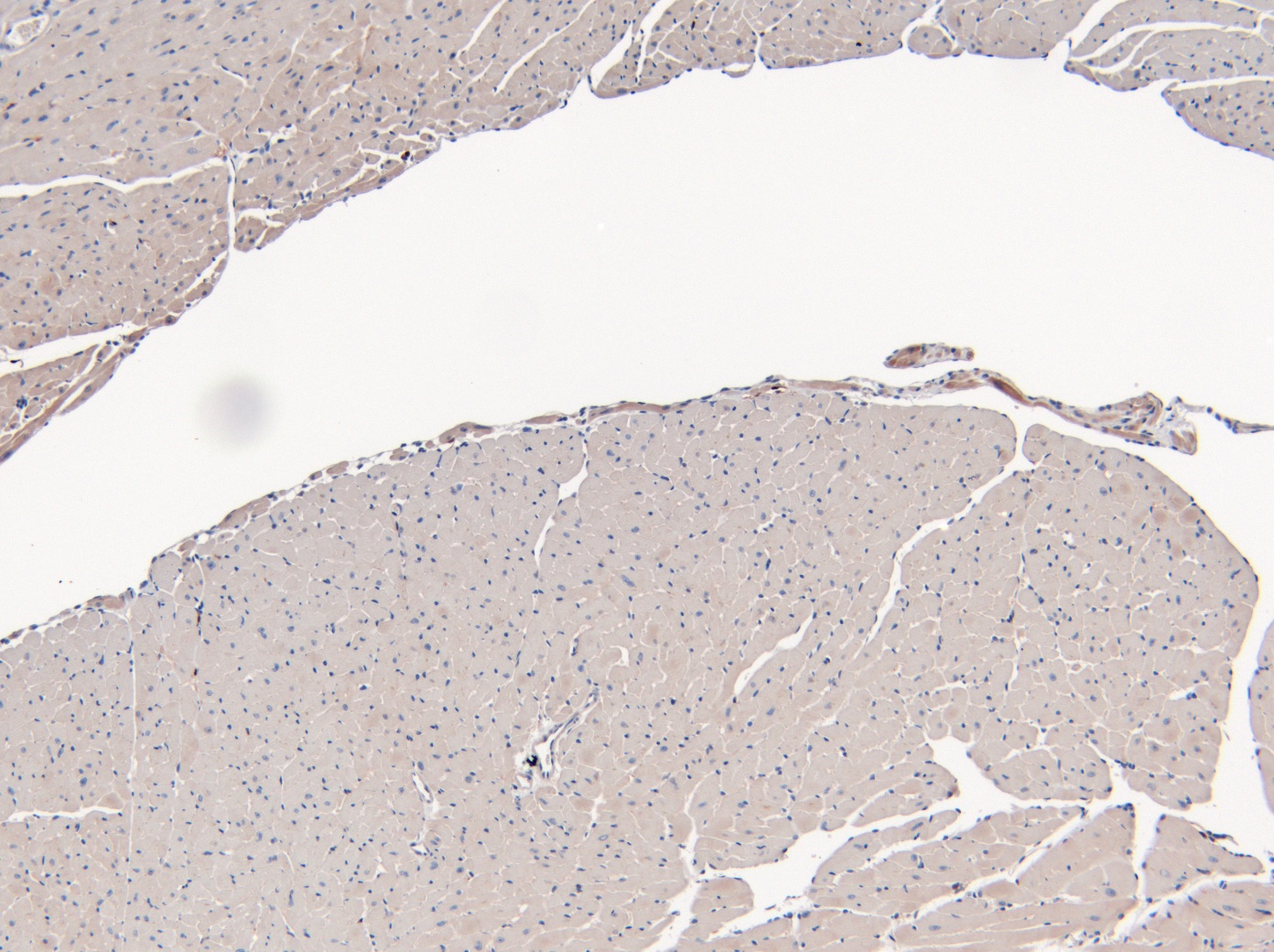

Supplement: Figure 4—source data 1. [file elife-55513-fig4-data1.zip › p16_images_for_eLife/p16_images_Ann_Chiao_for_eLife/Old SS-31 Treated/OSS_3/MS_6_p16_10x_d_RGB.jpg]

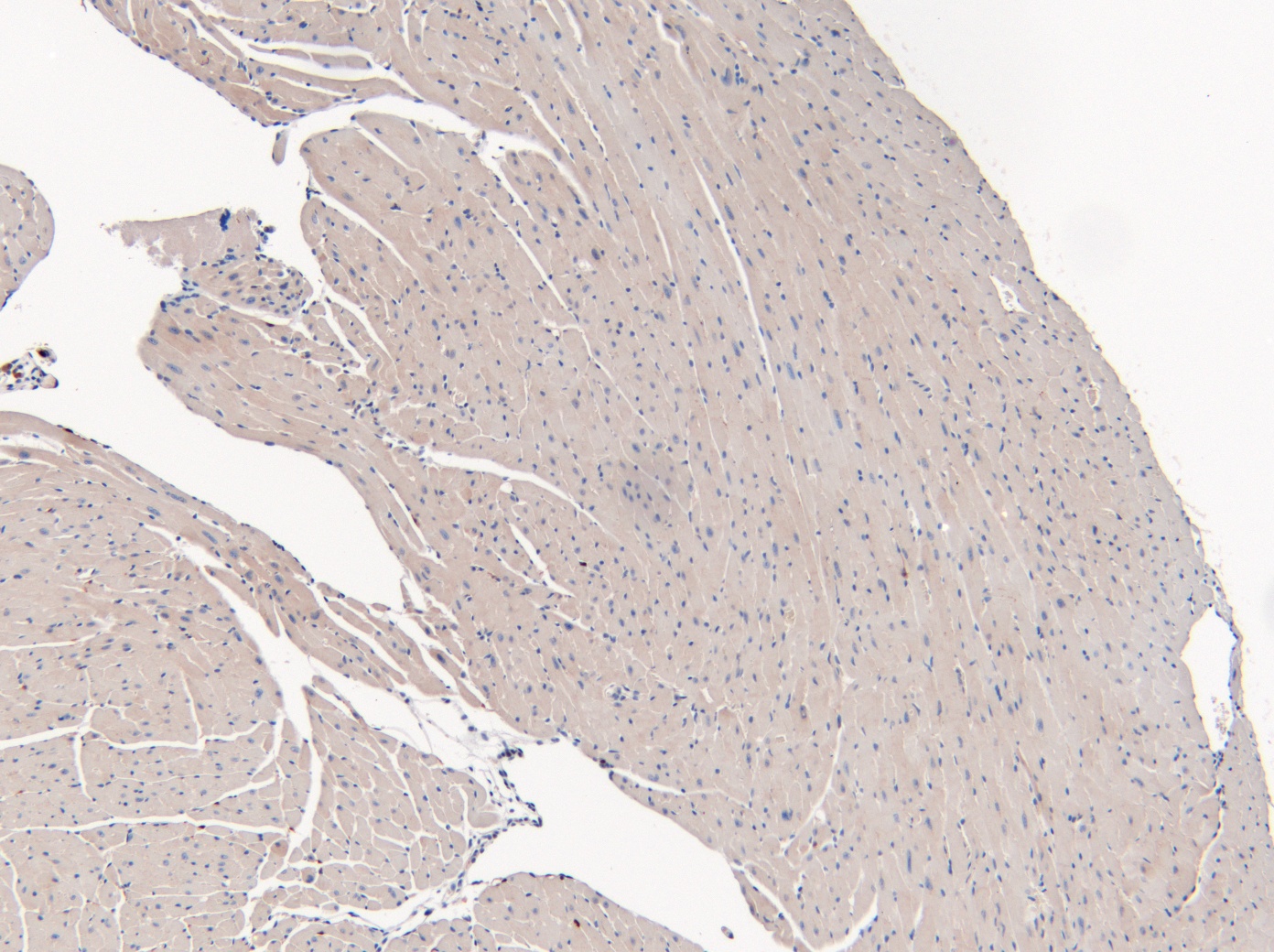

Supplement: Figure 4—source data 1. [file elife-55513-fig4-data1.zip › p16_images_for_eLife/p16_images_Ann_Chiao_for_eLife/Old SS-31 Treated/OSS_3/MS_6_p16_10x_e_RGB.jpg]

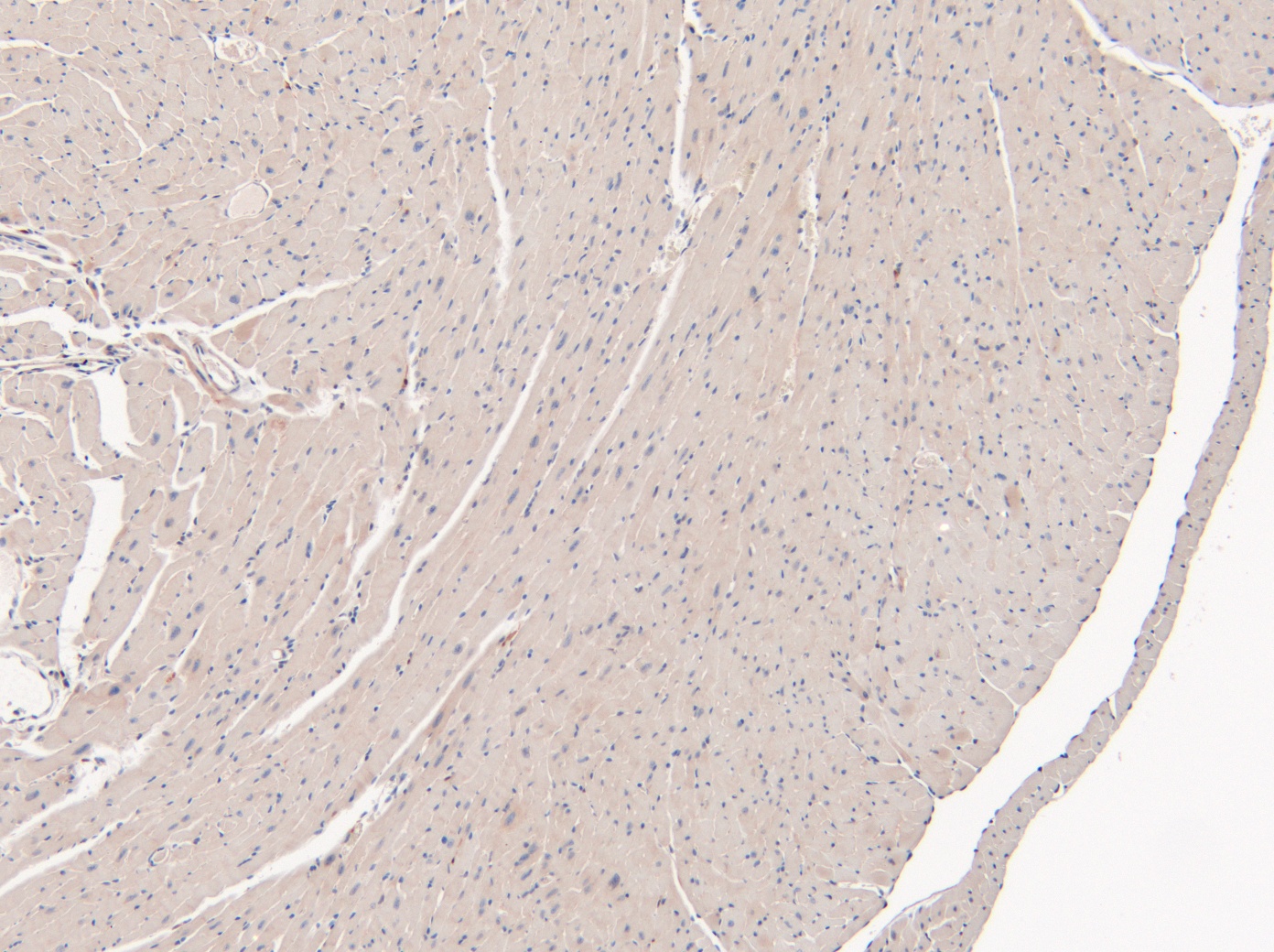

Supplement: Figure 4—source data 1. [file elife-55513-fig4-data1.zip › p16_images_for_eLife/p16_images_Ann_Chiao_for_eLife/Old SS-31 Treated/OSS_3/MS_6_p16_10x_f_RGB.jpg]

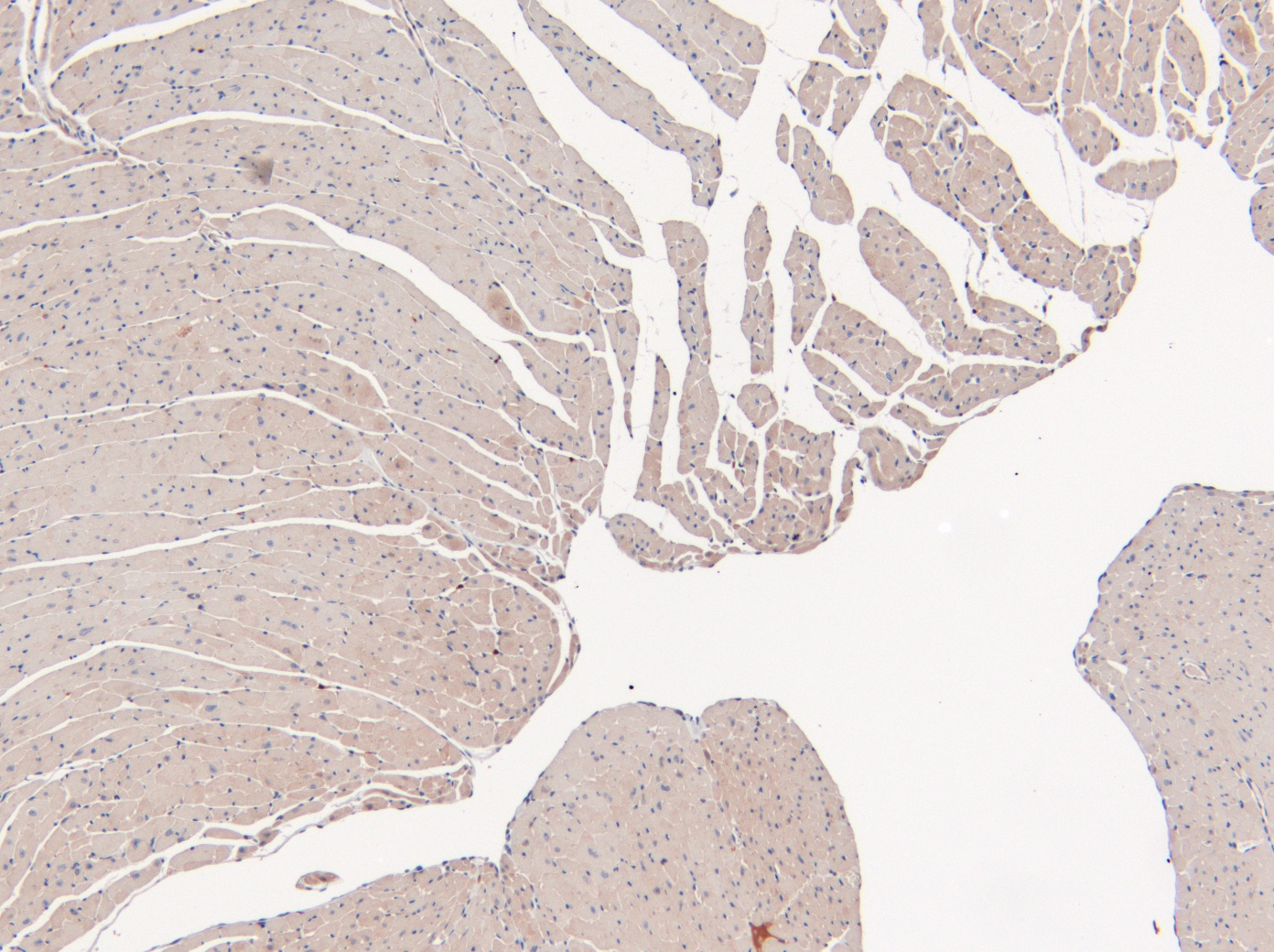

Supplement: Figure 4—source data 1. [file elife-55513-fig4-data1.zip › p16_images_for_eLife/p16_images_Ann_Chiao_for_eLife/Old SS-31 Treated/OSS_3/MS_6_p16_10x_g_RGB.jpg]

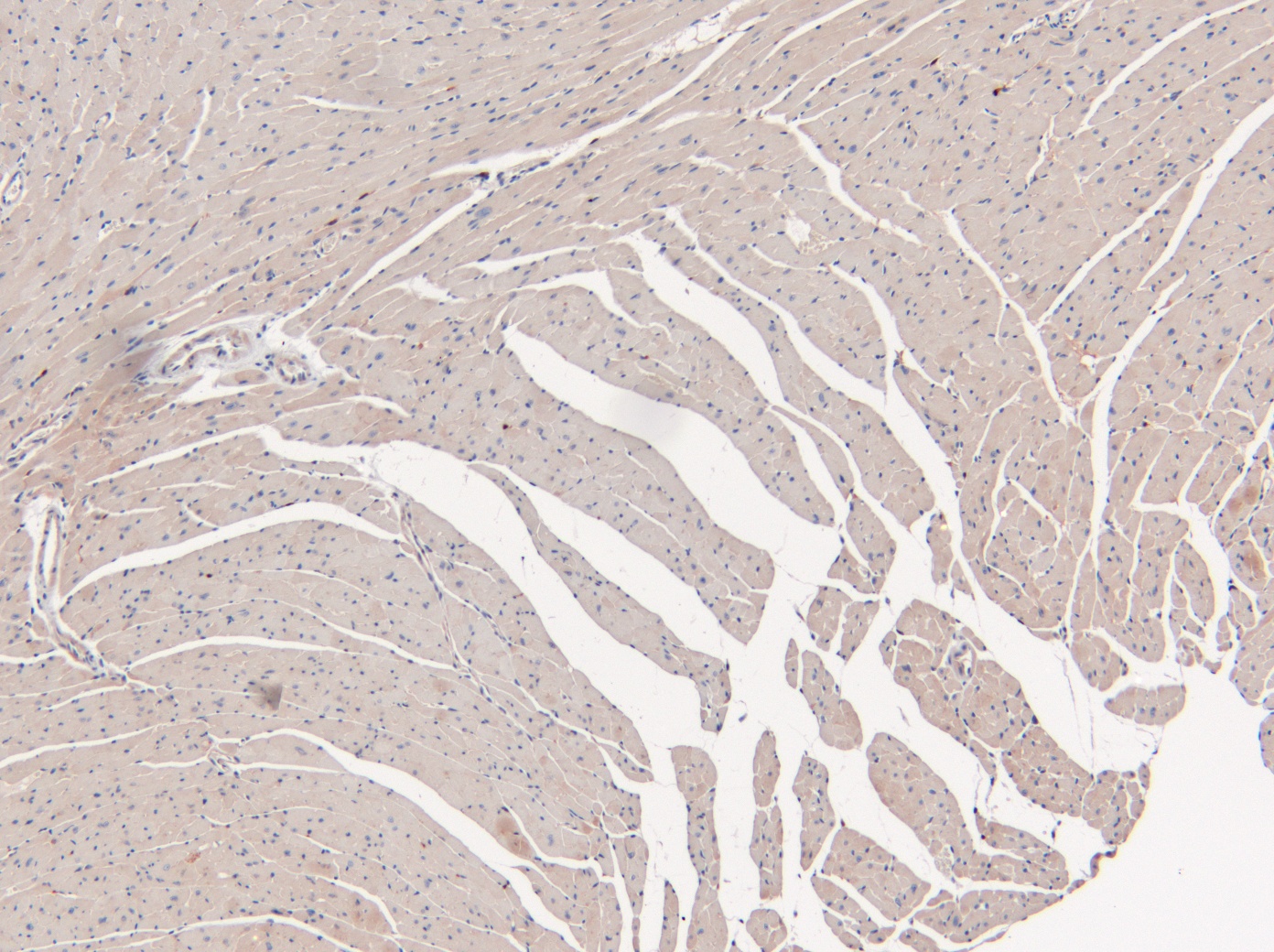

Supplement: Figure 4—source data 1. [file elife-55513-fig4-data1.zip › p16_images_for_eLife/p16_images_Ann_Chiao_for_eLife/Old SS-31 Treated/OSS_3/MS_6_p16_10x_h_RGB.jpg]

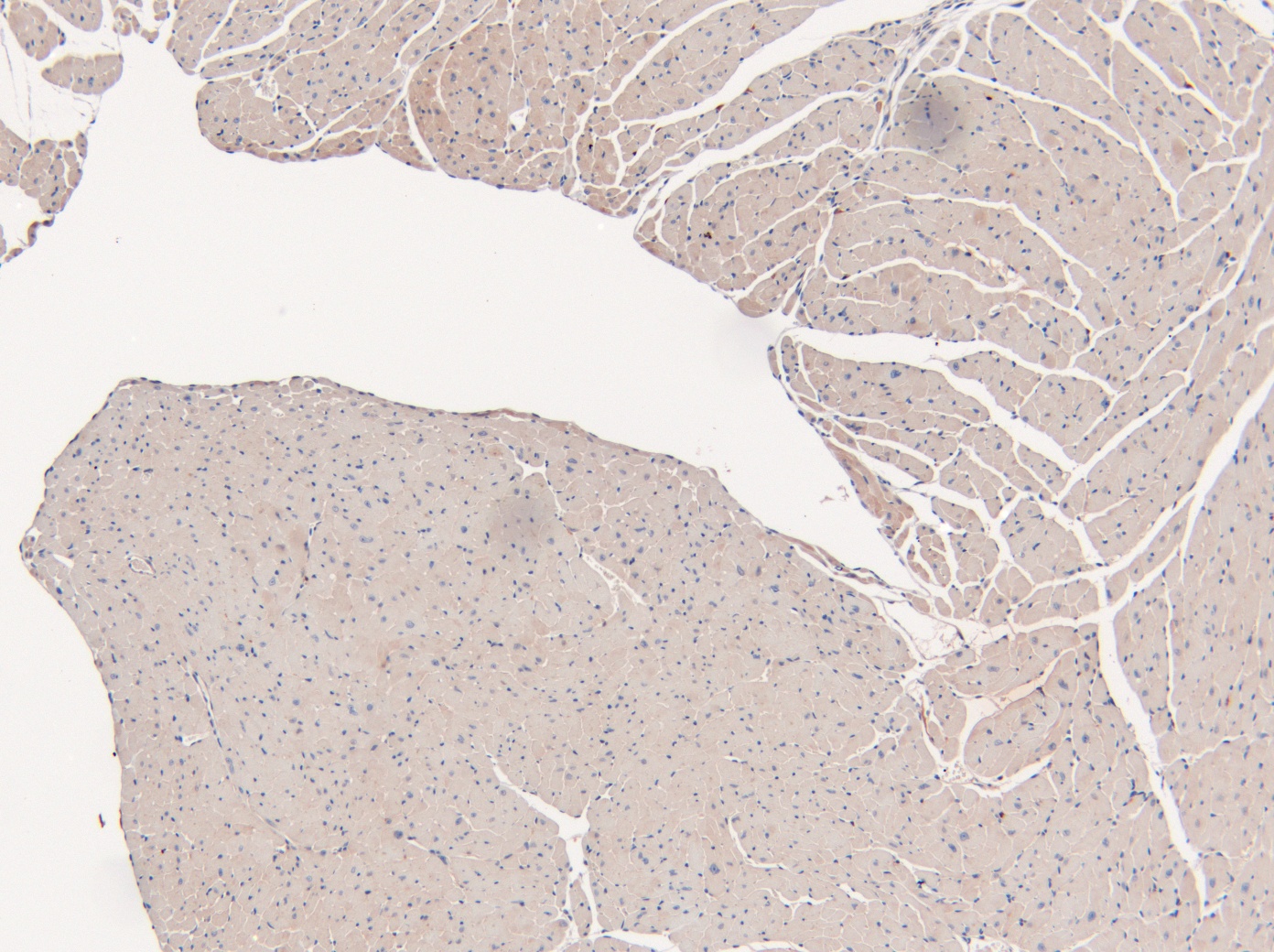

Supplement: Figure 4—source data 1. [file elife-55513-fig4-data1.zip › p16_images_for_eLife/p16_images_Ann_Chiao_for_eLife/Old SS-31 Treated/OSS_3/MS_6_p16_10x_i_RGB.jpg]

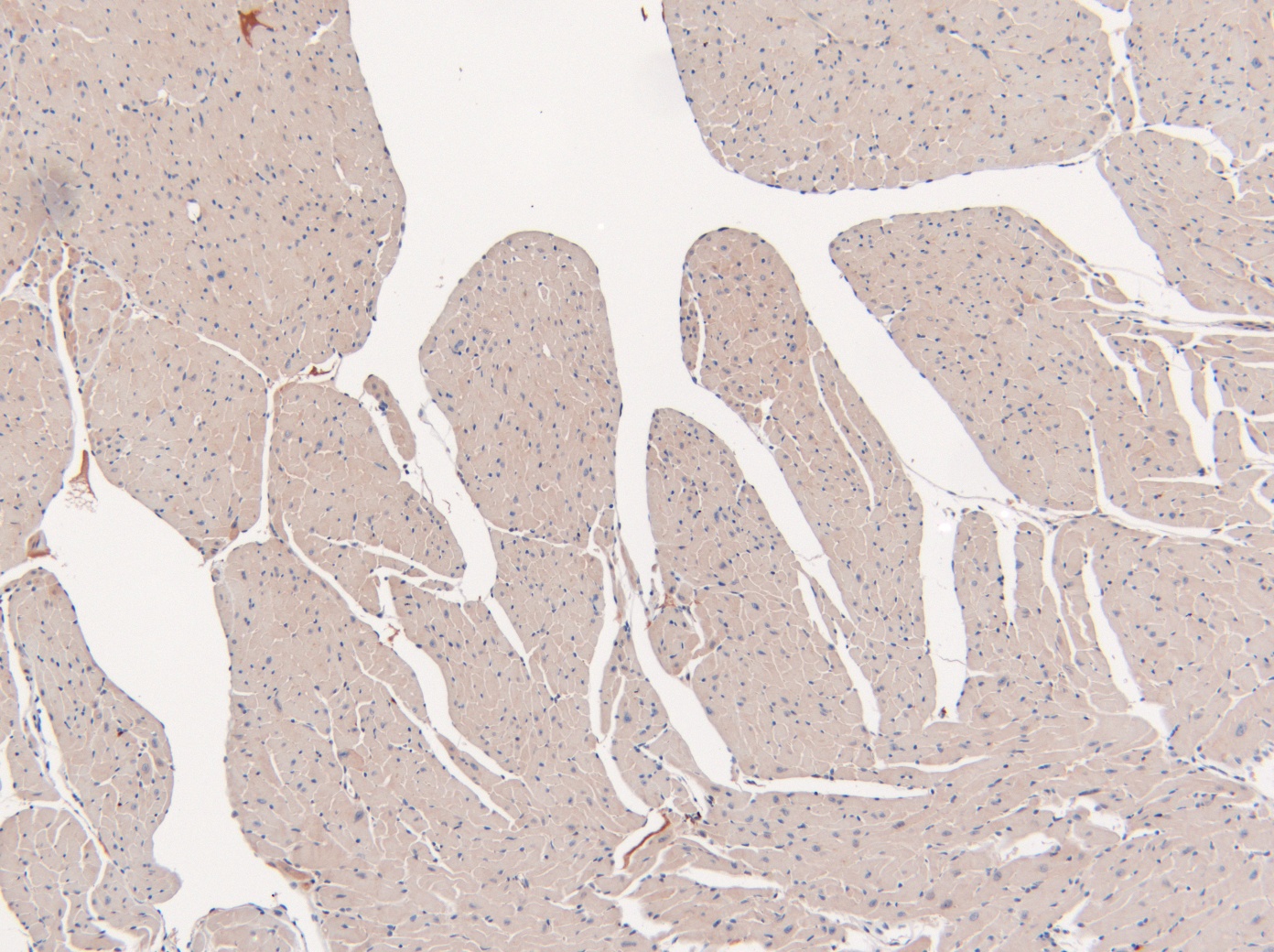

Supplement: Figure 4—source data 1. [file elife-55513-fig4-data1.zip › p16_images_for_eLife/p16_images_Ann_Chiao_for_eLife/Old SS-31 Treated/OSS_3/MS_6_p16_10x_j_RGB.jpg]

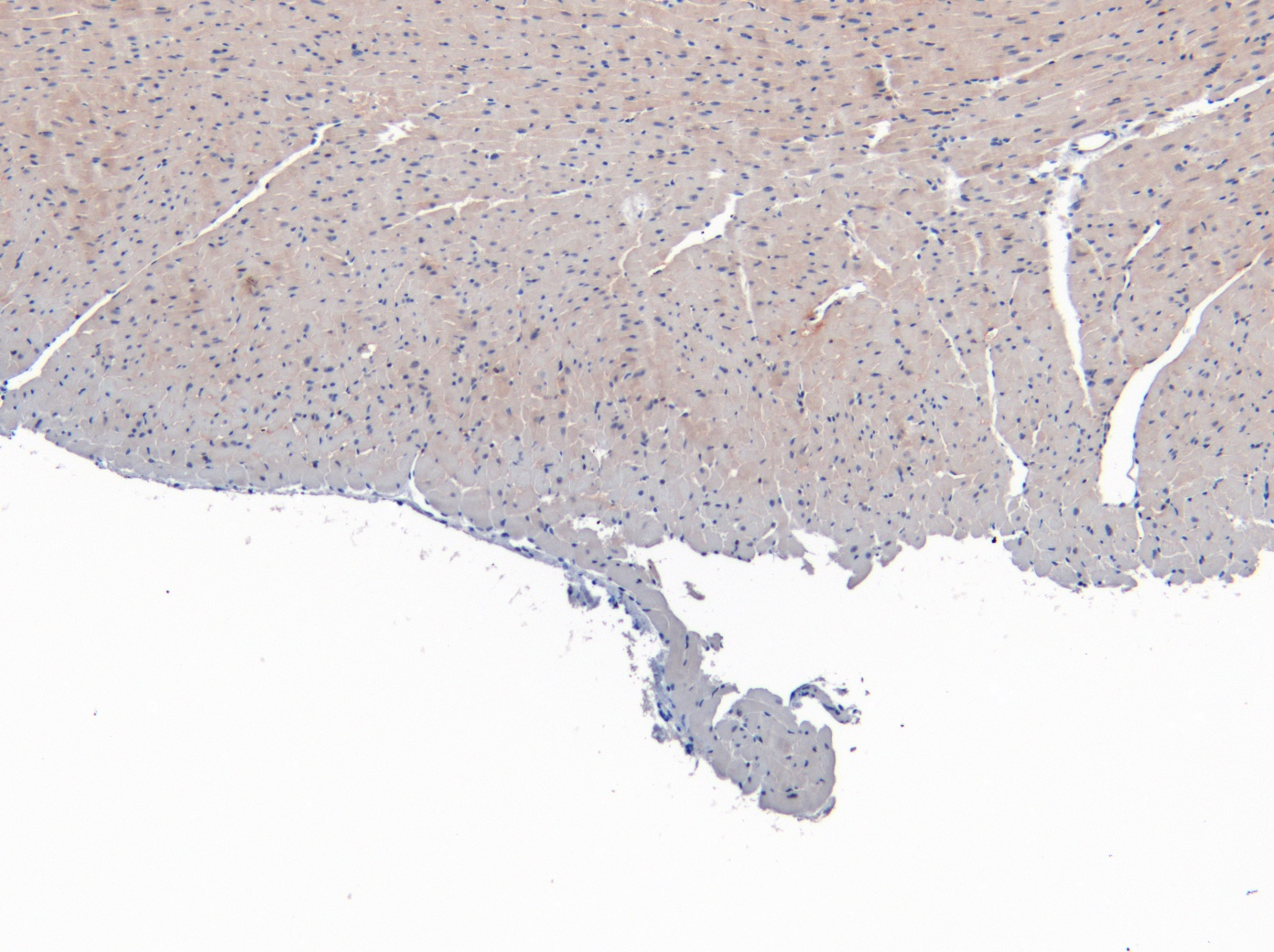

Supplement: Figure 4—source data 1. [file elife-55513-fig4-data1.zip › p16_images_for_eLife/p16_images_Ann_Chiao_for_eLife/Old SS-31 Treated/OSS_4/MS_8_p16_10x_a_RGB.jpg]

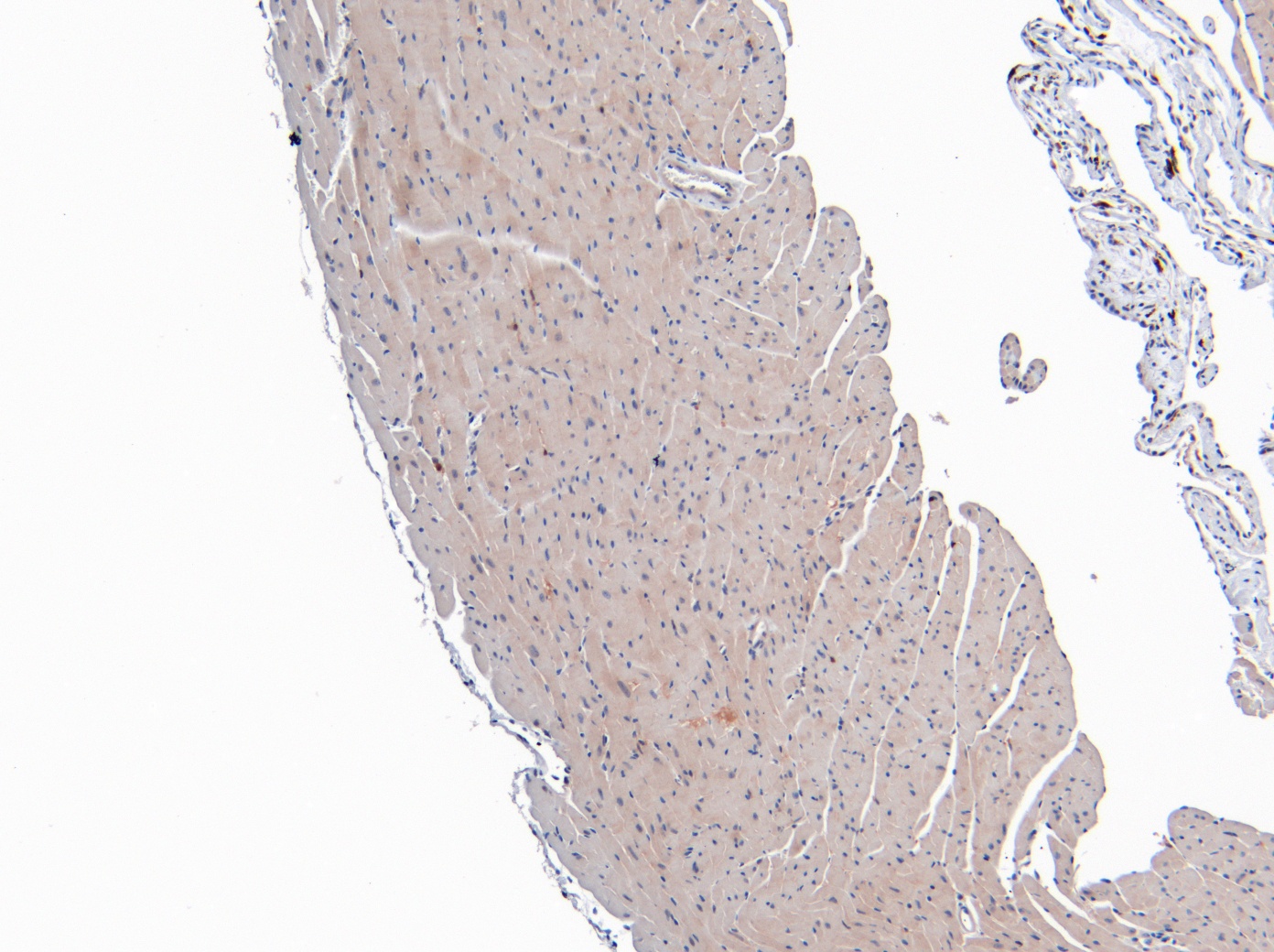

Supplement: Figure 4—source data 1. [file elife-55513-fig4-data1.zip › p16_images_for_eLife/p16_images_Ann_Chiao_for_eLife/Old SS-31 Treated/OSS_4/MS_8_p16_10x_b_RGB.jpg]

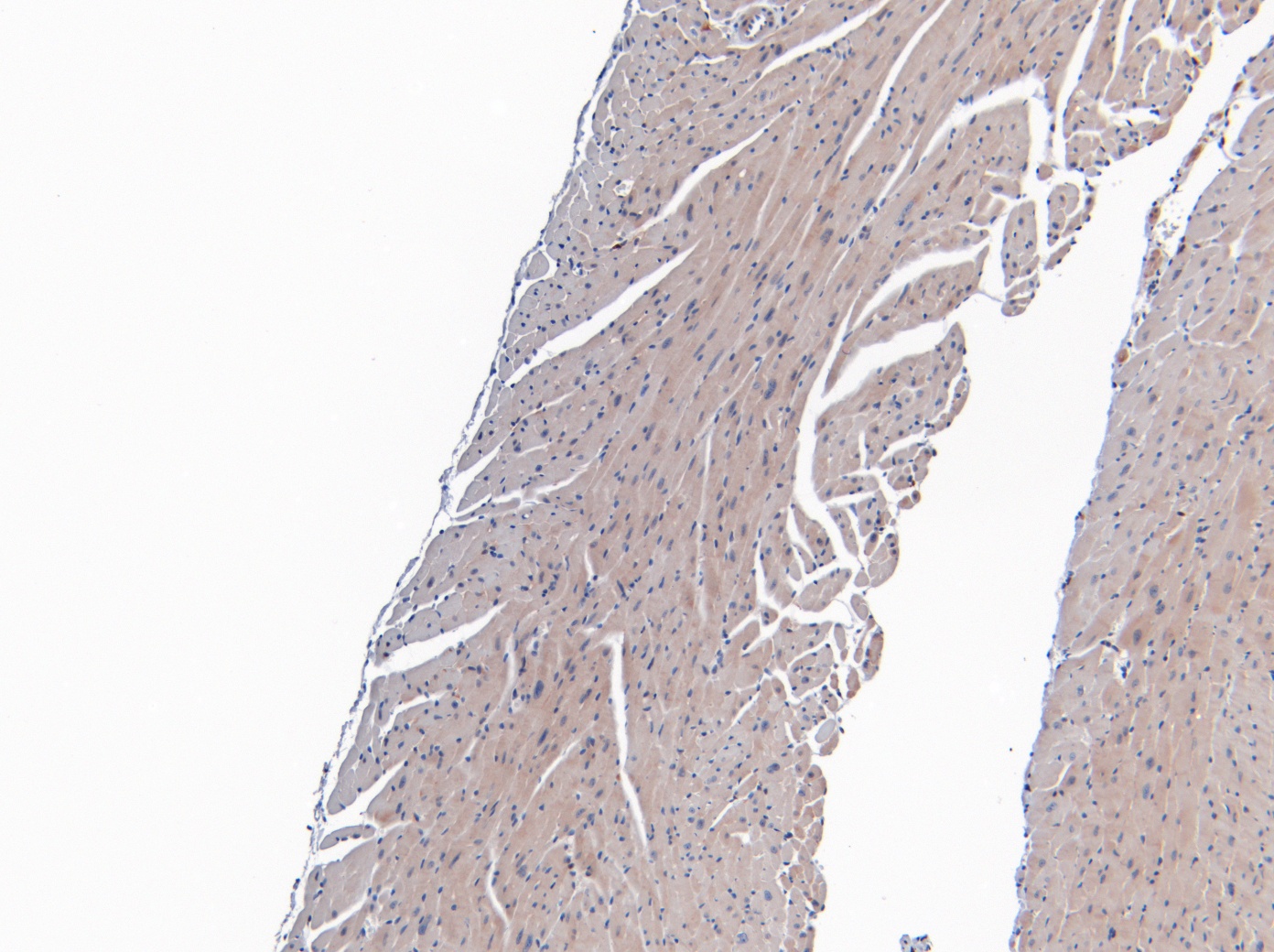

Supplement: Figure 4—source data 1. [file elife-55513-fig4-data1.zip › p16_images_for_eLife/p16_images_Ann_Chiao_for_eLife/Old SS-31 Treated/OSS_4/MS_8_p16_10x_c_RGB.jpg]

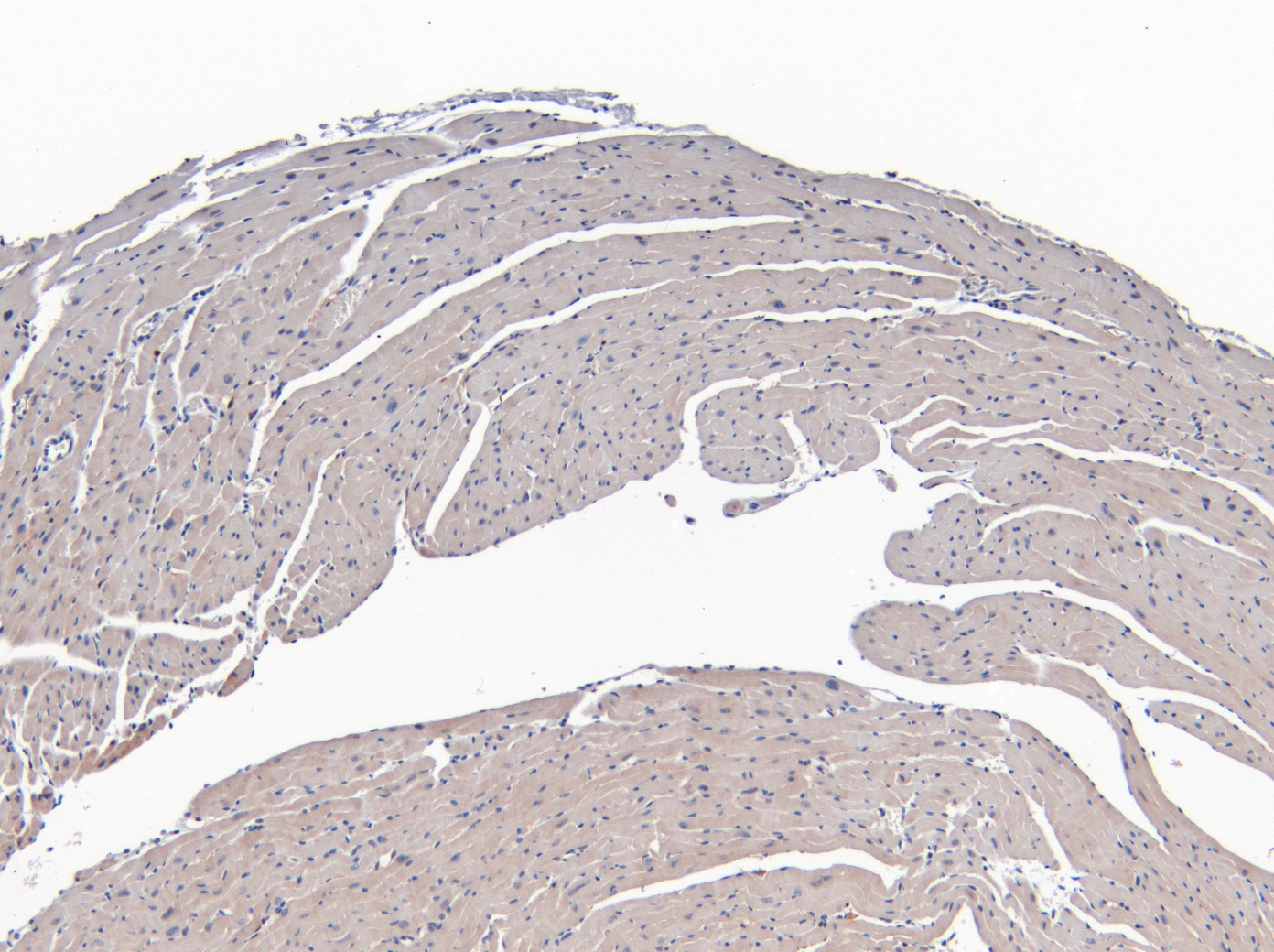

Supplement: Figure 4—source data 1. [file elife-55513-fig4-data1.zip › p16_images_for_eLife/p16_images_Ann_Chiao_for_eLife/Old SS-31 Treated/OSS_4/MS_8_p16_10x_d_RGB.jpg]

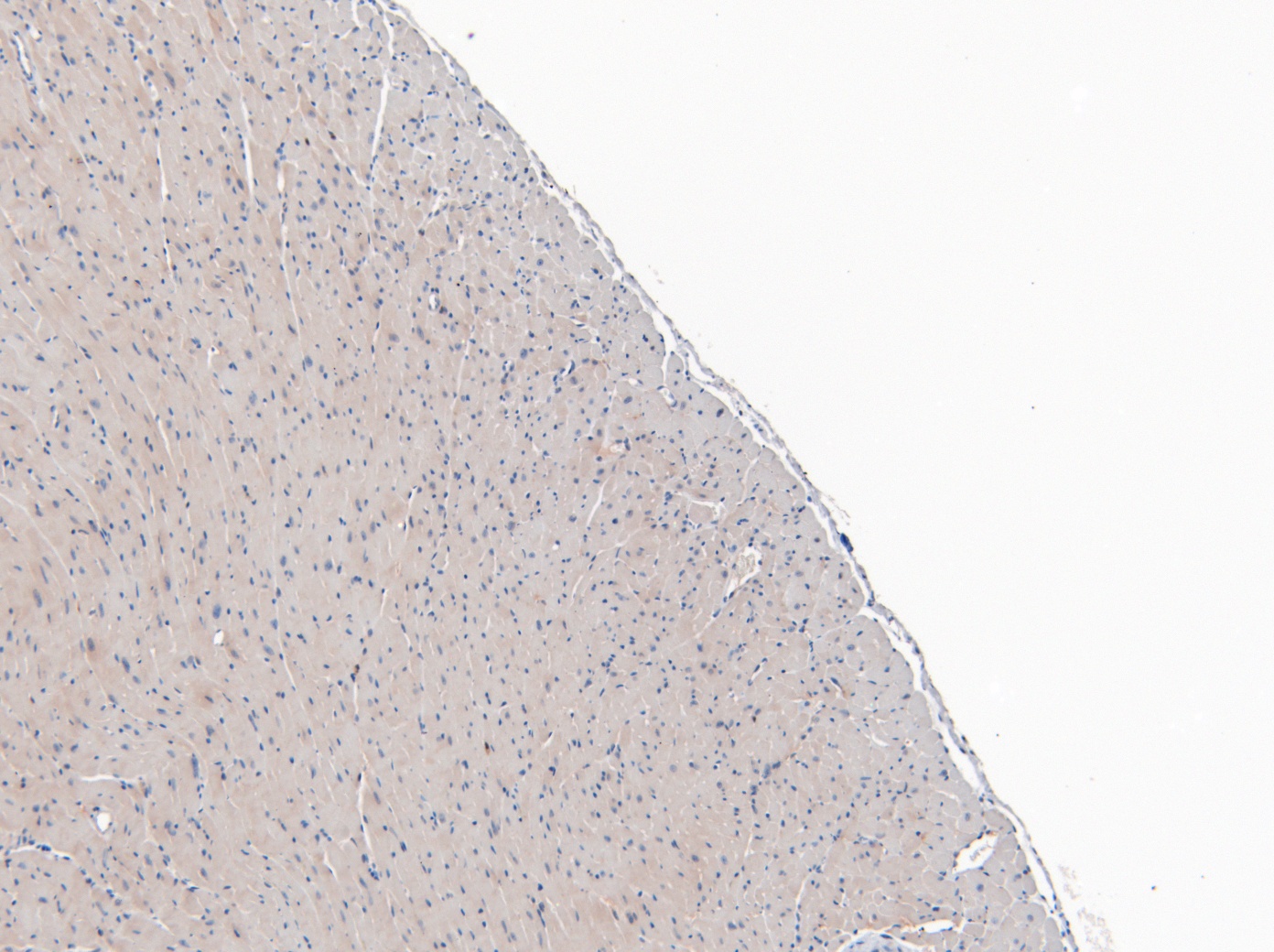

Supplement: Figure 4—source data 1. [file elife-55513-fig4-data1.zip › p16_images_for_eLife/p16_images_Ann_Chiao_for_eLife/Old SS-31 Treated/OSS_4/MS_8_p16_10x_e_RGB.jpg]

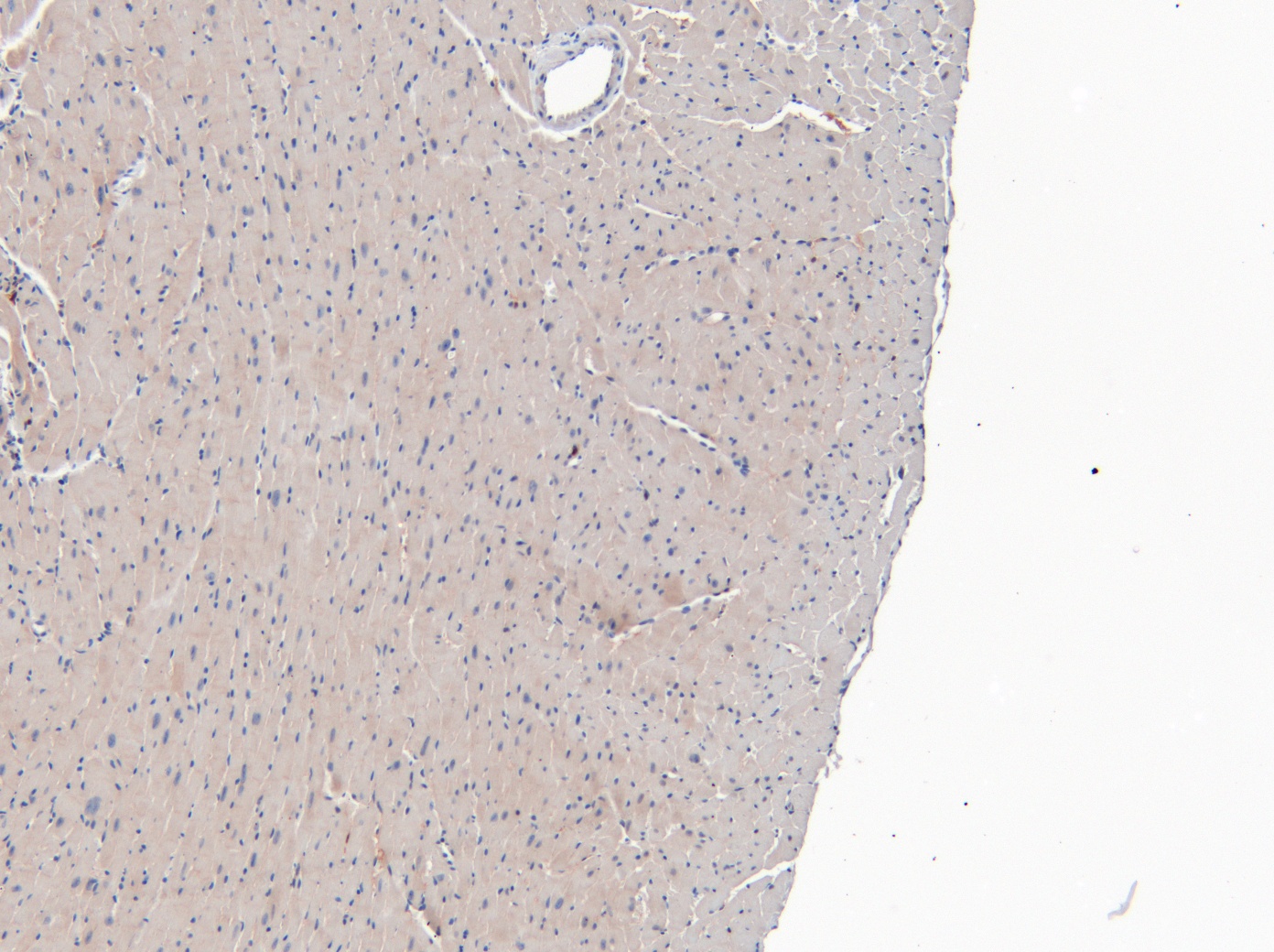

Supplement: Figure 4—source data 1. [file elife-55513-fig4-data1.zip › p16_images_for_eLife/p16_images_Ann_Chiao_for_eLife/Old SS-31 Treated/OSS_4/MS_8_p16_10x_f_RGB.jpg]

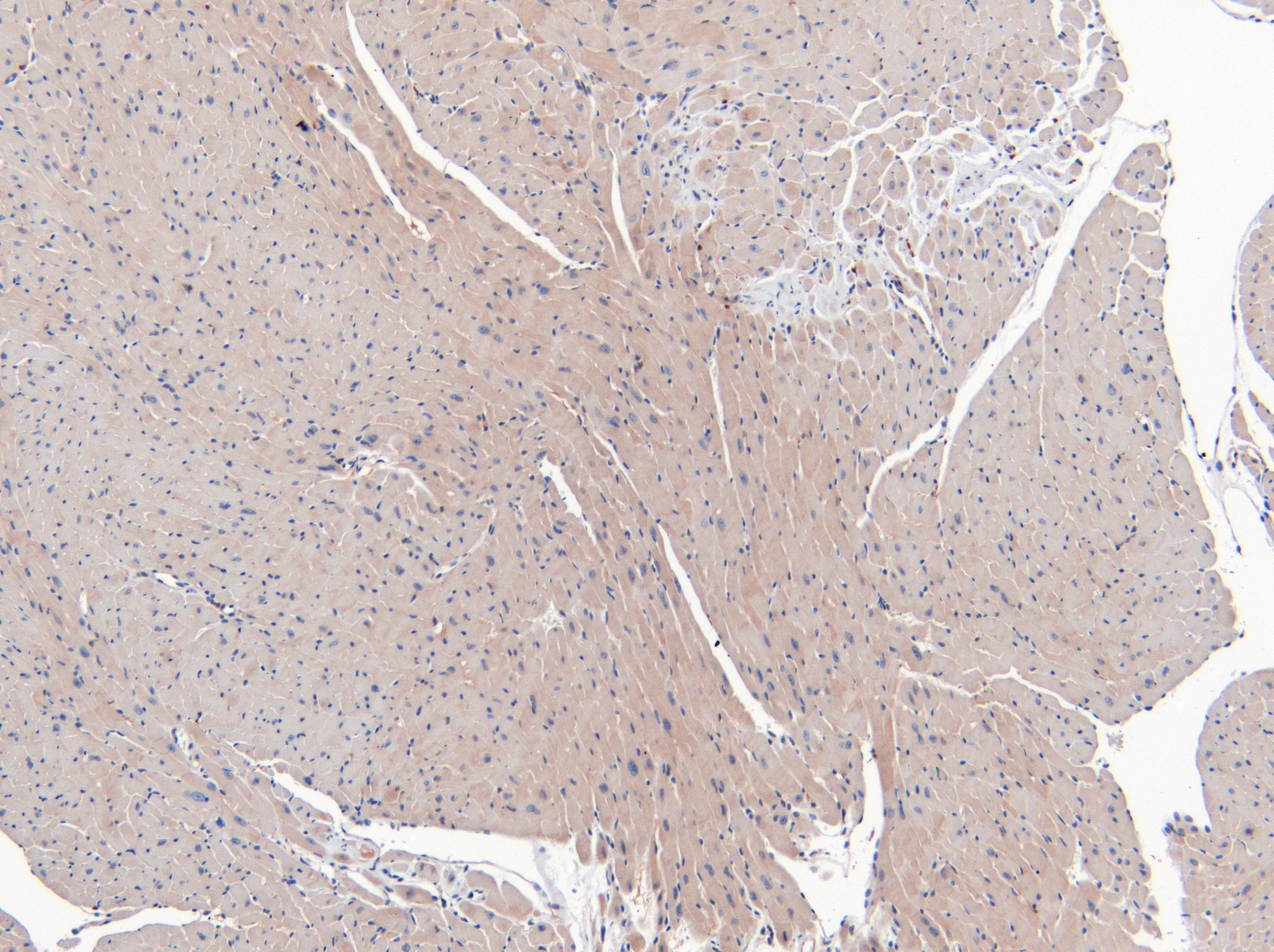

Supplement: Figure 4—source data 1. [file elife-55513-fig4-data1.zip › p16_images_for_eLife/p16_images_Ann_Chiao_for_eLife/Old SS-31 Treated/OSS_4/MS_8_p16_10x_g_RGB.jpg]

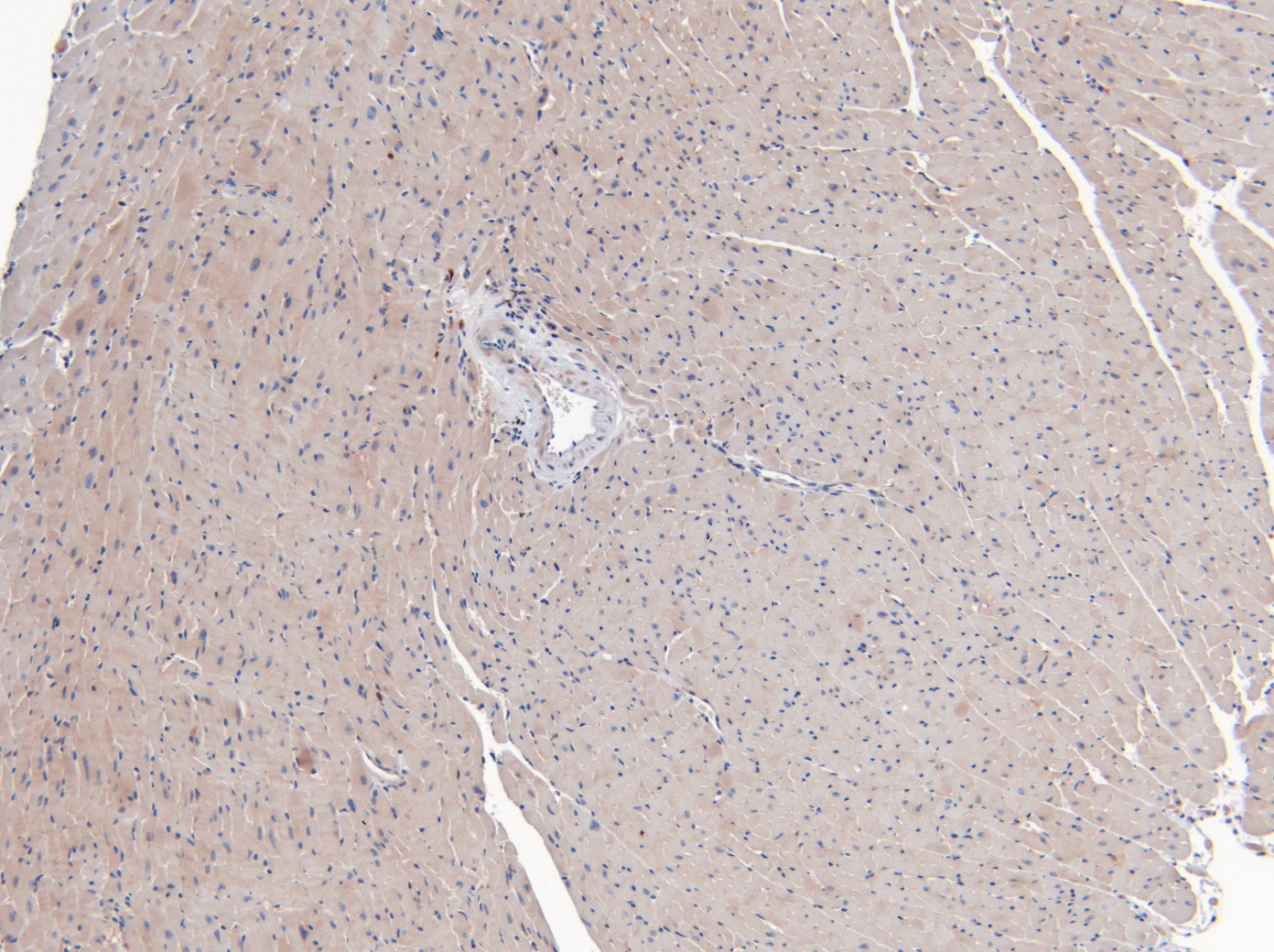

Supplement: Figure 4—source data 1. [file elife-55513-fig4-data1.zip › p16_images_for_eLife/p16_images_Ann_Chiao_for_eLife/Old SS-31 Treated/OSS_4/MS_8_p16_10x_h_RGB.jpg]

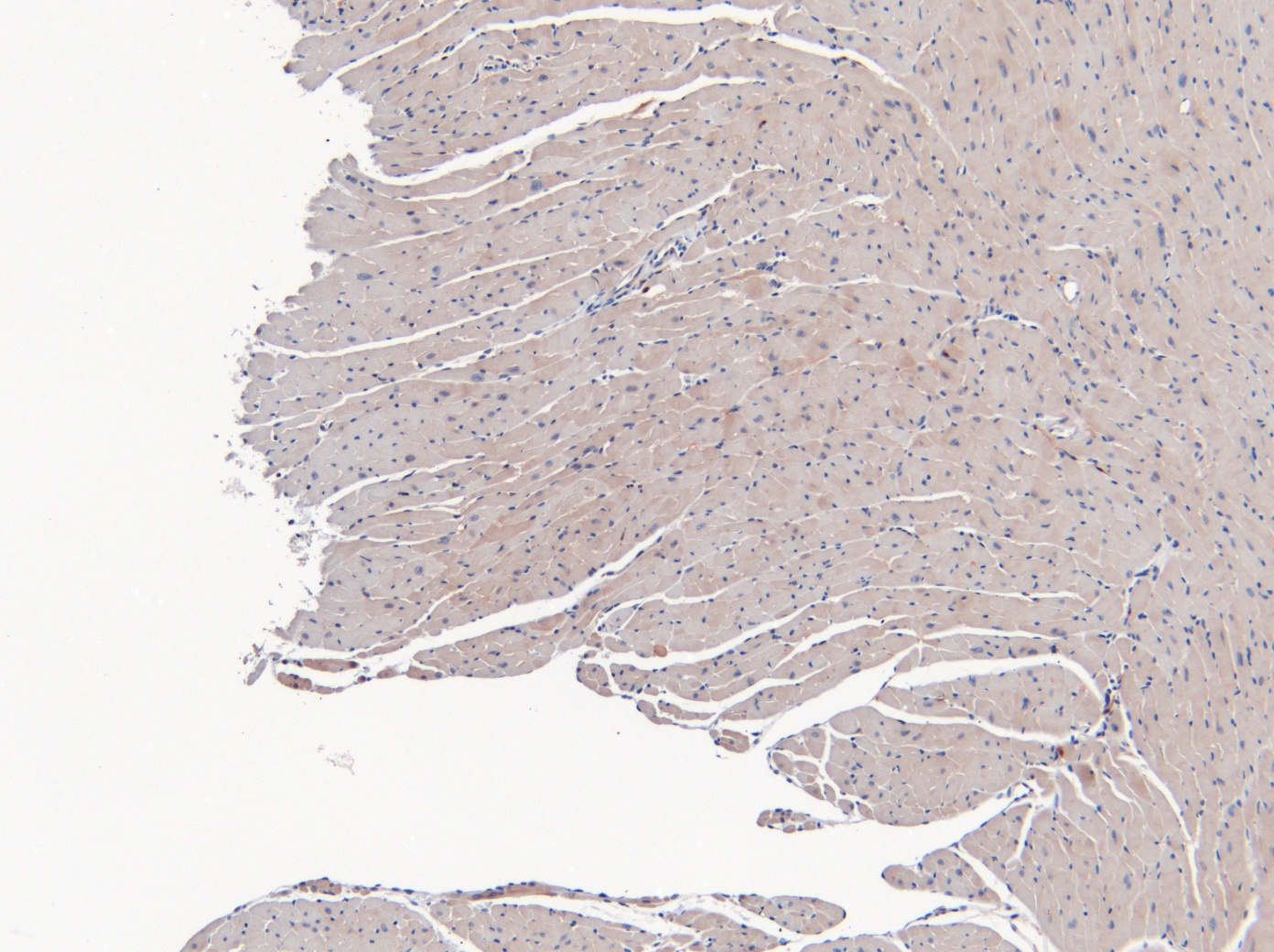

Supplement: Figure 4—source data 1. [file elife-55513-fig4-data1.zip › p16_images_for_eLife/p16_images_Ann_Chiao_for_eLife/Old SS-31 Treated/OSS_4/MS_8_p16_10x_i_RGB.jpg]

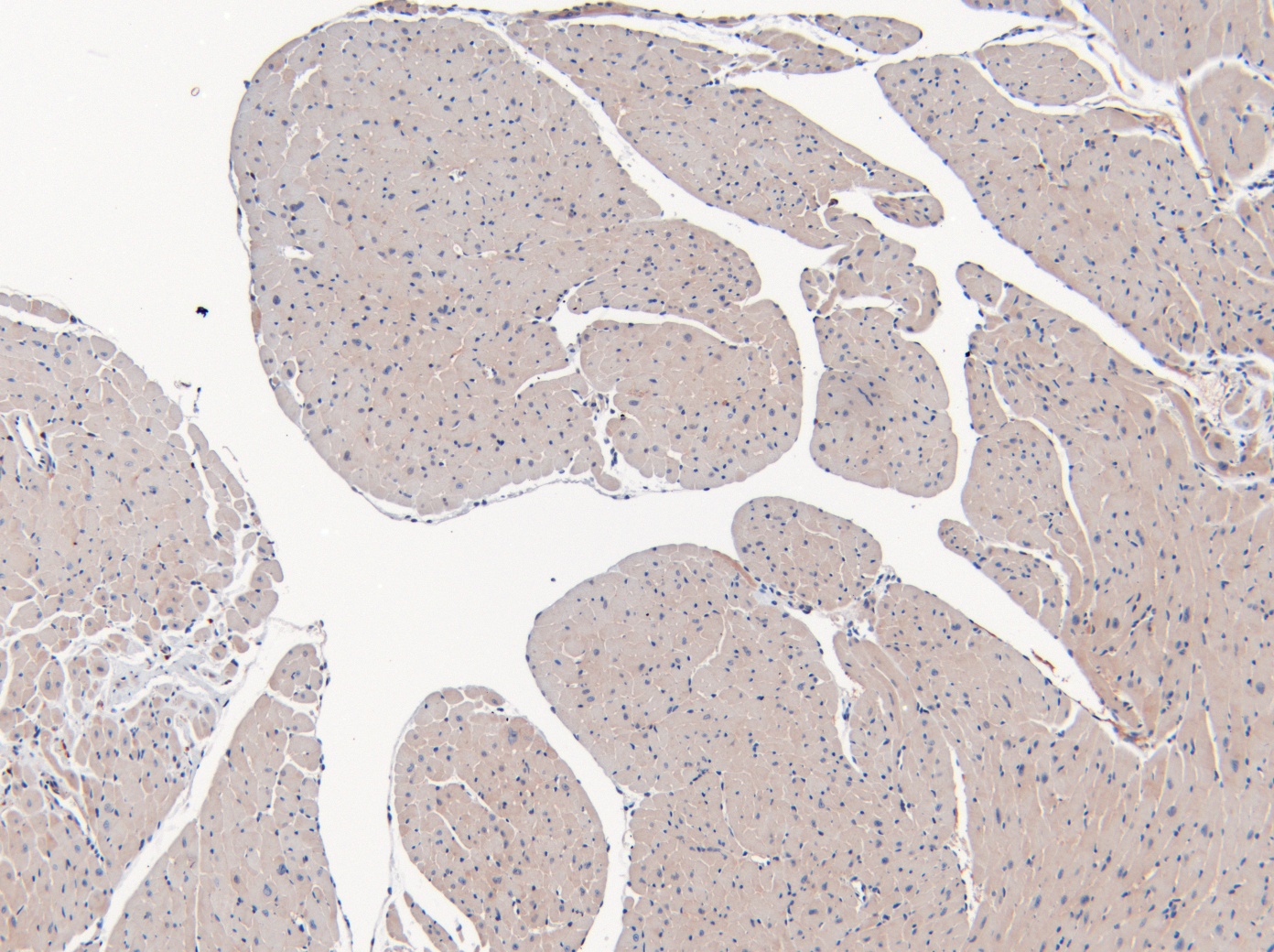

Supplement: Figure 4—source data 1. [file elife-55513-fig4-data1.zip › p16_images_for_eLife/p16_images_Ann_Chiao_for_eLife/Old SS-31 Treated/OSS_4/MS_8_p16_10x_j_RGB.jpg]

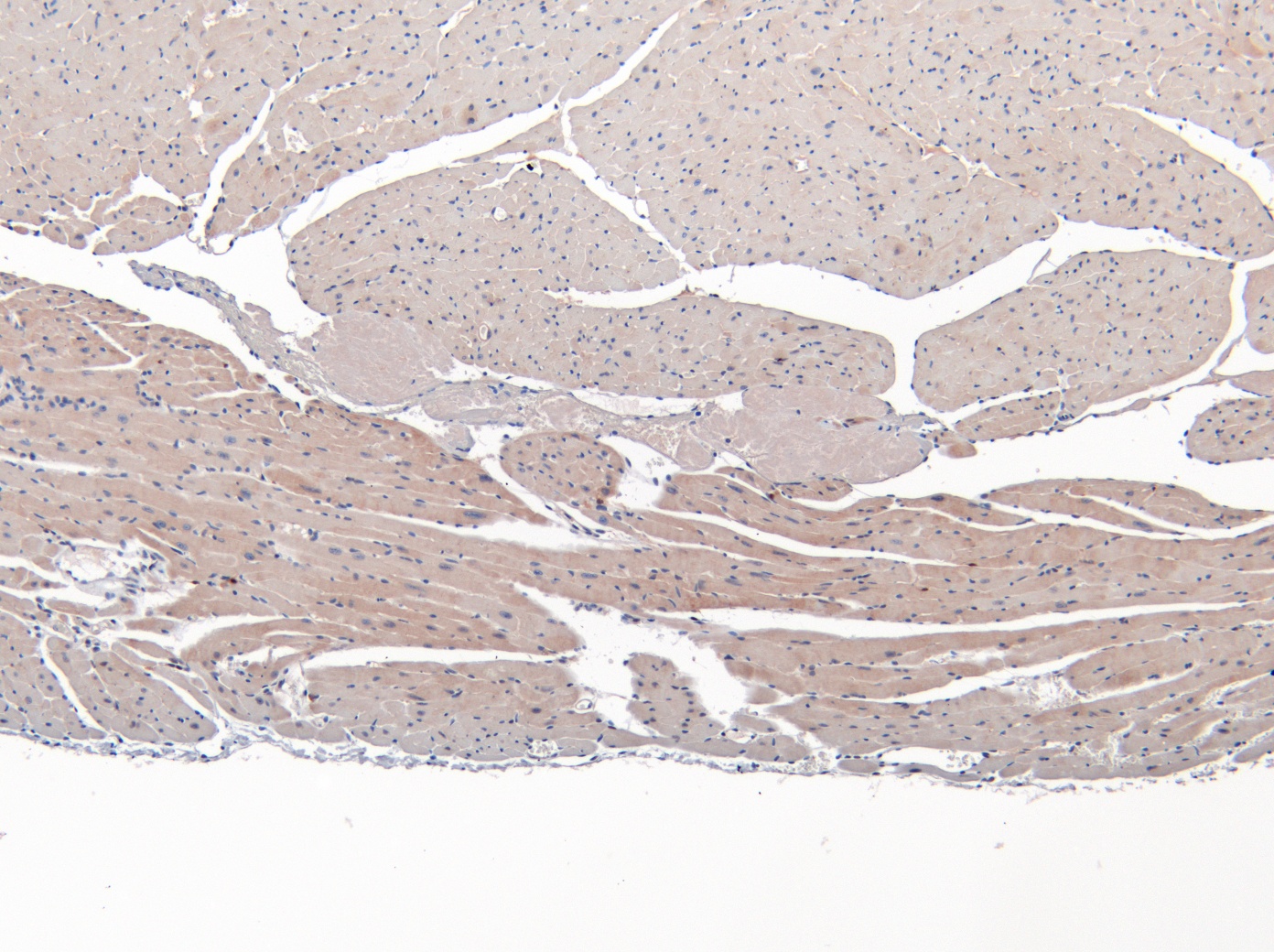

Supplement: Figure 4—source data 1. [file elife-55513-fig4-data1.zip › p16_images_for_eLife/p16_images_Ann_Chiao_for_eLife/Old SS-31 Treated/OSS_5/MS_9_p16_10x_a_RGB.jpg]

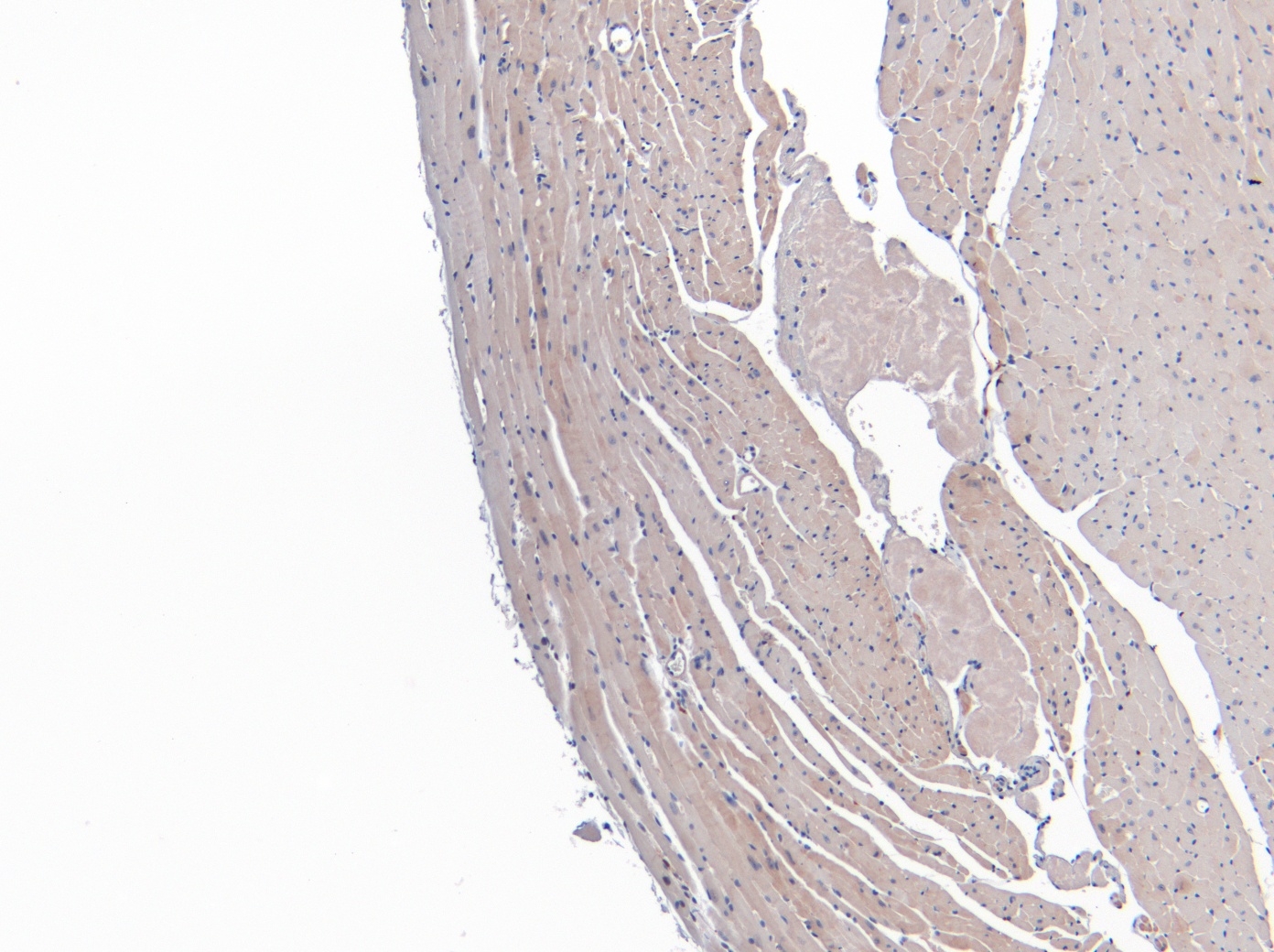

Supplement: Figure 4—source data 1. [file elife-55513-fig4-data1.zip › p16_images_for_eLife/p16_images_Ann_Chiao_for_eLife/Old SS-31 Treated/OSS_5/MS_9_p16_10x_b_RGB.jpg]

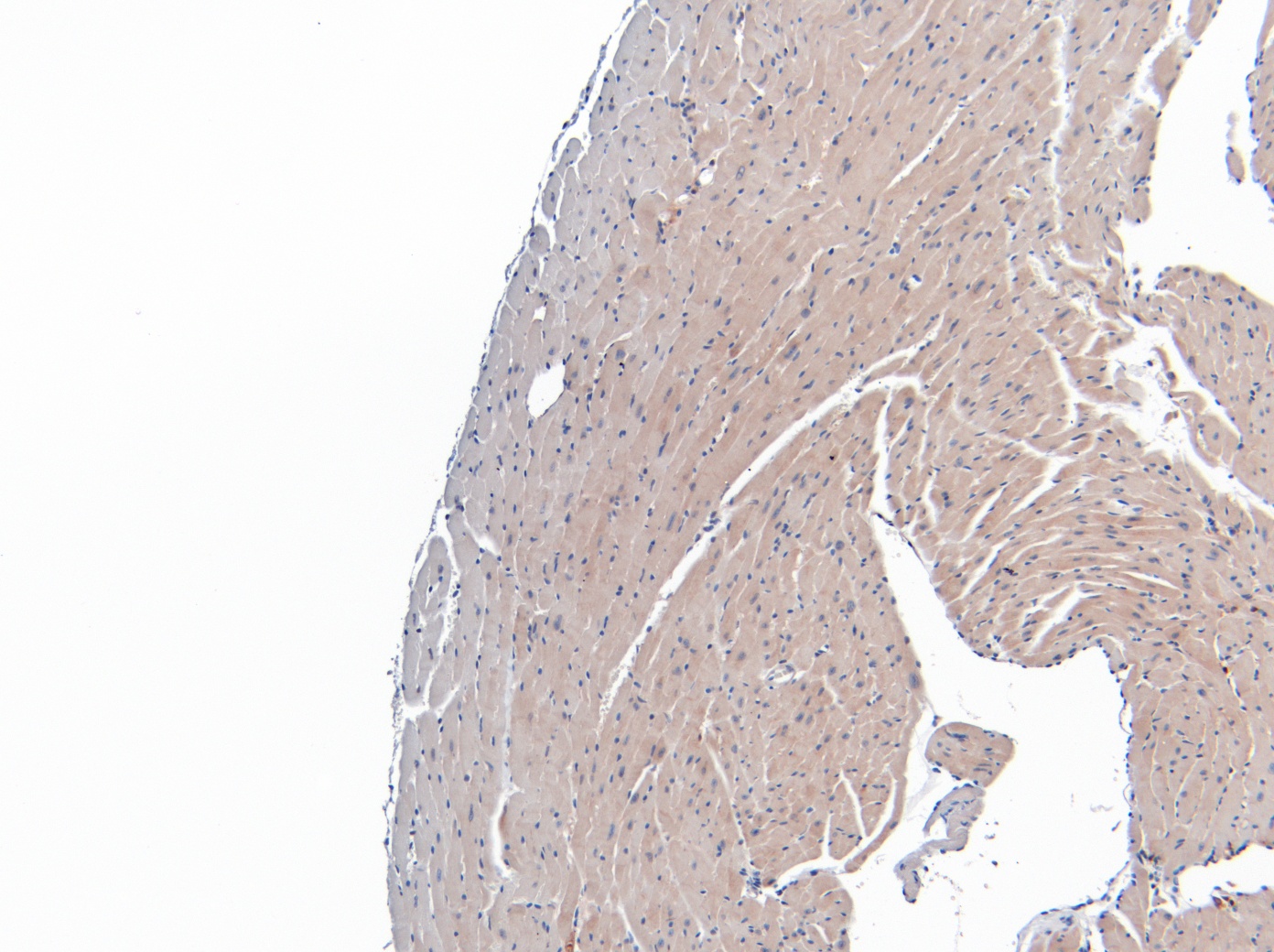

Supplement: Figure 4—source data 1. [file elife-55513-fig4-data1.zip › p16_images_for_eLife/p16_images_Ann_Chiao_for_eLife/Old SS-31 Treated/OSS_5/MS_9_p16_10x_c_RGB.jpg]

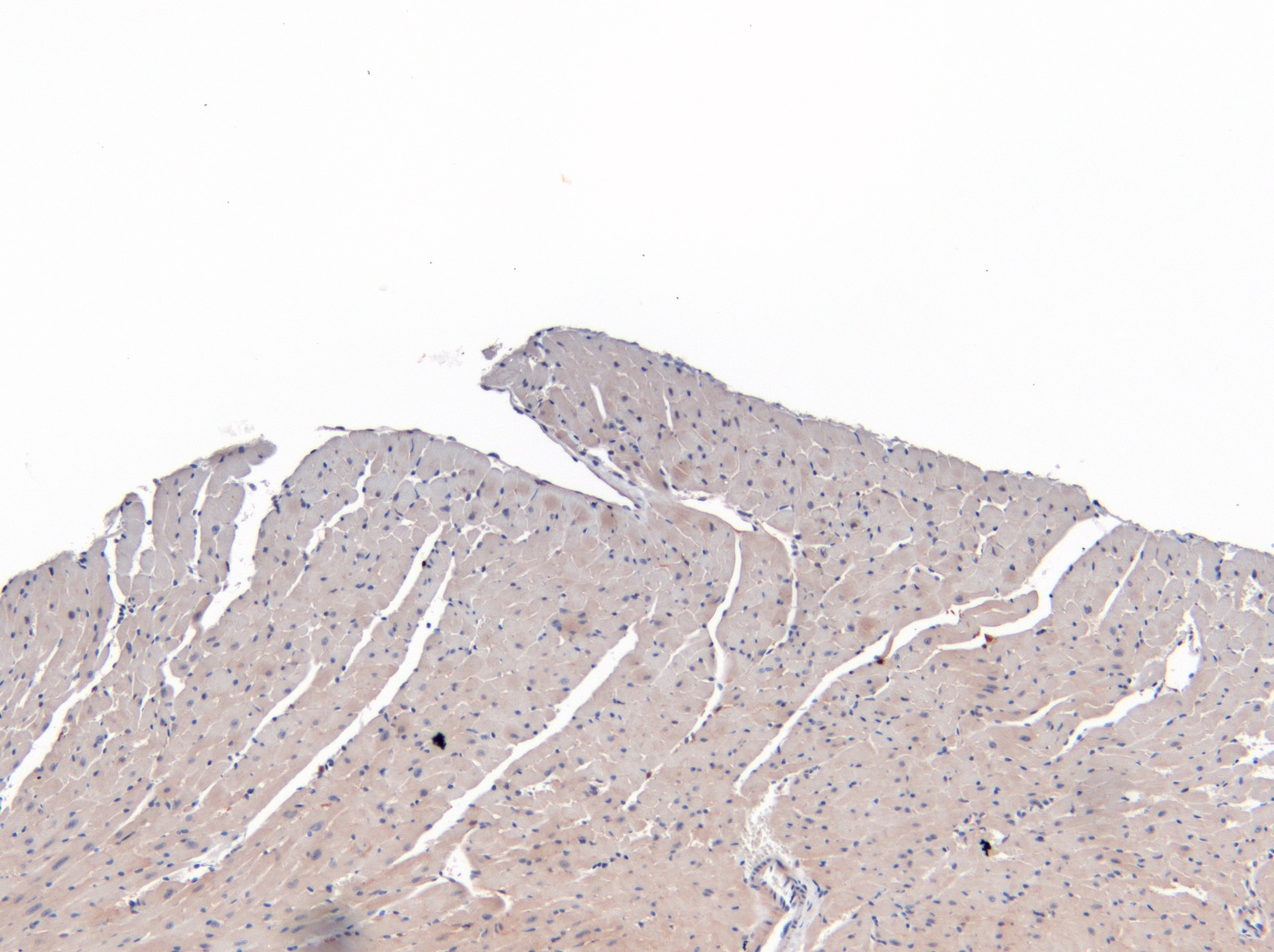

Supplement: Figure 4—source data 1. [file elife-55513-fig4-data1.zip › p16_images_for_eLife/p16_images_Ann_Chiao_for_eLife/Old SS-31 Treated/OSS_5/MS_9_p16_10x_d_RGB.jpg]

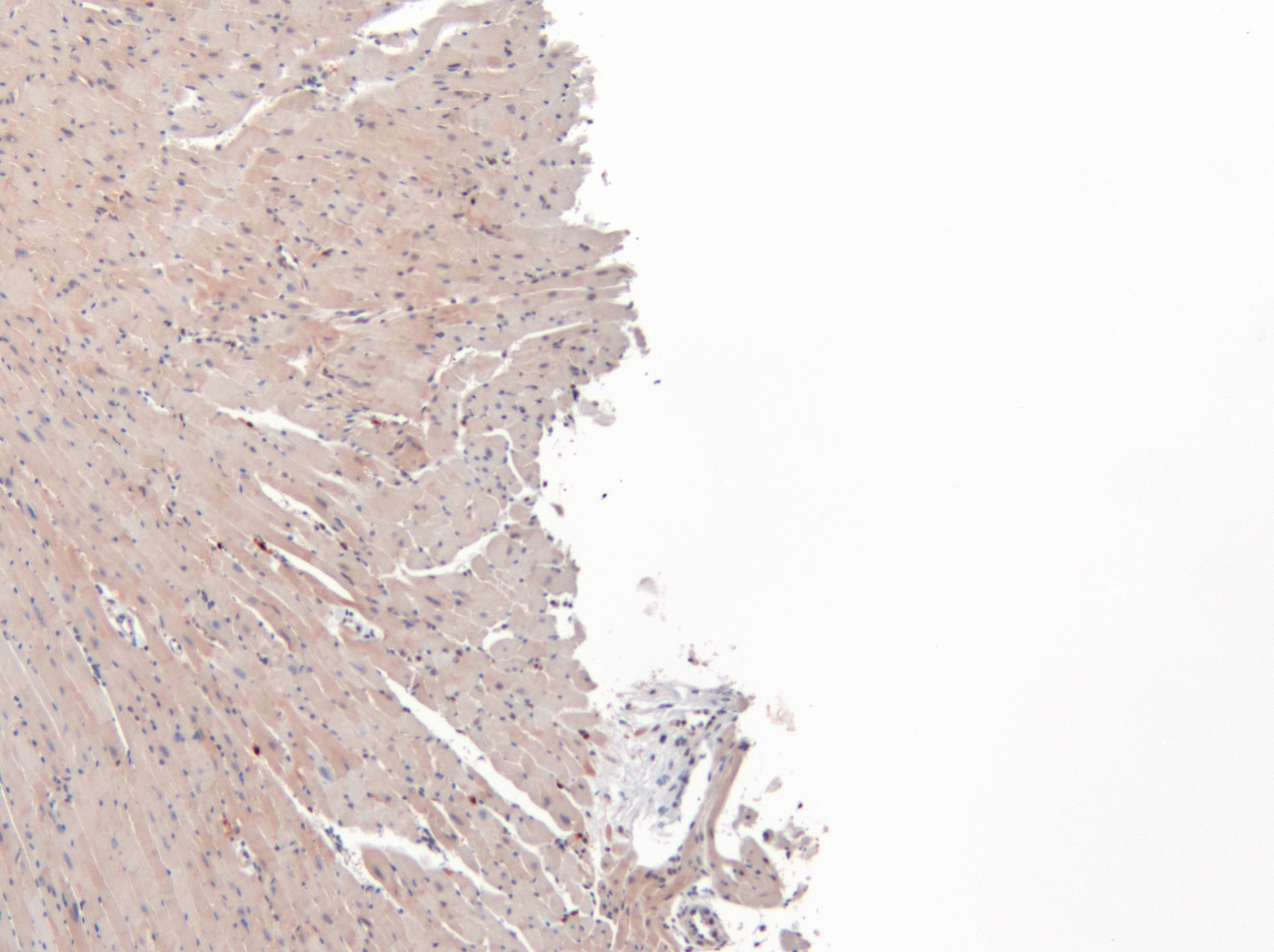

Supplement: Figure 4—source data 1. [file elife-55513-fig4-data1.zip › p16_images_for_eLife/p16_images_Ann_Chiao_for_eLife/Old SS-31 Treated/OSS_5/MS_9_p16_10x_e_RGB.jpg]

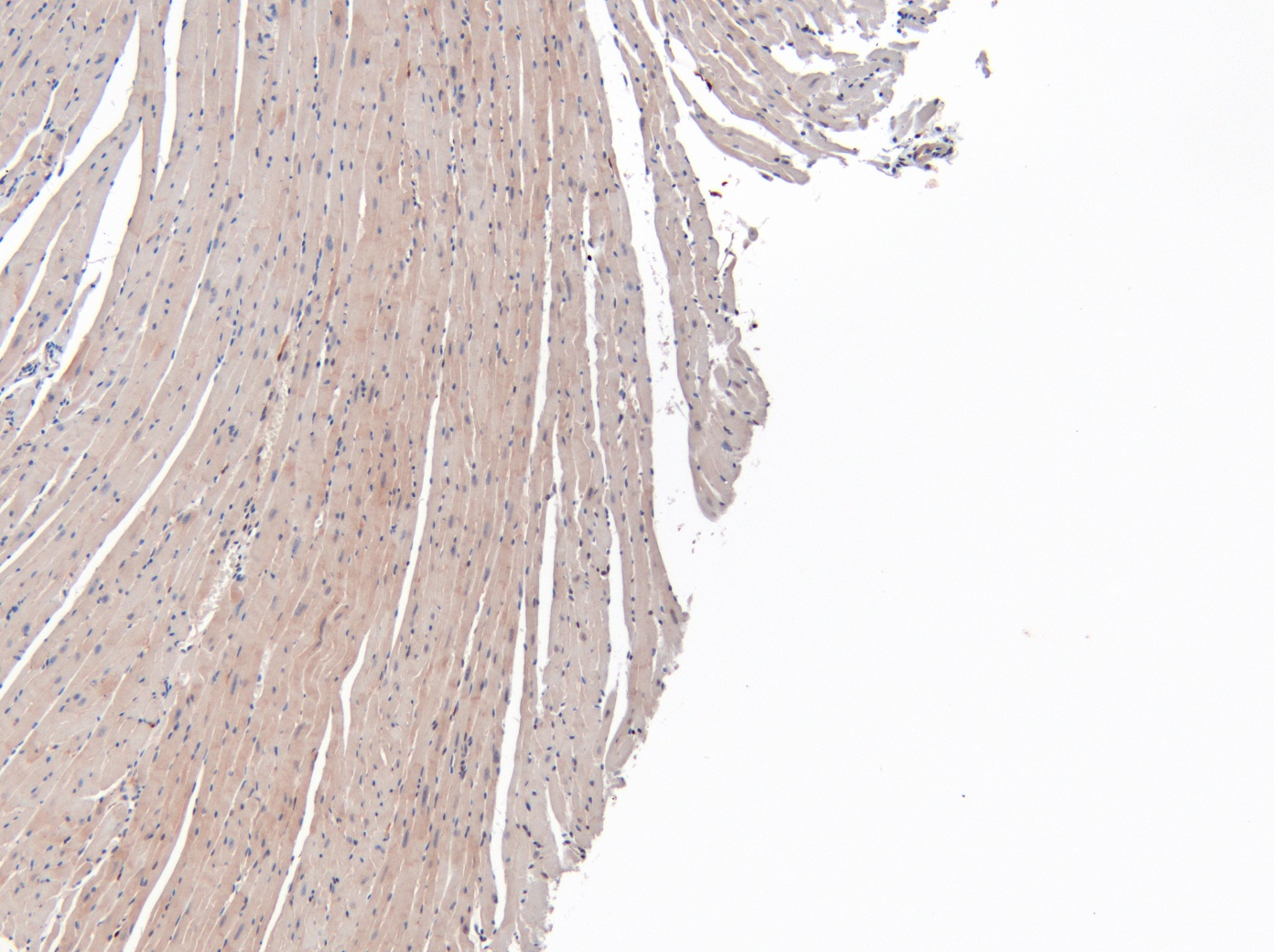

Supplement: Figure 4—source data 1. [file elife-55513-fig4-data1.zip › p16_images_for_eLife/p16_images_Ann_Chiao_for_eLife/Old SS-31 Treated/OSS_5/MS_9_p16_10x_f_RGB.jpg]

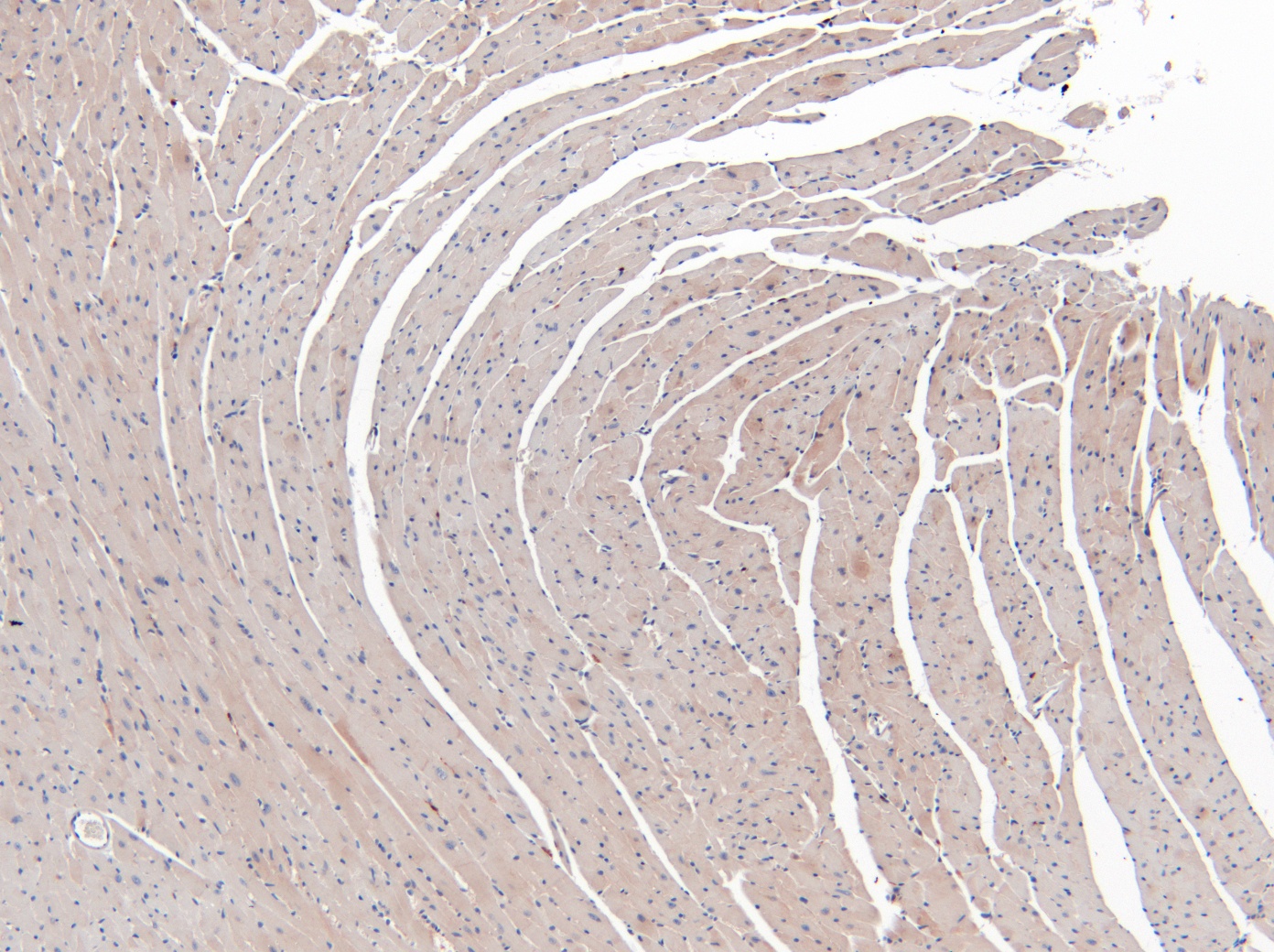

Supplement: Figure 4—source data 1. [file elife-55513-fig4-data1.zip › p16_images_for_eLife/p16_images_Ann_Chiao_for_eLife/Old SS-31 Treated/OSS_5/MS_9_p16_10x_g_RGB.jpg]

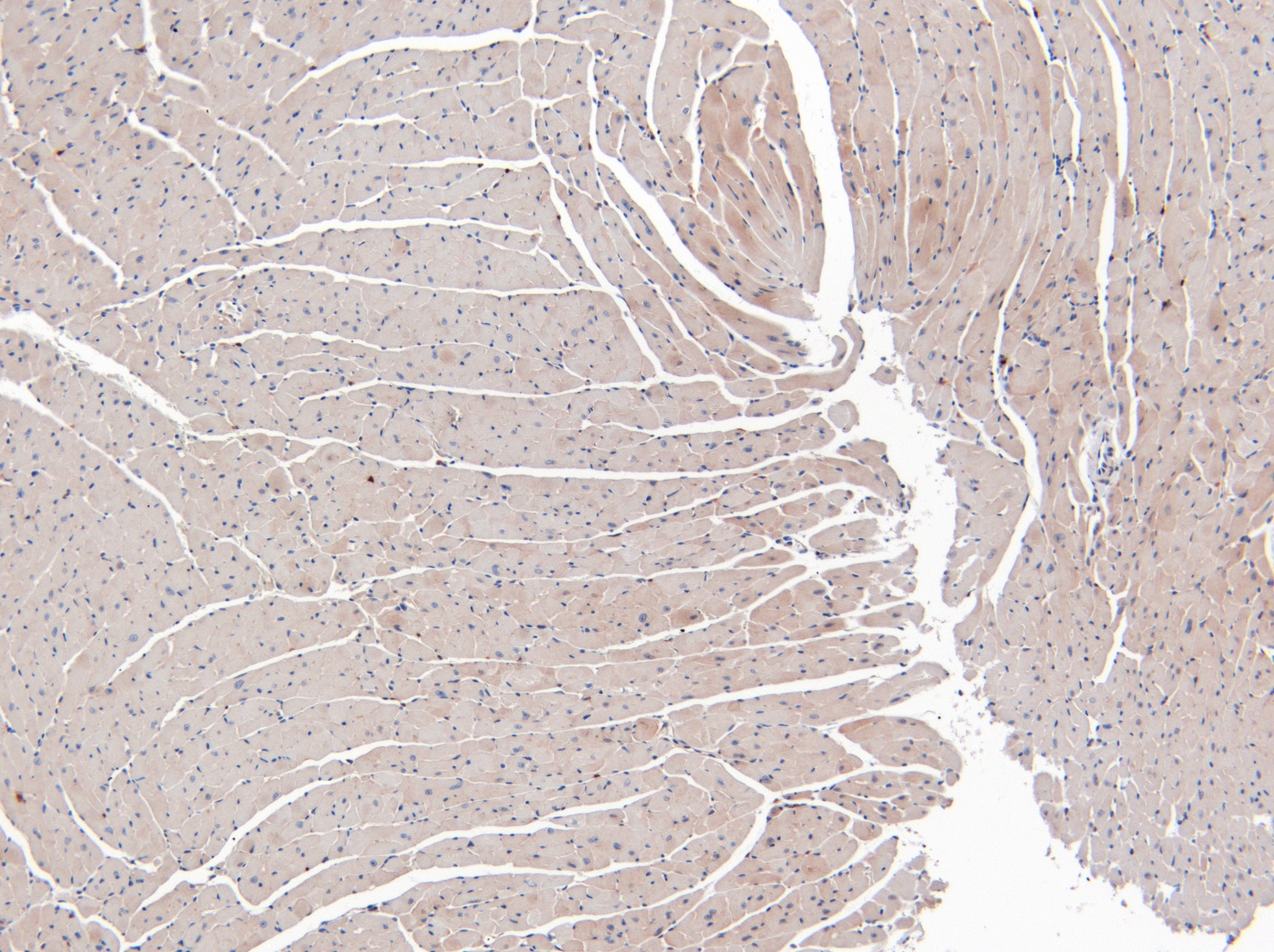

Supplement: Figure 4—source data 1. [file elife-55513-fig4-data1.zip › p16_images_for_eLife/p16_images_Ann_Chiao_for_eLife/Old SS-31 Treated/OSS_5/MS_9_p16_10x_h_RGB.jpg]

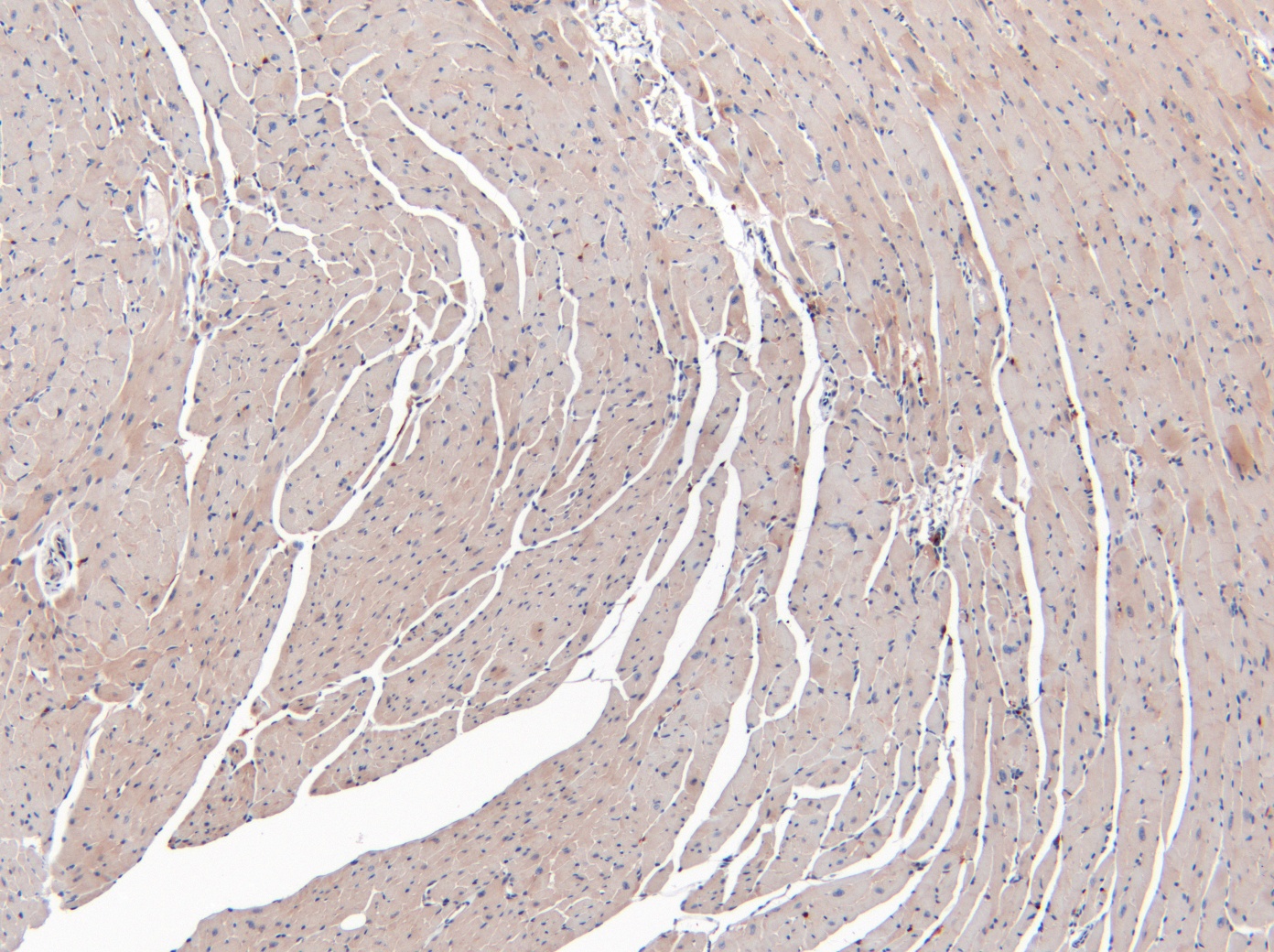

Supplement: Figure 4—source data 1. [file elife-55513-fig4-data1.zip › p16_images_for_eLife/p16_images_Ann_Chiao_for_eLife/Old SS-31 Treated/OSS_5/MS_9_p16_10x_i_RGB.jpg]

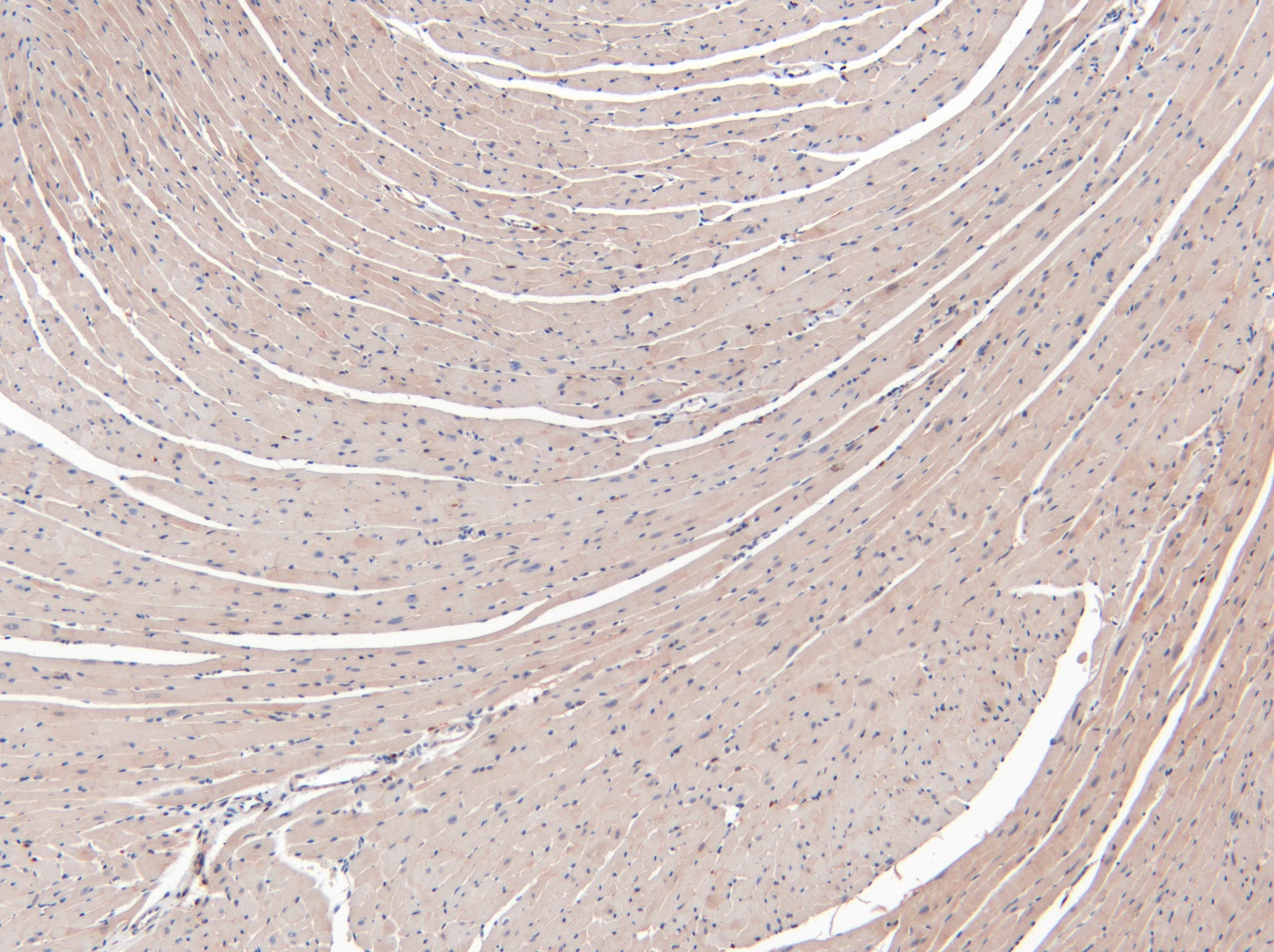

Supplement: Figure 4—source data 1. [file elife-55513-fig4-data1.zip › p16_images_for_eLife/p16_images_Ann_Chiao_for_eLife/Old SS-31 Treated/OSS_5/MS_9_p16_10x_j_RGB.jpg]

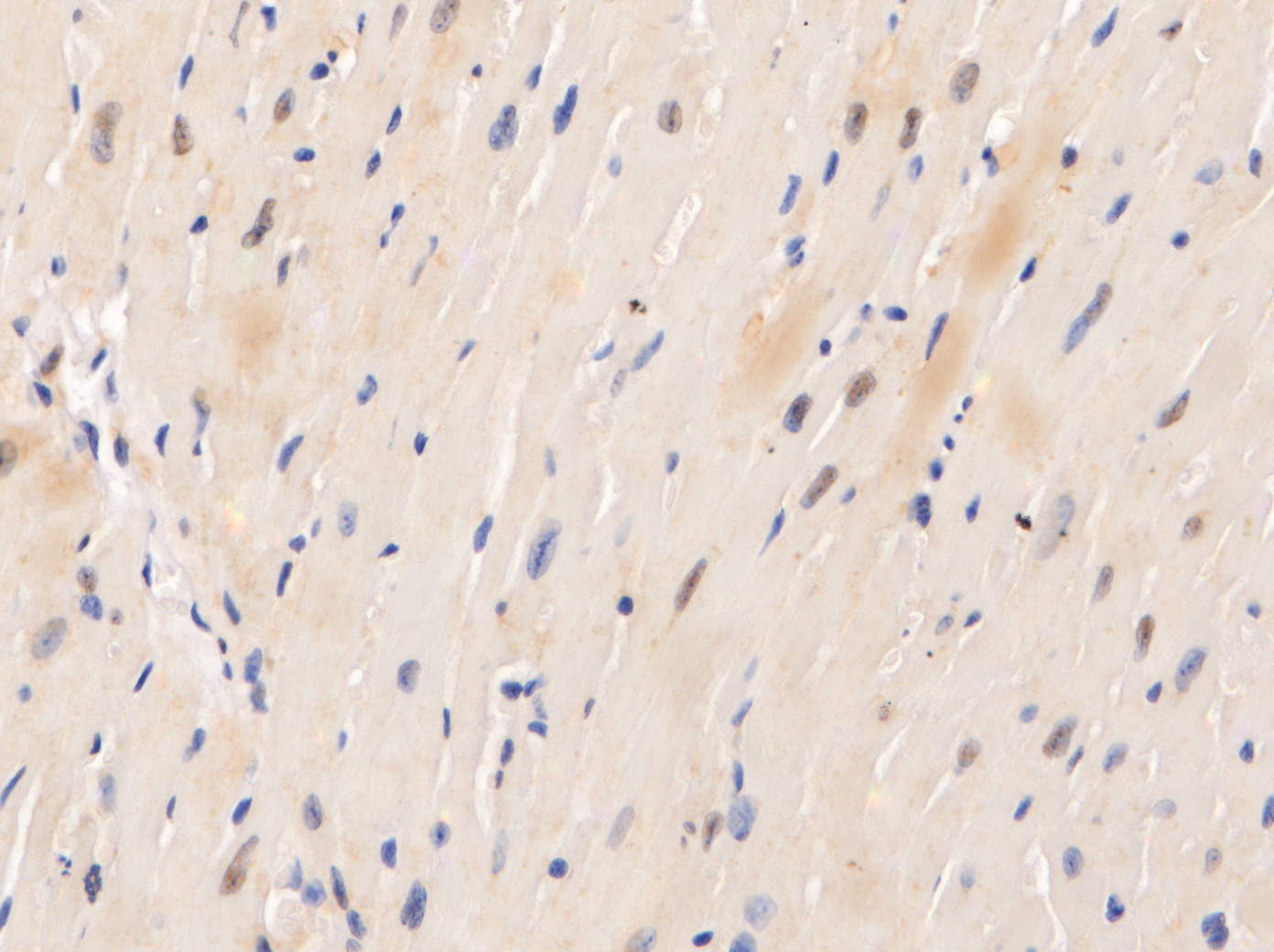

Supplement: Figure 4—source data 2. [file elife-55513-fig4-data2.zip › p19_images_for_eLife/p19_images_Ann_Chiao_for_eLife/Old controls/OCL_1/Copy of MS2_1_40x_RGB.jpg]

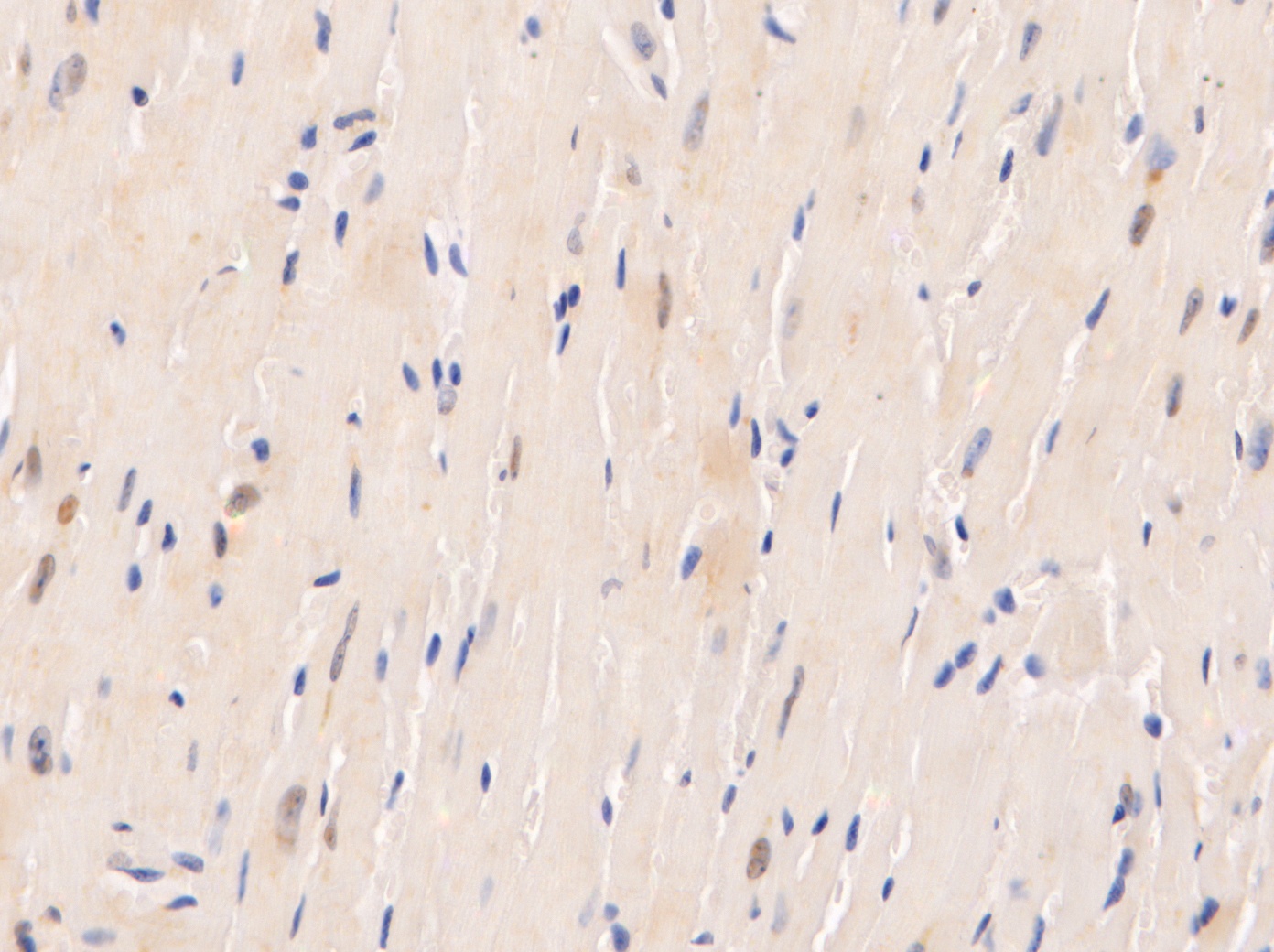

Supplement: Figure 4—source data 2. [file elife-55513-fig4-data2.zip › p19_images_for_eLife/p19_images_Ann_Chiao_for_eLife/Old controls/OCL_1/Copy of MS2_2_40x_RGB.jpg]

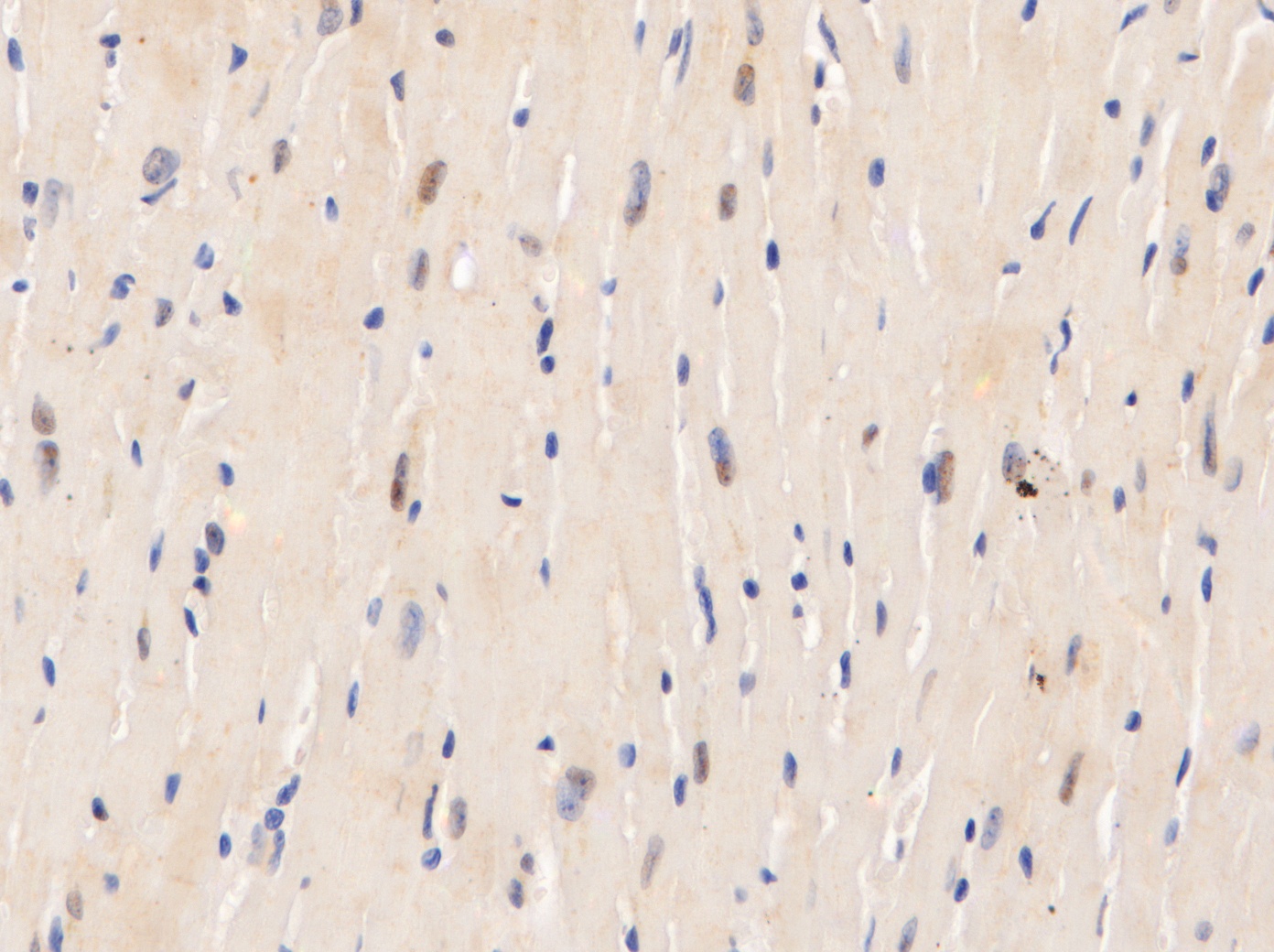

Supplement: Figure 4—source data 2. [file elife-55513-fig4-data2.zip › p19_images_for_eLife/p19_images_Ann_Chiao_for_eLife/Old controls/OCL_1/Copy of MS2_3_40x_RGB.jpg]

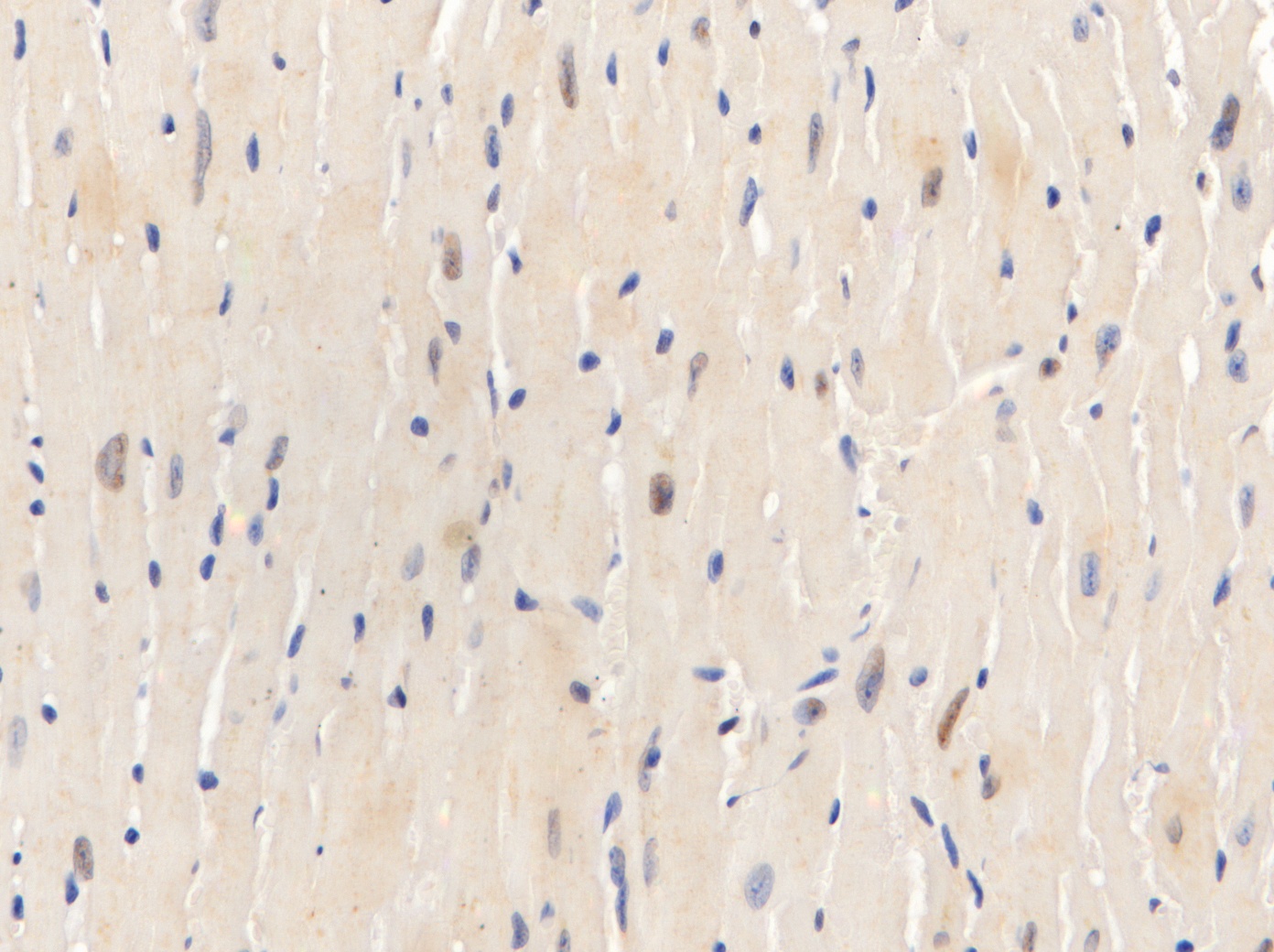

Supplement: Figure 4—source data 2. [file elife-55513-fig4-data2.zip › p19_images_for_eLife/p19_images_Ann_Chiao_for_eLife/Old controls/OCL_1/Copy of MS2_4_40x_RGB.jpg]

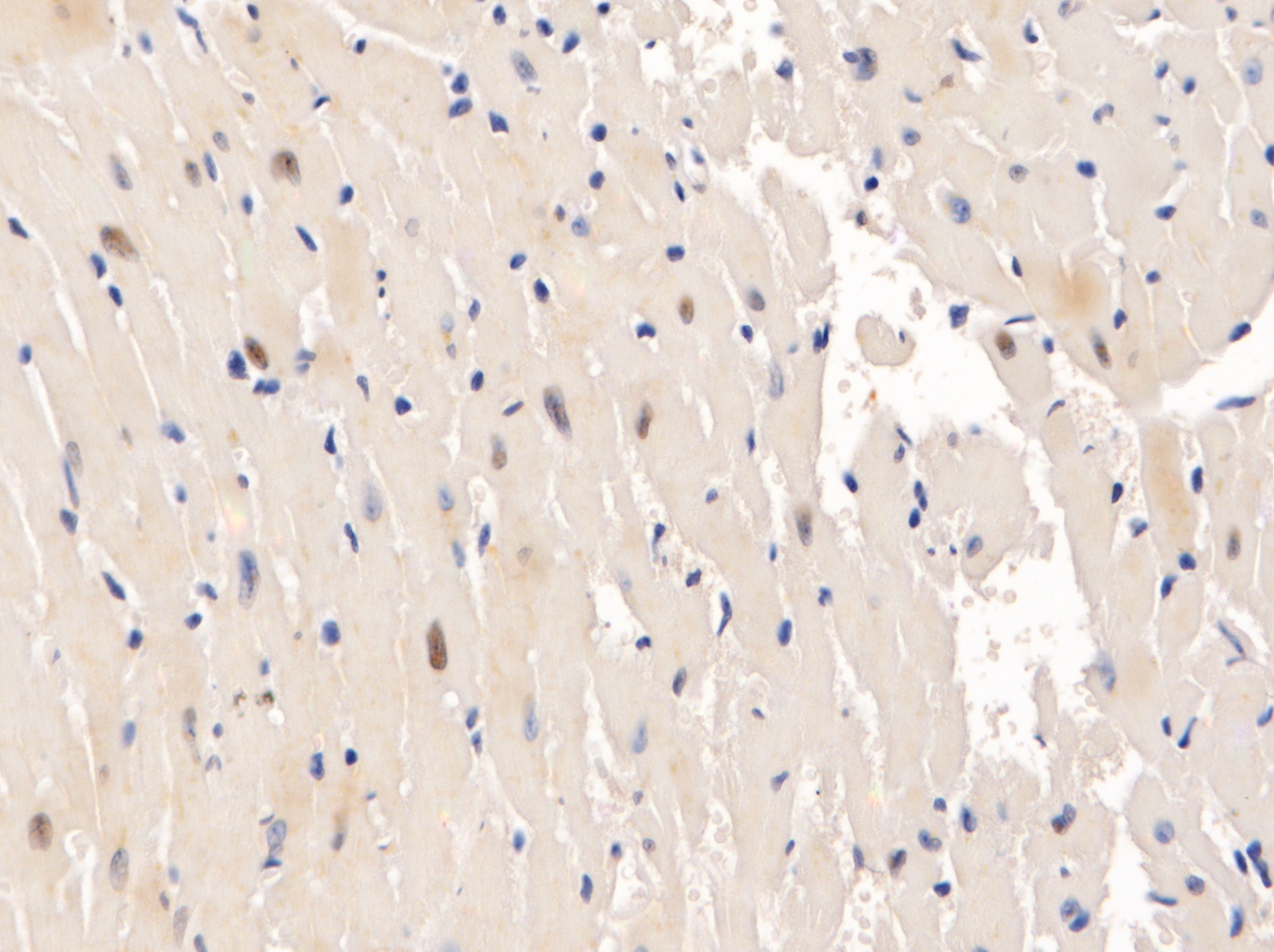

Supplement: Figure 4—source data 2. [file elife-55513-fig4-data2.zip › p19_images_for_eLife/p19_images_Ann_Chiao_for_eLife/Old controls/OCL_1/Copy of MS2_5_40x_RGB.jpg]

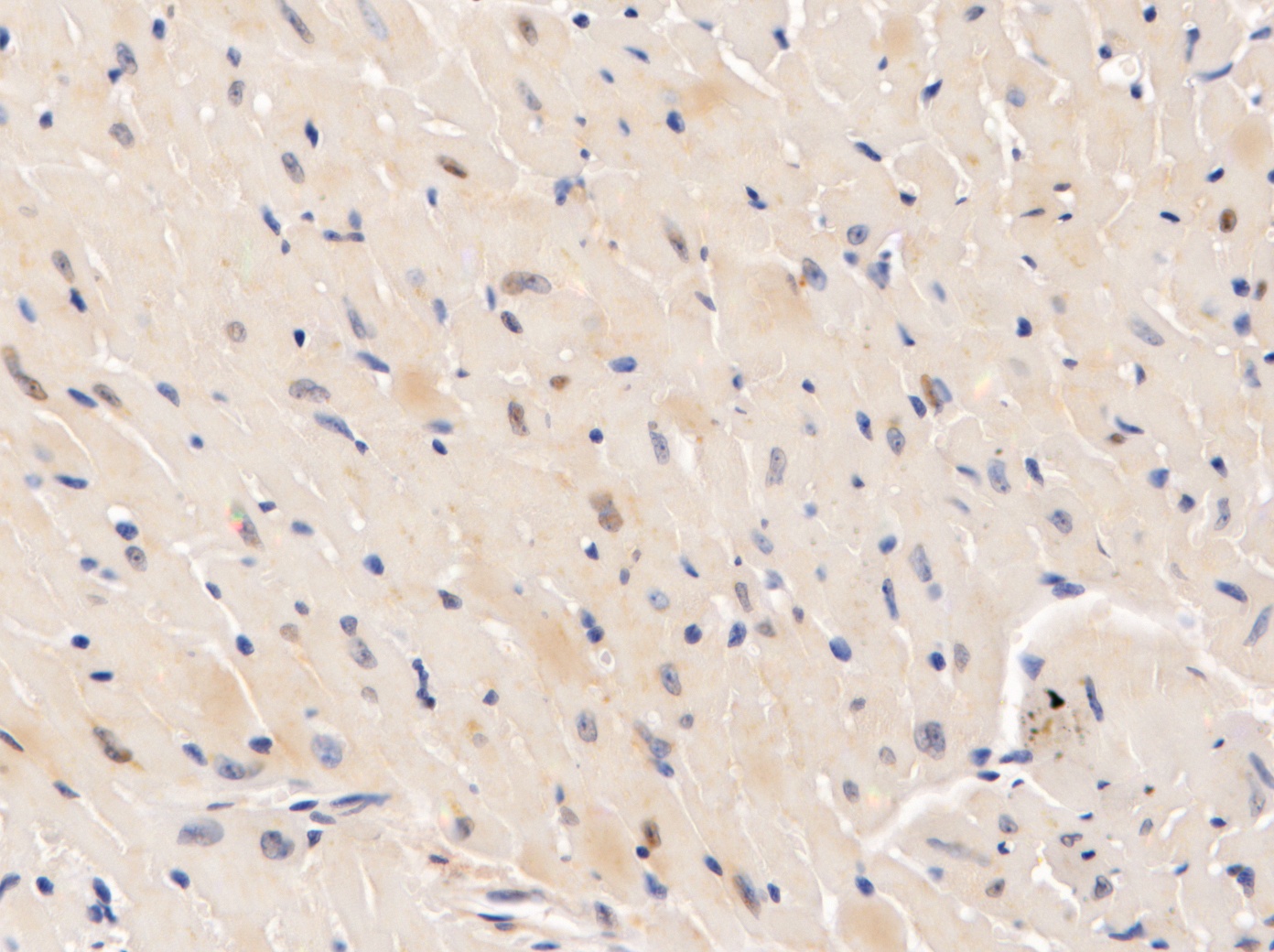

Supplement: Figure 4—source data 2. [file elife-55513-fig4-data2.zip › p19_images_for_eLife/p19_images_Ann_Chiao_for_eLife/Old controls/OCL_1/Copy of MS2_6_40x_RGB.jpg]

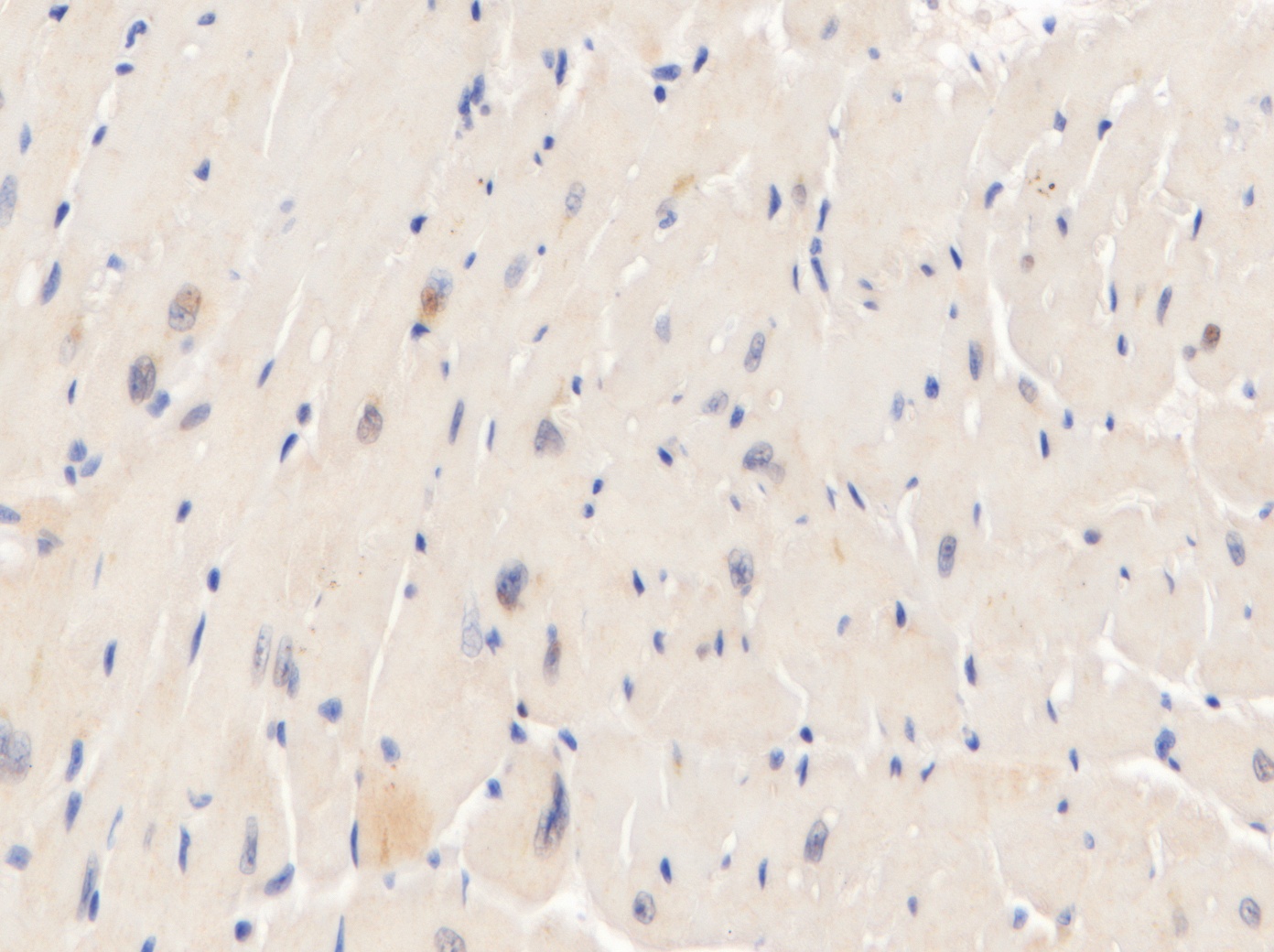

Supplement: Figure 4—source data 2. [file elife-55513-fig4-data2.zip › p19_images_for_eLife/p19_images_Ann_Chiao_for_eLife/Old controls/OCL_2/Copy of MS4_1_40x_RGB.jpg]

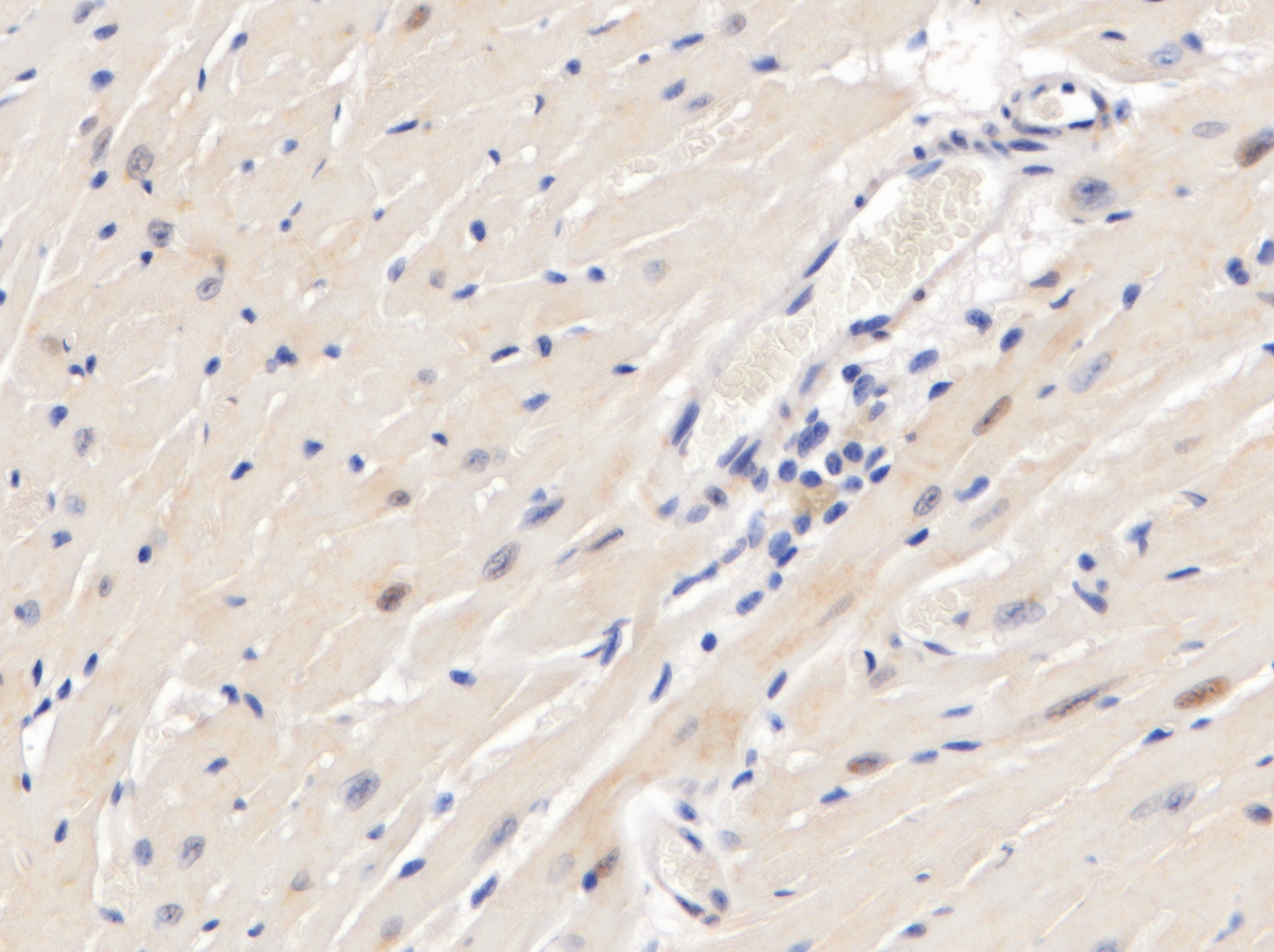

Supplement: Figure 4—source data 2. [file elife-55513-fig4-data2.zip › p19_images_for_eLife/p19_images_Ann_Chiao_for_eLife/Old controls/OCL_2/Copy of MS4_2_40x_RGB.jpg]

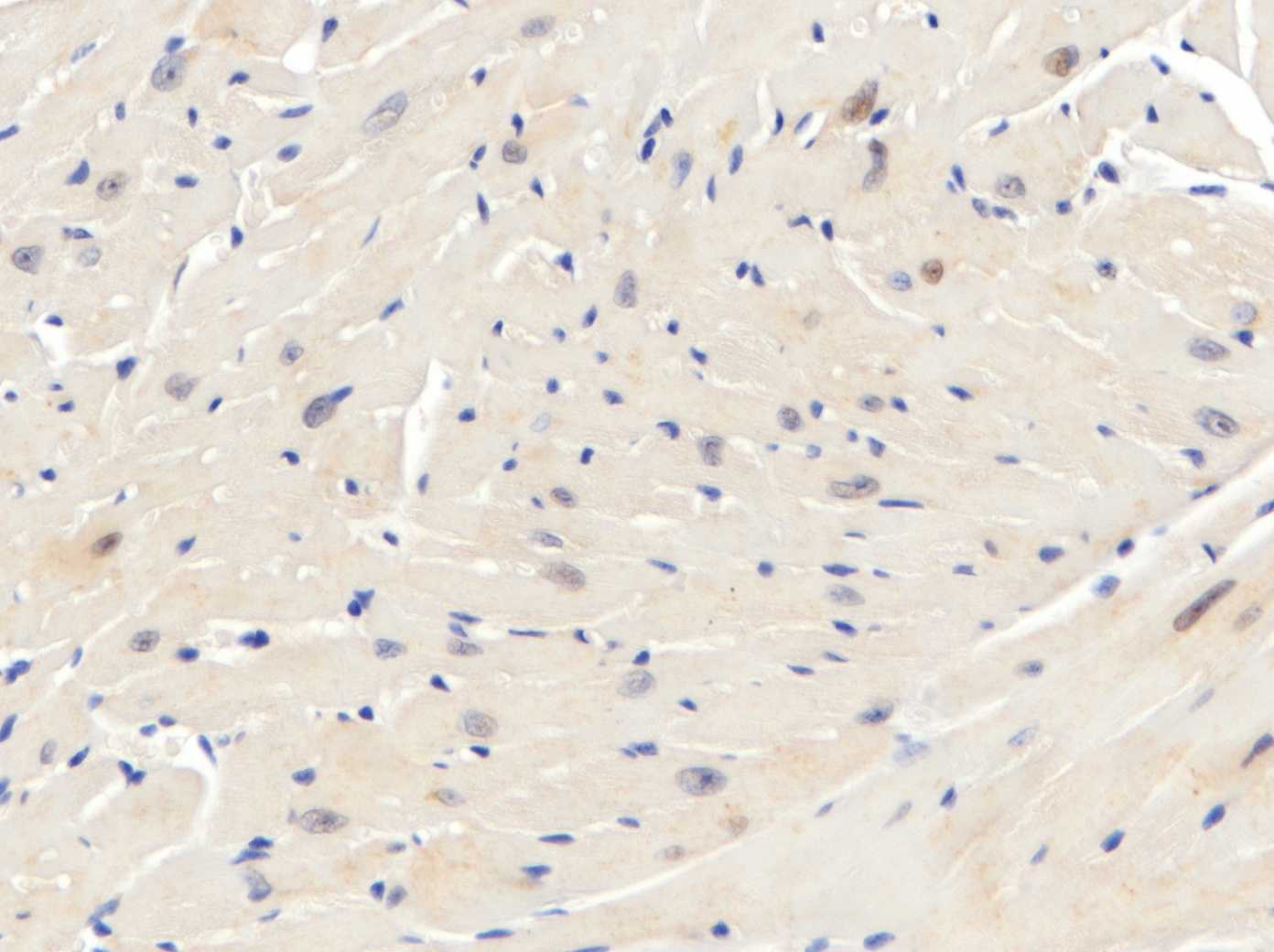

Supplement: Figure 4—source data 2. [file elife-55513-fig4-data2.zip › p19_images_for_eLife/p19_images_Ann_Chiao_for_eLife/Old controls/OCL_2/Copy of MS4_3_40x_RGB.jpg]

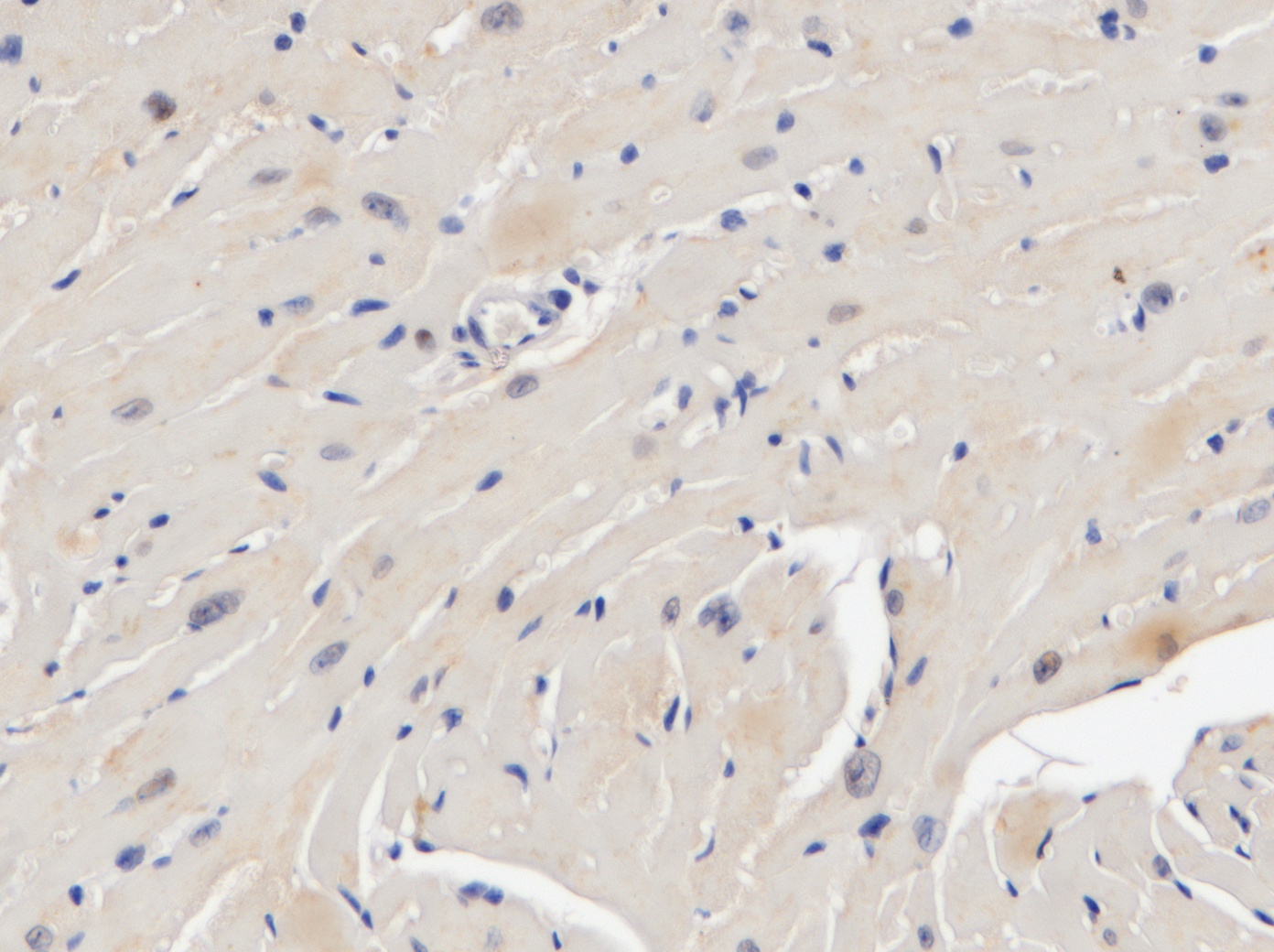

Supplement: Figure 4—source data 2. [file elife-55513-fig4-data2.zip › p19_images_for_eLife/p19_images_Ann_Chiao_for_eLife/Old controls/OCL_2/Copy of MS4_4_40x_RGB.jpg]

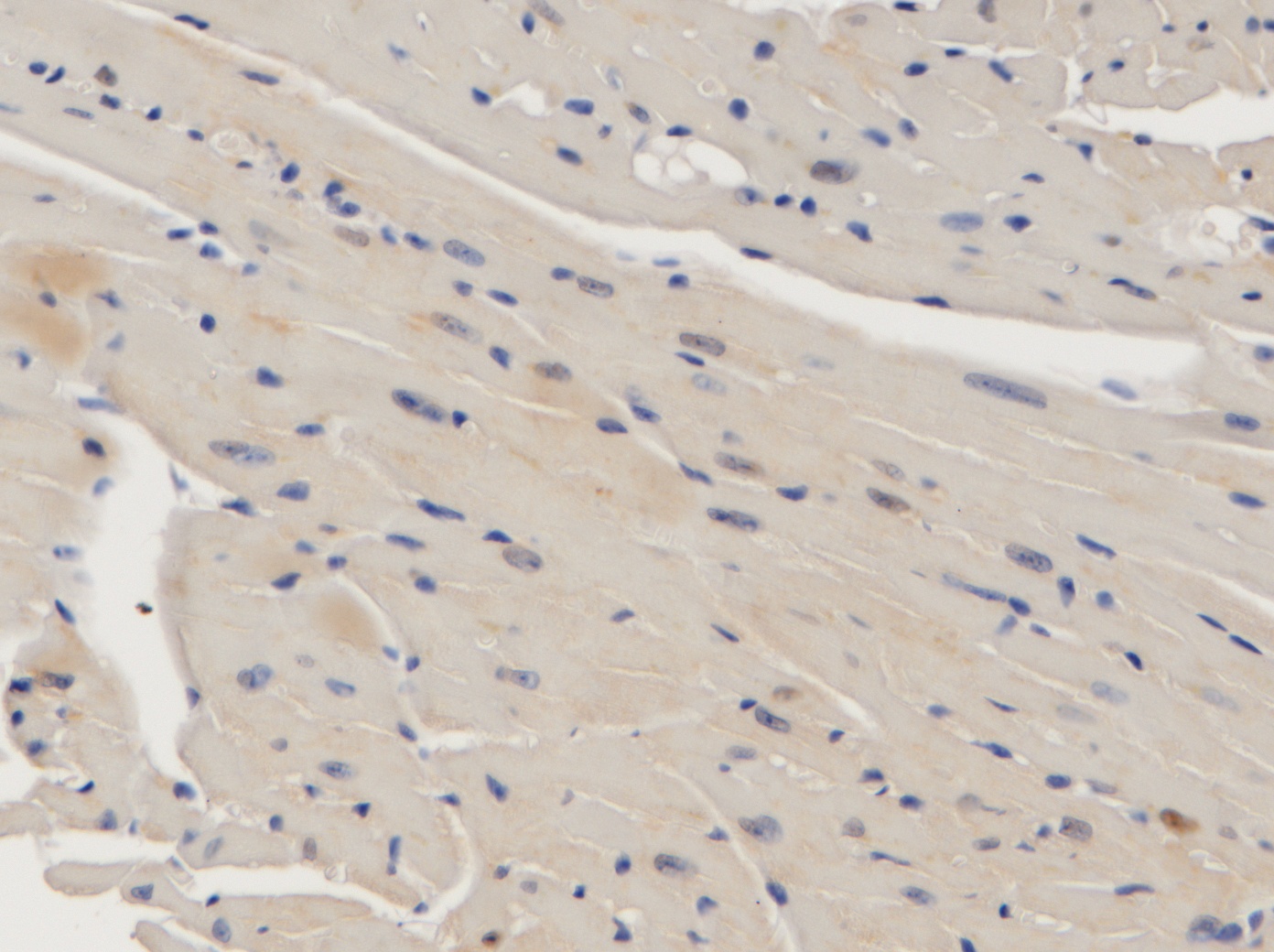

Supplement: Figure 4—source data 2. [file elife-55513-fig4-data2.zip › p19_images_for_eLife/p19_images_Ann_Chiao_for_eLife/Old controls/OCL_2/Copy of MS4_5_40x_RGB.jpg]

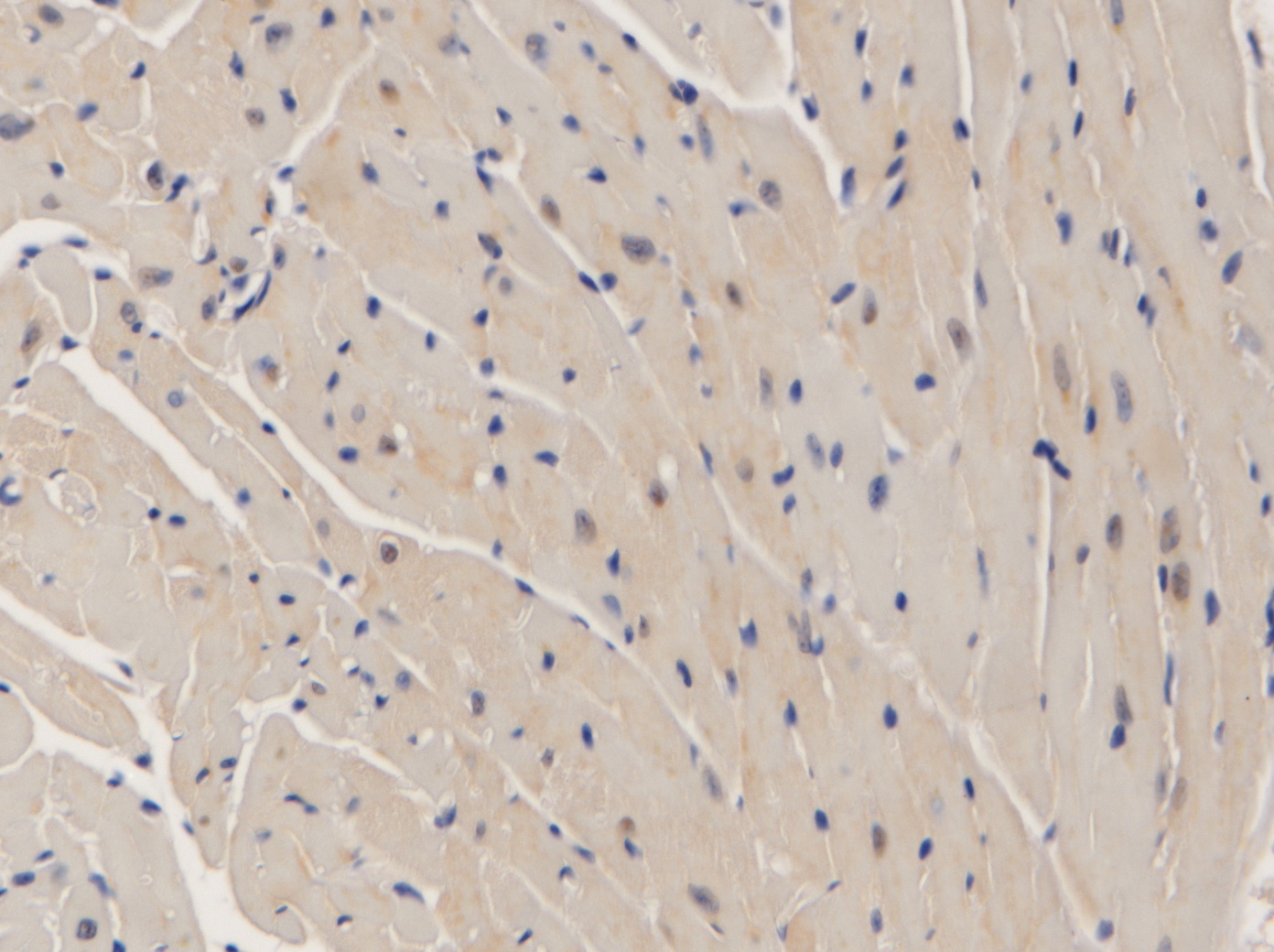

Supplement: Figure 4—source data 2. [file elife-55513-fig4-data2.zip › p19_images_for_eLife/p19_images_Ann_Chiao_for_eLife/Old controls/OCL_2/Copy of MS4_6_40x_RGB.jpg]

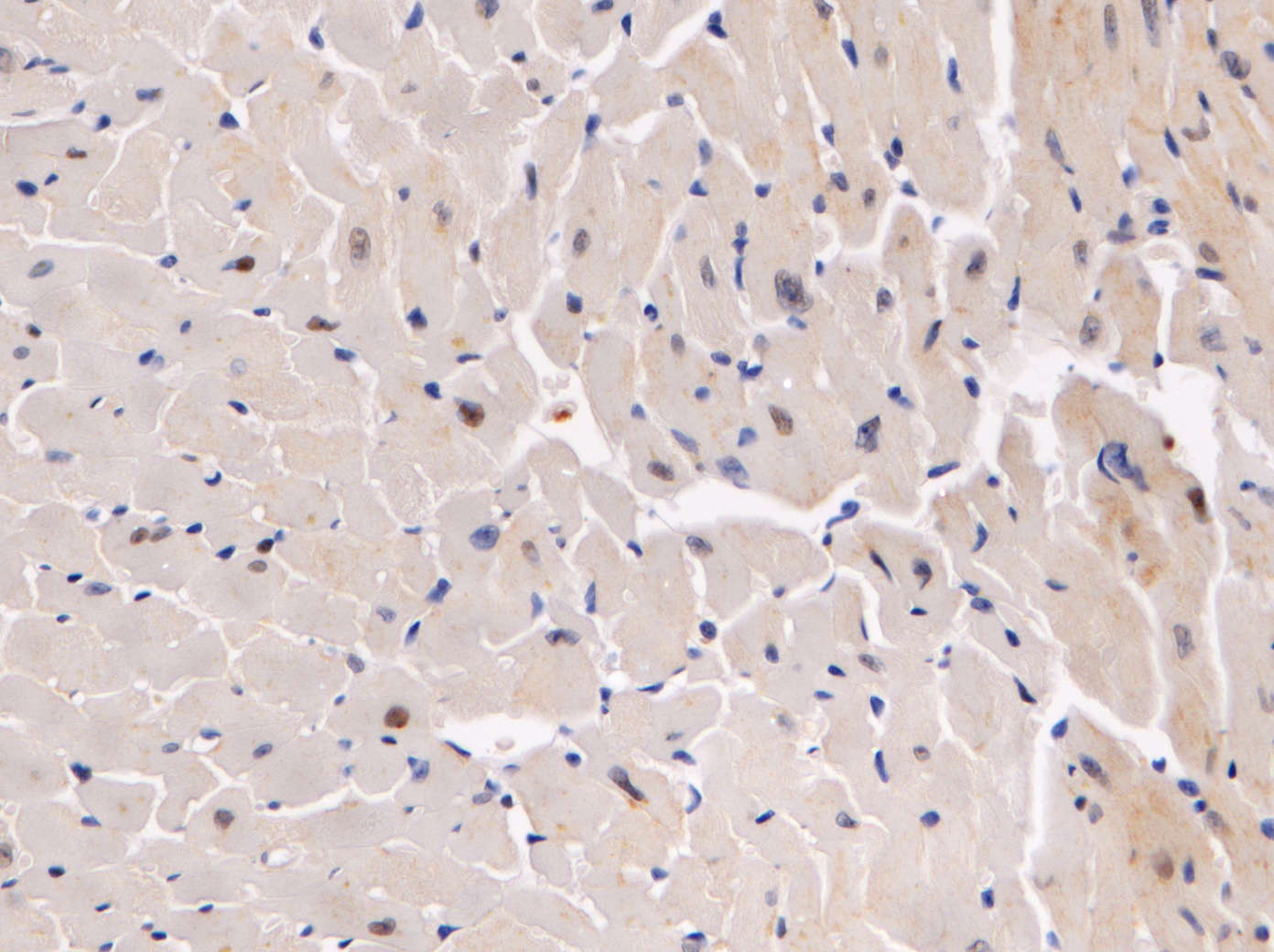

Supplement: Figure 4—source data 2. [file elife-55513-fig4-data2.zip › p19_images_for_eLife/p19_images_Ann_Chiao_for_eLife/Old controls/OCL_3/Copy of MS5_1_40x_RGB.jpg]

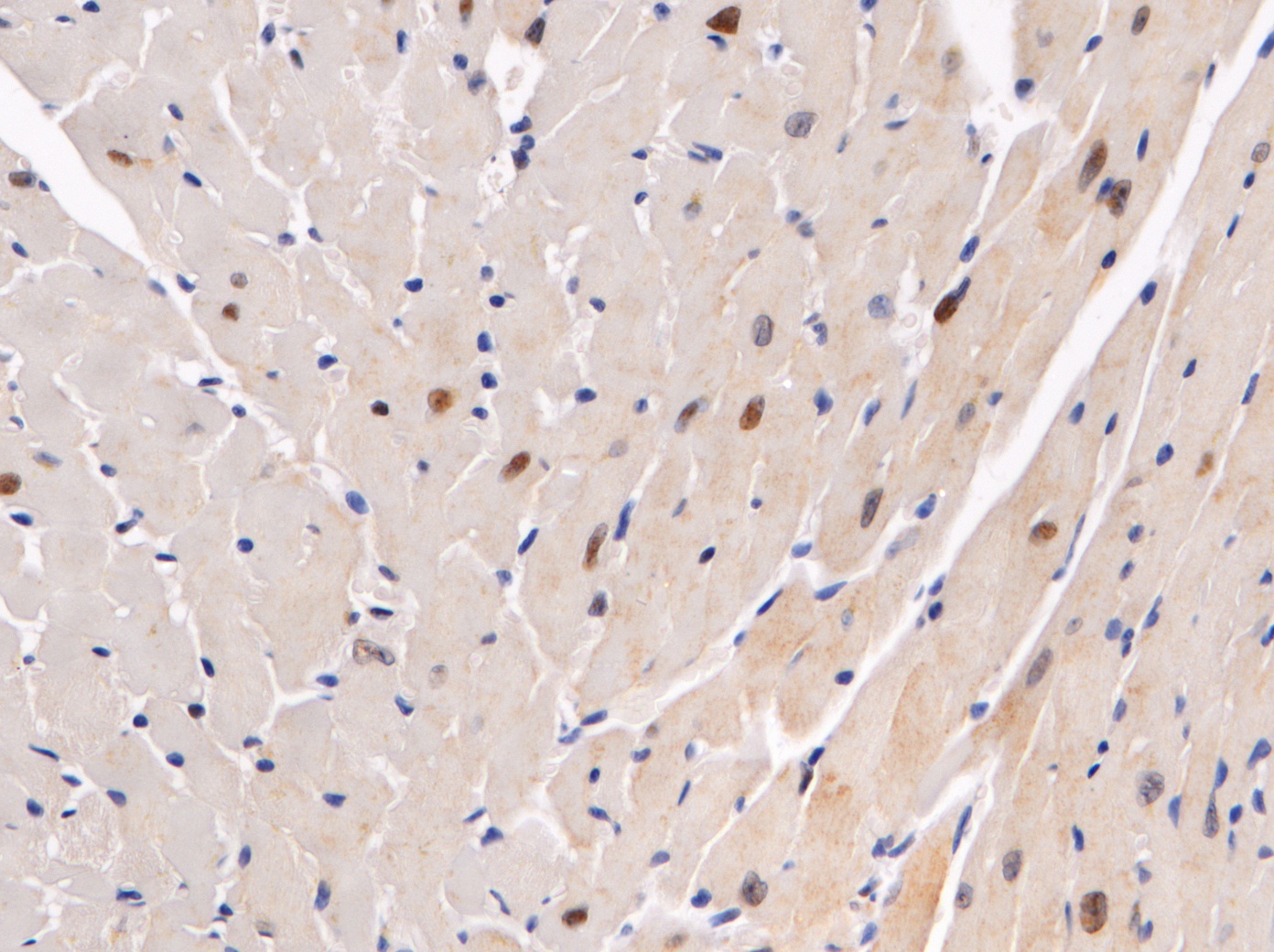

Supplement: Figure 4—source data 2. [file elife-55513-fig4-data2.zip › p19_images_for_eLife/p19_images_Ann_Chiao_for_eLife/Old controls/OCL_3/Copy of MS5_2_40x_RGB.jpg]

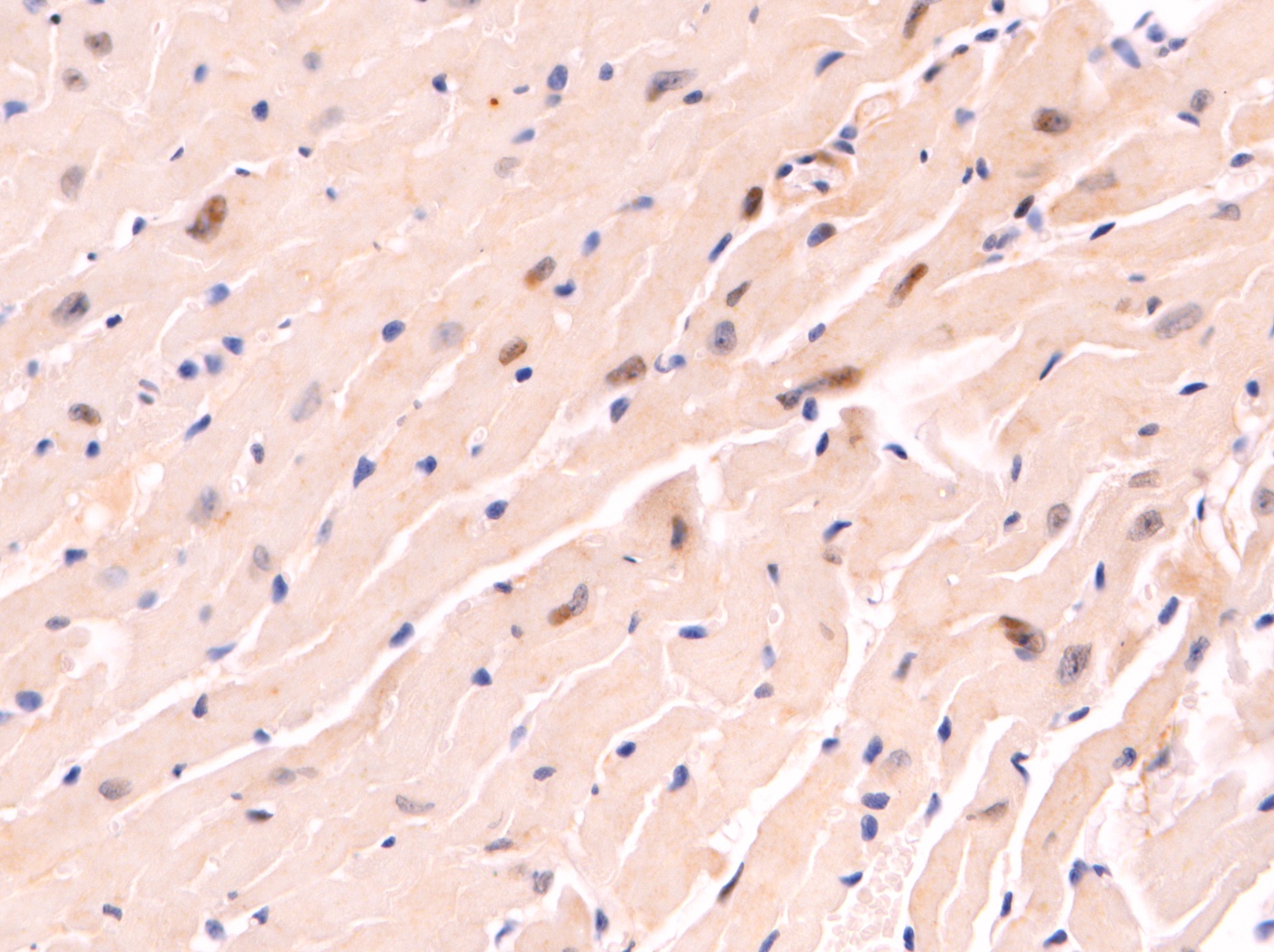

Supplement: Figure 4—source data 2. [file elife-55513-fig4-data2.zip › p19_images_for_eLife/p19_images_Ann_Chiao_for_eLife/Old controls/OCL_3/Copy of MS5_3_40x_RGB.jpg]

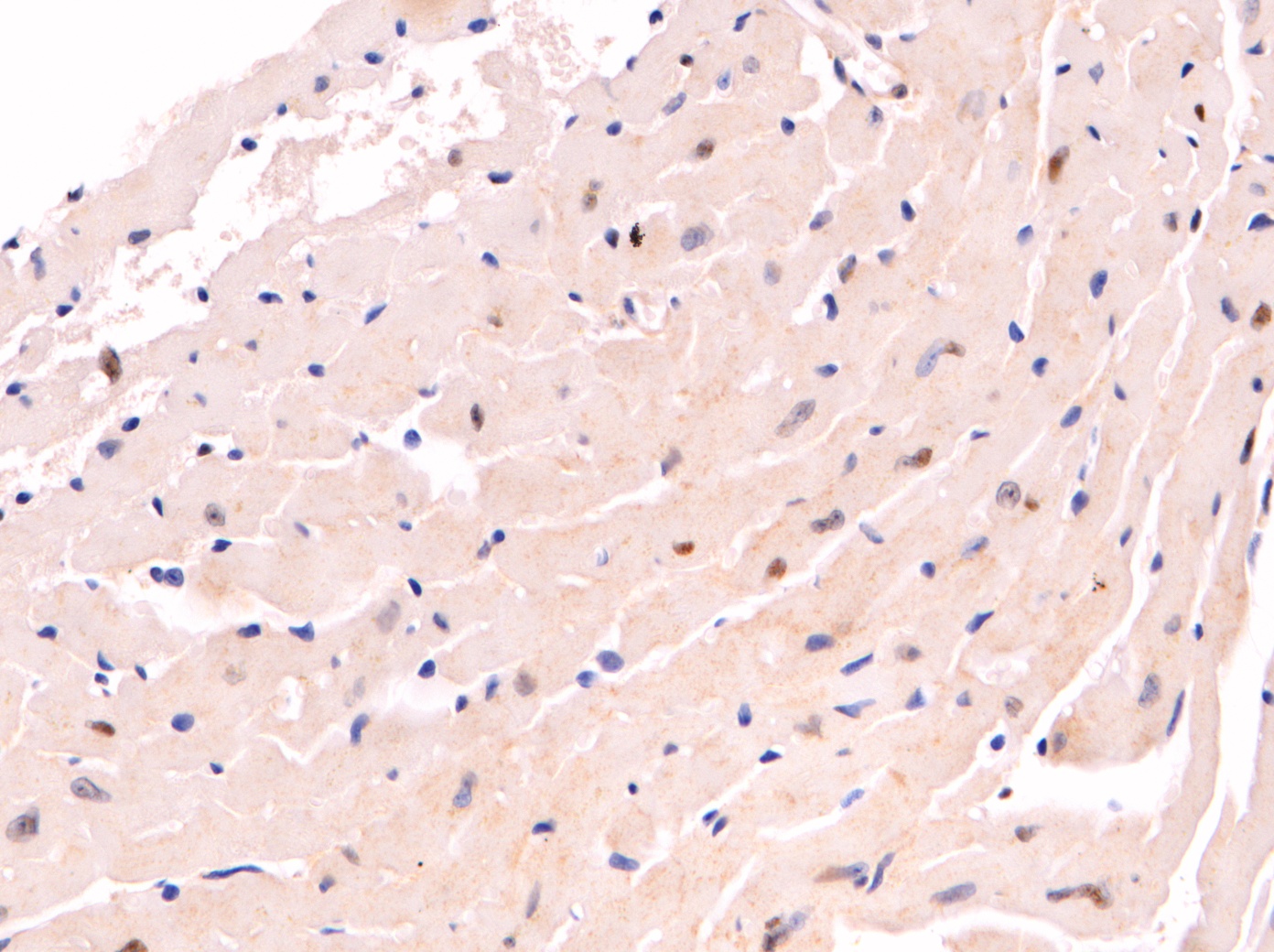

Supplement: Figure 4—source data 2. [file elife-55513-fig4-data2.zip › p19_images_for_eLife/p19_images_Ann_Chiao_for_eLife/Old controls/OCL_3/Copy of MS5_4_40x_RGB.jpg]

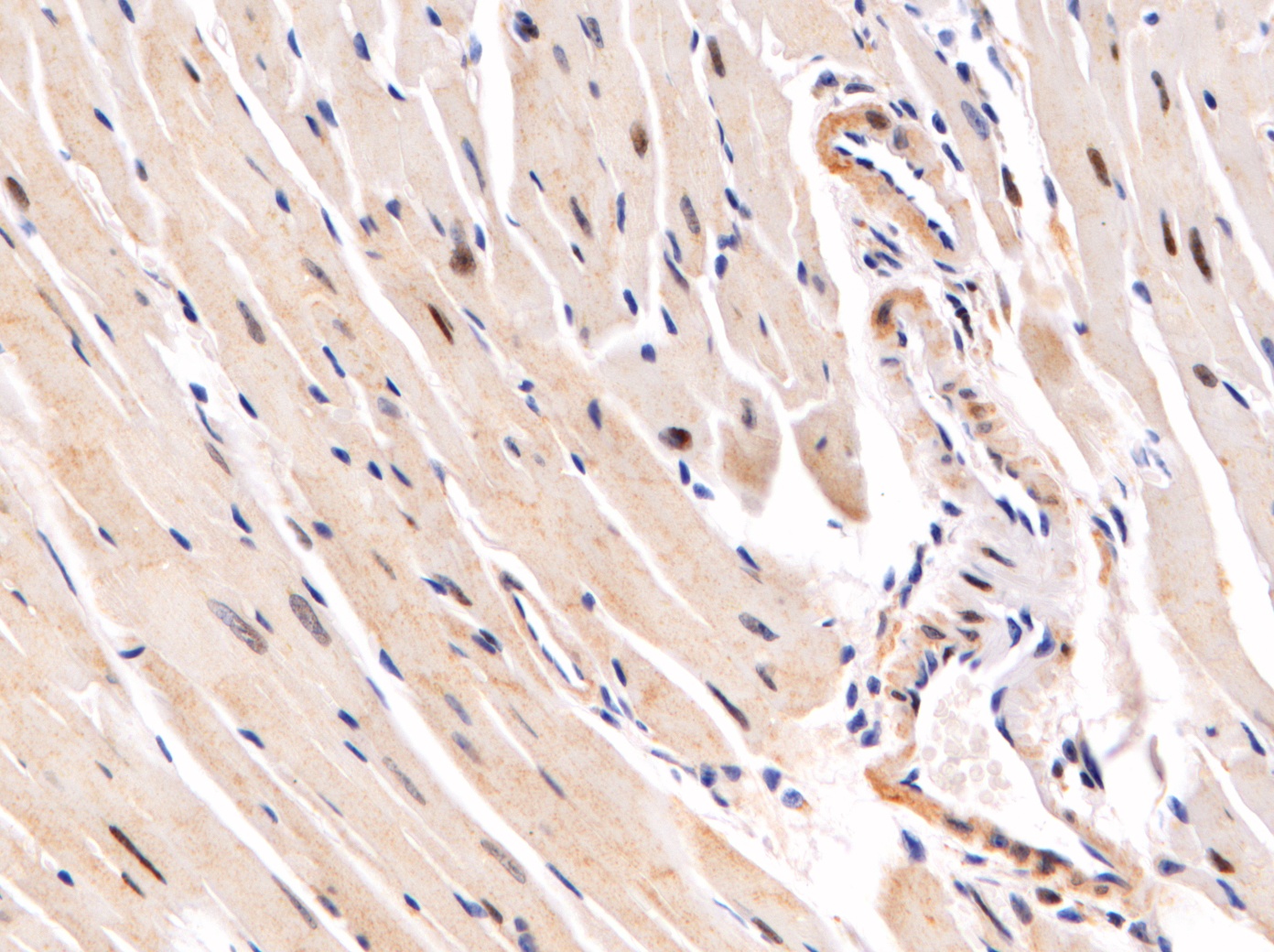

Supplement: Figure 4—source data 2. [file elife-55513-fig4-data2.zip › p19_images_for_eLife/p19_images_Ann_Chiao_for_eLife/Old controls/OCL_3/Copy of MS5_5_40x_RGB.jpg]

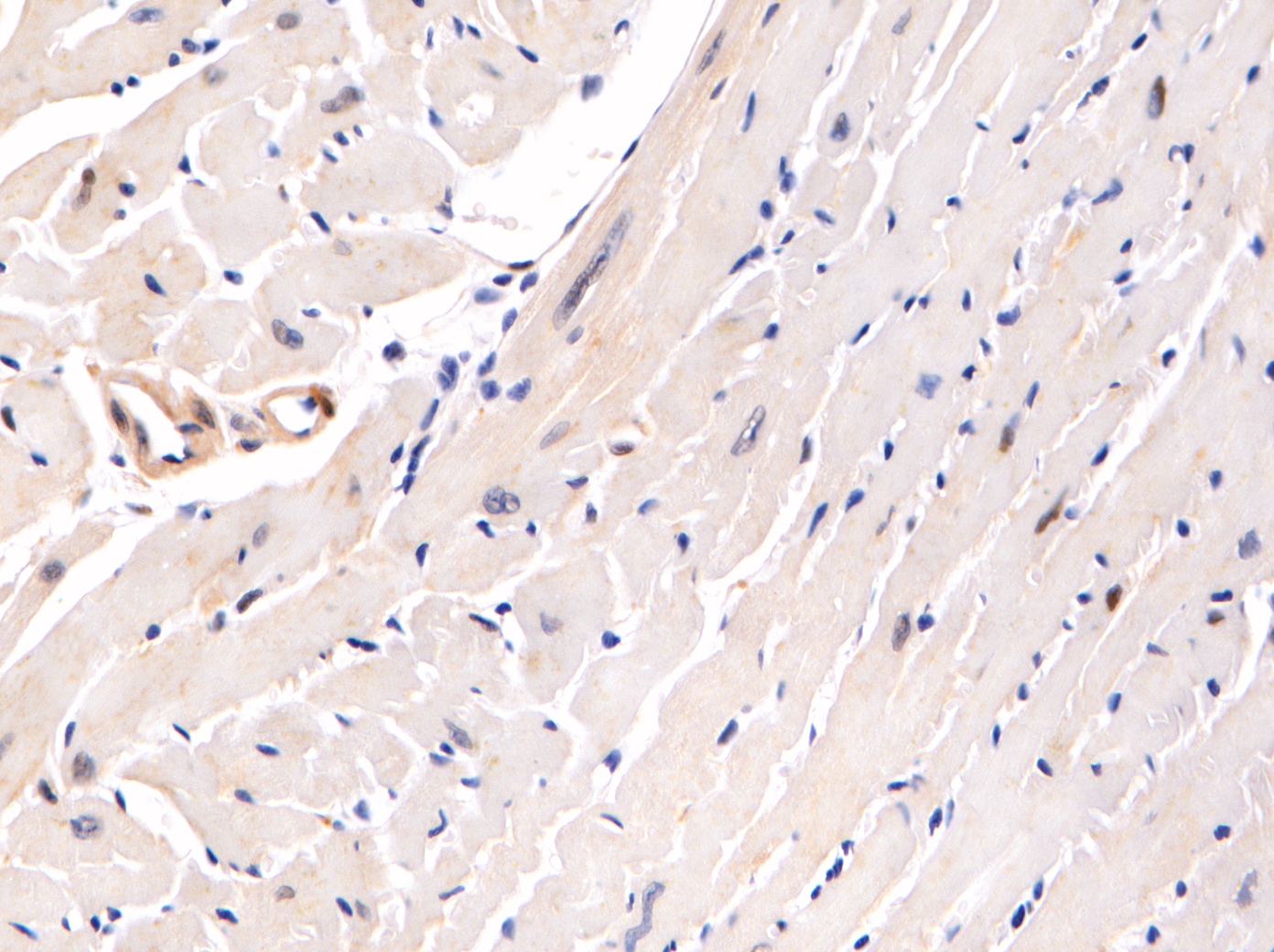

Supplement: Figure 4—source data 2. [file elife-55513-fig4-data2.zip › p19_images_for_eLife/p19_images_Ann_Chiao_for_eLife/Old controls/OCL_3/Copy of MS5_6_40x_RGB.jpg]

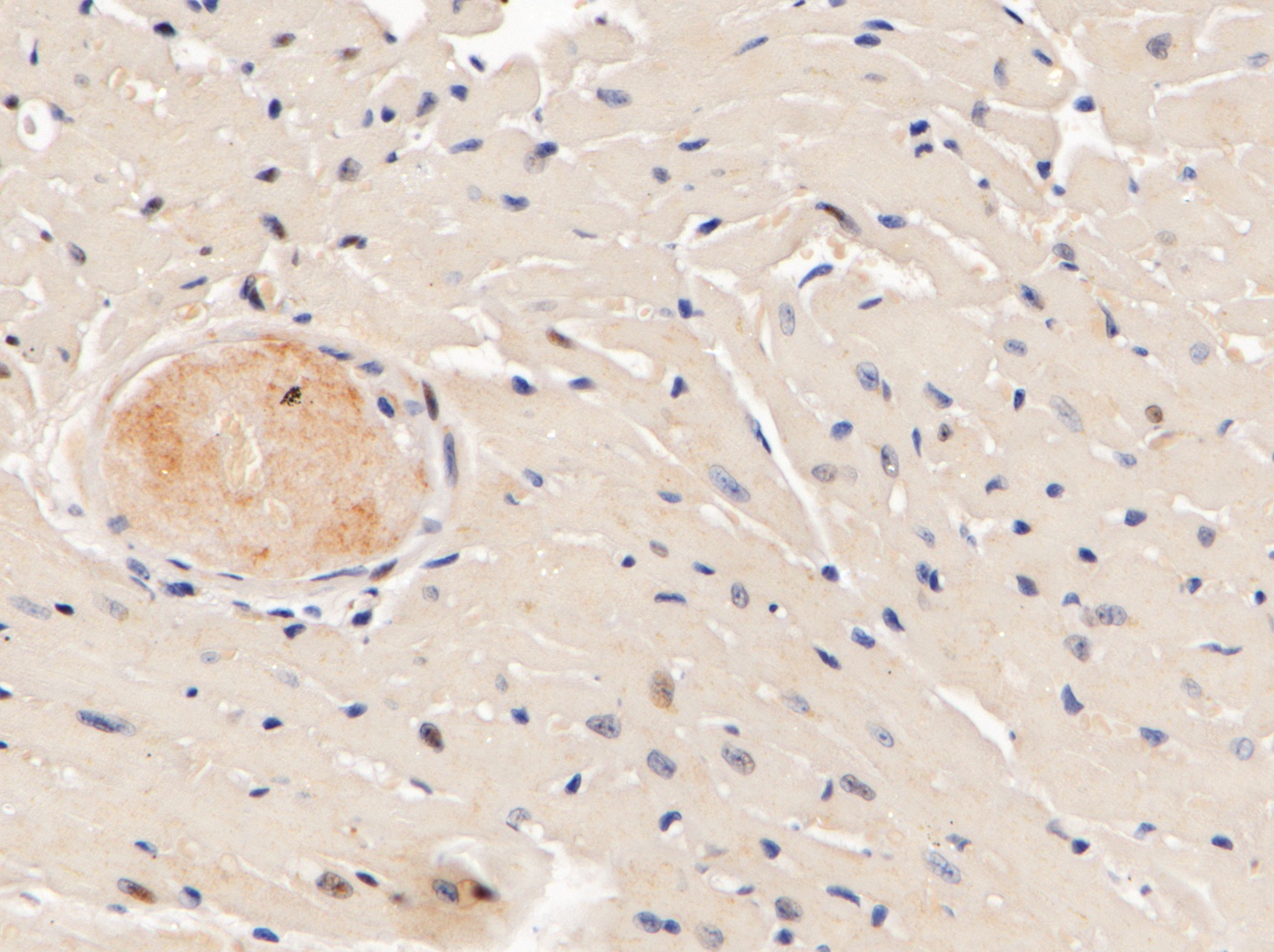

Supplement: Figure 4—source data 2. [file elife-55513-fig4-data2.zip › p19_images_for_eLife/p19_images_Ann_Chiao_for_eLife/Old controls/OCL_4/Copy of MS7_1_40x_RGB.jpg]

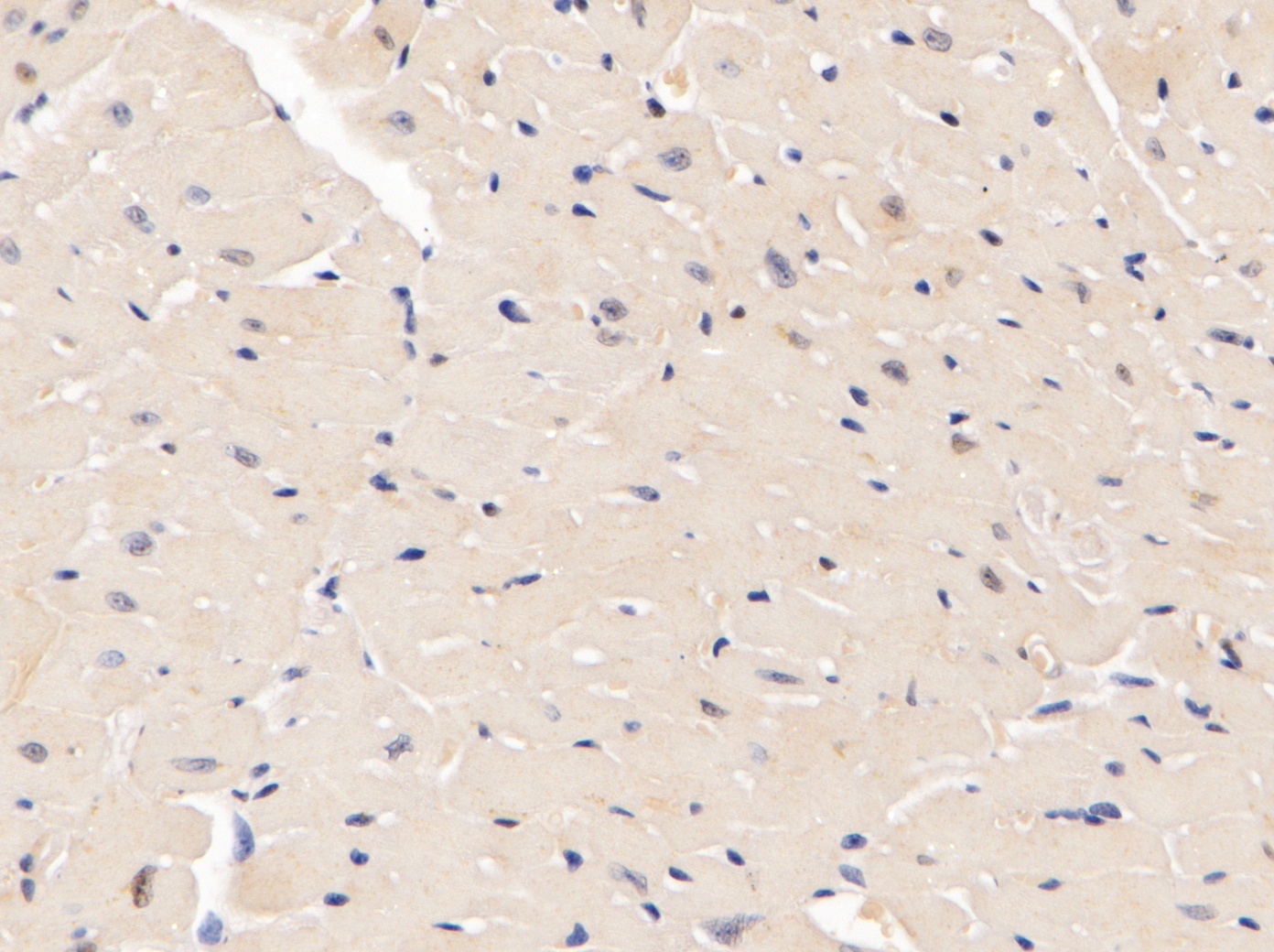

Supplement: Figure 4—source data 2. [file elife-55513-fig4-data2.zip › p19_images_for_eLife/p19_images_Ann_Chiao_for_eLife/Old controls/OCL_4/Copy of MS7_2_40x_RGB.jpg]

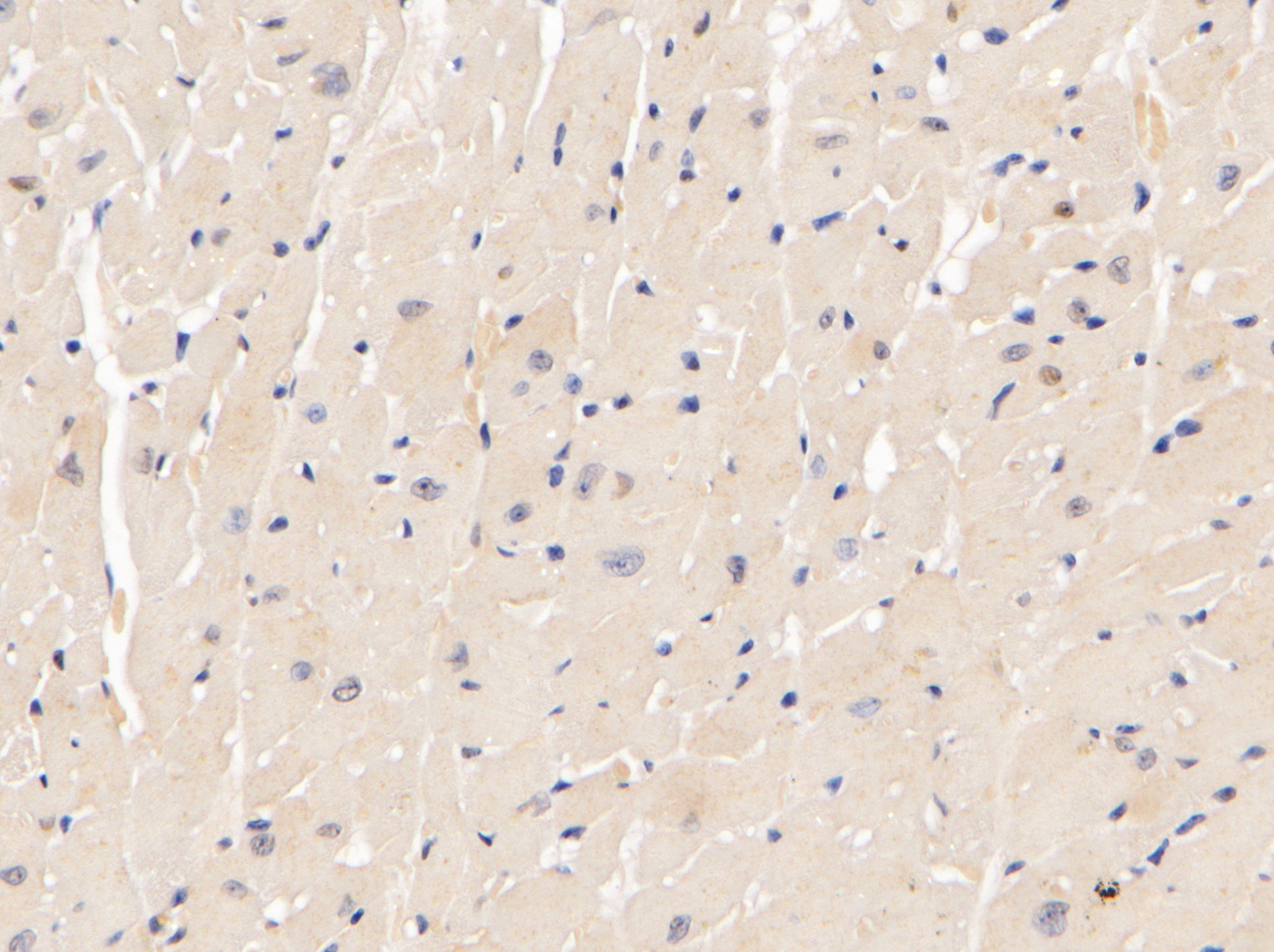

Supplement: Figure 4—source data 2. [file elife-55513-fig4-data2.zip › p19_images_for_eLife/p19_images_Ann_Chiao_for_eLife/Old controls/OCL_4/Copy of MS7_3_40x_RGB.jpg]

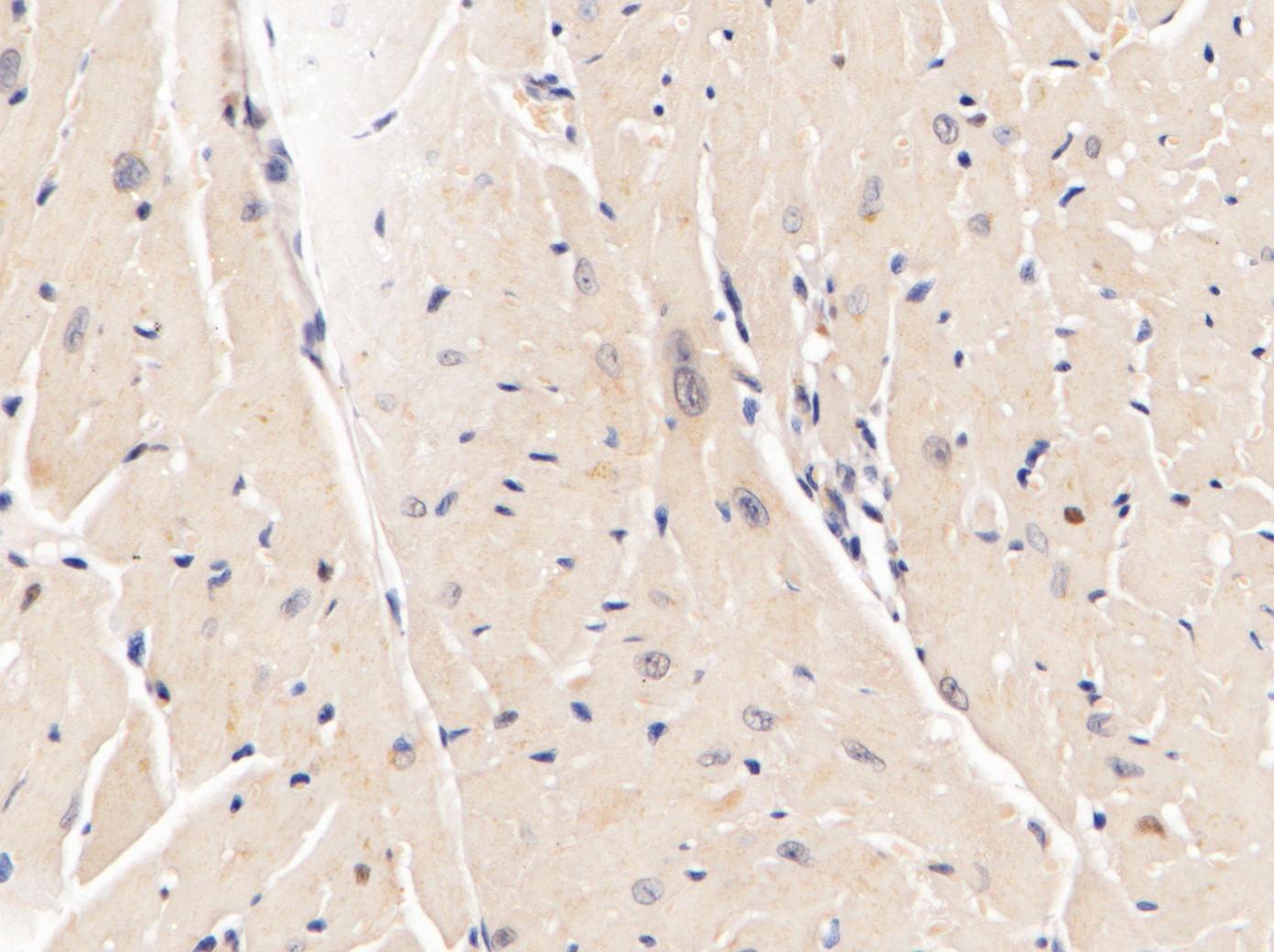

Supplement: Figure 4—source data 2. [file elife-55513-fig4-data2.zip › p19_images_for_eLife/p19_images_Ann_Chiao_for_eLife/Old controls/OCL_4/Copy of MS7_4_40x_RGB.jpg]

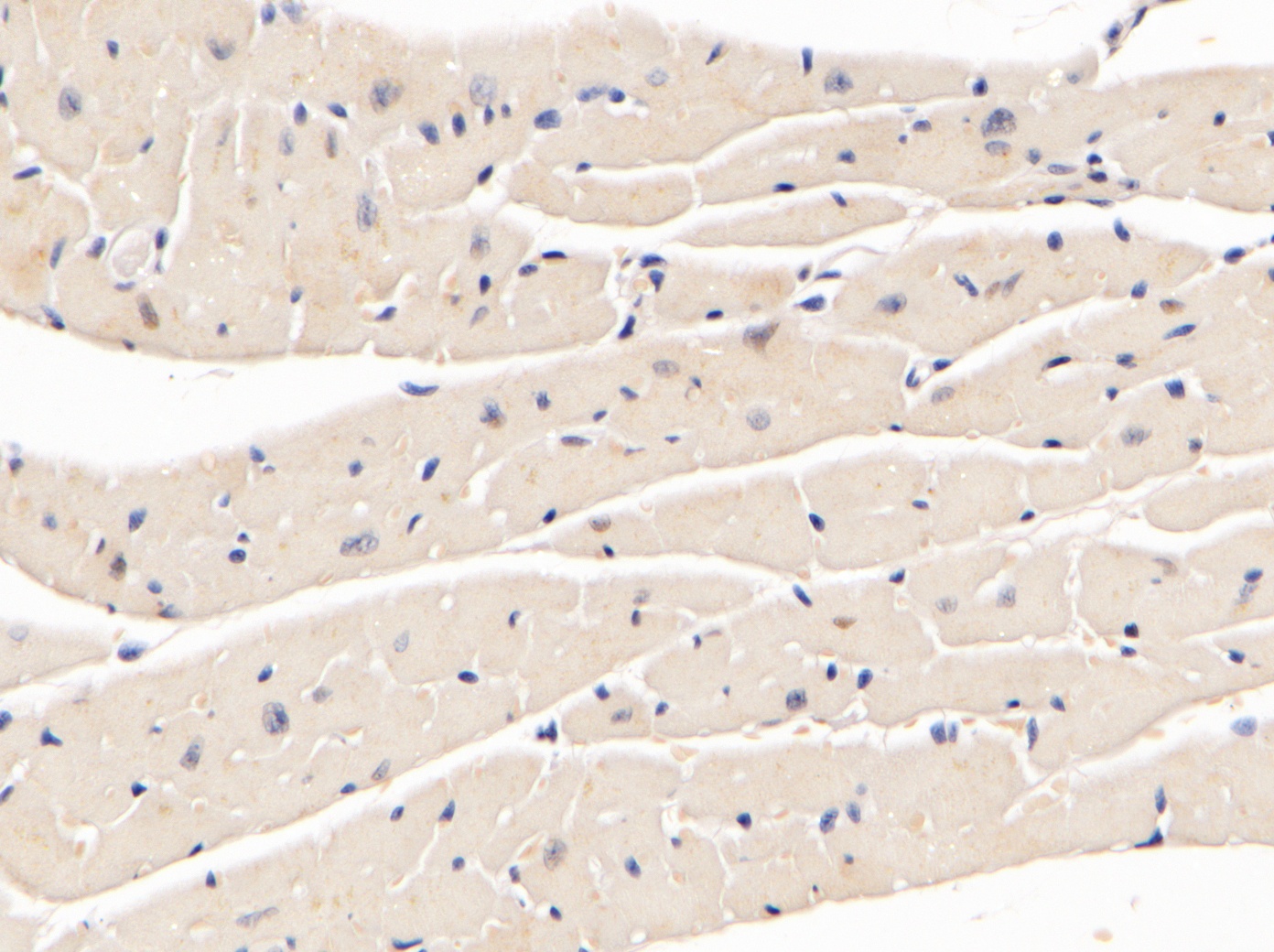

Supplement: Figure 4—source data 2. [file elife-55513-fig4-data2.zip › p19_images_for_eLife/p19_images_Ann_Chiao_for_eLife/Old controls/OCL_4/Copy of MS7_5_40x_RGB.jpg]

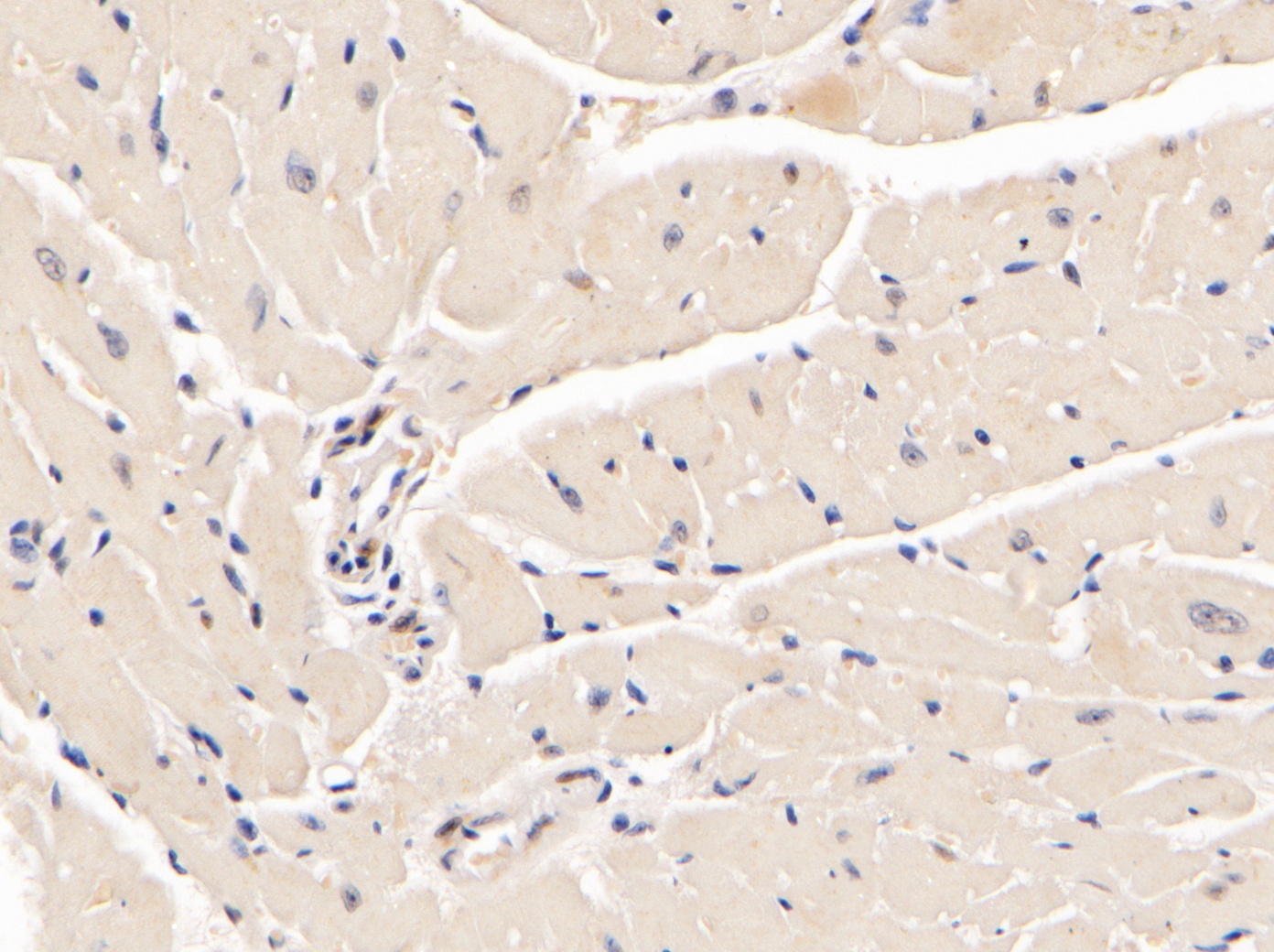

Supplement: Figure 4—source data 2. [file elife-55513-fig4-data2.zip › p19_images_for_eLife/p19_images_Ann_Chiao_for_eLife/Old controls/OCL_4/Copy of MS7_6_40x_RGB.jpg]

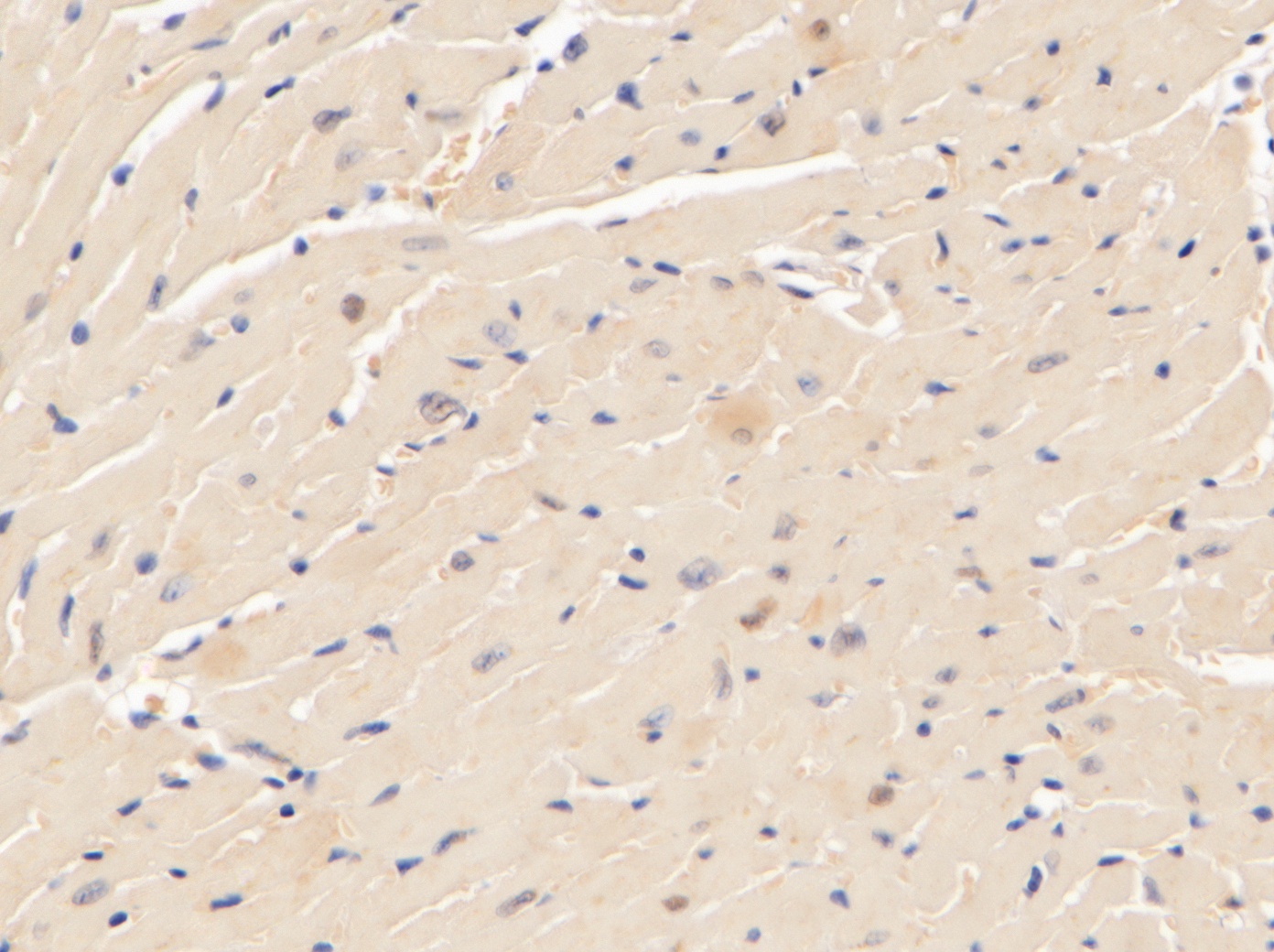

Supplement: Figure 4—source data 2. [file elife-55513-fig4-data2.zip › p19_images_for_eLife/p19_images_Ann_Chiao_for_eLife/Old controls/OCL_5/Copy of MS10_1_40x_RGB.jpg]

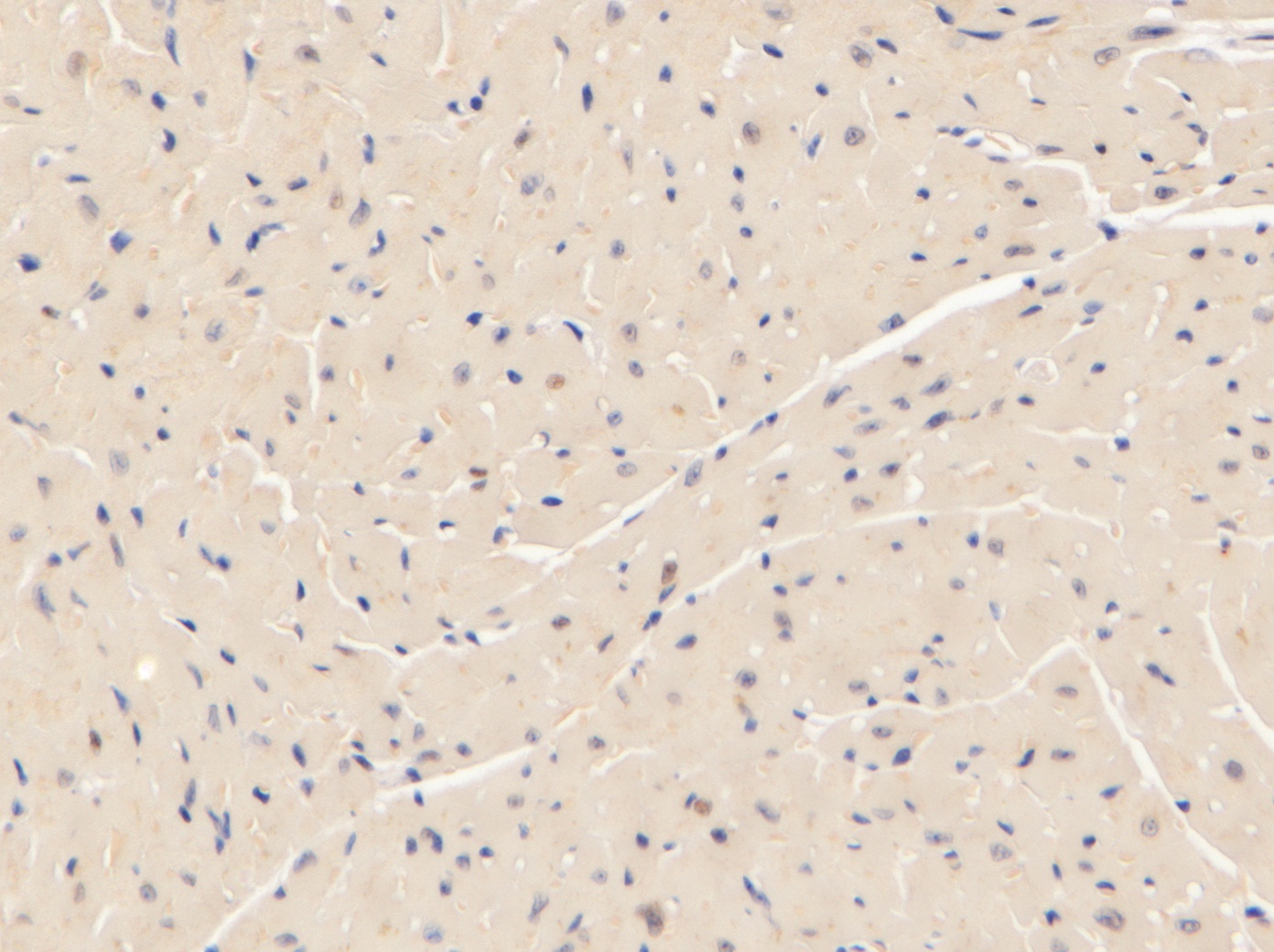

Supplement: Figure 4—source data 2. [file elife-55513-fig4-data2.zip › p19_images_for_eLife/p19_images_Ann_Chiao_for_eLife/Old controls/OCL_5/Copy of MS10_2_40x_RGB.jpg]

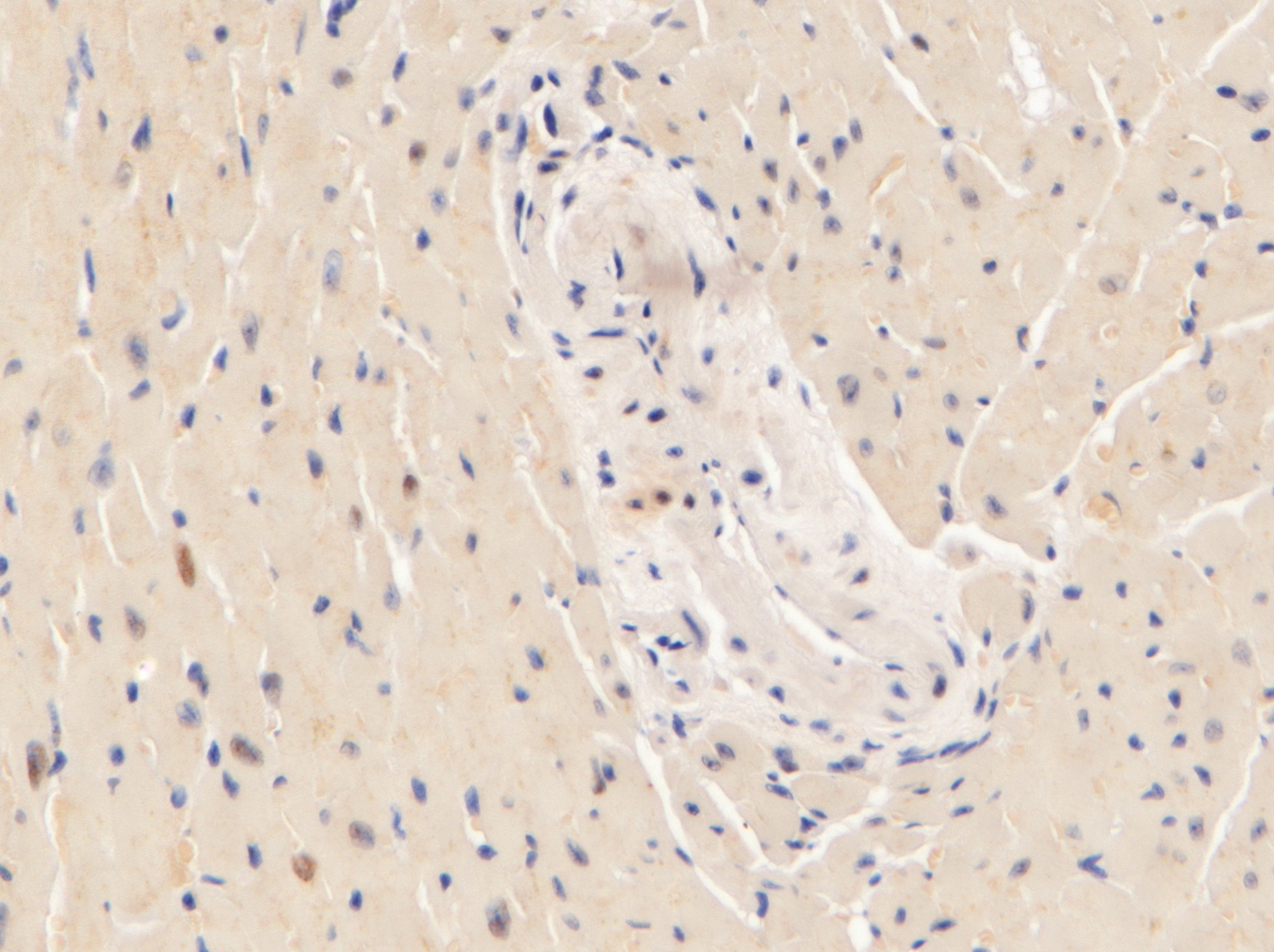

Supplement: Figure 4—source data 2. [file elife-55513-fig4-data2.zip › p19_images_for_eLife/p19_images_Ann_Chiao_for_eLife/Old controls/OCL_5/Copy of MS10_3_40x_RGB.jpg]

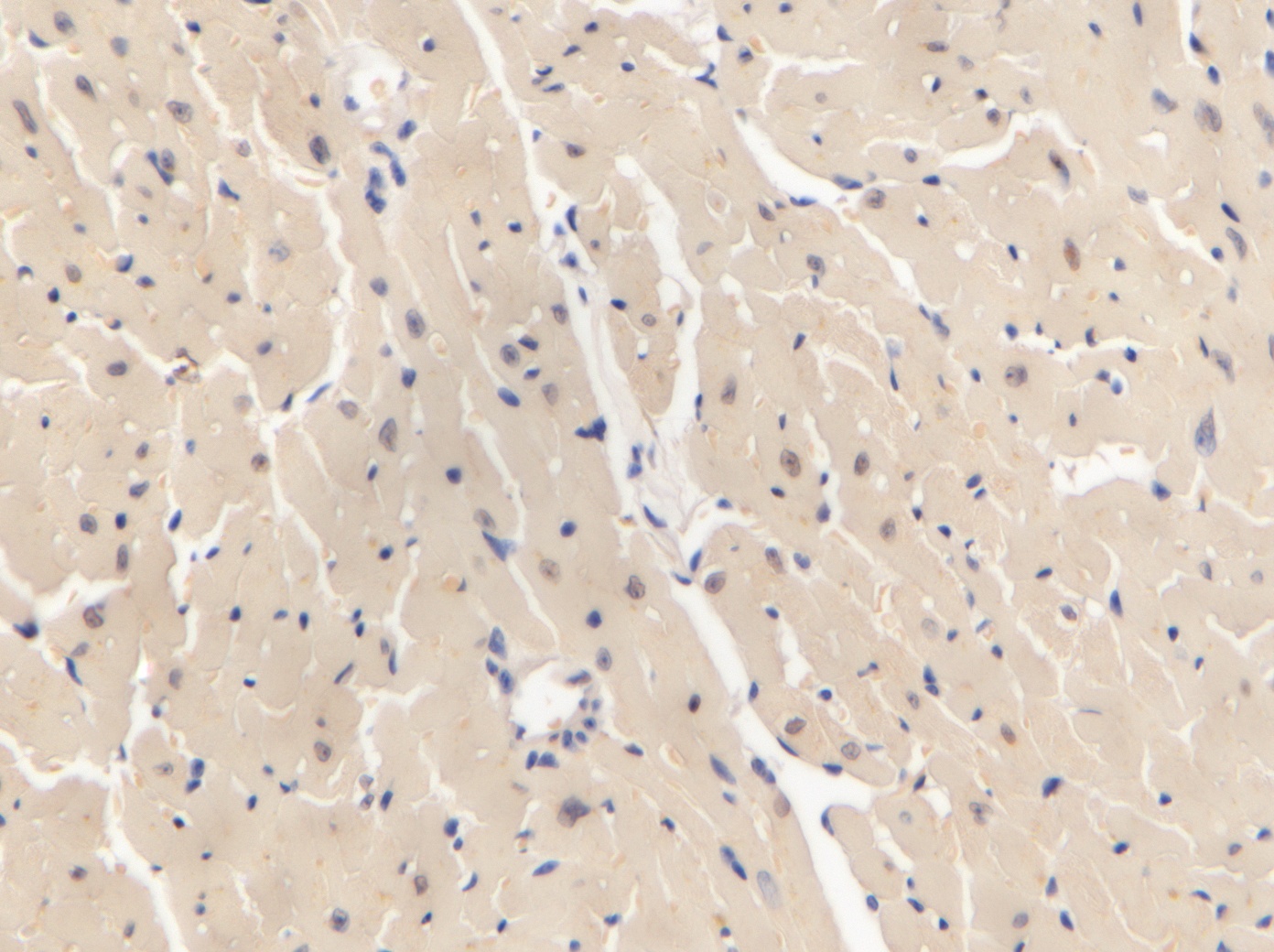

Supplement: Figure 4—source data 2. [file elife-55513-fig4-data2.zip › p19_images_for_eLife/p19_images_Ann_Chiao_for_eLife/Old controls/OCL_5/Copy of MS10_4_40x_RGB.jpg]

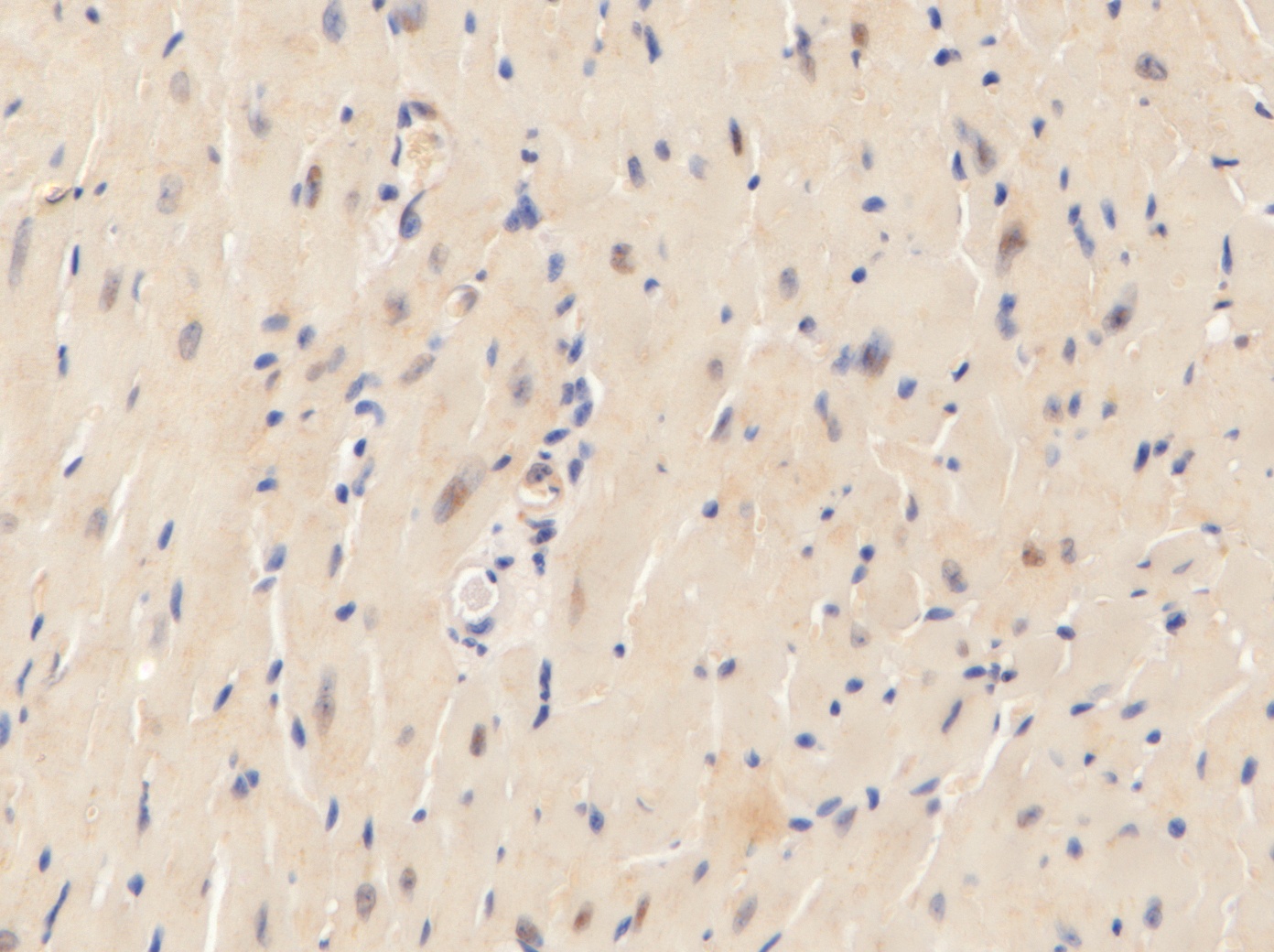

Supplement: Figure 4—source data 2. [file elife-55513-fig4-data2.zip › p19_images_for_eLife/p19_images_Ann_Chiao_for_eLife/Old controls/OCL_5/Copy of MS10_5_40x_RGB.jpg]

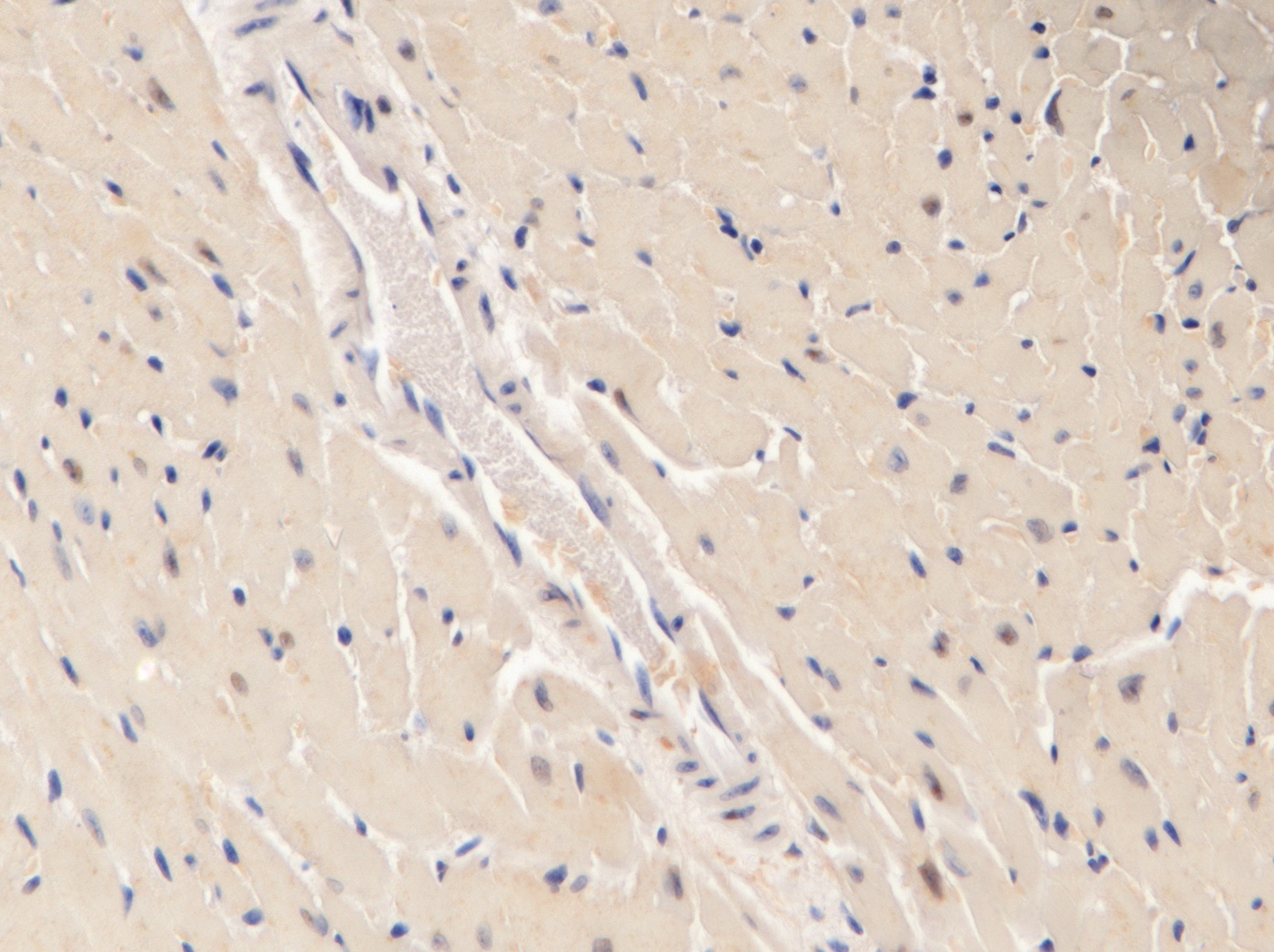

Supplement: Figure 4—source data 2. [file elife-55513-fig4-data2.zip › p19_images_for_eLife/p19_images_Ann_Chiao_for_eLife/Old controls/OCL_5/Copy of MS10_6_40x_RGB.jpg]

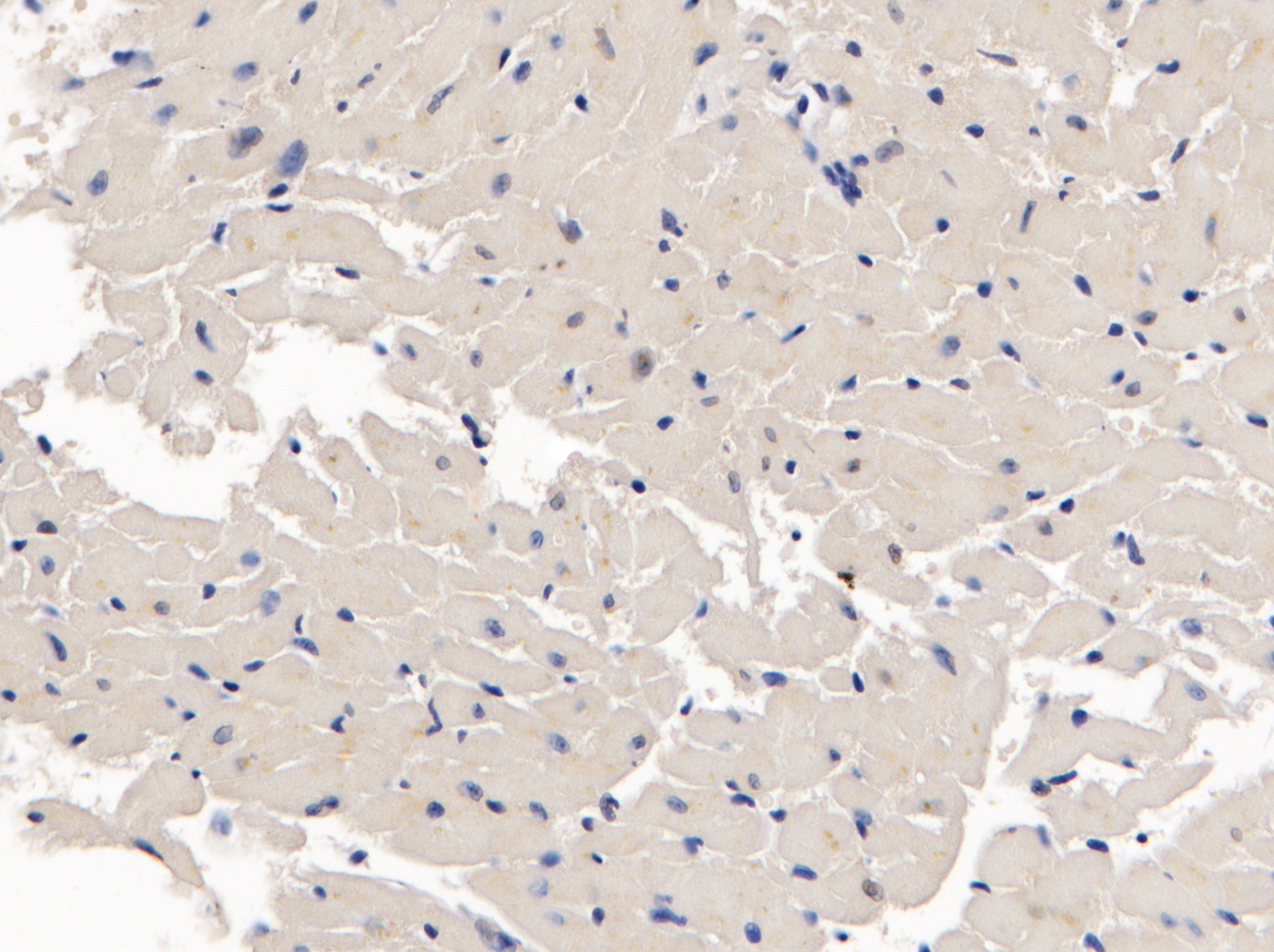

Supplement: Figure 4—source data 2. [file elife-55513-fig4-data2.zip › p19_images_for_eLife/p19_images_Ann_Chiao_for_eLife/Old SS-31 treated/OSS_1/Copy of MS1_1_40x_RGB.jpg]

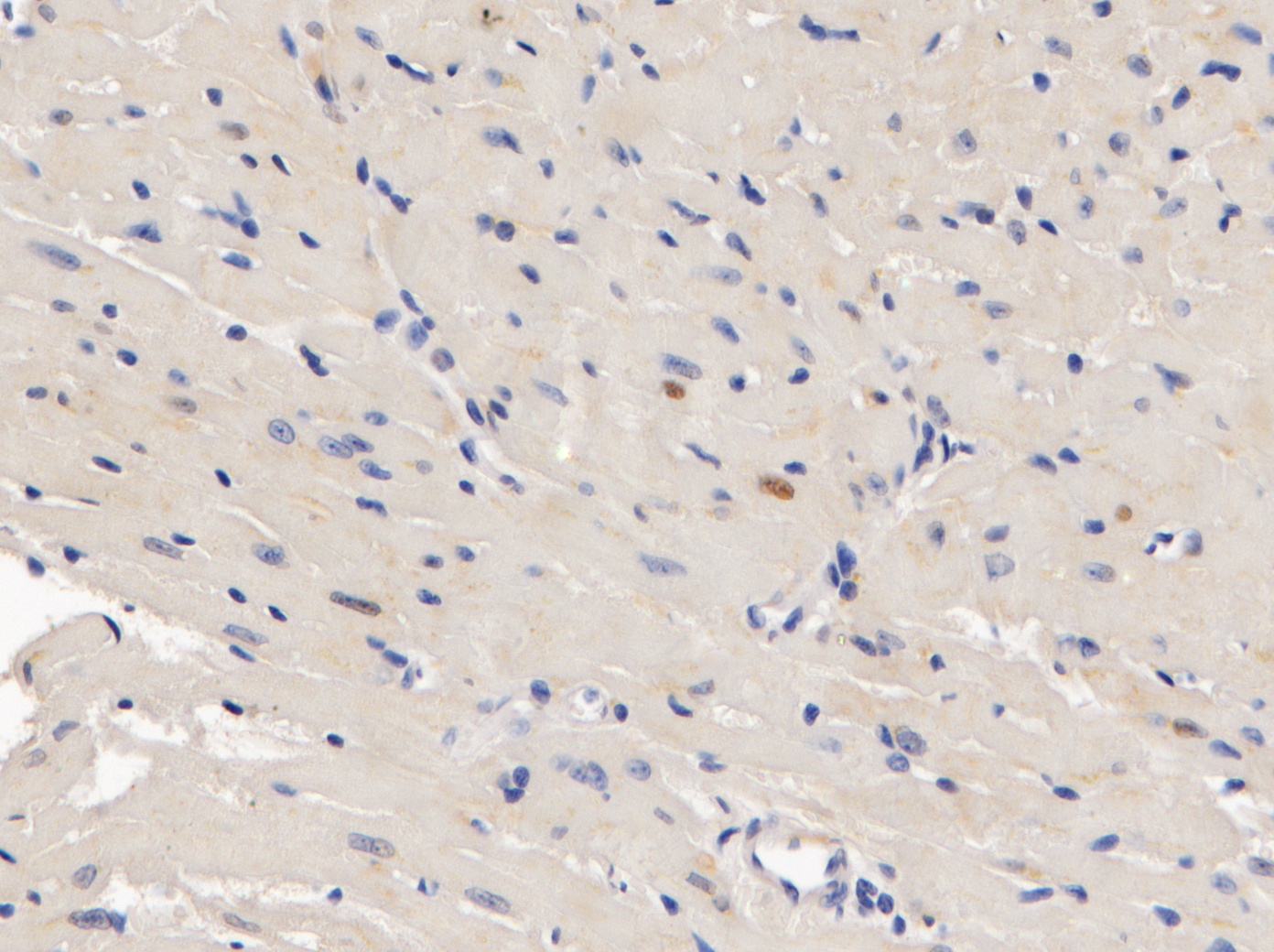

Supplement: Figure 4—source data 2. [file elife-55513-fig4-data2.zip › p19_images_for_eLife/p19_images_Ann_Chiao_for_eLife/Old SS-31 treated/OSS_1/Copy of MS1_2_40x_RGB.jpg]

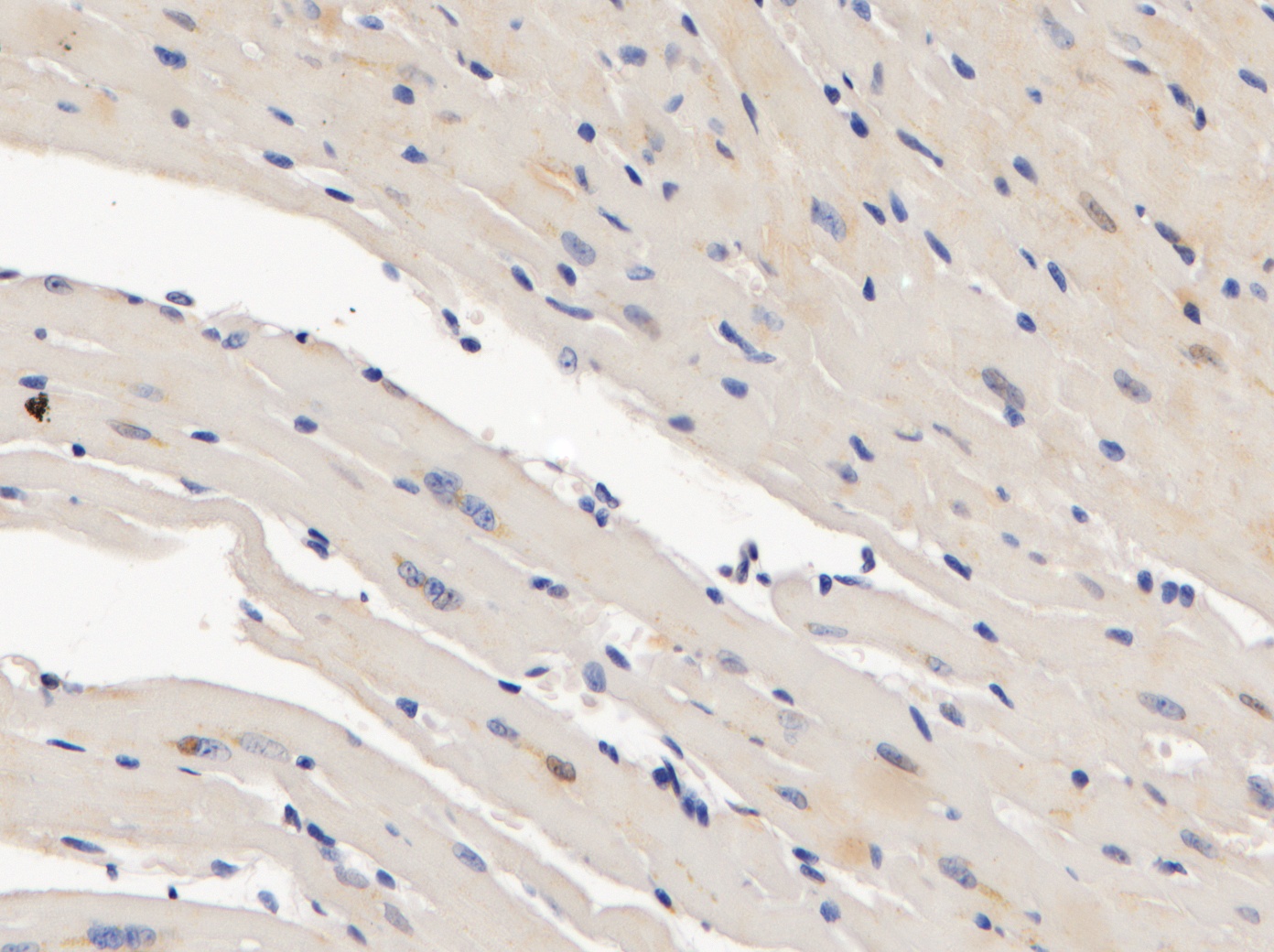

Supplement: Figure 4—source data 2. [file elife-55513-fig4-data2.zip › p19_images_for_eLife/p19_images_Ann_Chiao_for_eLife/Old SS-31 treated/OSS_1/Copy of MS1_3_40x_RGB.jpg]

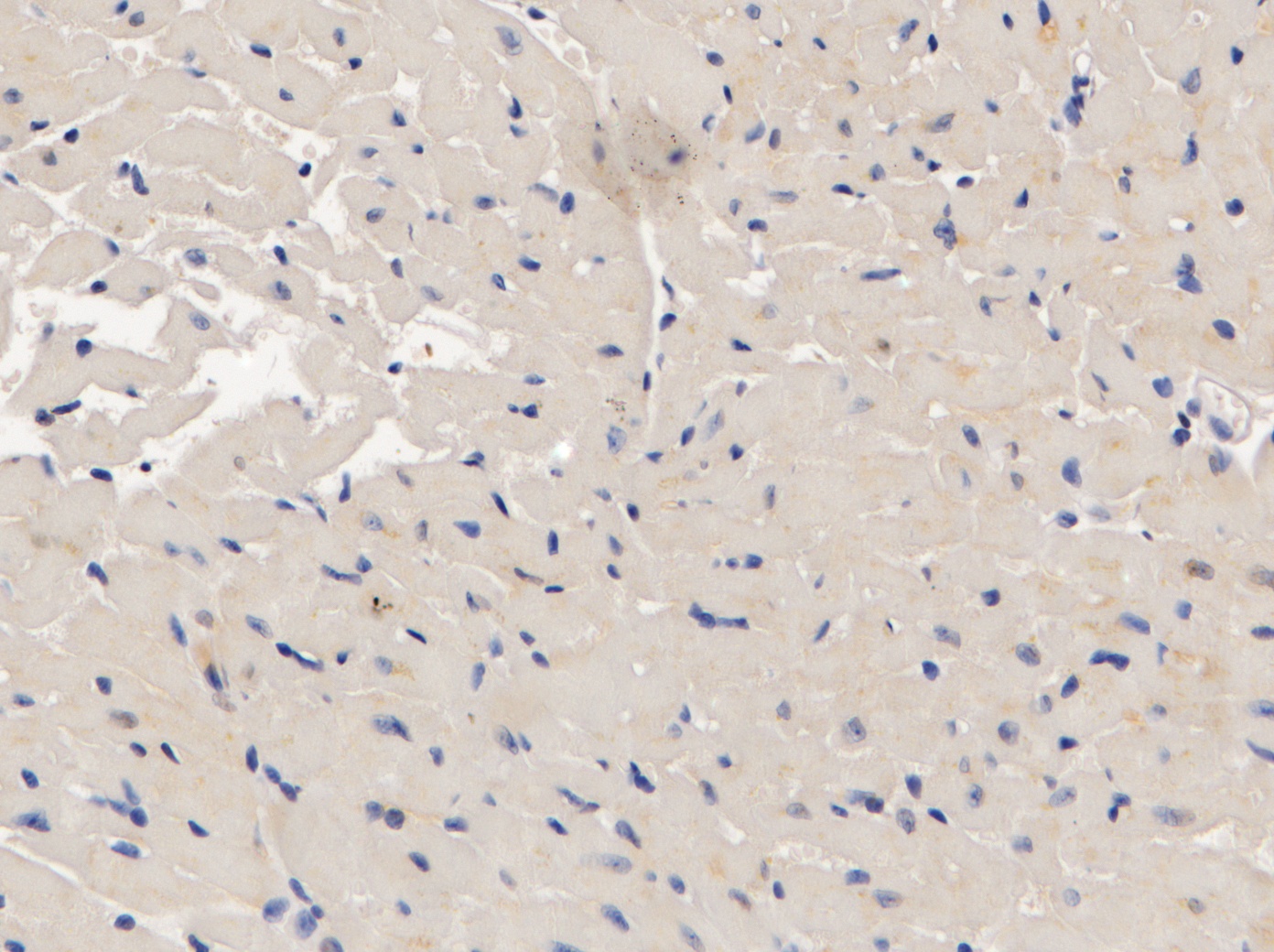

Supplement: Figure 4—source data 2. [file elife-55513-fig4-data2.zip › p19_images_for_eLife/p19_images_Ann_Chiao_for_eLife/Old SS-31 treated/OSS_1/Copy of MS1_4_40x_RGB.jpg]

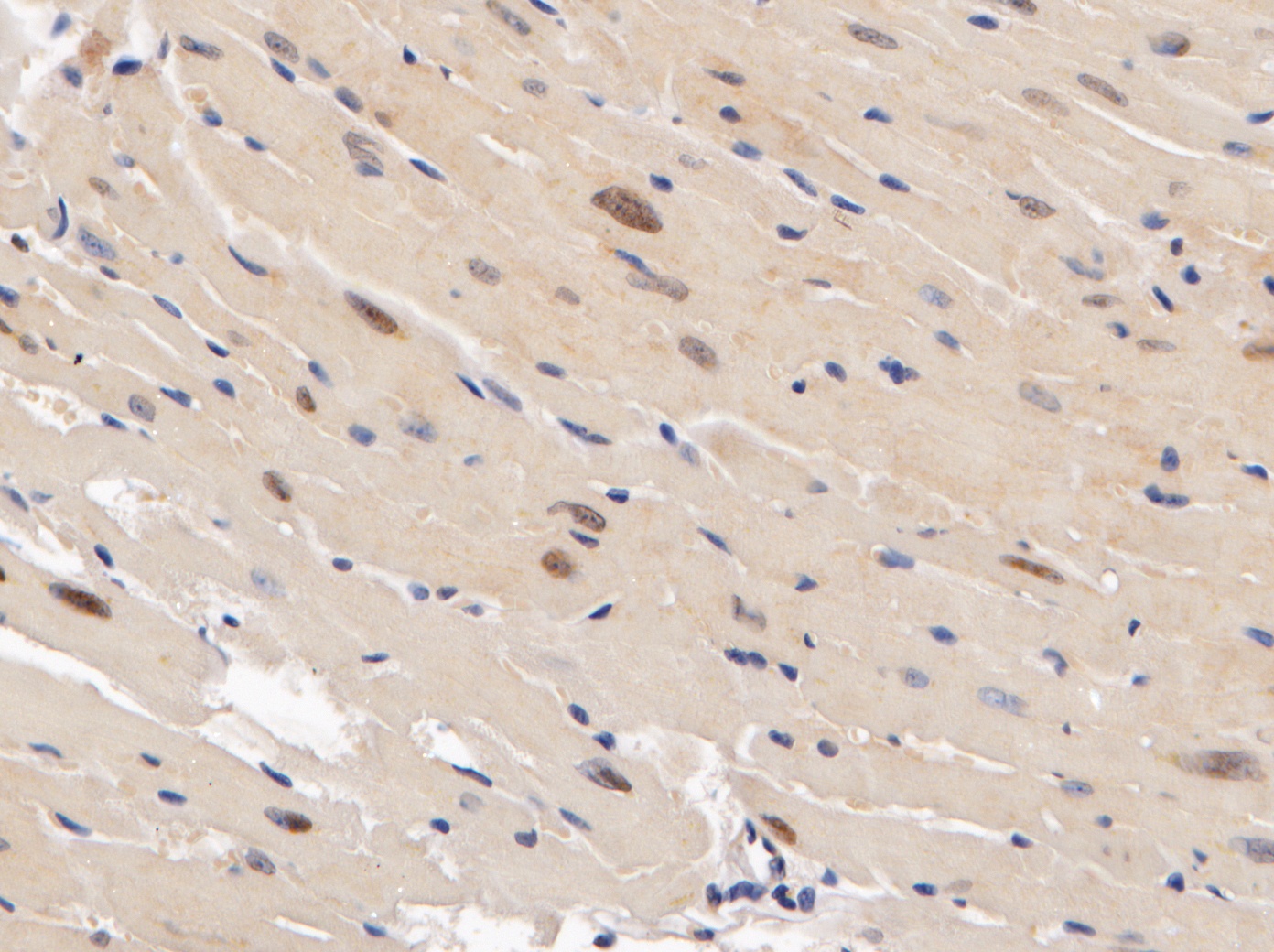

Supplement: Figure 4—source data 2. [file elife-55513-fig4-data2.zip › p19_images_for_eLife/p19_images_Ann_Chiao_for_eLife/Old SS-31 treated/OSS_2/Copy of MS3_1_40x_RGB.jpg]

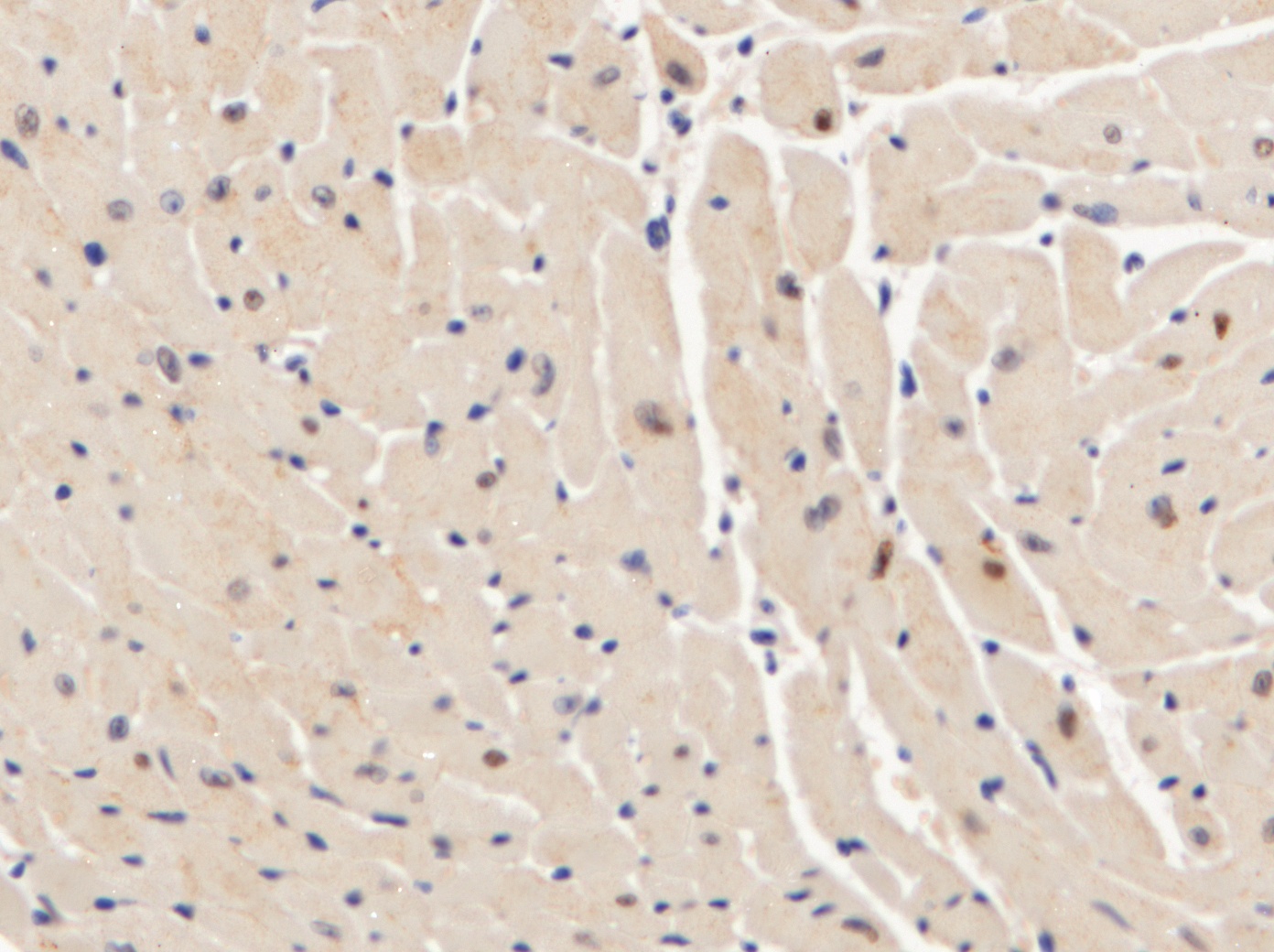

Supplement: Figure 4—source data 2. [file elife-55513-fig4-data2.zip › p19_images_for_eLife/p19_images_Ann_Chiao_for_eLife/Old SS-31 treated/OSS_2/Copy of MS3_2_40x_RGB.jpg]

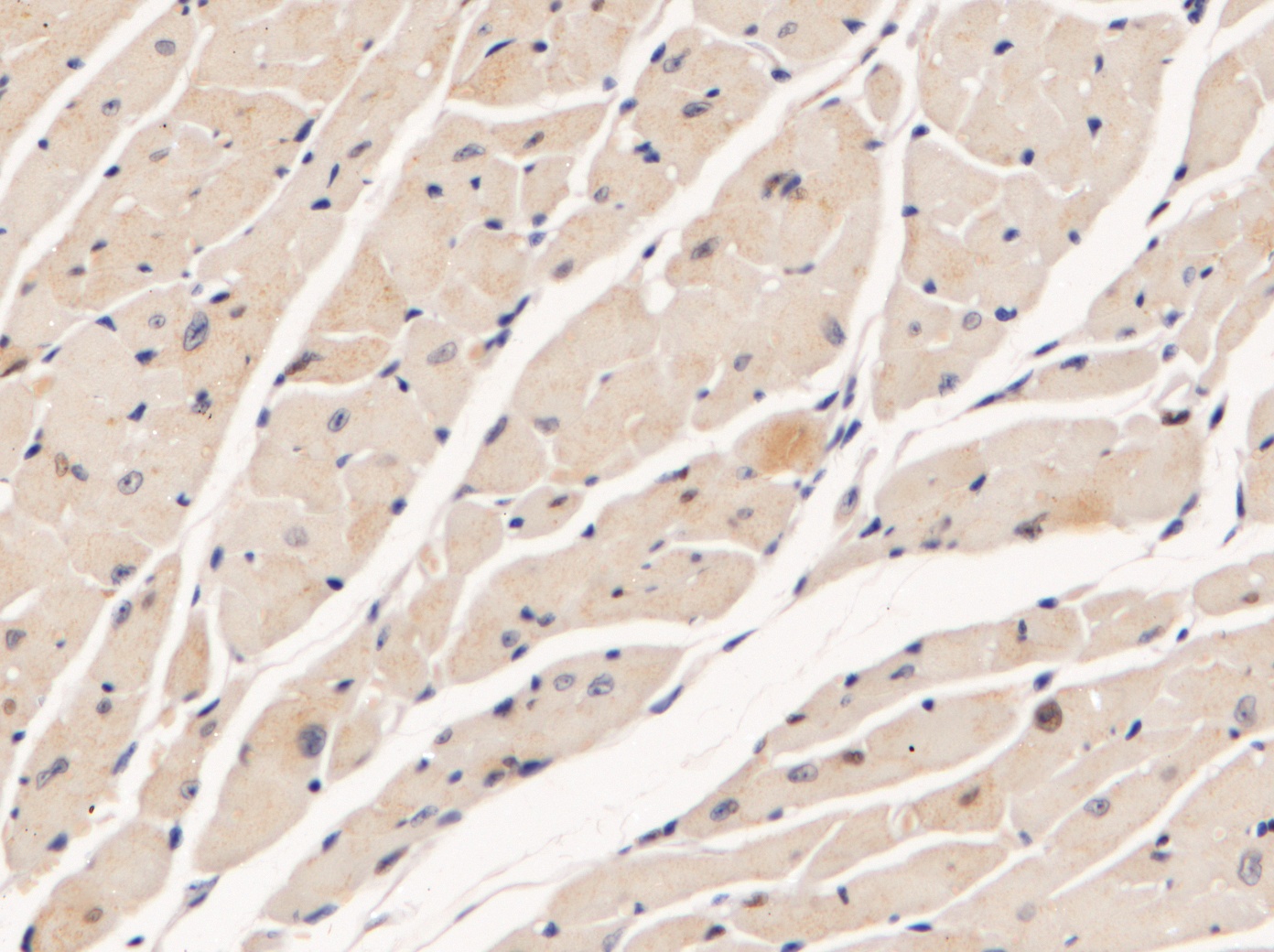

Supplement: Figure 4—source data 2. [file elife-55513-fig4-data2.zip › p19_images_for_eLife/p19_images_Ann_Chiao_for_eLife/Old SS-31 treated/OSS_2/Copy of MS3_3_40x_RGB.jpg]

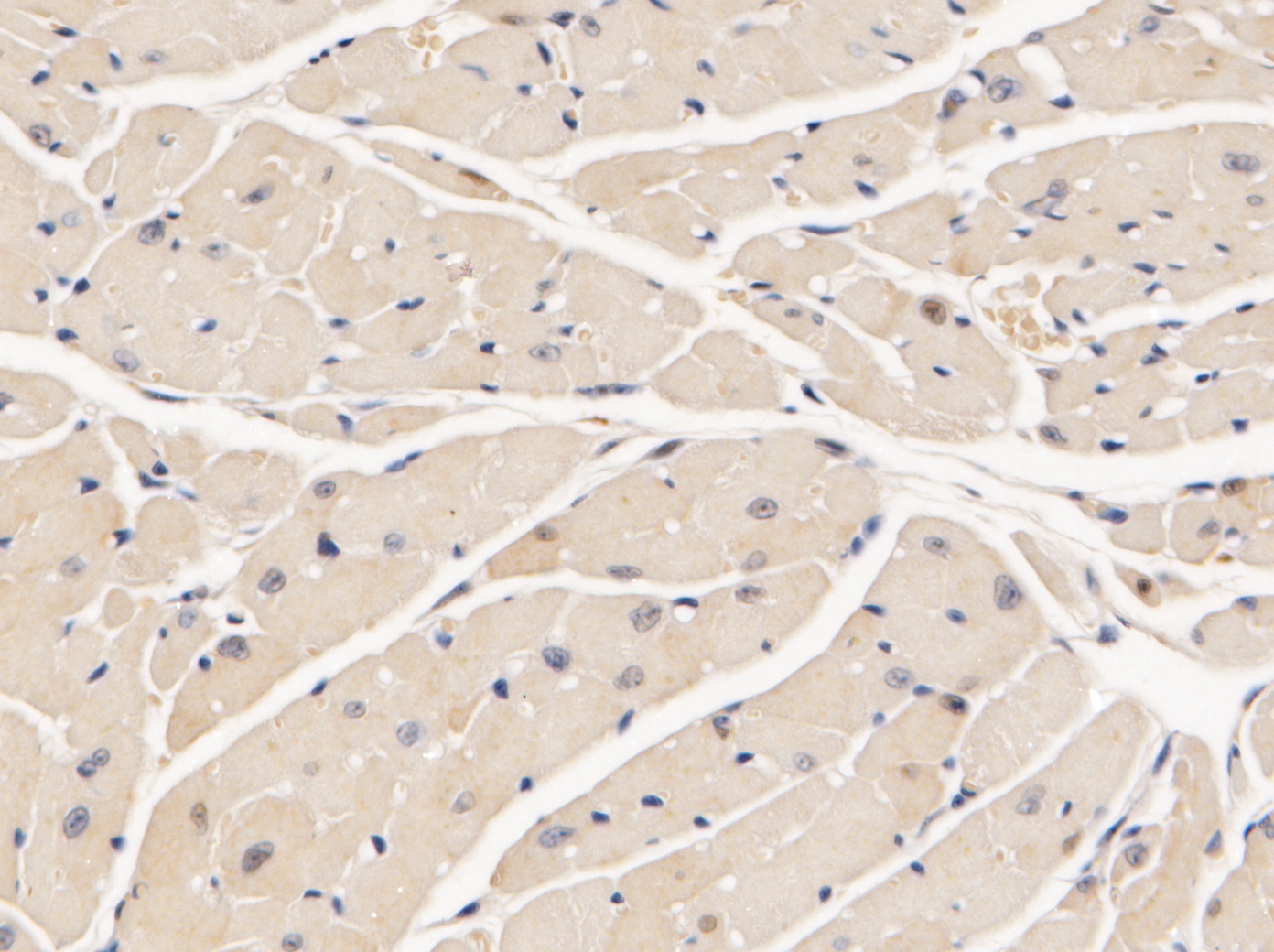

Supplement: Figure 4—source data 2. [file elife-55513-fig4-data2.zip › p19_images_for_eLife/p19_images_Ann_Chiao_for_eLife/Old SS-31 treated/OSS_2/Copy of MS3_4_40x_RGB.jpg]

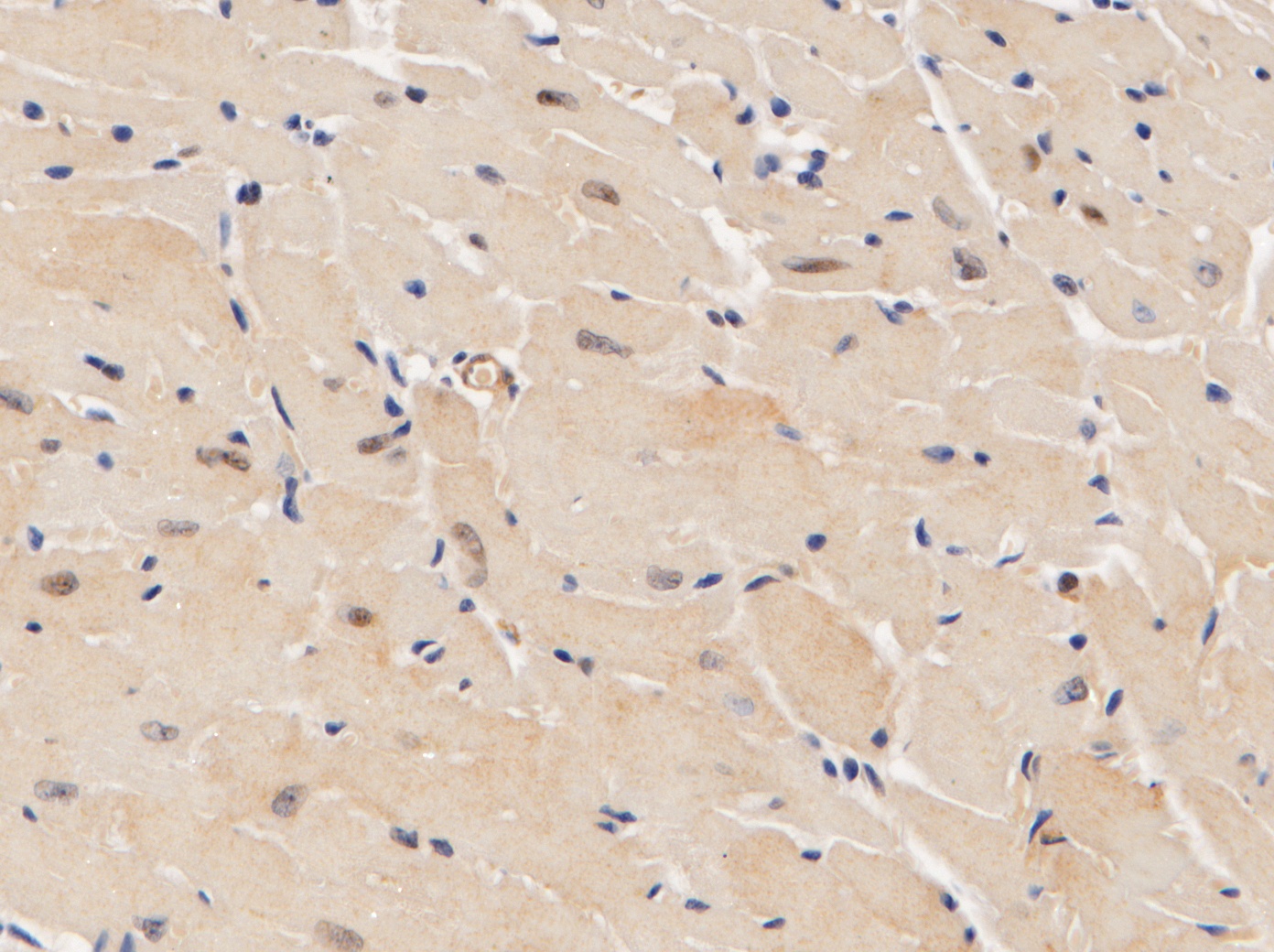

Supplement: Figure 4—source data 2. [file elife-55513-fig4-data2.zip › p19_images_for_eLife/p19_images_Ann_Chiao_for_eLife/Old SS-31 treated/OSS_2/Copy of MS3_6_40x_RGB.jpg]

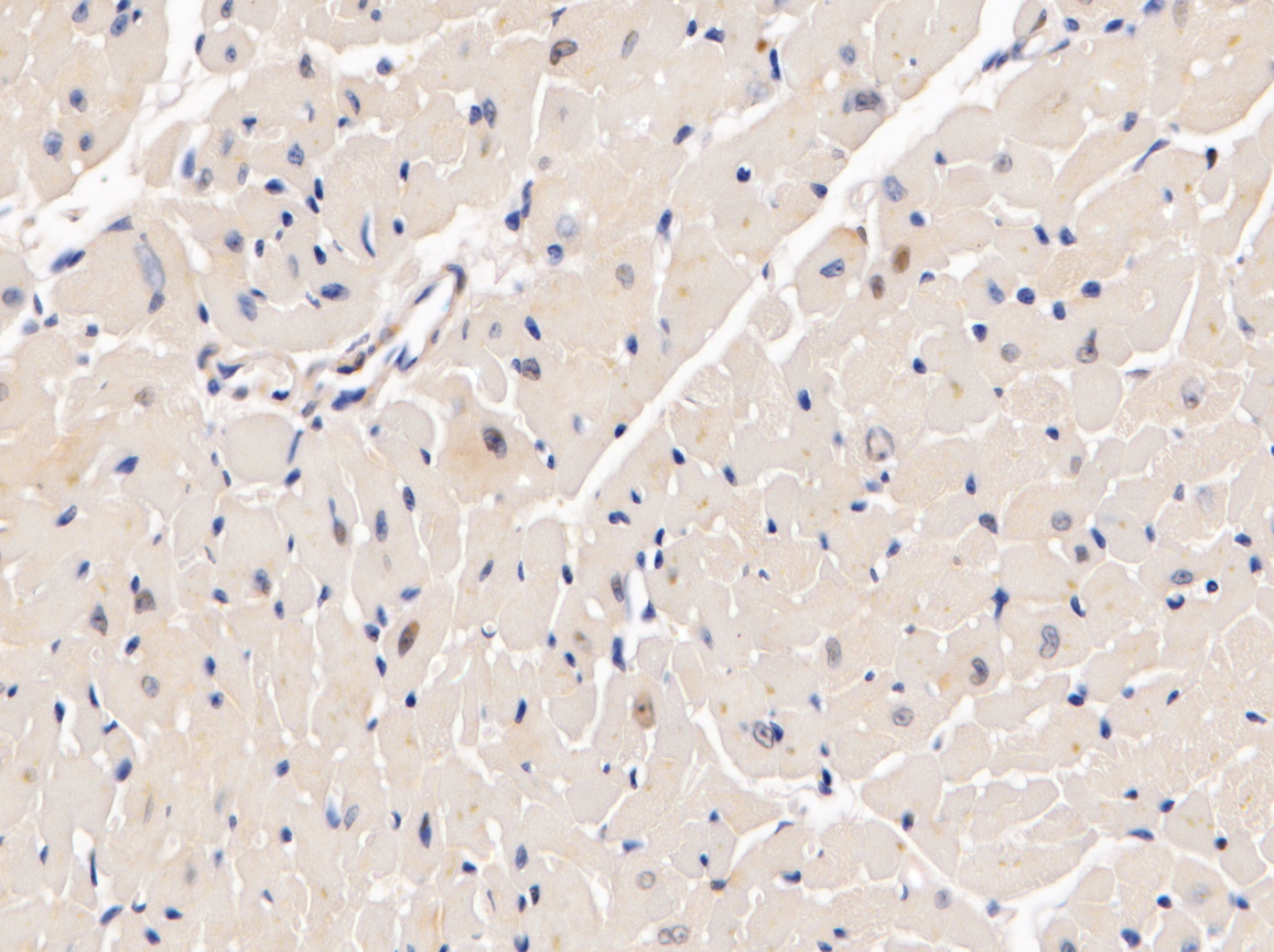

Supplement: Figure 4—source data 2. [file elife-55513-fig4-data2.zip › p19_images_for_eLife/p19_images_Ann_Chiao_for_eLife/Old SS-31 treated/OSS_3/Copy of MS6_1_40x_RGB.jpg]
