# Supplementary figures and images for: Late-life restoration of mitochondrial function reverses cardiac dysfunction in old mice (part 1 of 3)
Source: eLife. 2020 Jul 10;9:e55513. doi: 10.7554/eLife.55513 (PMC7377906; doi:10.7554/eLife.55513)

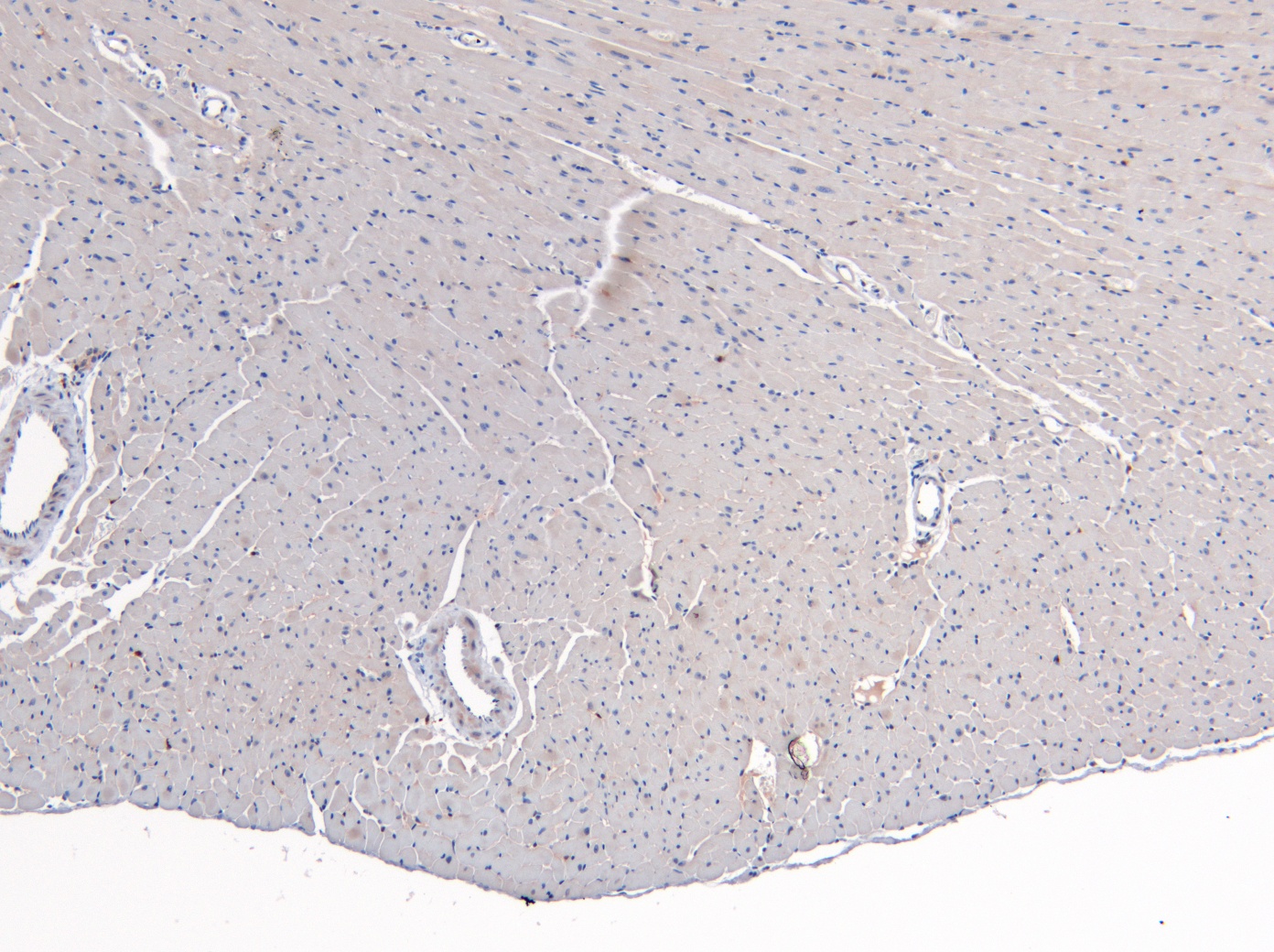

Supplement: Figure 4—source data 1. [file elife-55513-fig4-data1.zip › p16_images_for_eLife/p16_images_Ann_Chiao_for_eLife/Old Controls/OCL_1/MS_2_p16_10x_a_RGB.jpg]

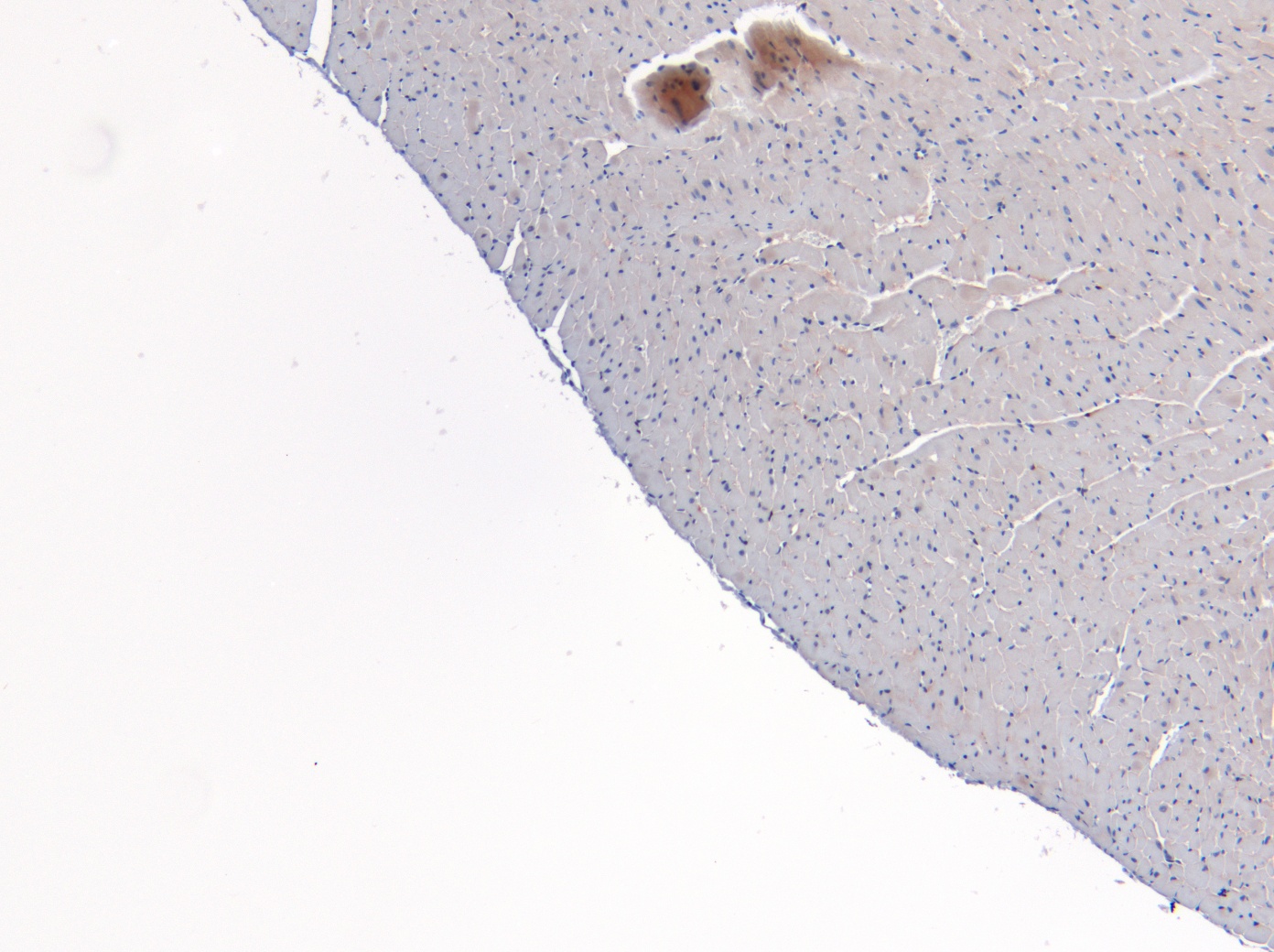

Supplement: Figure 4—source data 1. [file elife-55513-fig4-data1.zip › p16_images_for_eLife/p16_images_Ann_Chiao_for_eLife/Old Controls/OCL_1/MS_2_p16_10x_b_RGB.jpg]

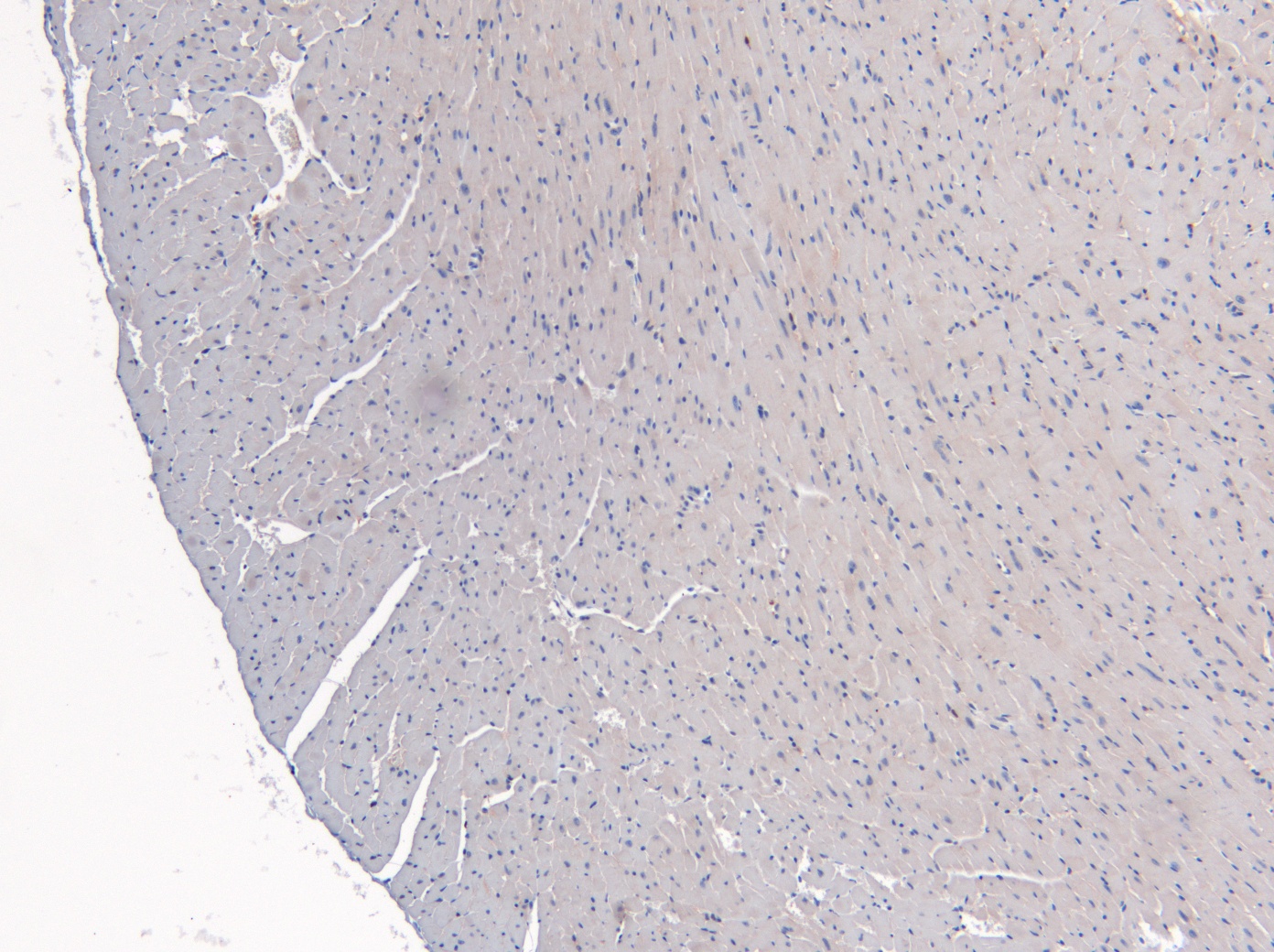

Supplement: Figure 4—source data 1. [file elife-55513-fig4-data1.zip › p16_images_for_eLife/p16_images_Ann_Chiao_for_eLife/Old Controls/OCL_1/MS_2_p16_10x_c_RGB.jpg]

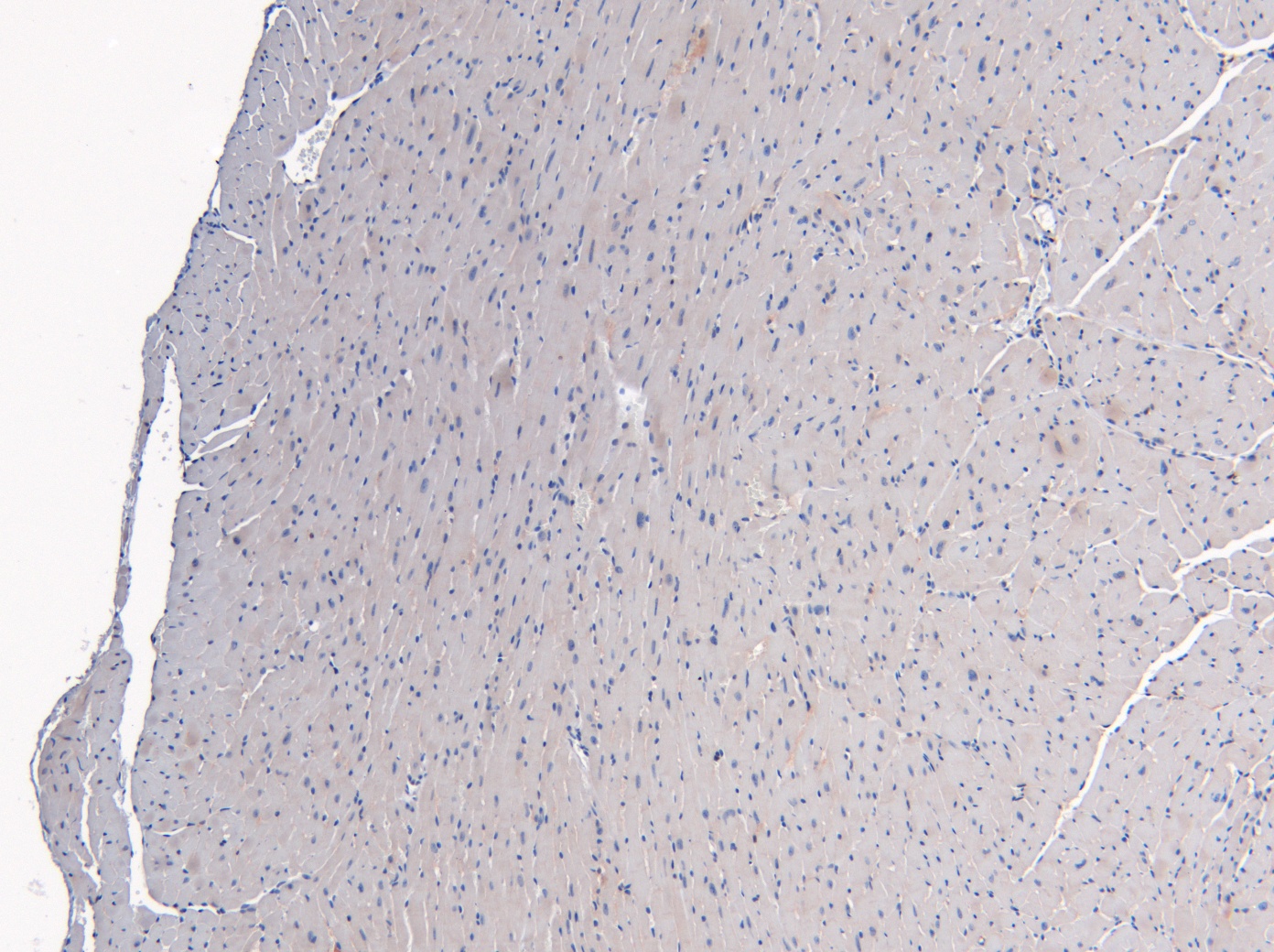

Supplement: Figure 4—source data 1. [file elife-55513-fig4-data1.zip › p16_images_for_eLife/p16_images_Ann_Chiao_for_eLife/Old Controls/OCL_1/MS_2_p16_10x_d_RGB.jpg]

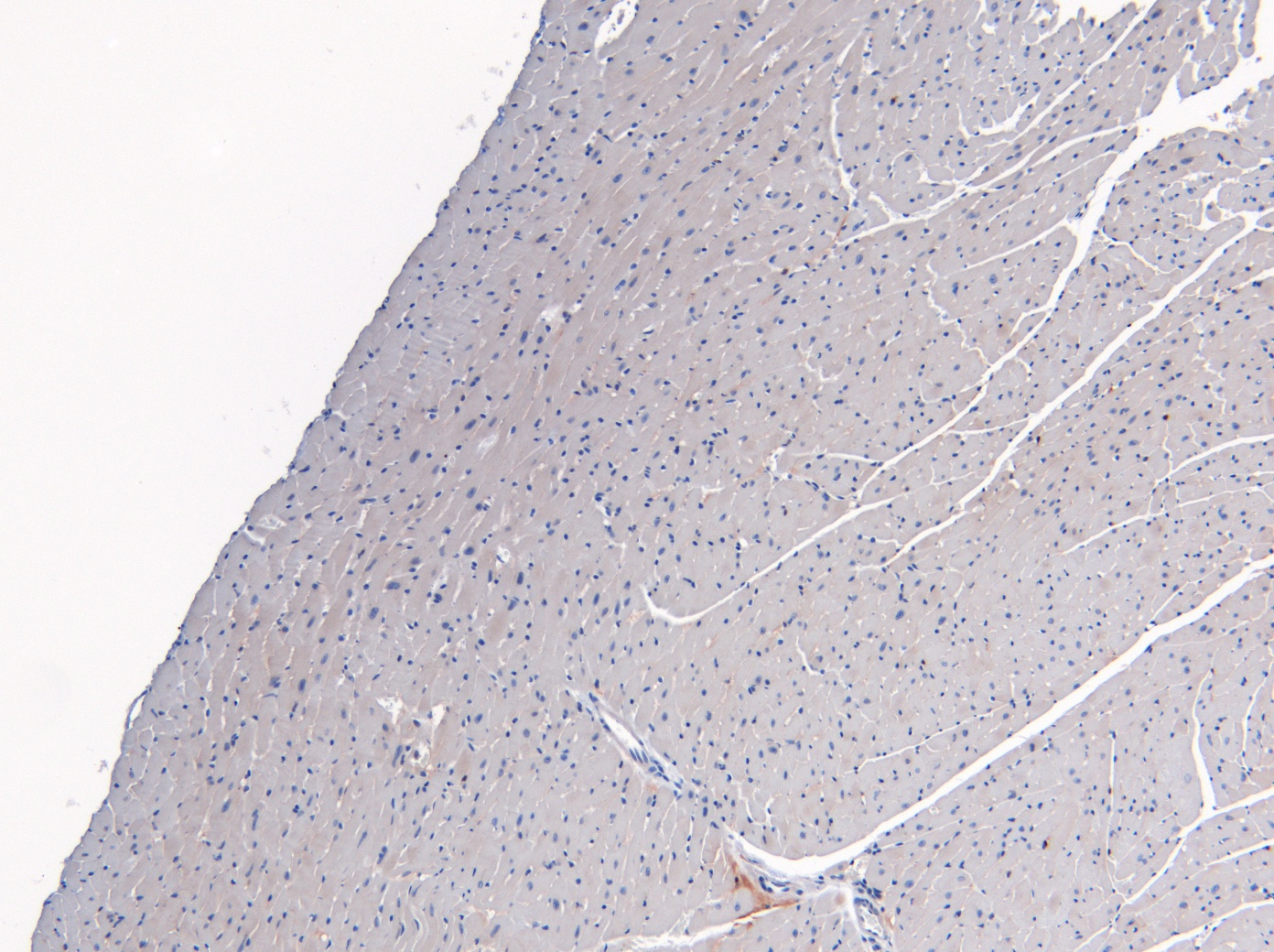

Supplement: Figure 4—source data 1. [file elife-55513-fig4-data1.zip › p16_images_for_eLife/p16_images_Ann_Chiao_for_eLife/Old Controls/OCL_1/MS_2_p16_10x_e_RGB.jpg]

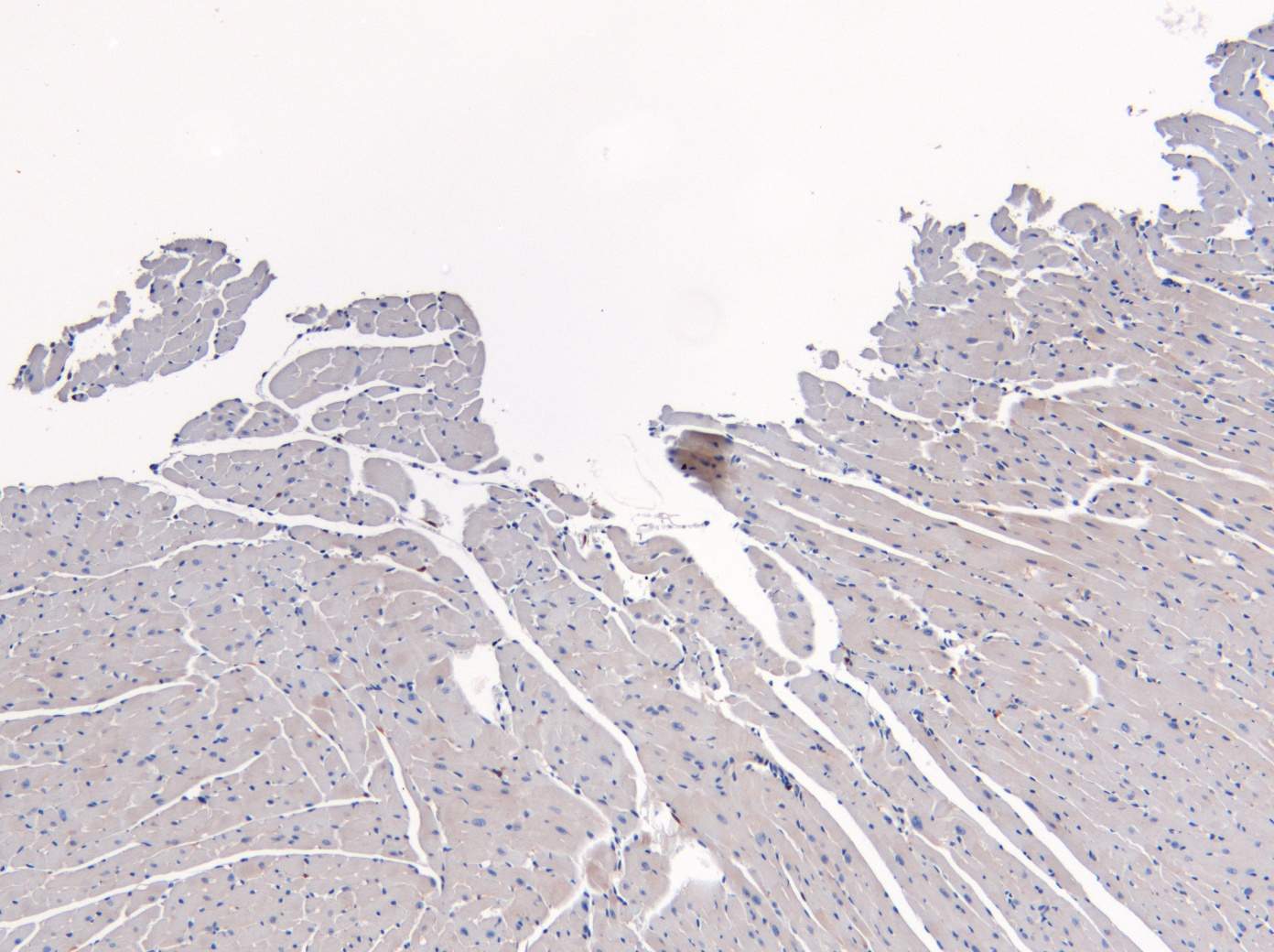

Supplement: Figure 4—source data 1. [file elife-55513-fig4-data1.zip › p16_images_for_eLife/p16_images_Ann_Chiao_for_eLife/Old Controls/OCL_1/MS_2_p16_10x_f_RGB.jpg]

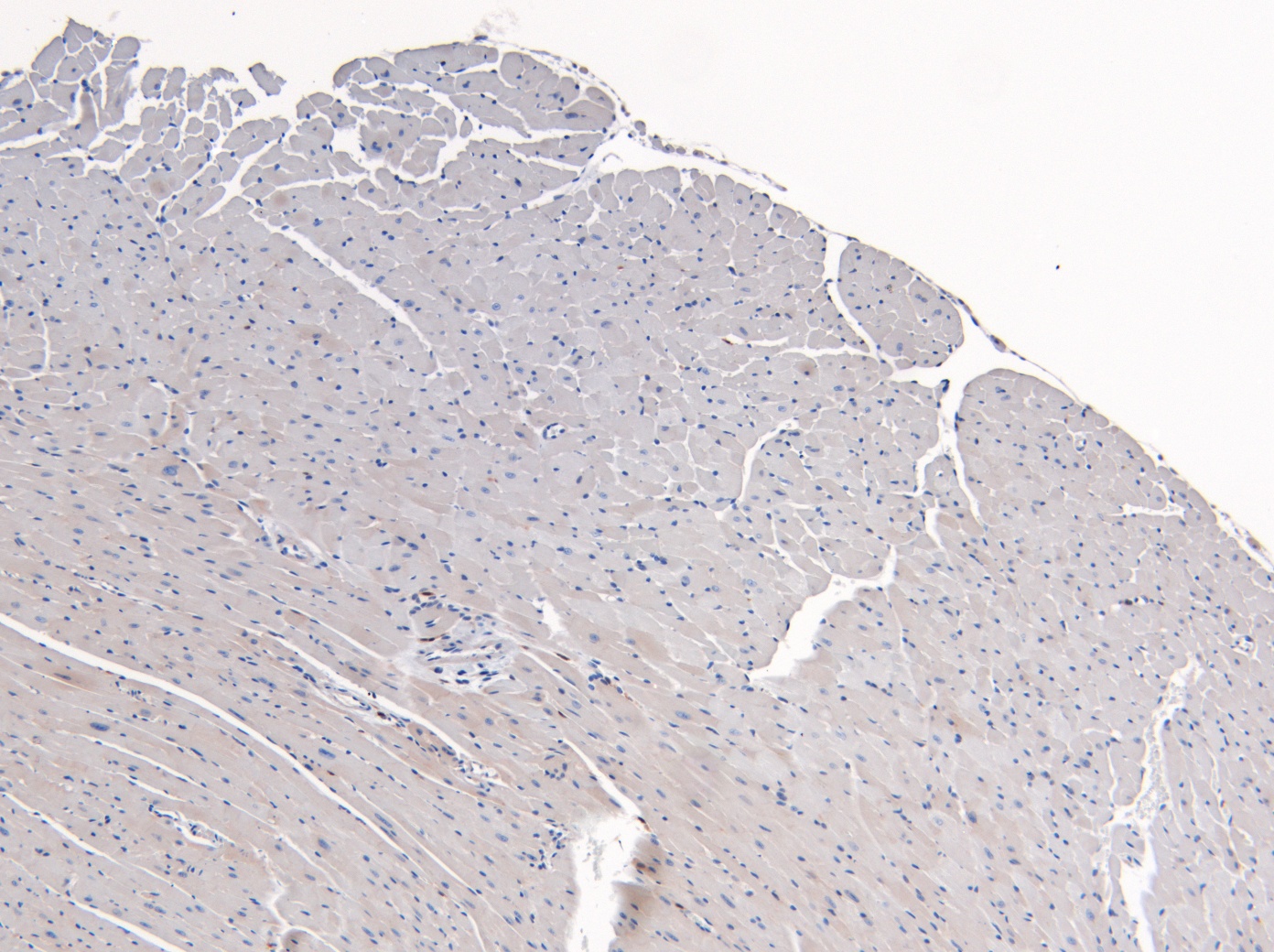

Supplement: Figure 4—source data 1. [file elife-55513-fig4-data1.zip › p16_images_for_eLife/p16_images_Ann_Chiao_for_eLife/Old Controls/OCL_1/MS_2_p16_10x_g_RGB.jpg]

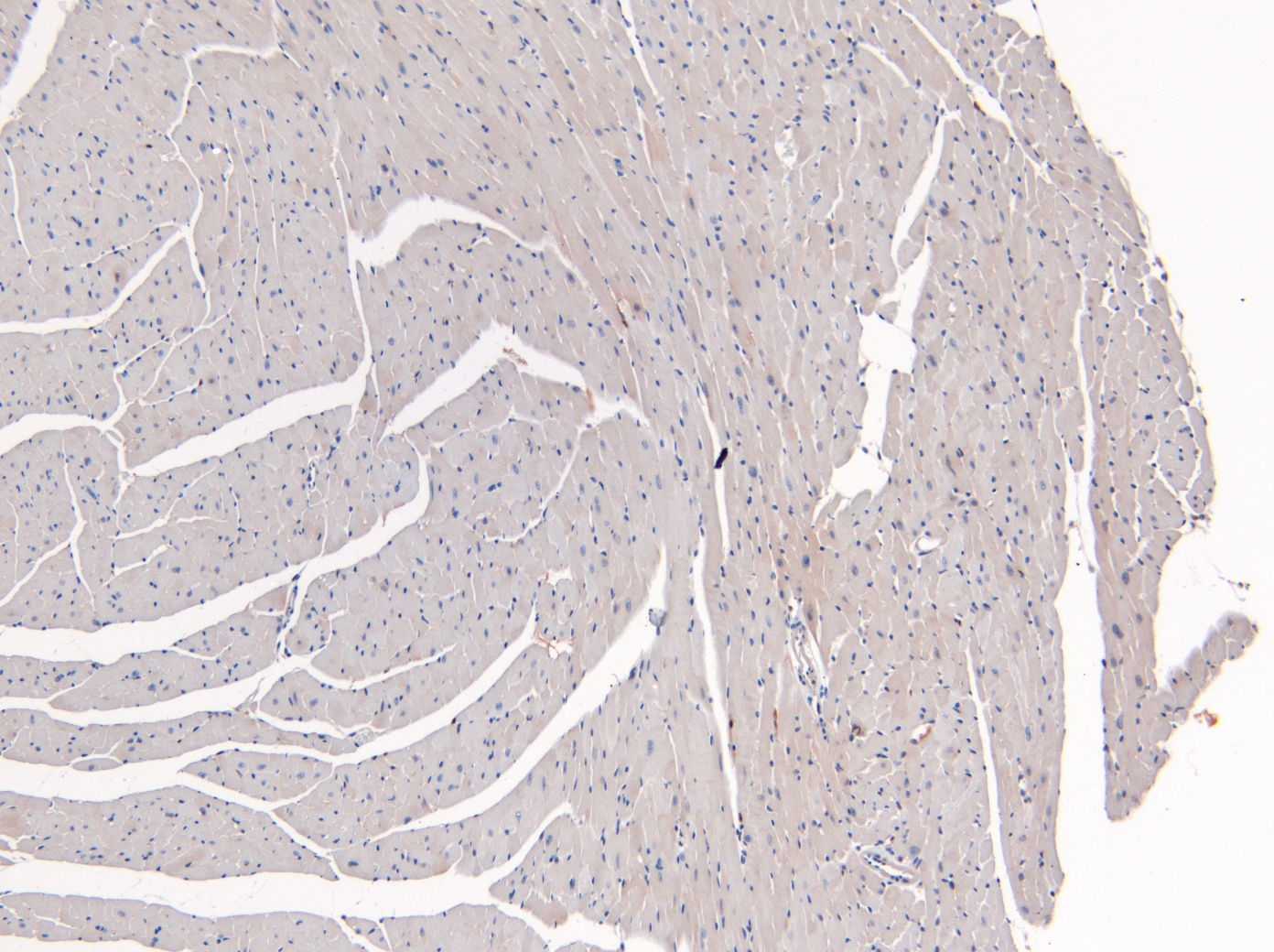

Supplement: Figure 4—source data 1. [file elife-55513-fig4-data1.zip › p16_images_for_eLife/p16_images_Ann_Chiao_for_eLife/Old Controls/OCL_1/MS_2_p16_10x_h_RGB.jpg]

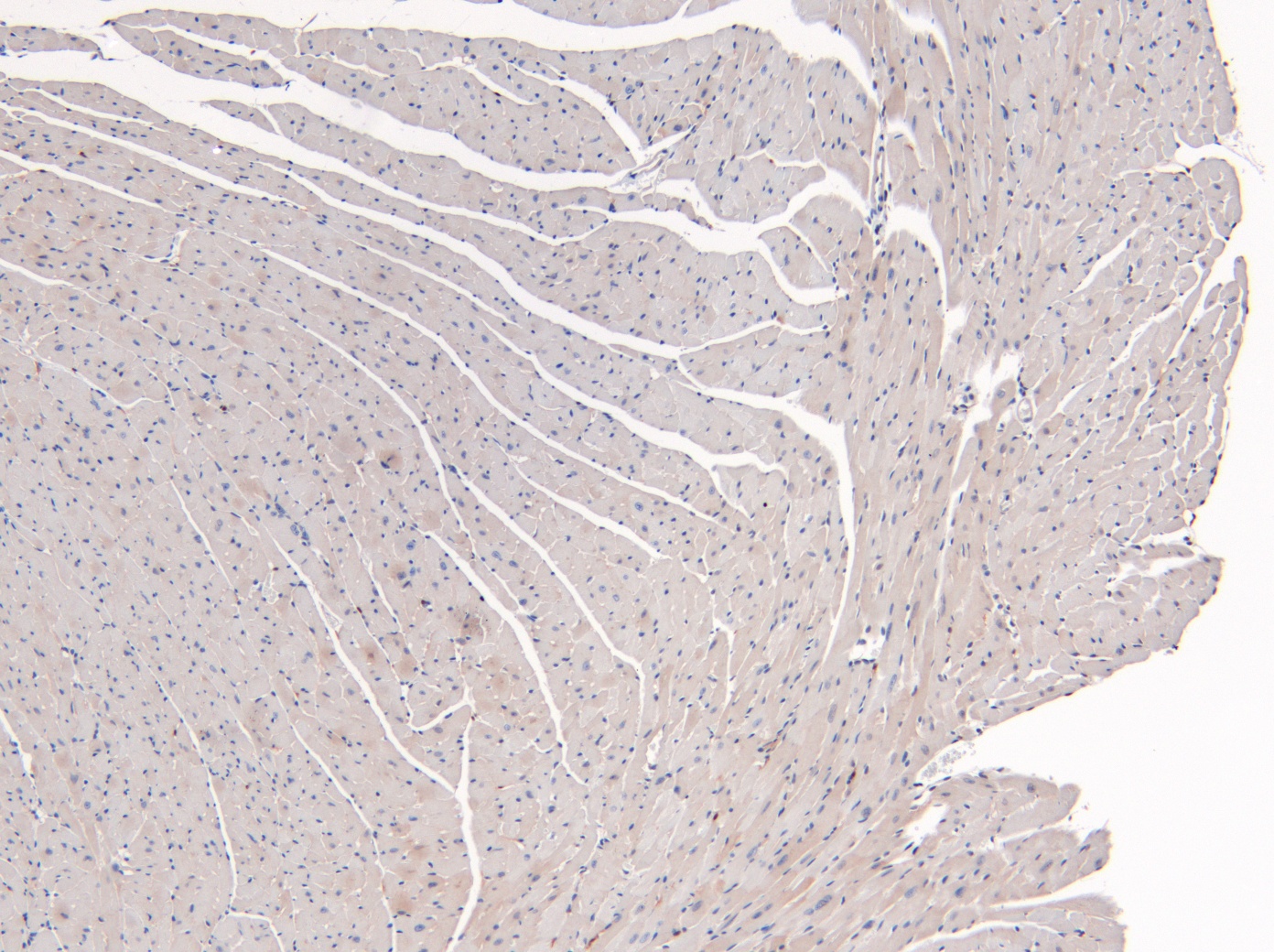

Supplement: Figure 4—source data 1. [file elife-55513-fig4-data1.zip › p16_images_for_eLife/p16_images_Ann_Chiao_for_eLife/Old Controls/OCL_1/MS_2_p16_10x_i_RGB.jpg]

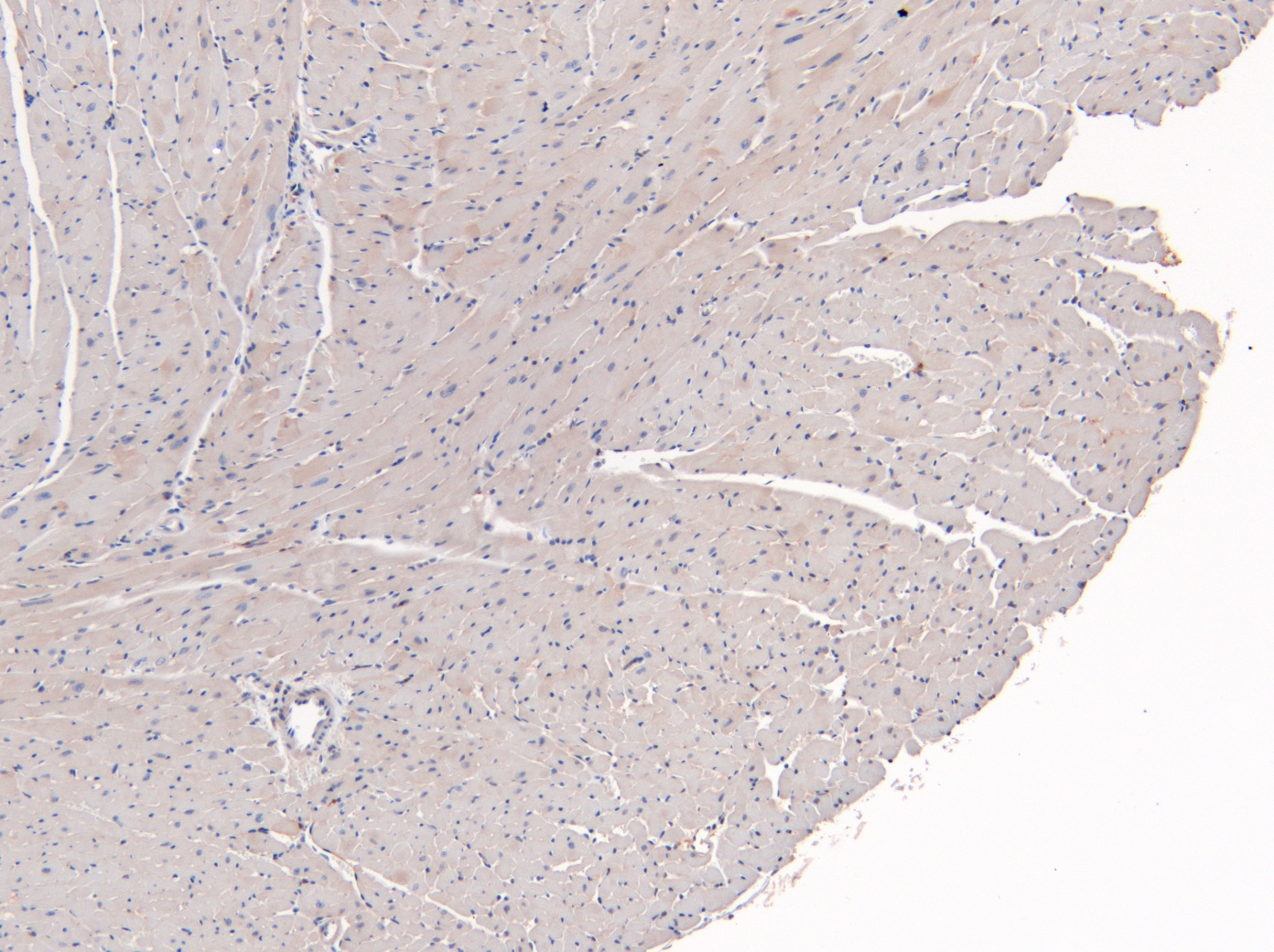

Supplement: Figure 4—source data 1. [file elife-55513-fig4-data1.zip › p16_images_for_eLife/p16_images_Ann_Chiao_for_eLife/Old Controls/OCL_1/MS_2_p16_10x_j_RGB.jpg]

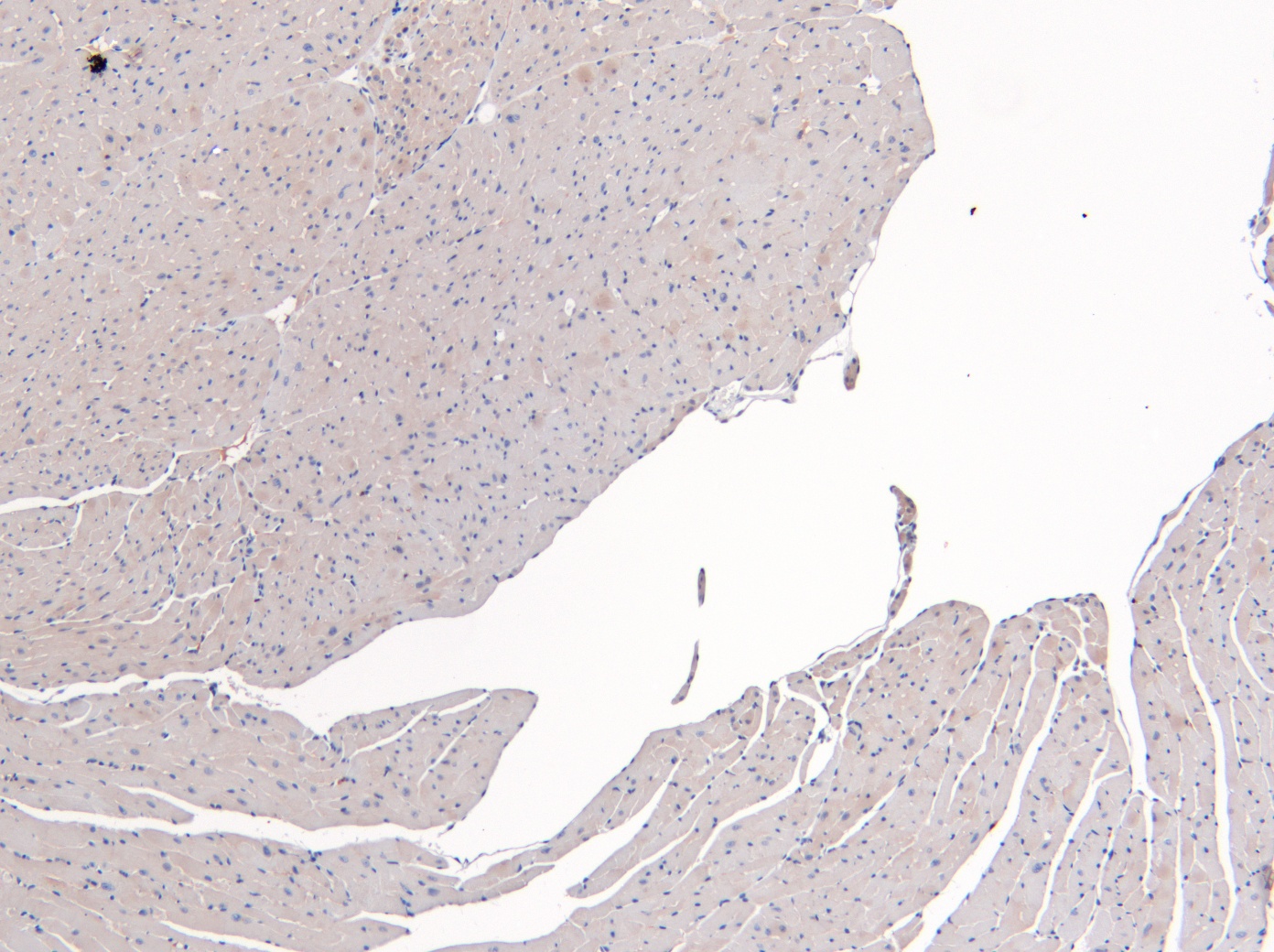

Supplement: Figure 4—source data 1. [file elife-55513-fig4-data1.zip › p16_images_for_eLife/p16_images_Ann_Chiao_for_eLife/Old Controls/OCL_1/MS_2_p16_10x_k_RGB.jpg]

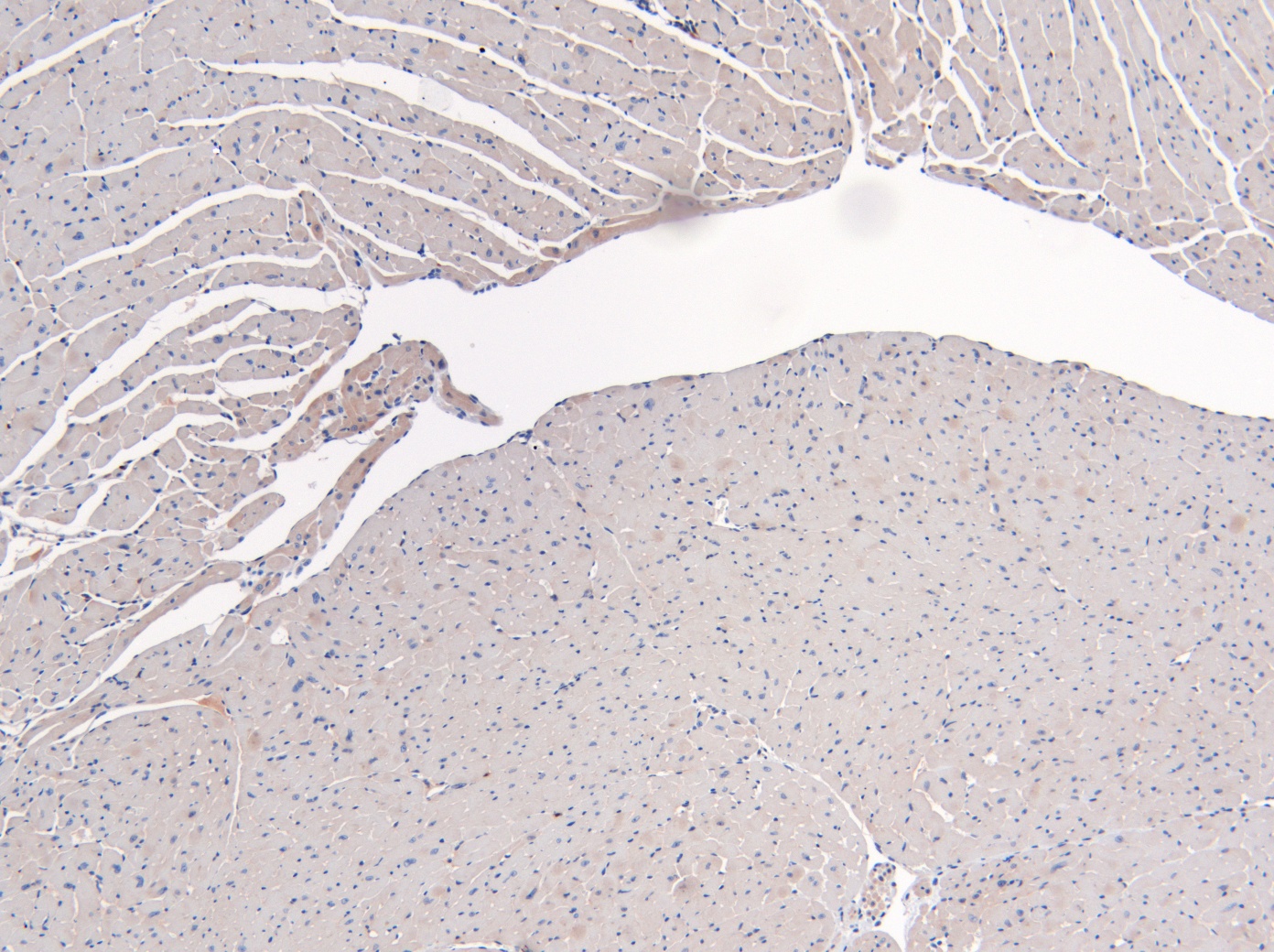

Supplement: Figure 4—source data 1. [file elife-55513-fig4-data1.zip › p16_images_for_eLife/p16_images_Ann_Chiao_for_eLife/Old Controls/OCL_1/MS_2_p16_10x_l_RGB.jpg]

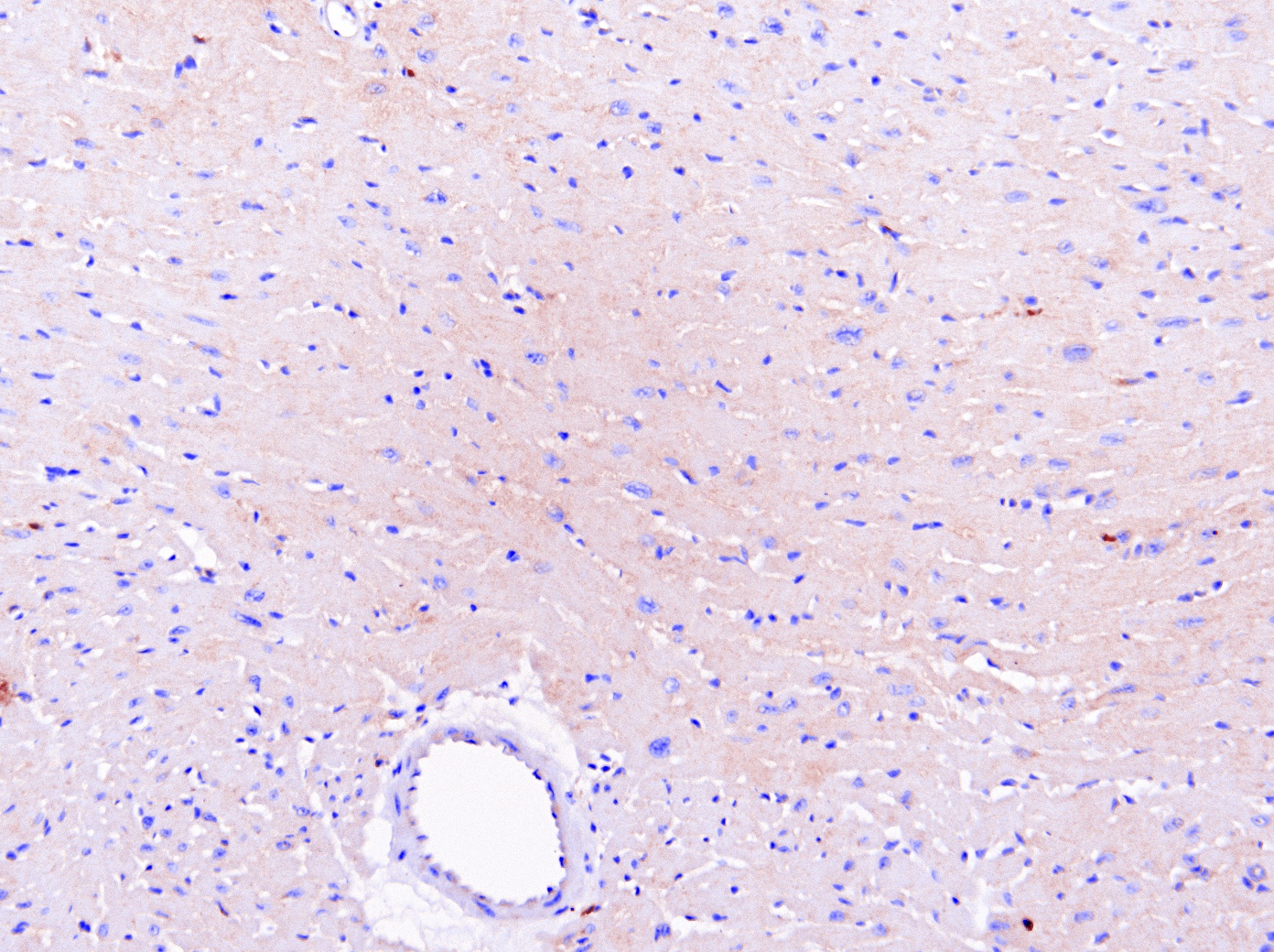

Supplement: Figure 4—source data 1. [file elife-55513-fig4-data1.zip › p16_images_for_eLife/p16_images_Ann_Chiao_for_eLife/Old Controls/OCL_10/Composite_9.1_a.jpg]

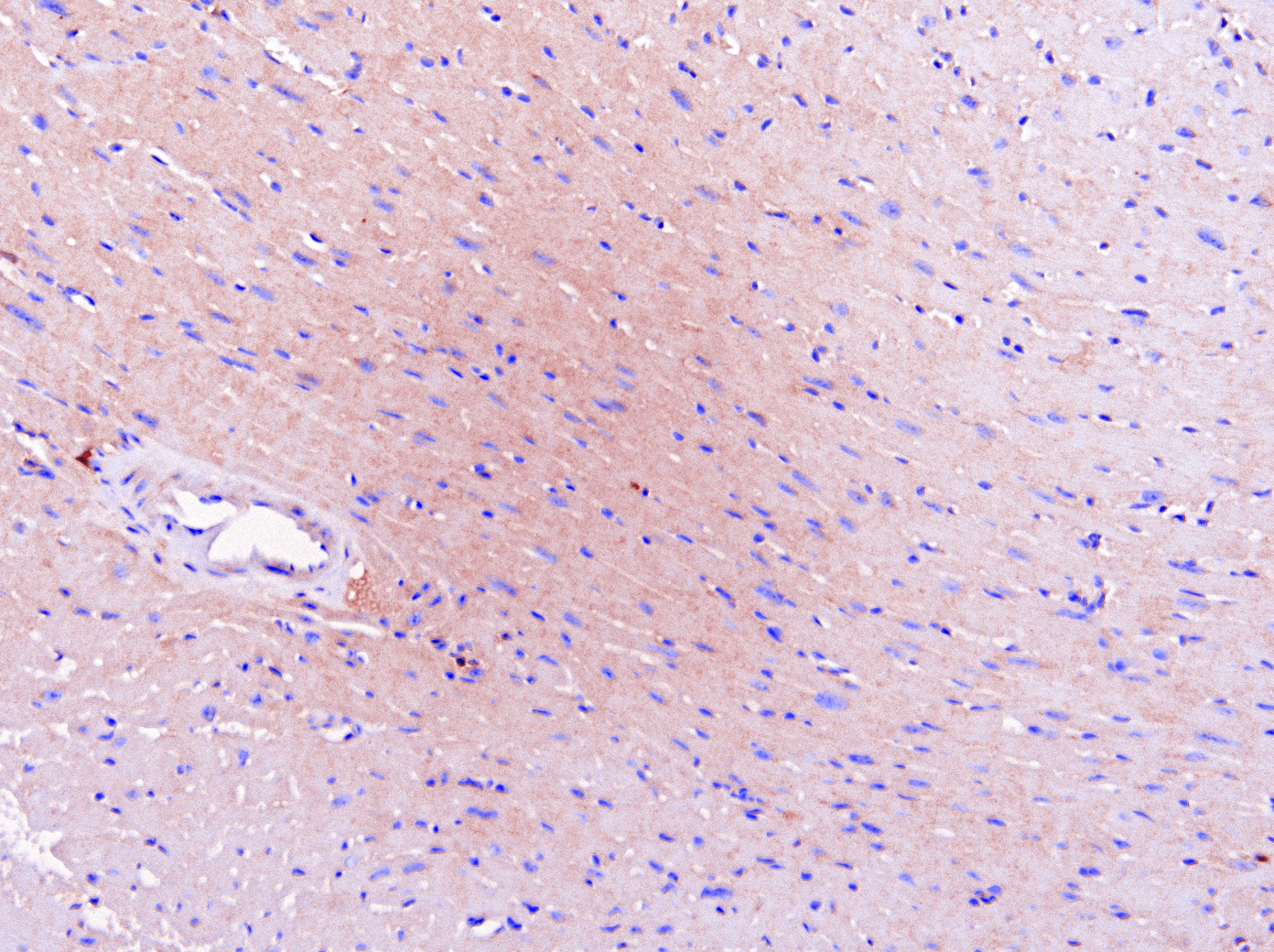

Supplement: Figure 4—source data 1. [file elife-55513-fig4-data1.zip › p16_images_for_eLife/p16_images_Ann_Chiao_for_eLife/Old Controls/OCL_10/Composite_9.1_b.jpg]

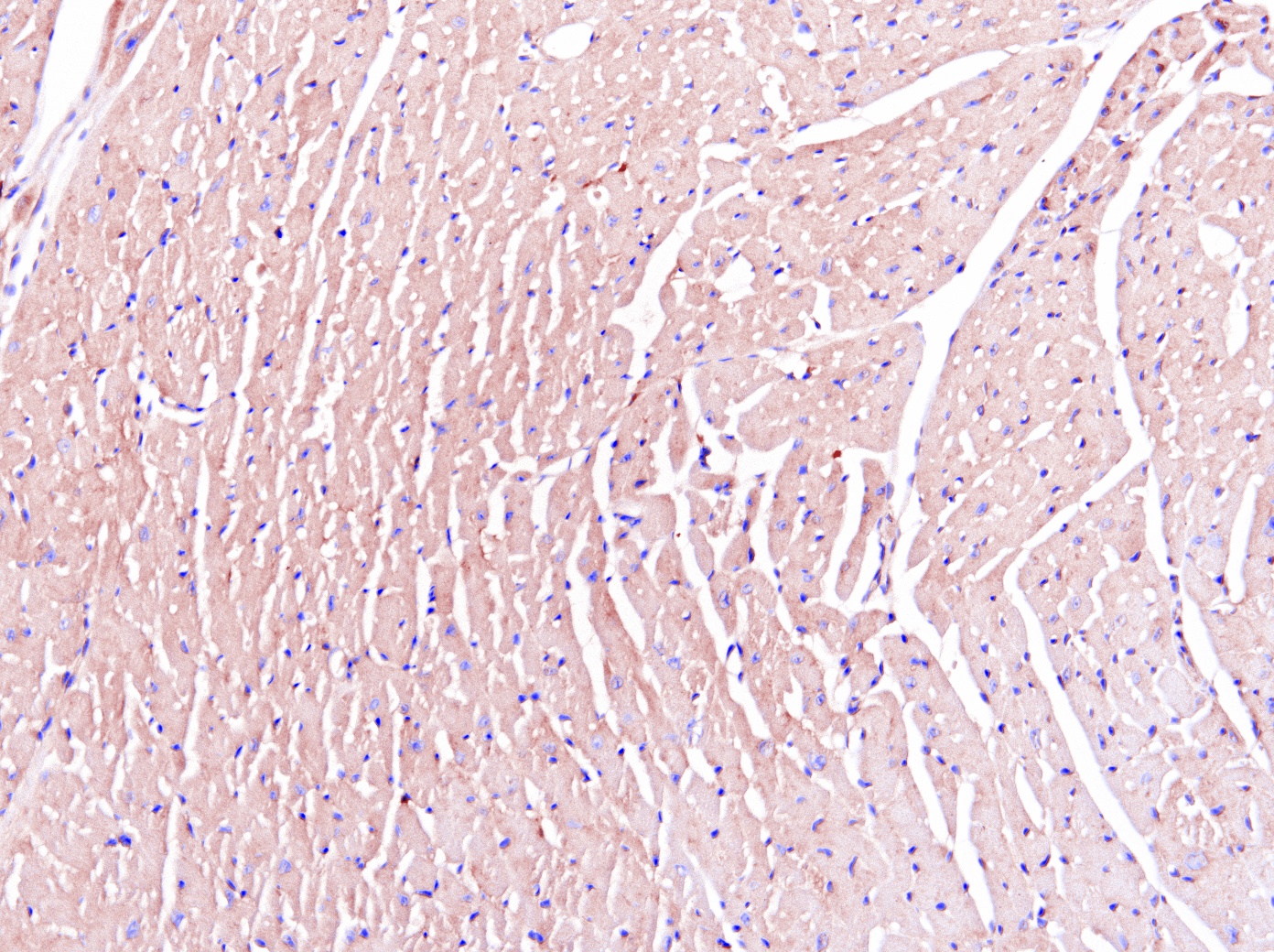

Supplement: Figure 4—source data 1. [file elife-55513-fig4-data1.zip › p16_images_for_eLife/p16_images_Ann_Chiao_for_eLife/Old Controls/OCL_10/Composite_9.1_c.jpg]

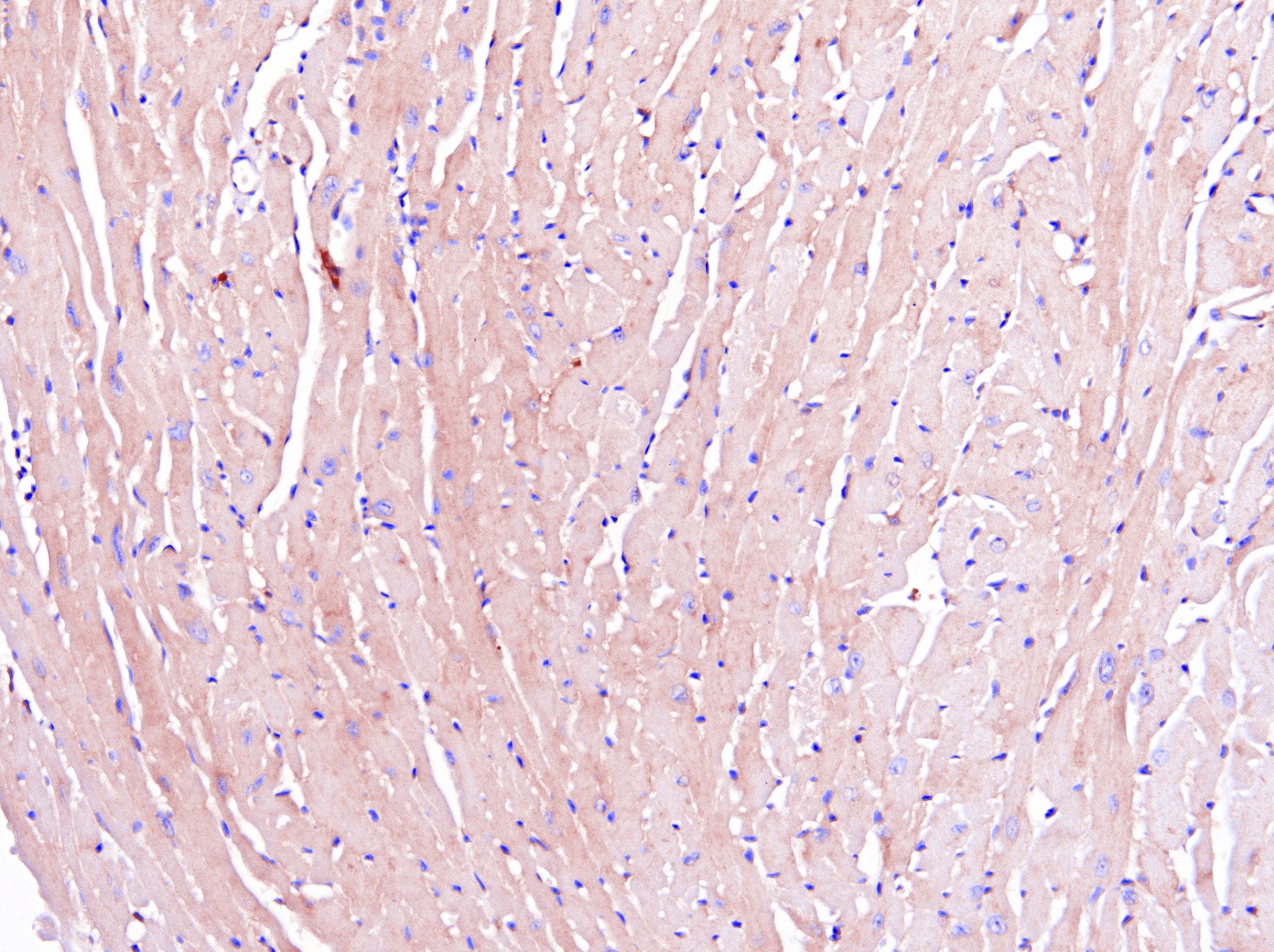

Supplement: Figure 4—source data 1. [file elife-55513-fig4-data1.zip › p16_images_for_eLife/p16_images_Ann_Chiao_for_eLife/Old Controls/OCL_10/Composite_9.1_d.jpg]

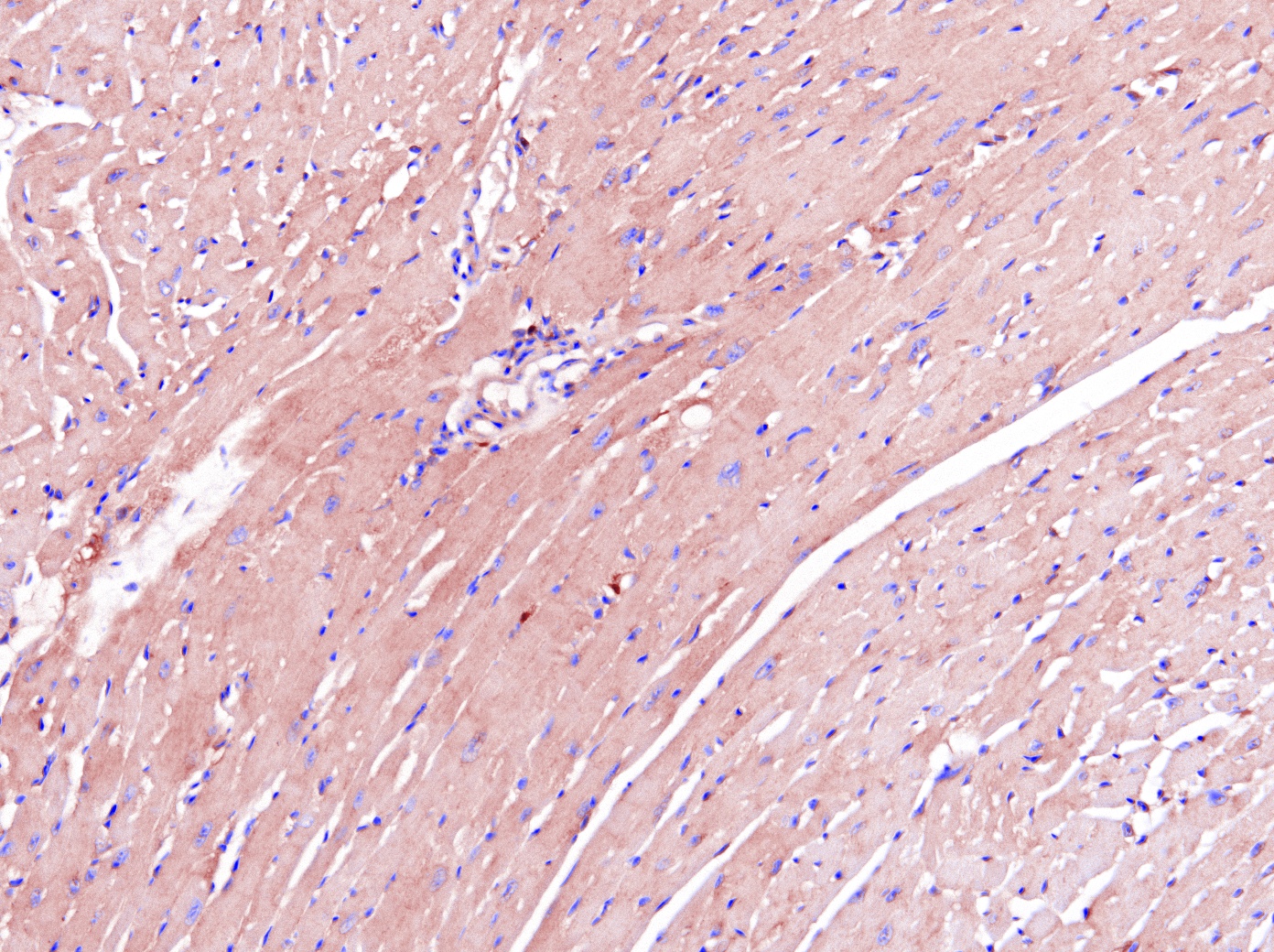

Supplement: Figure 4—source data 1. [file elife-55513-fig4-data1.zip › p16_images_for_eLife/p16_images_Ann_Chiao_for_eLife/Old Controls/OCL_10/Composite_9.1_e.jpg]

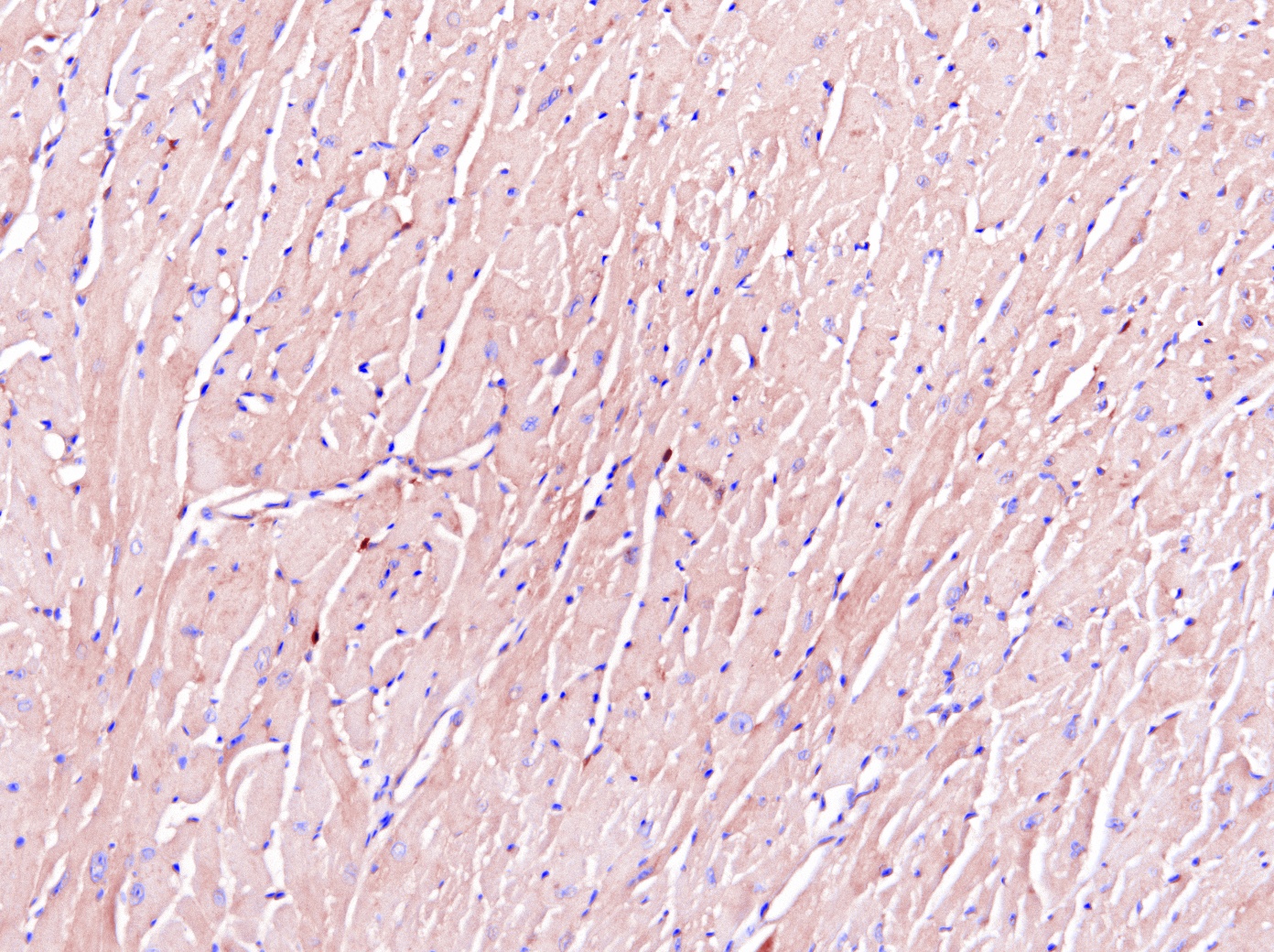

Supplement: Figure 4—source data 1. [file elife-55513-fig4-data1.zip › p16_images_for_eLife/p16_images_Ann_Chiao_for_eLife/Old Controls/OCL_10/Composite_9.1_f.jpg]

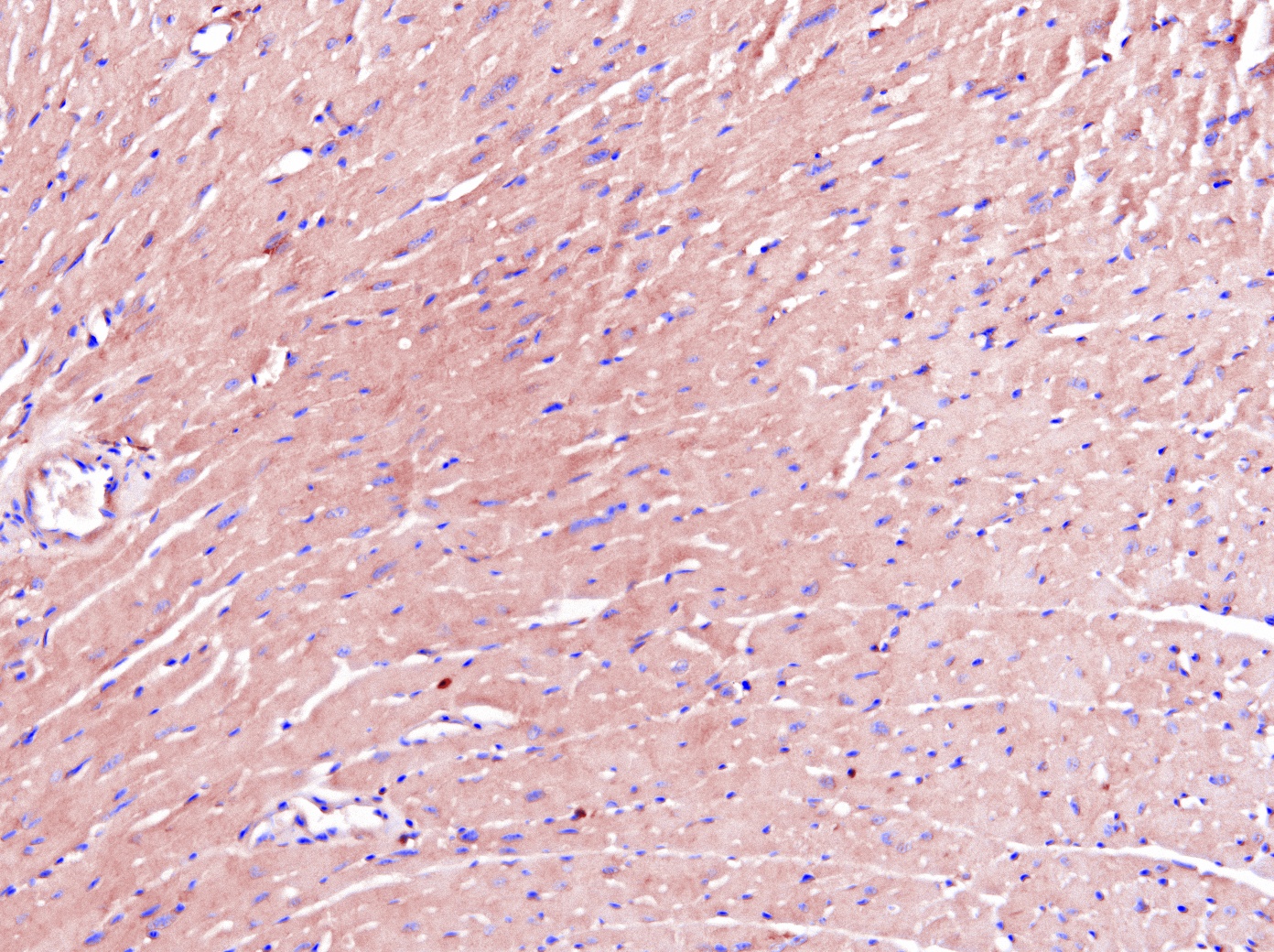

Supplement: Figure 4—source data 1. [file elife-55513-fig4-data1.zip › p16_images_for_eLife/p16_images_Ann_Chiao_for_eLife/Old Controls/OCL_10/Composite_9.1_g.jpg]

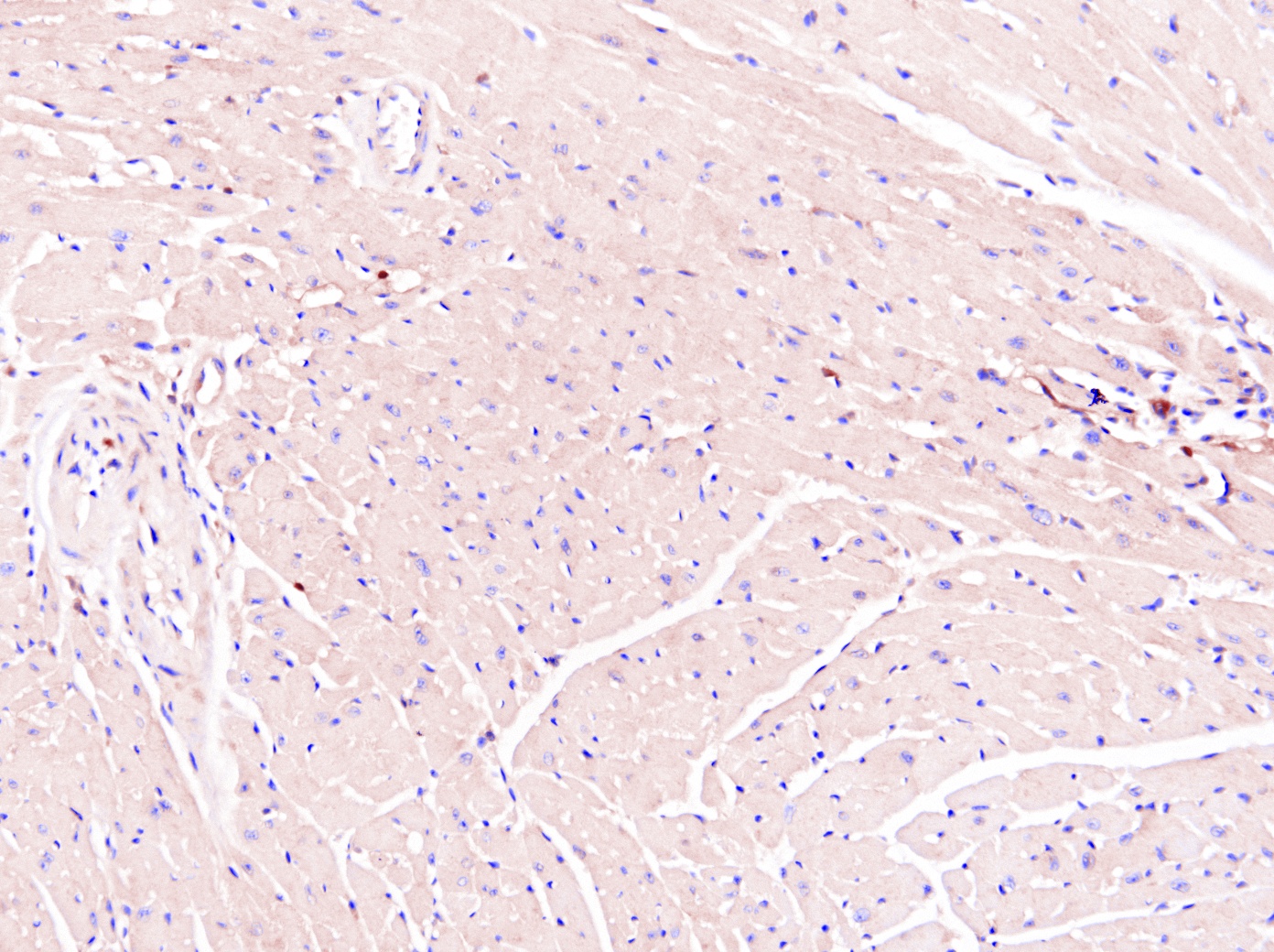

Supplement: Figure 4—source data 1. [file elife-55513-fig4-data1.zip › p16_images_for_eLife/p16_images_Ann_Chiao_for_eLife/Old Controls/OCL_10/Composite_9.1_h.jpg]

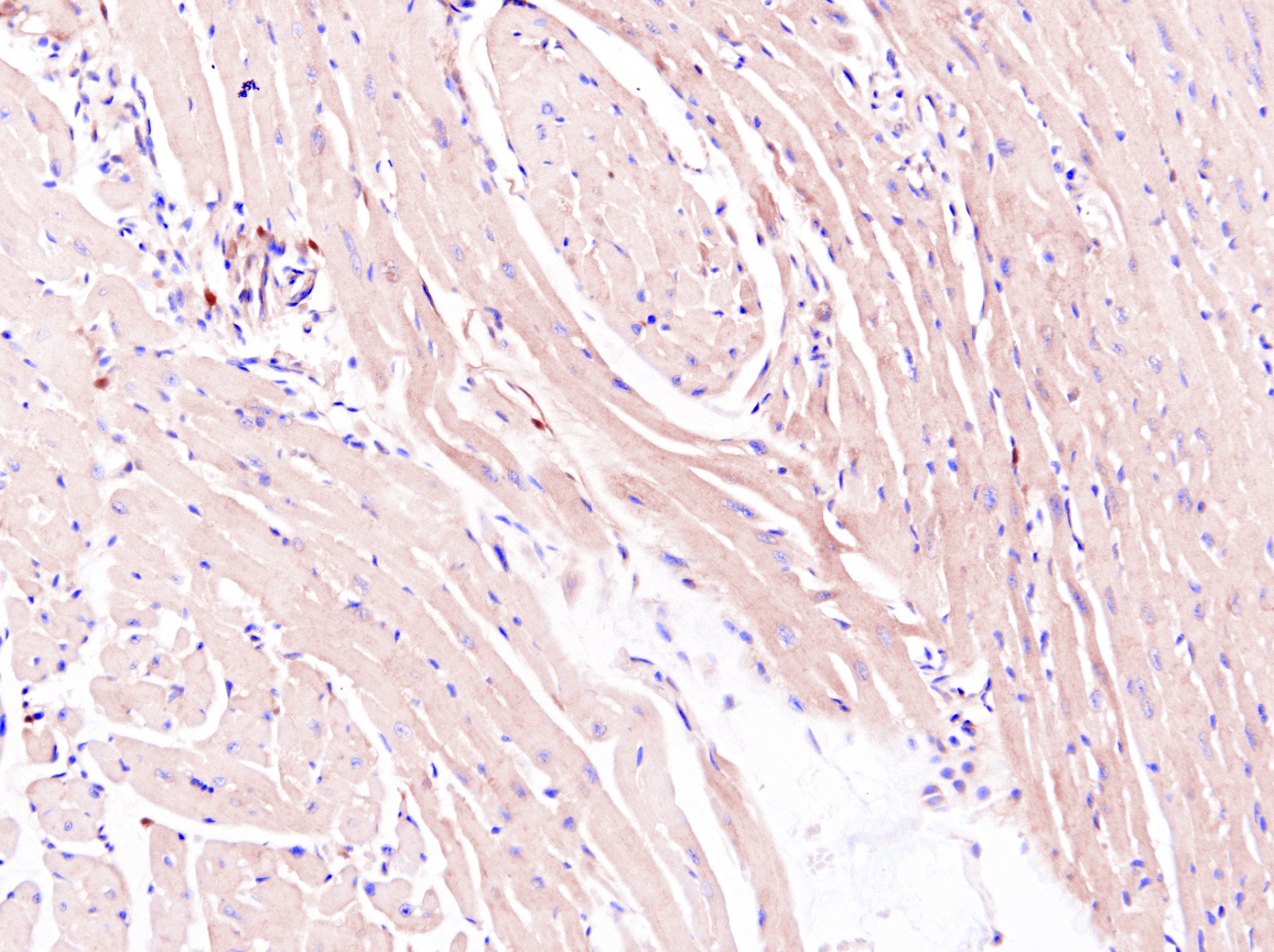

Supplement: Figure 4—source data 1. [file elife-55513-fig4-data1.zip › p16_images_for_eLife/p16_images_Ann_Chiao_for_eLife/Old Controls/OCL_10/Composite_9.1_i.jpg]

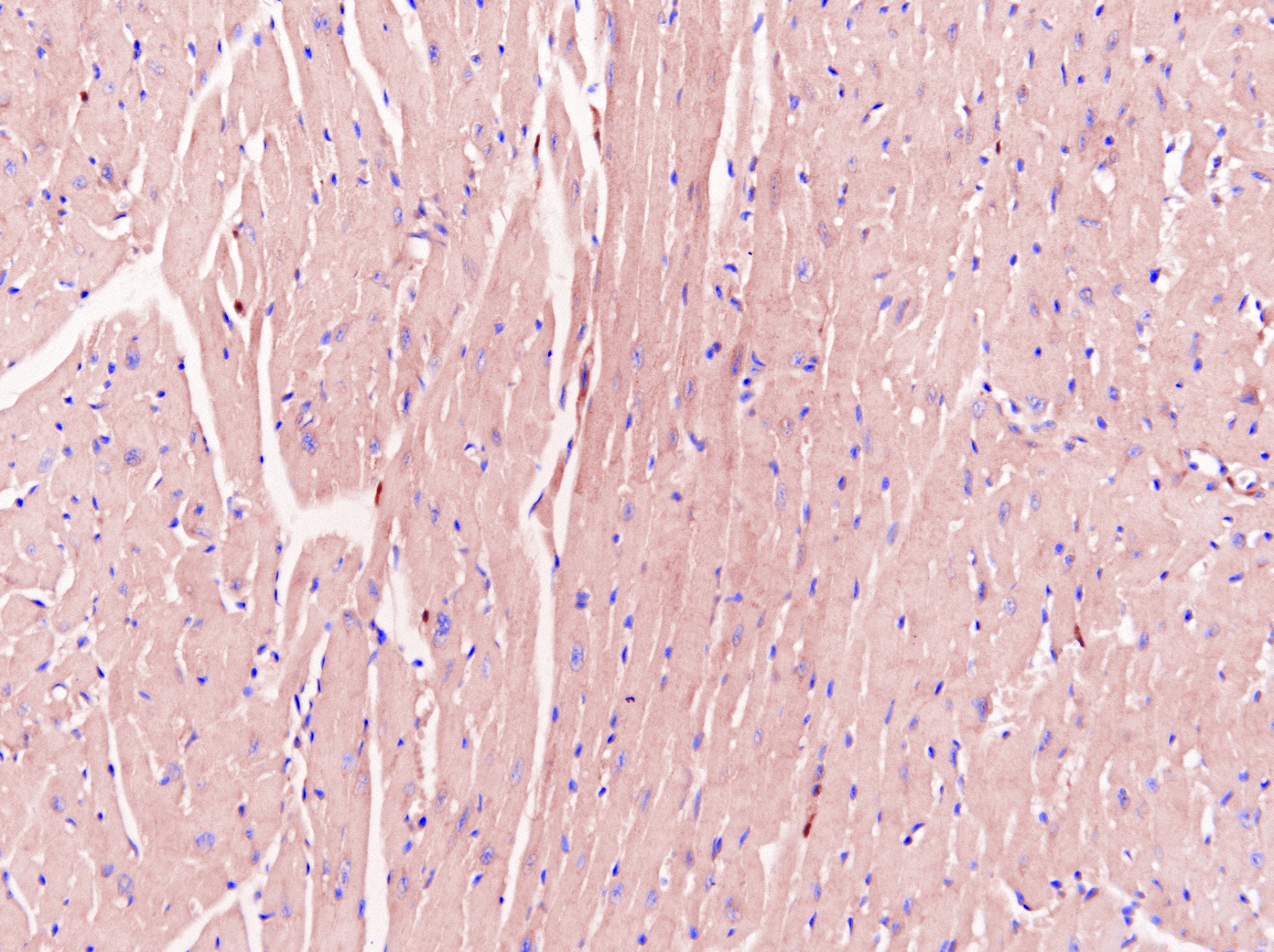

Supplement: Figure 4—source data 1. [file elife-55513-fig4-data1.zip › p16_images_for_eLife/p16_images_Ann_Chiao_for_eLife/Old Controls/OCL_10/Composite_9.1_j.jpg]

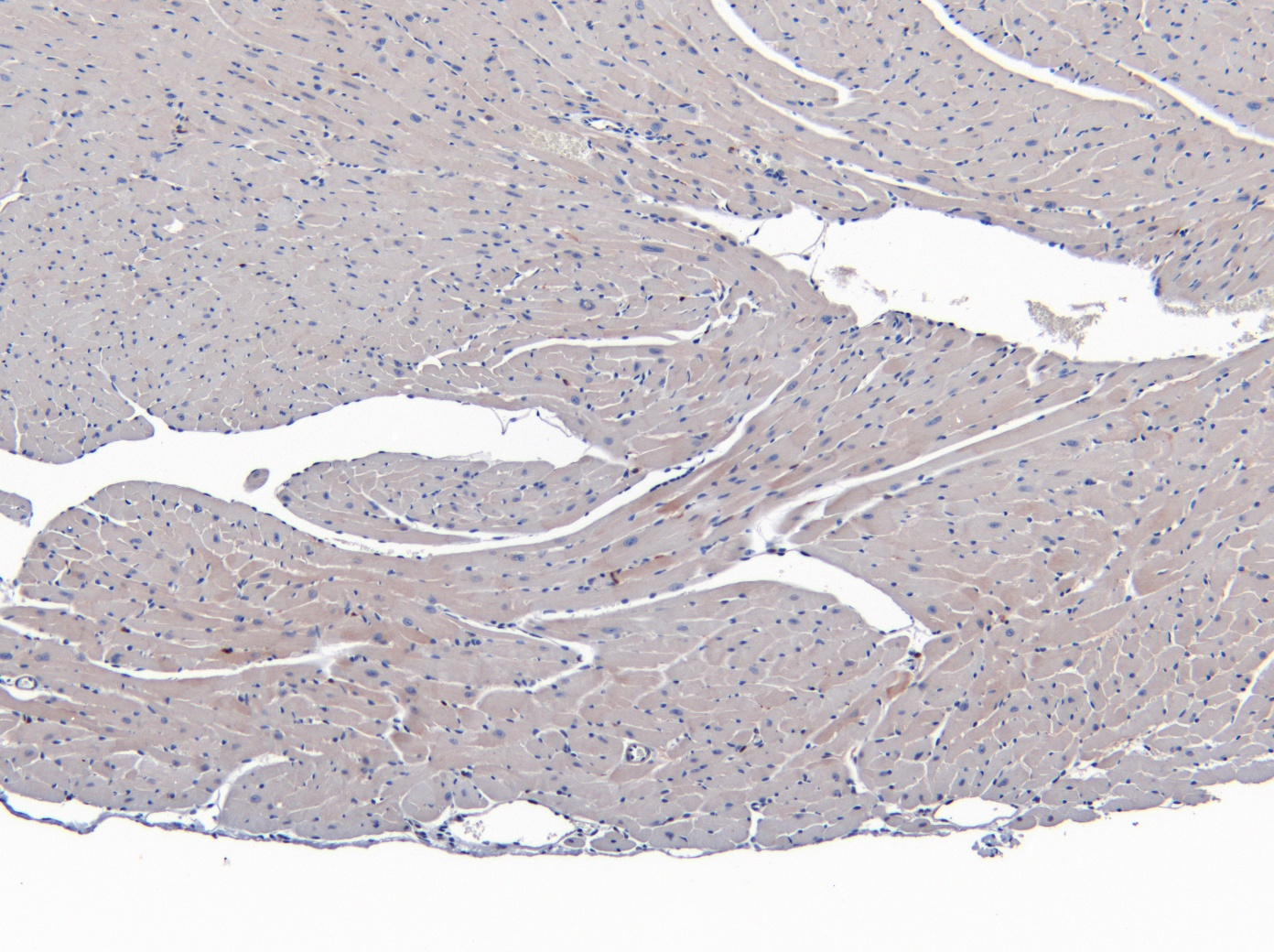

Supplement: Figure 4—source data 1. [file elife-55513-fig4-data1.zip › p16_images_for_eLife/p16_images_Ann_Chiao_for_eLife/Old Controls/OCL_2/MS_4_p16_10x_a_RGB.jpg]

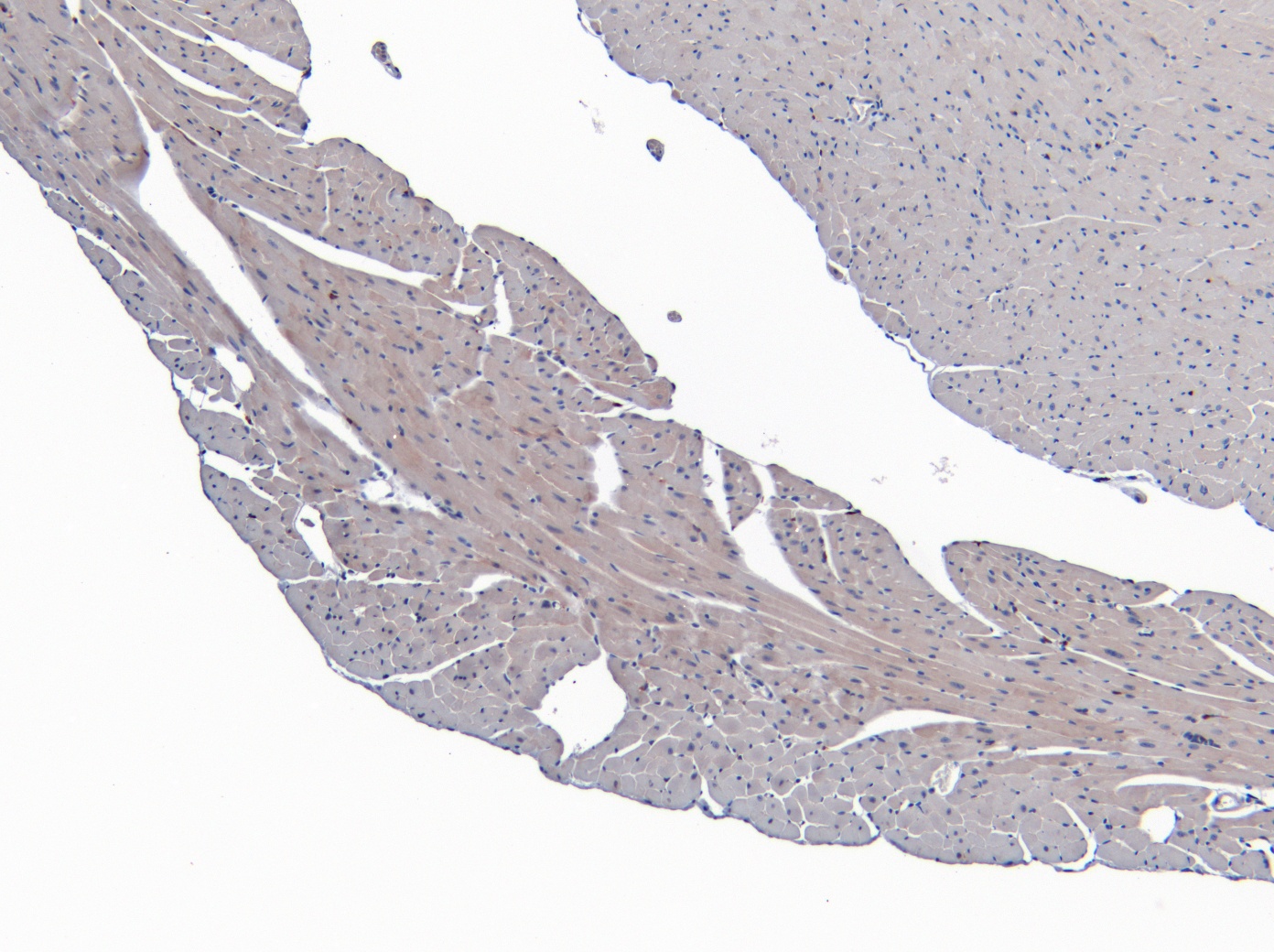

Supplement: Figure 4—source data 1. [file elife-55513-fig4-data1.zip › p16_images_for_eLife/p16_images_Ann_Chiao_for_eLife/Old Controls/OCL_2/MS_4_p16_10x_b_RGB.jpg]

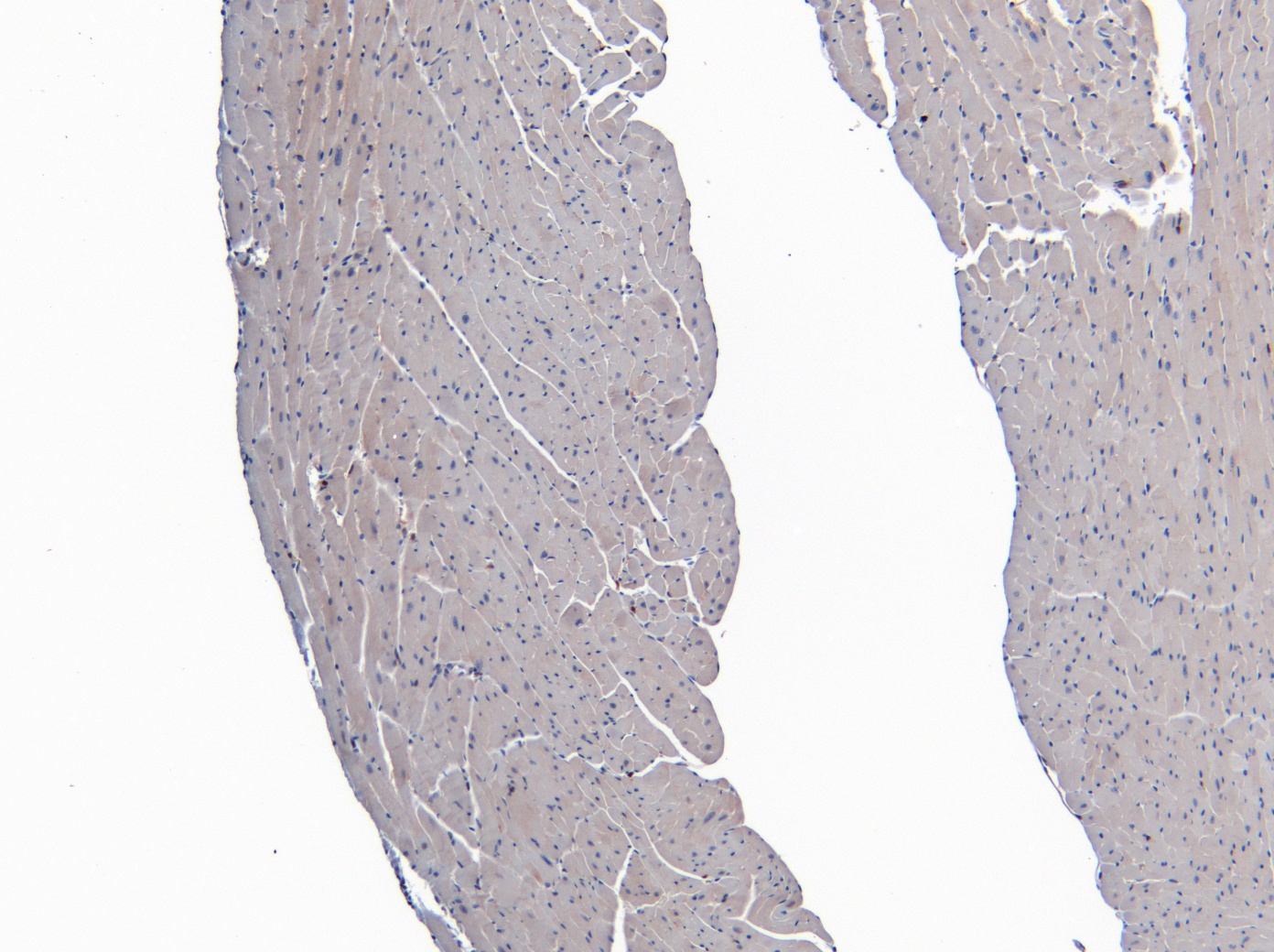

Supplement: Figure 4—source data 1. [file elife-55513-fig4-data1.zip › p16_images_for_eLife/p16_images_Ann_Chiao_for_eLife/Old Controls/OCL_2/MS_4_p16_10x_c_RGB.jpg]

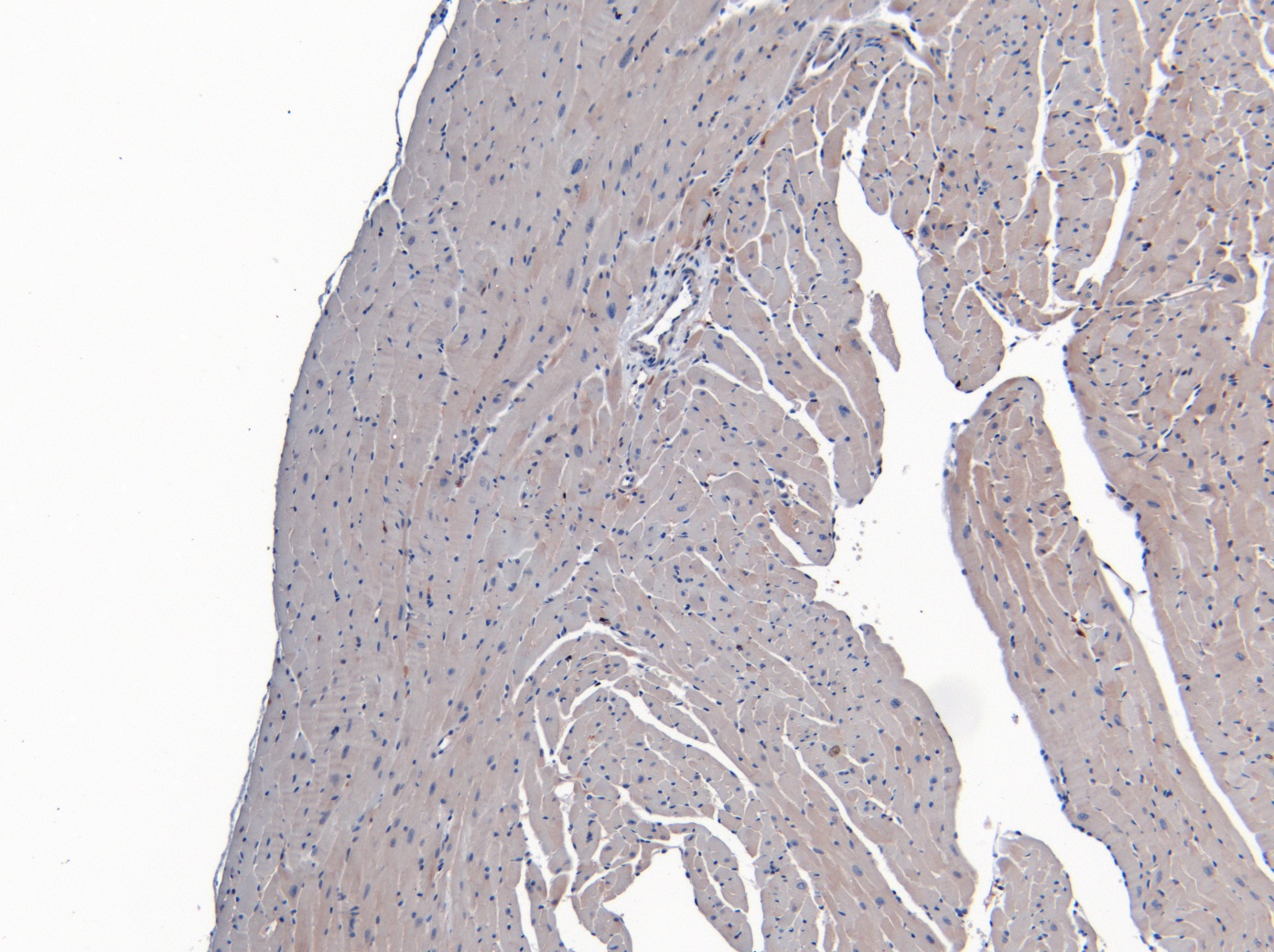

Supplement: Figure 4—source data 1. [file elife-55513-fig4-data1.zip › p16_images_for_eLife/p16_images_Ann_Chiao_for_eLife/Old Controls/OCL_2/MS_4_p16_10x_d_RGB.jpg]

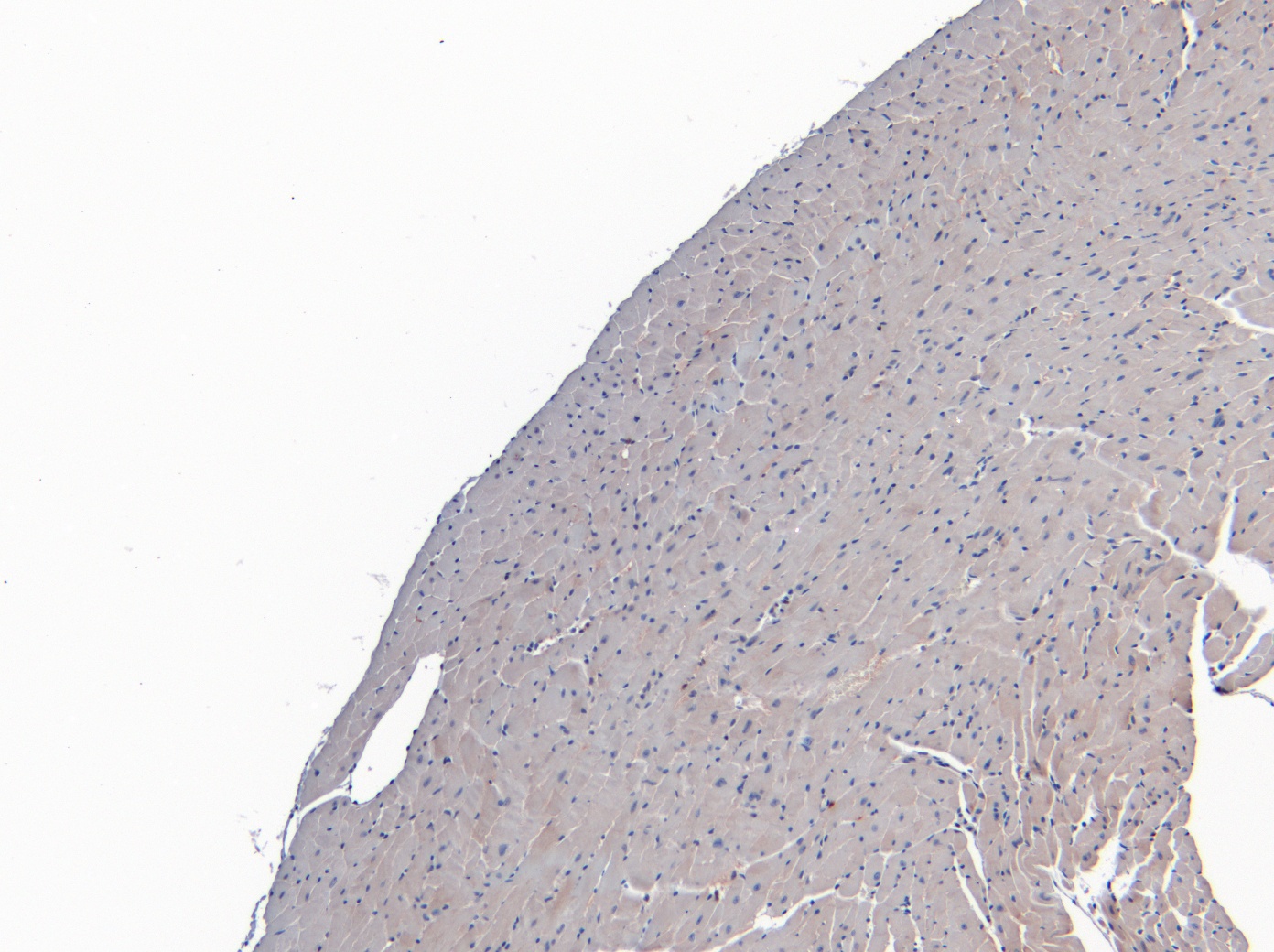

Supplement: Figure 4—source data 1. [file elife-55513-fig4-data1.zip › p16_images_for_eLife/p16_images_Ann_Chiao_for_eLife/Old Controls/OCL_2/MS_4_p16_10x_e_RGB.jpg]

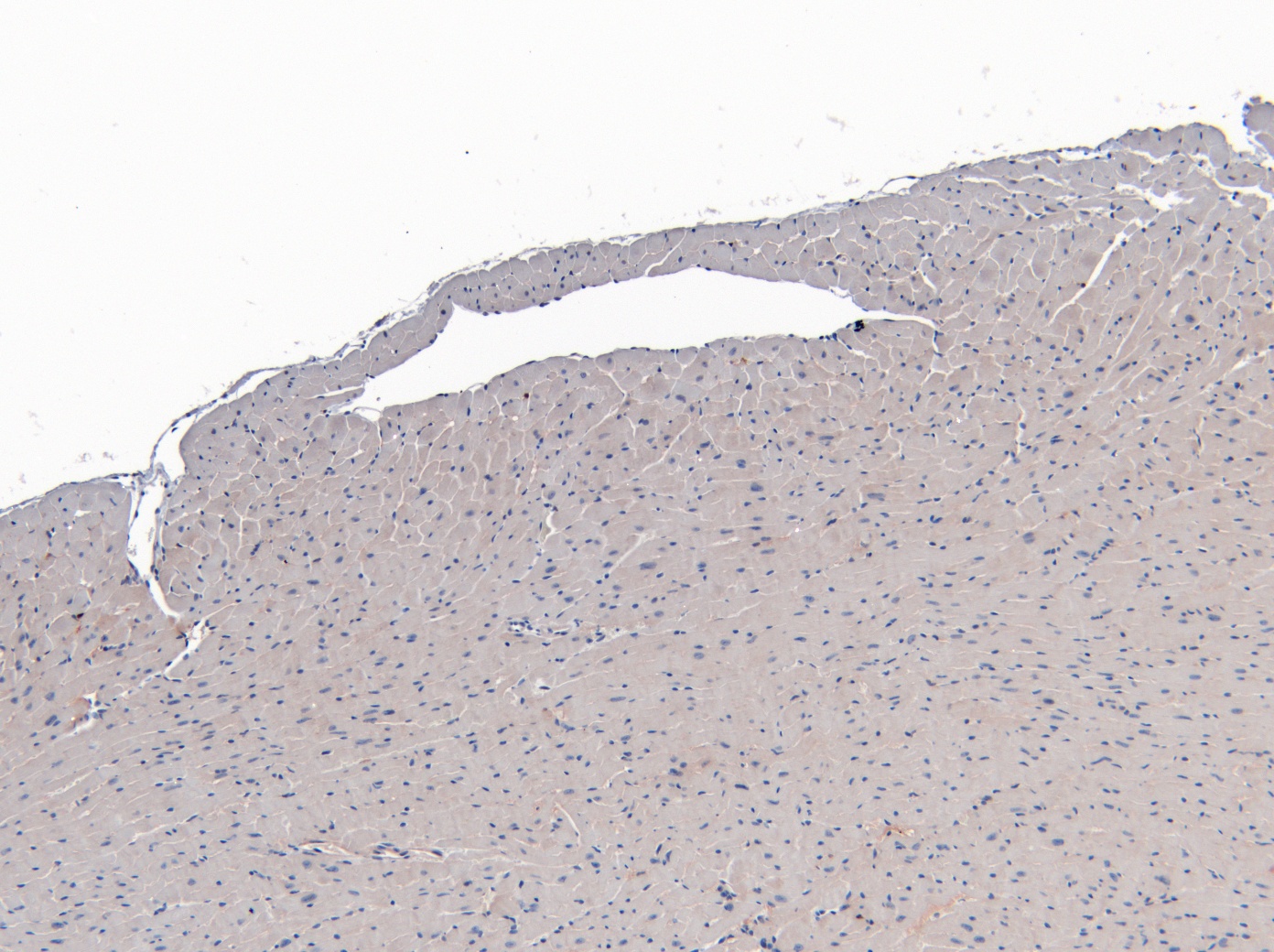

Supplement: Figure 4—source data 1. [file elife-55513-fig4-data1.zip › p16_images_for_eLife/p16_images_Ann_Chiao_for_eLife/Old Controls/OCL_2/MS_4_p16_10x_f_RGB.jpg]

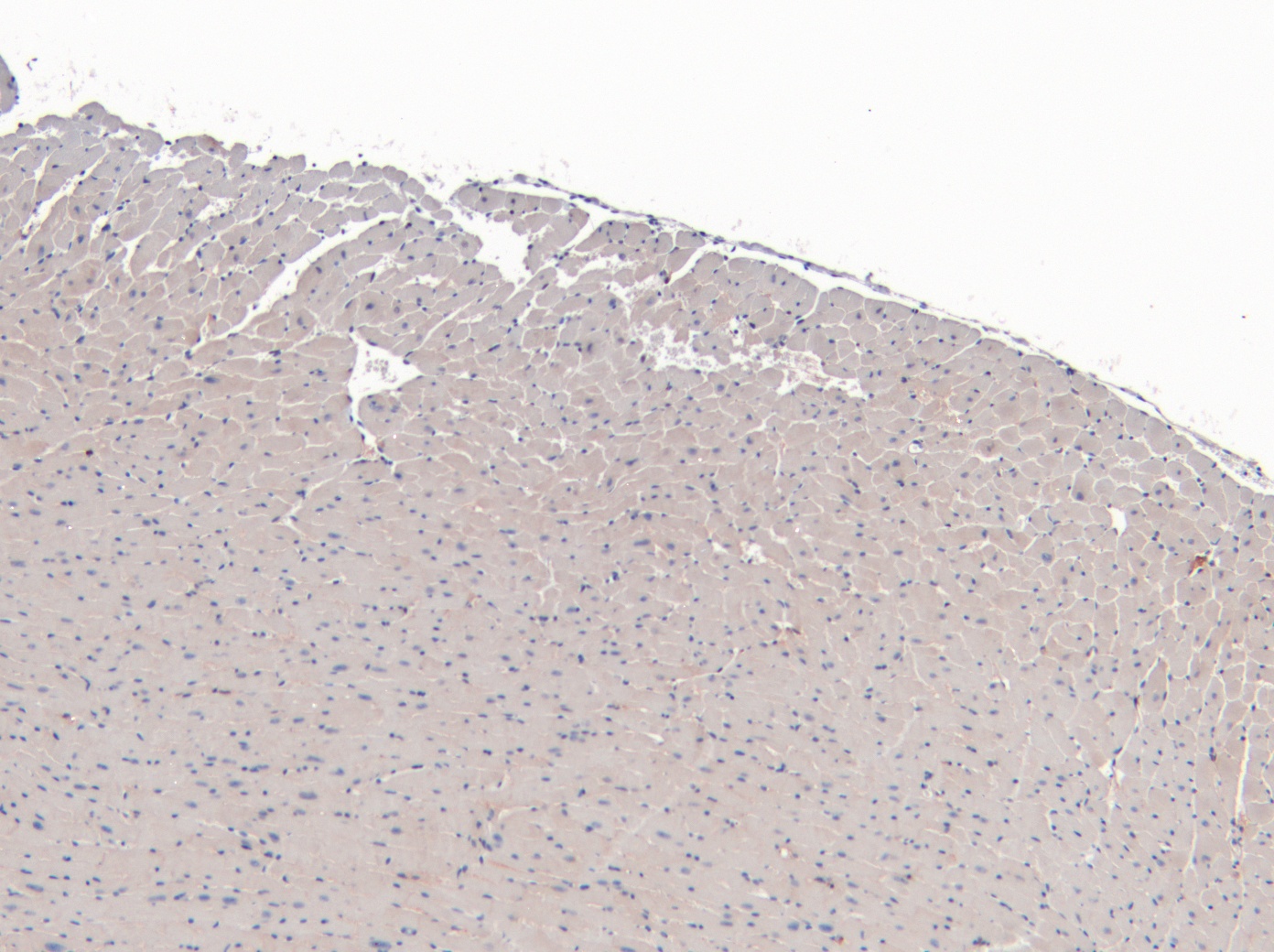

Supplement: Figure 4—source data 1. [file elife-55513-fig4-data1.zip › p16_images_for_eLife/p16_images_Ann_Chiao_for_eLife/Old Controls/OCL_2/MS_4_p16_10x_g_RGB.jpg]

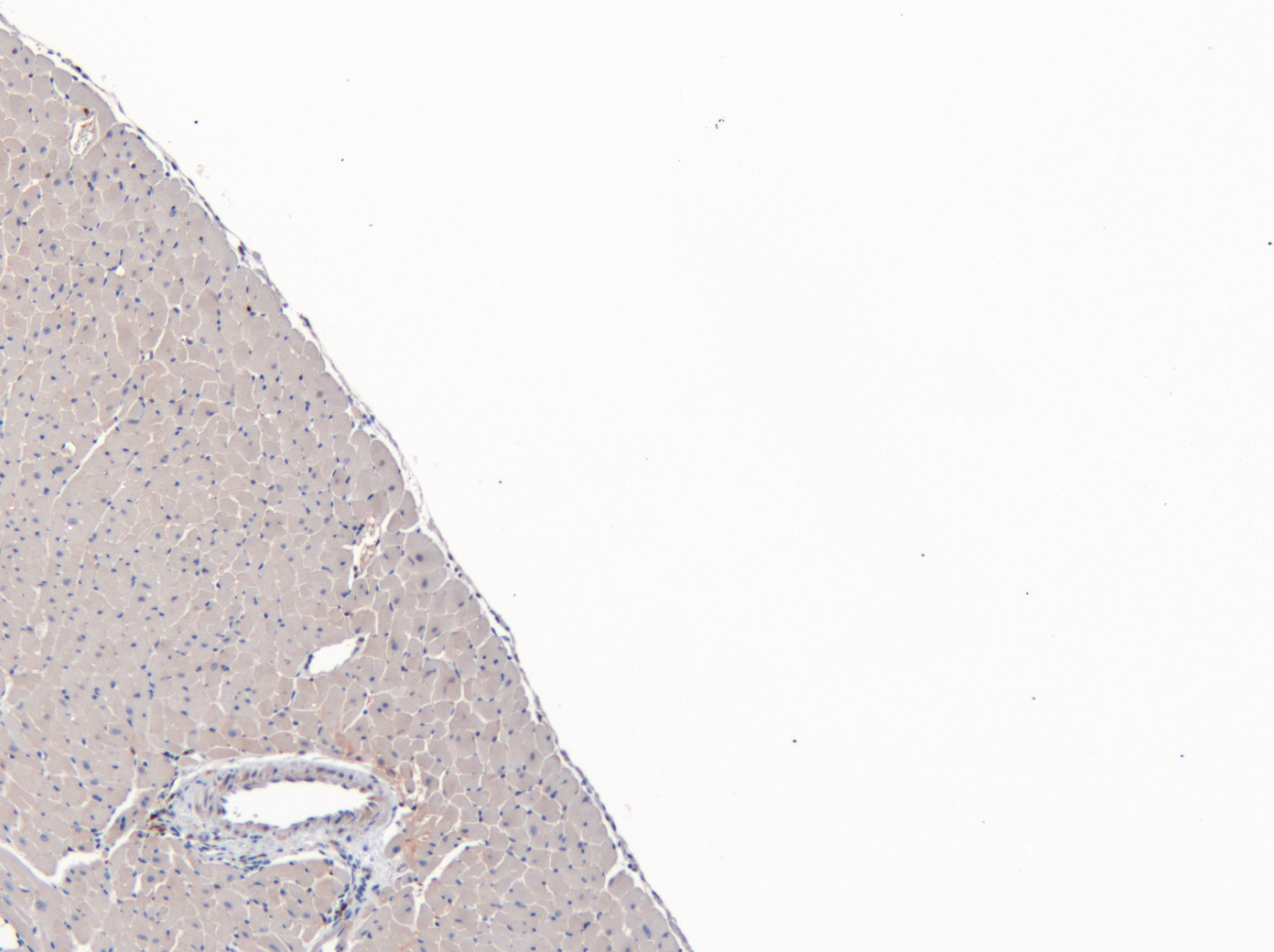

Supplement: Figure 4—source data 1. [file elife-55513-fig4-data1.zip › p16_images_for_eLife/p16_images_Ann_Chiao_for_eLife/Old Controls/OCL_2/MS_4_p16_10x_h_RGB.jpg]

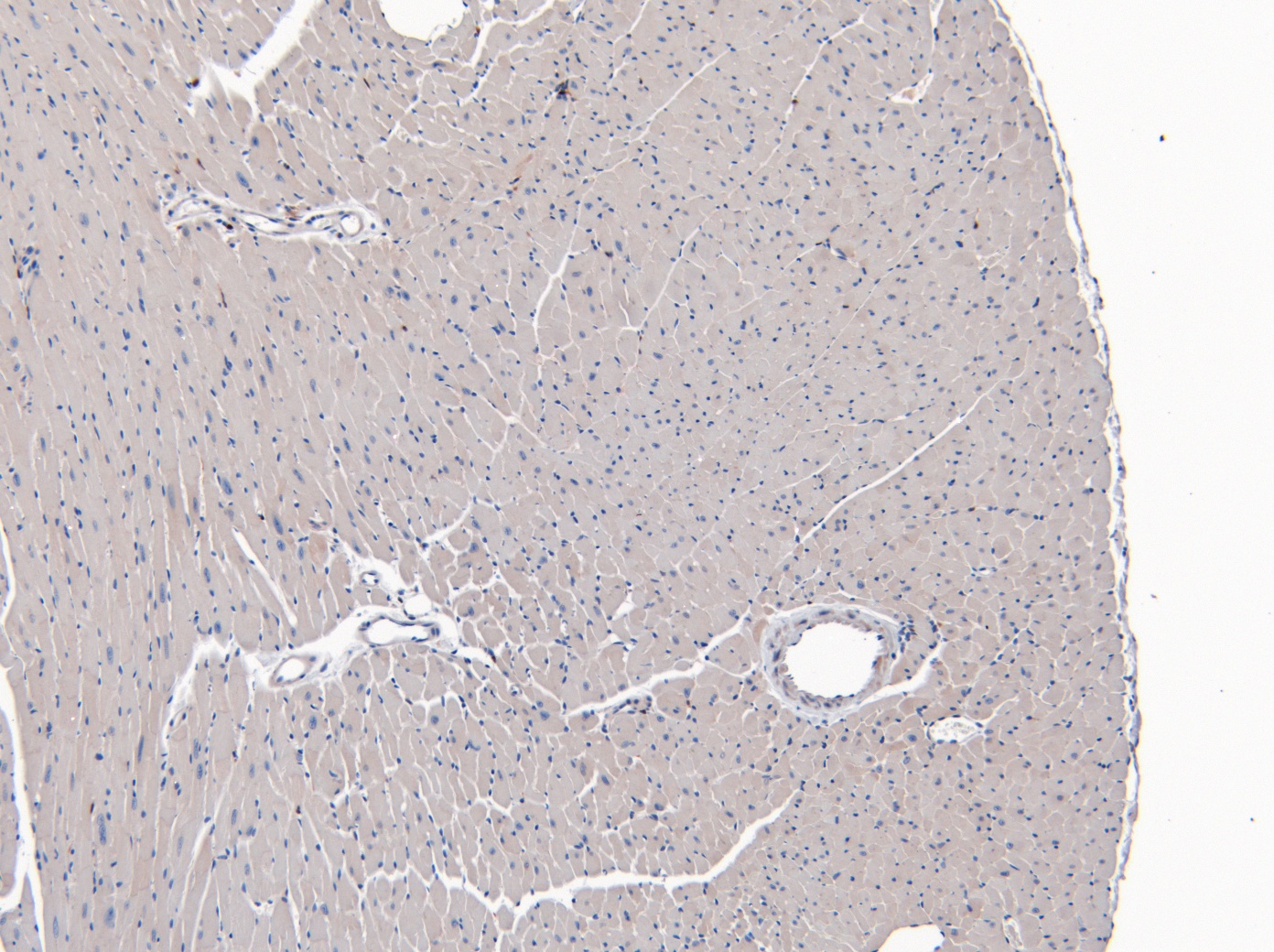

Supplement: Figure 4—source data 1. [file elife-55513-fig4-data1.zip › p16_images_for_eLife/p16_images_Ann_Chiao_for_eLife/Old Controls/OCL_2/MS_4_p16_10x_i_RGB.jpg]

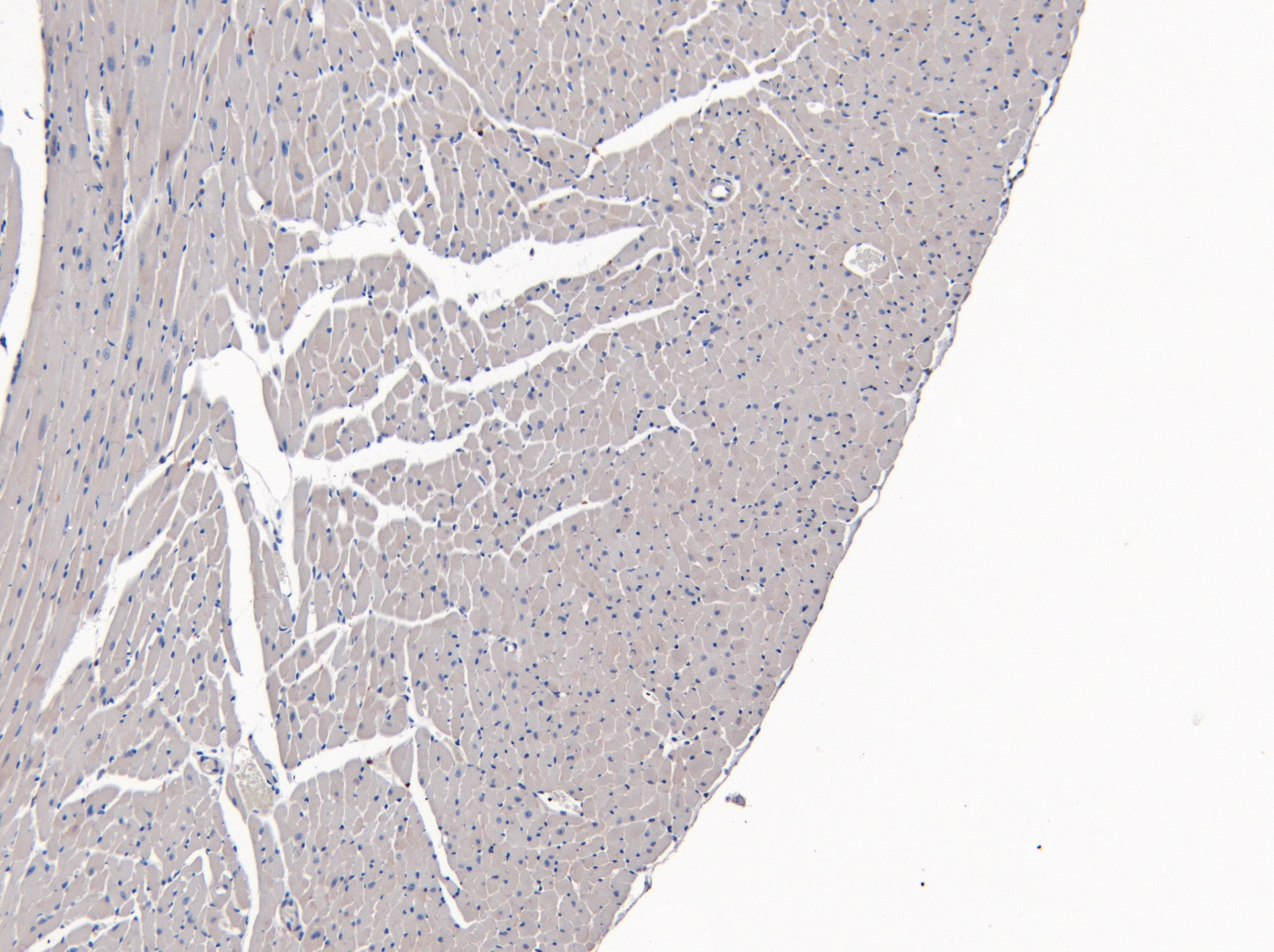

Supplement: Figure 4—source data 1. [file elife-55513-fig4-data1.zip › p16_images_for_eLife/p16_images_Ann_Chiao_for_eLife/Old Controls/OCL_2/MS_4_p16_10x_j_RGB.jpg]

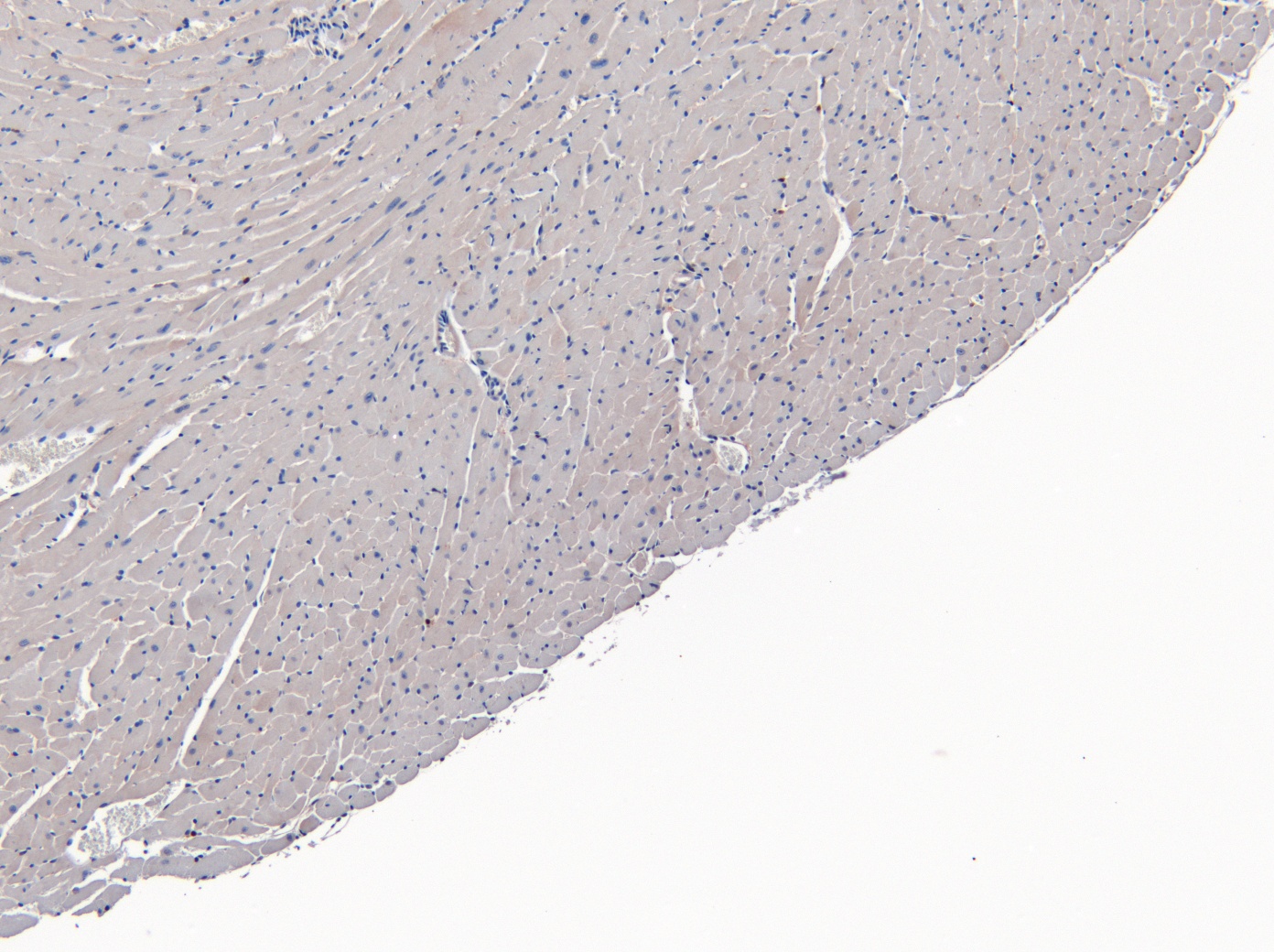

Supplement: Figure 4—source data 1. [file elife-55513-fig4-data1.zip › p16_images_for_eLife/p16_images_Ann_Chiao_for_eLife/Old Controls/OCL_2/MS_4_p16_10x_k_RGB.jpg]

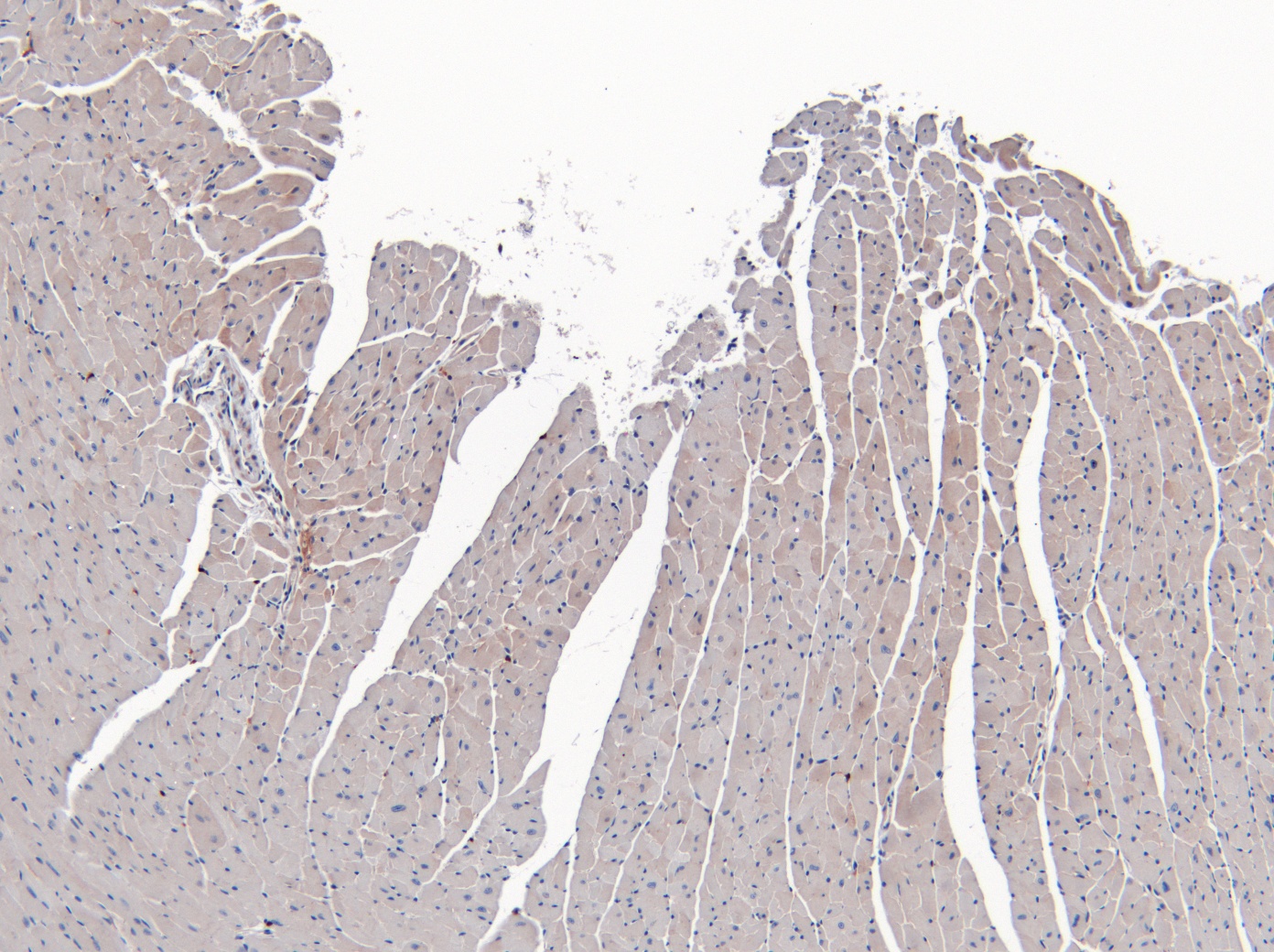

Supplement: Figure 4—source data 1. [file elife-55513-fig4-data1.zip › p16_images_for_eLife/p16_images_Ann_Chiao_for_eLife/Old Controls/OCL_2/MS_4_p16_10x_l_RGB.jpg]

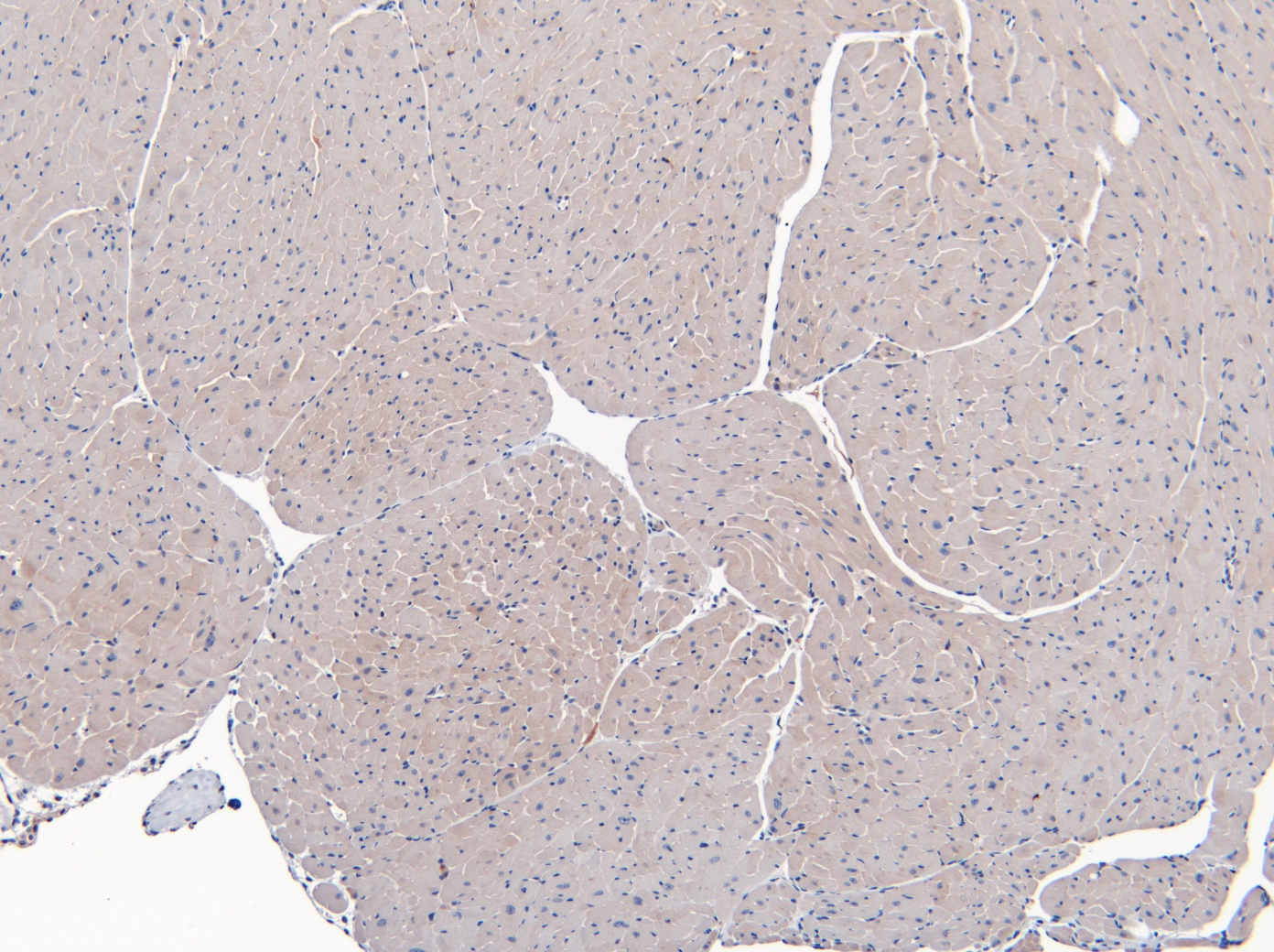

Supplement: Figure 4—source data 1. [file elife-55513-fig4-data1.zip › p16_images_for_eLife/p16_images_Ann_Chiao_for_eLife/Old Controls/OCL_2/MS_4_p16_10x_m_RGB.jpg]

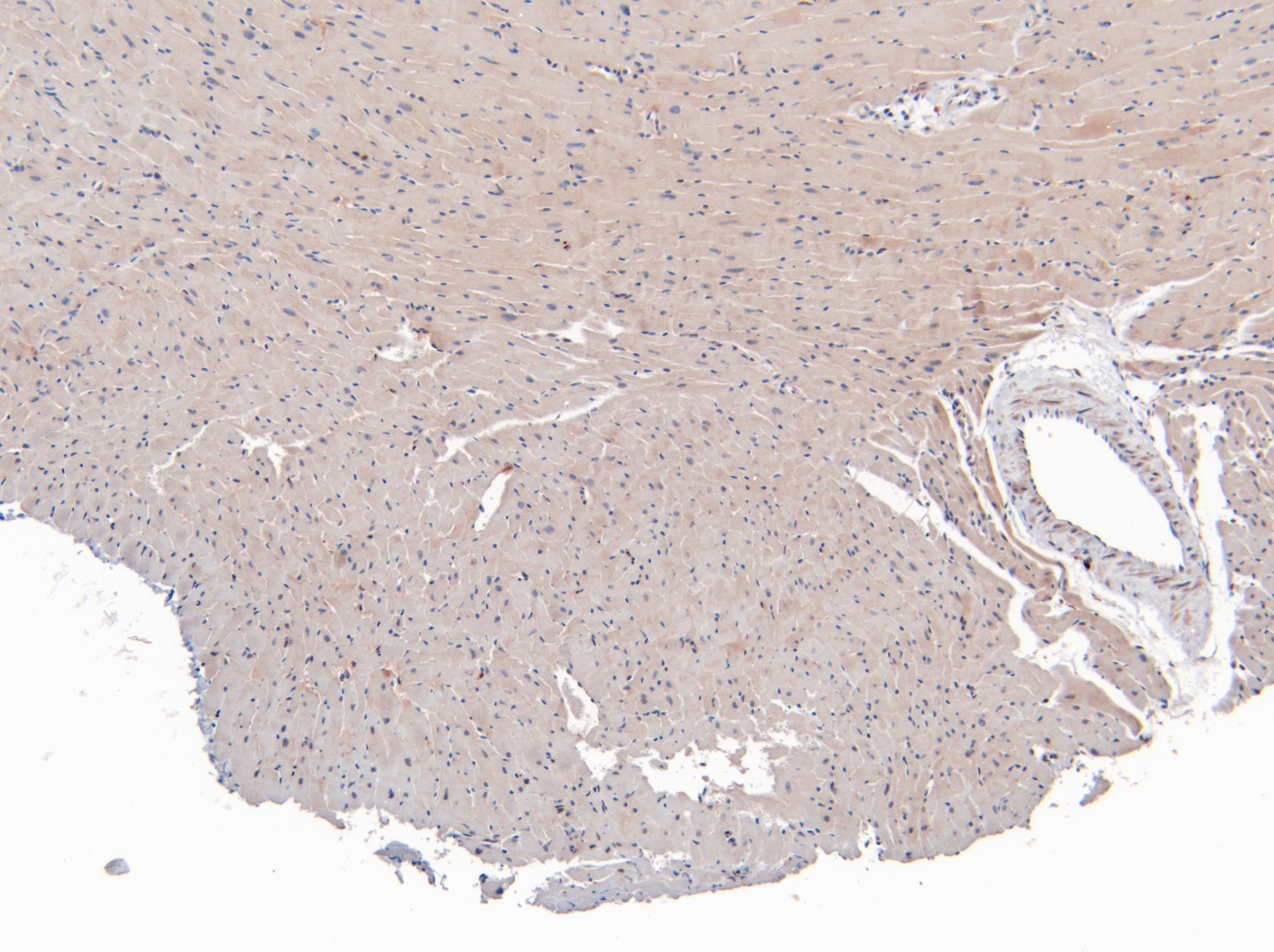

Supplement: Figure 4—source data 1. [file elife-55513-fig4-data1.zip › p16_images_for_eLife/p16_images_Ann_Chiao_for_eLife/Old Controls/OCL_3/MS_5_p16_10x_a_RGB.jpg]

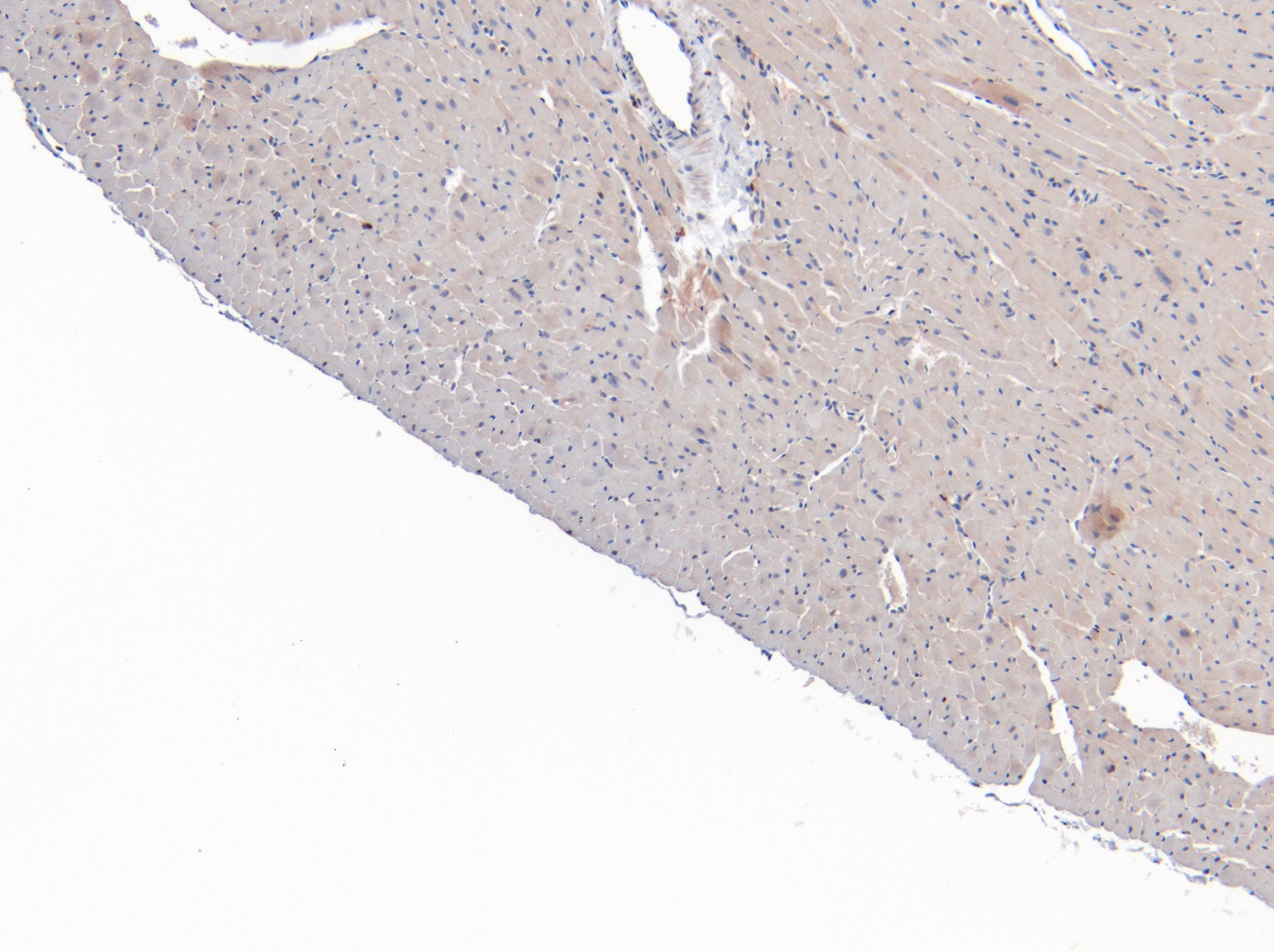

Supplement: Figure 4—source data 1. [file elife-55513-fig4-data1.zip › p16_images_for_eLife/p16_images_Ann_Chiao_for_eLife/Old Controls/OCL_3/MS_5_p16_10x_b_RGB.jpg]

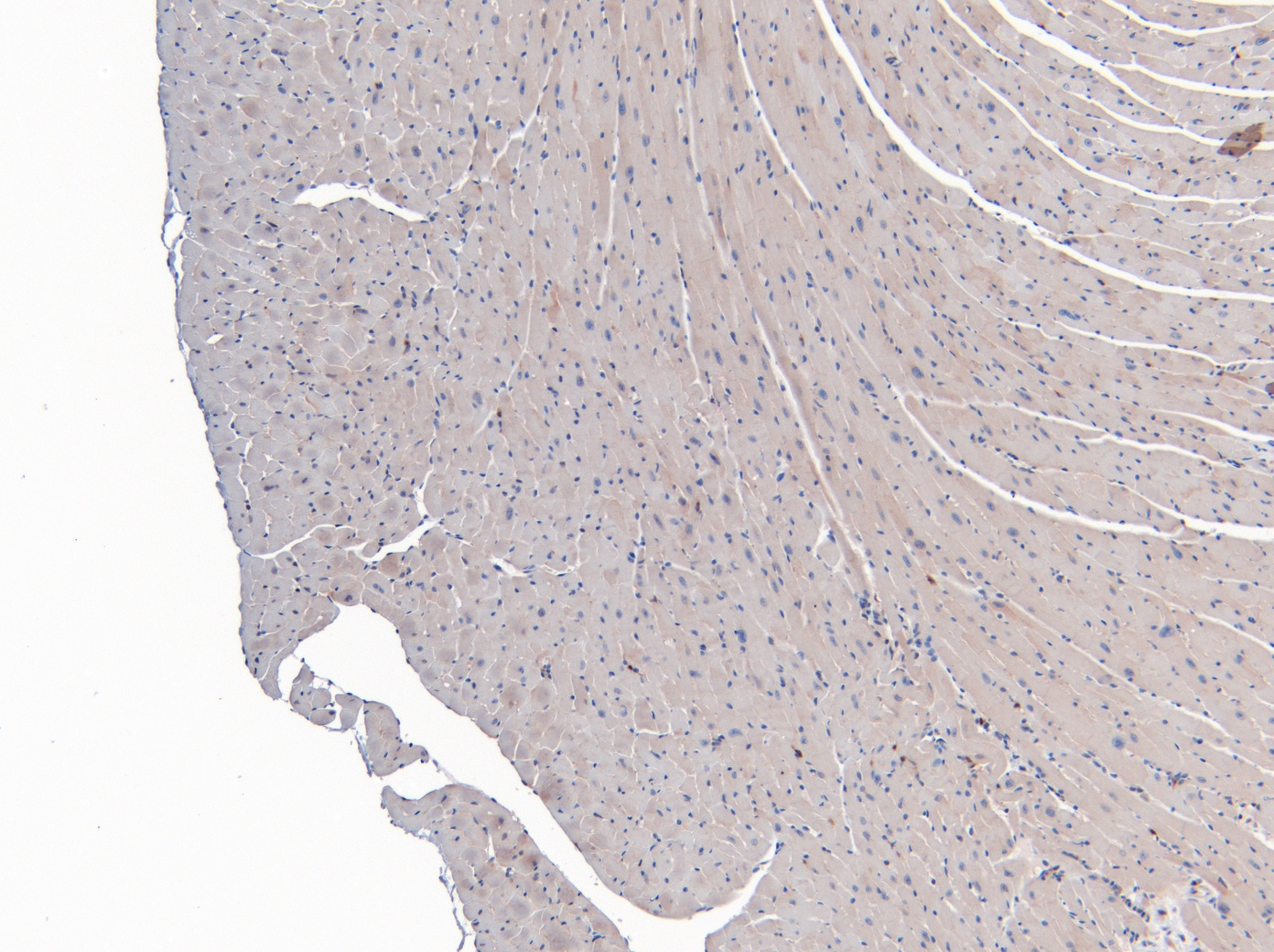

Supplement: Figure 4—source data 1. [file elife-55513-fig4-data1.zip › p16_images_for_eLife/p16_images_Ann_Chiao_for_eLife/Old Controls/OCL_3/MS_5_p16_10x_c_RGB.jpg]

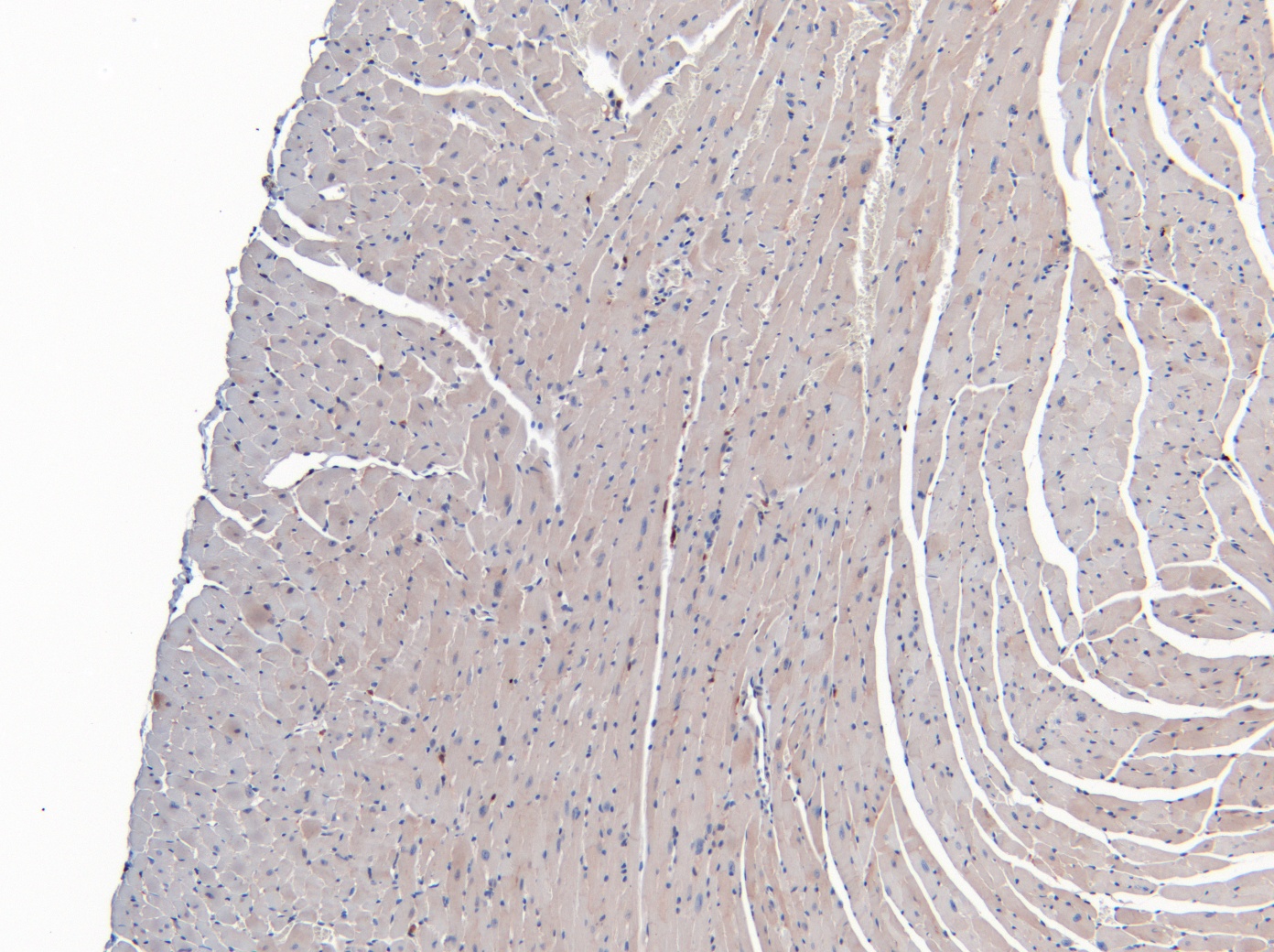

Supplement: Figure 4—source data 1. [file elife-55513-fig4-data1.zip › p16_images_for_eLife/p16_images_Ann_Chiao_for_eLife/Old Controls/OCL_3/MS_5_p16_10x_d_RGB.jpg]

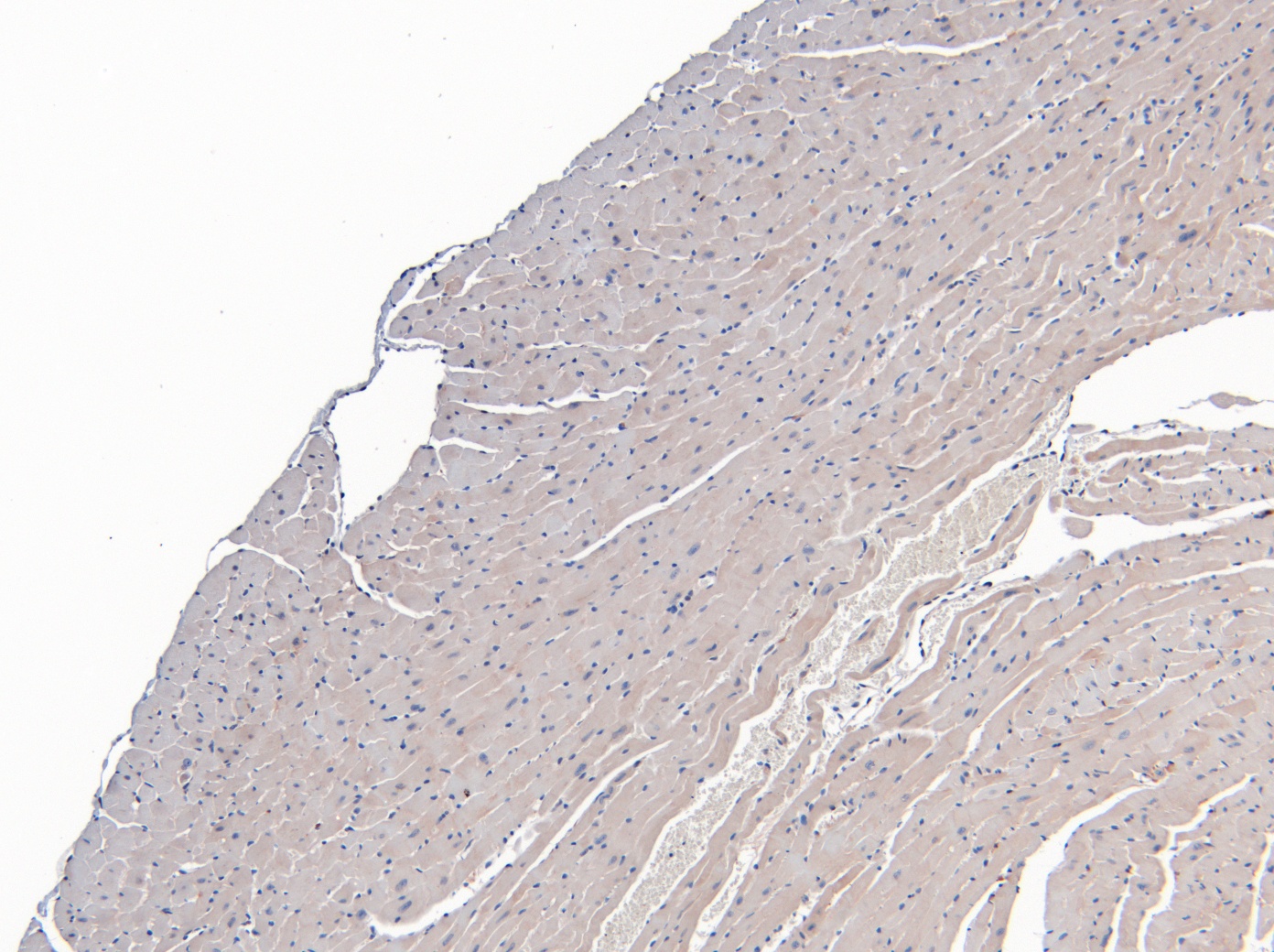

Supplement: Figure 4—source data 1. [file elife-55513-fig4-data1.zip › p16_images_for_eLife/p16_images_Ann_Chiao_for_eLife/Old Controls/OCL_3/MS_5_p16_10x_e_RGB.jpg]

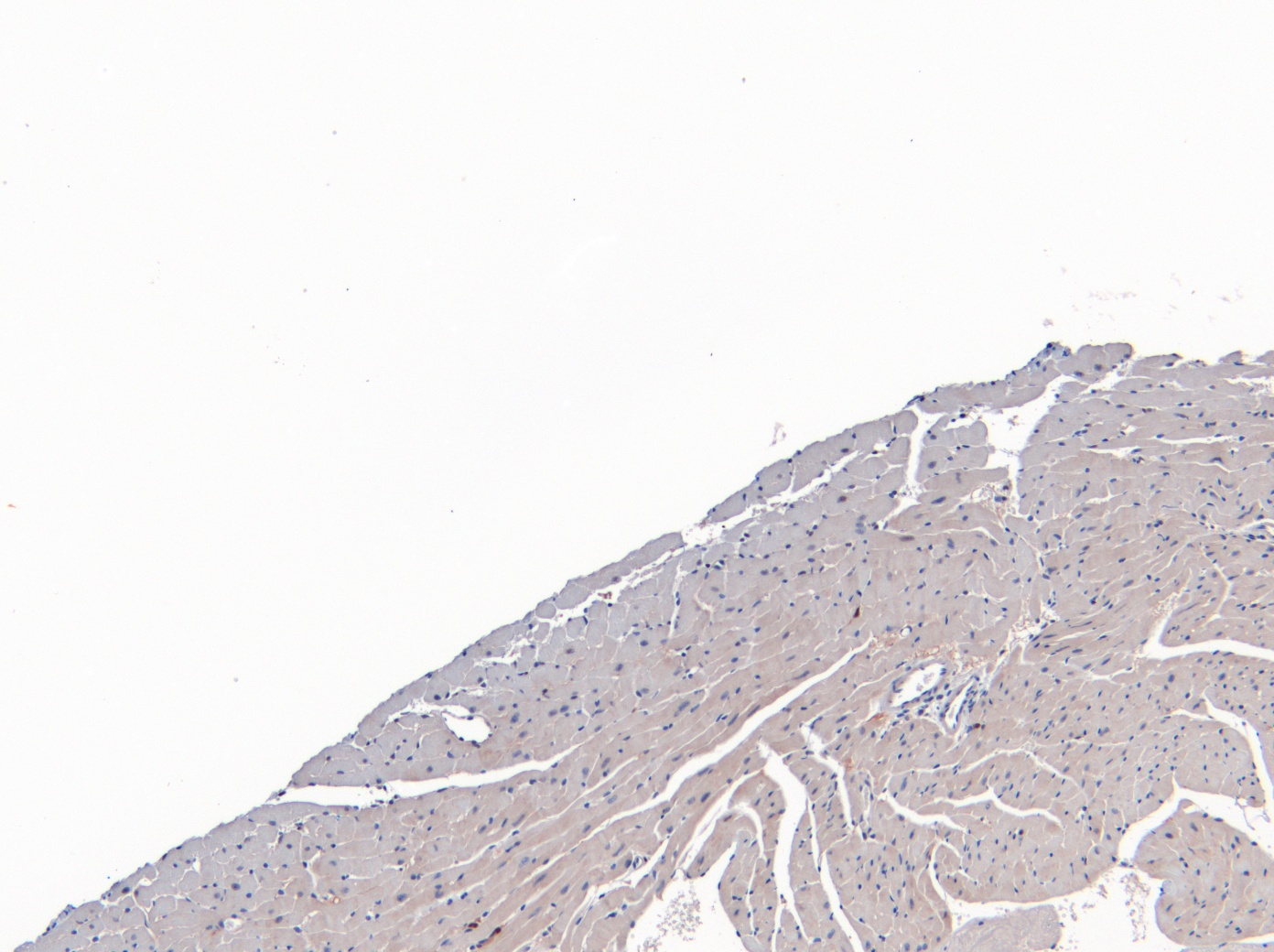

Supplement: Figure 4—source data 1. [file elife-55513-fig4-data1.zip › p16_images_for_eLife/p16_images_Ann_Chiao_for_eLife/Old Controls/OCL_3/MS_5_p16_10x_f_RGB.jpg]

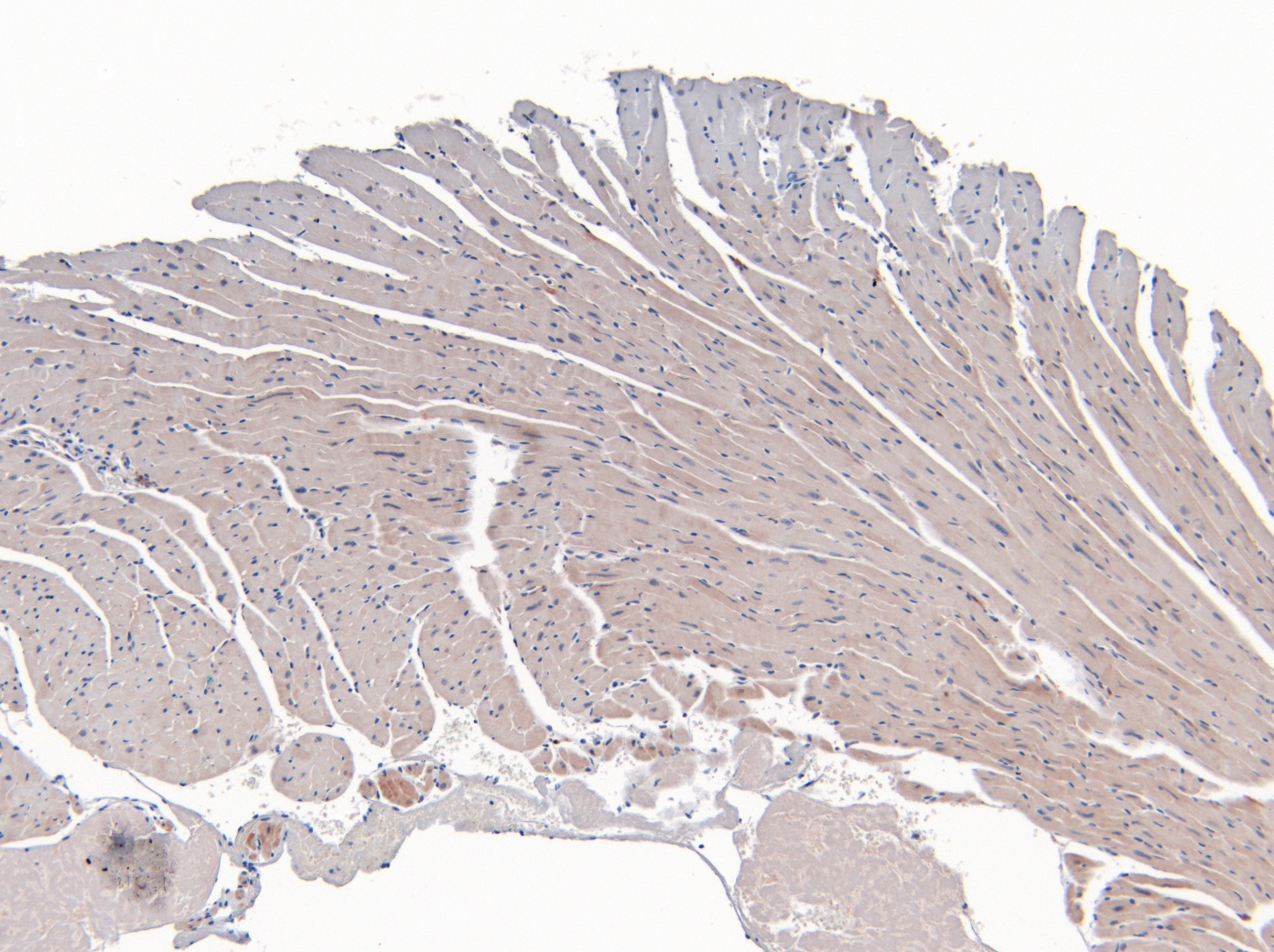

Supplement: Figure 4—source data 1. [file elife-55513-fig4-data1.zip › p16_images_for_eLife/p16_images_Ann_Chiao_for_eLife/Old Controls/OCL_3/MS_5_p16_10x_g_RGB.jpg]

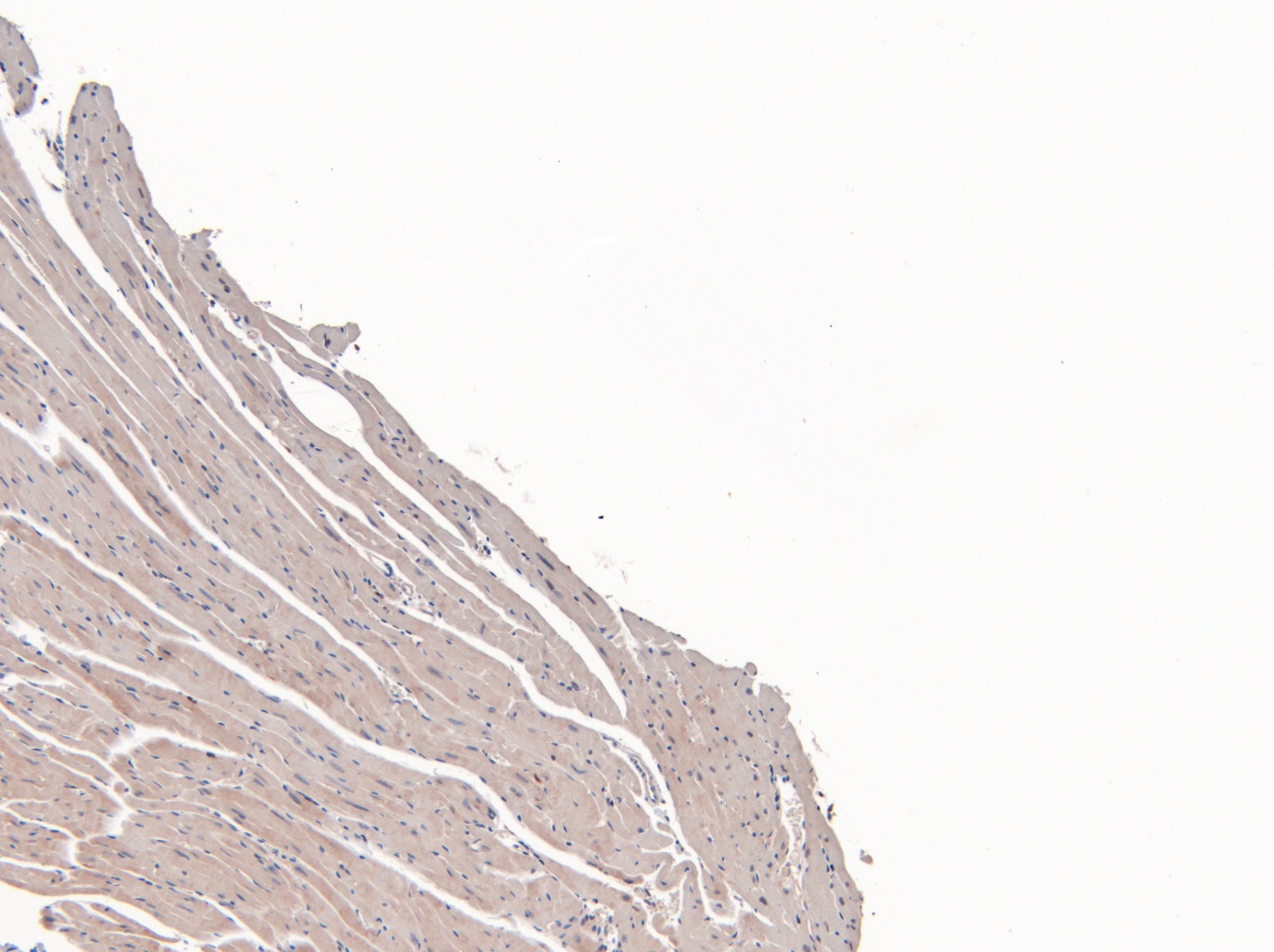

Supplement: Figure 4—source data 1. [file elife-55513-fig4-data1.zip › p16_images_for_eLife/p16_images_Ann_Chiao_for_eLife/Old Controls/OCL_3/MS_5_p16_10x_h_RGB.jpg]

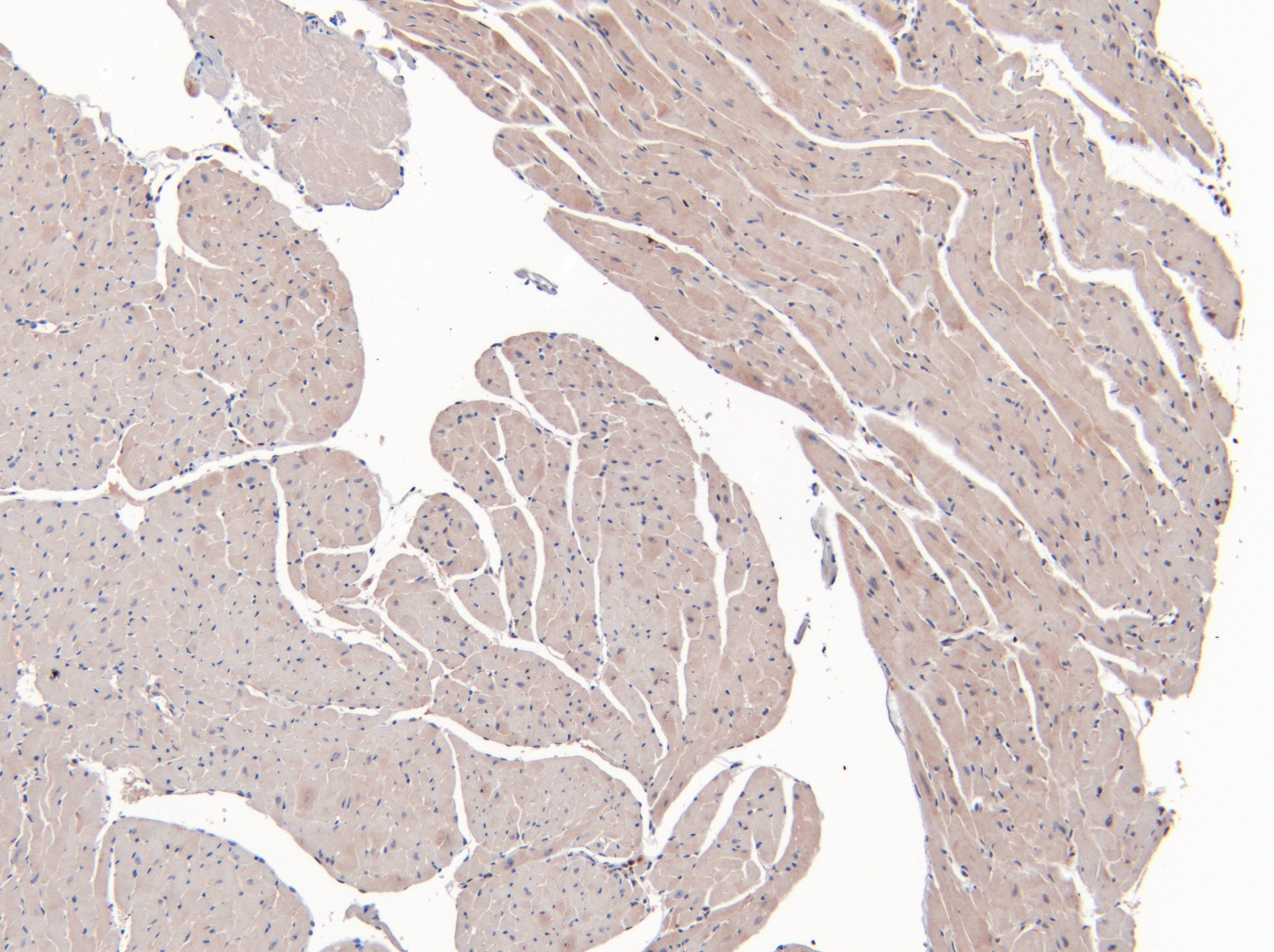

Supplement: Figure 4—source data 1. [file elife-55513-fig4-data1.zip › p16_images_for_eLife/p16_images_Ann_Chiao_for_eLife/Old Controls/OCL_3/MS_5_p16_10x_i_RGB.jpg]

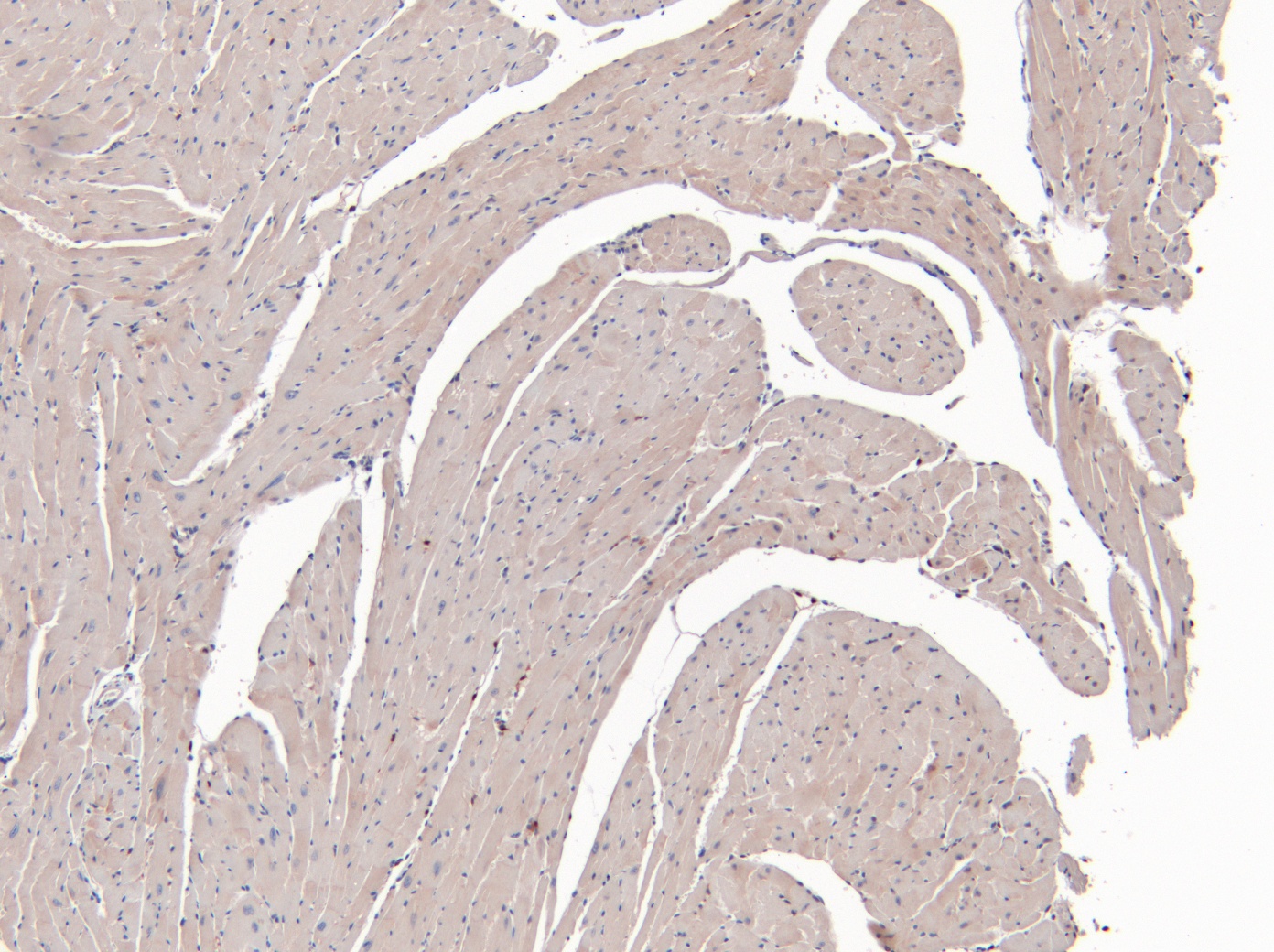

Supplement: Figure 4—source data 1. [file elife-55513-fig4-data1.zip › p16_images_for_eLife/p16_images_Ann_Chiao_for_eLife/Old Controls/OCL_3/MS_5_p16_10x_j_RGB.jpg]

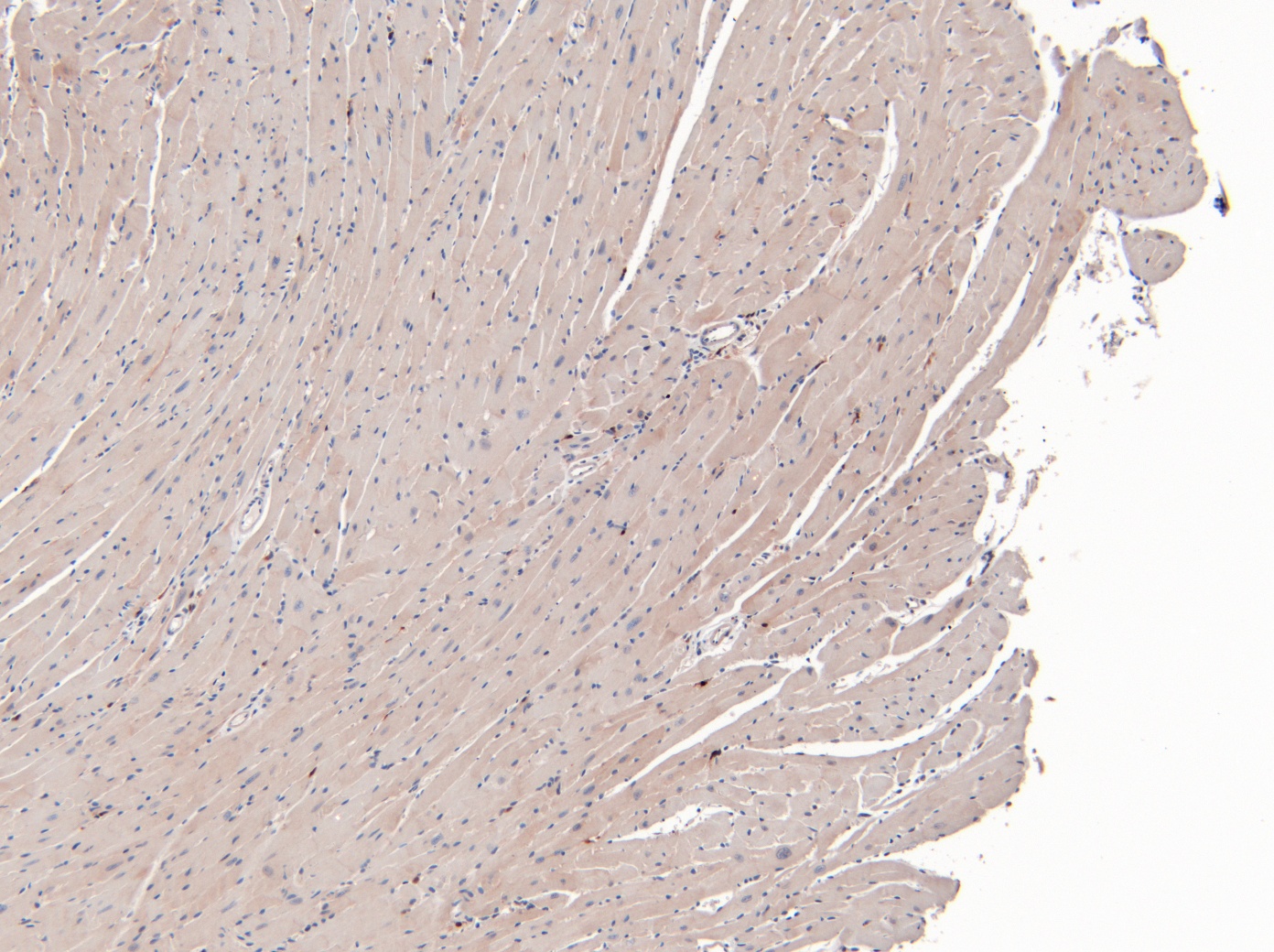

Supplement: Figure 4—source data 1. [file elife-55513-fig4-data1.zip › p16_images_for_eLife/p16_images_Ann_Chiao_for_eLife/Old Controls/OCL_3/MS_5_p16_10x_k_RGB.jpg]

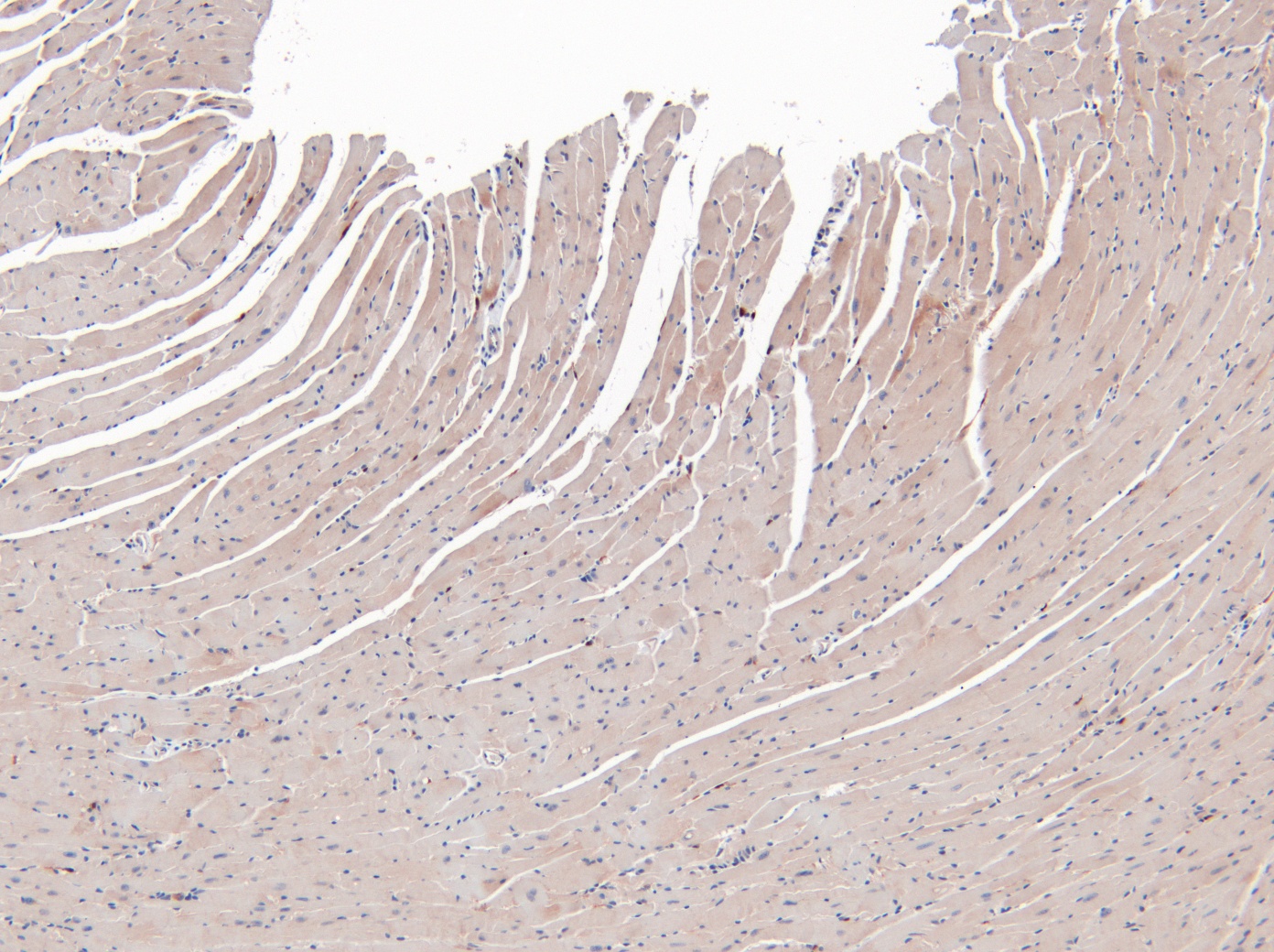

Supplement: Figure 4—source data 1. [file elife-55513-fig4-data1.zip › p16_images_for_eLife/p16_images_Ann_Chiao_for_eLife/Old Controls/OCL_3/MS_5_p16_10x_l_RGB.jpg]

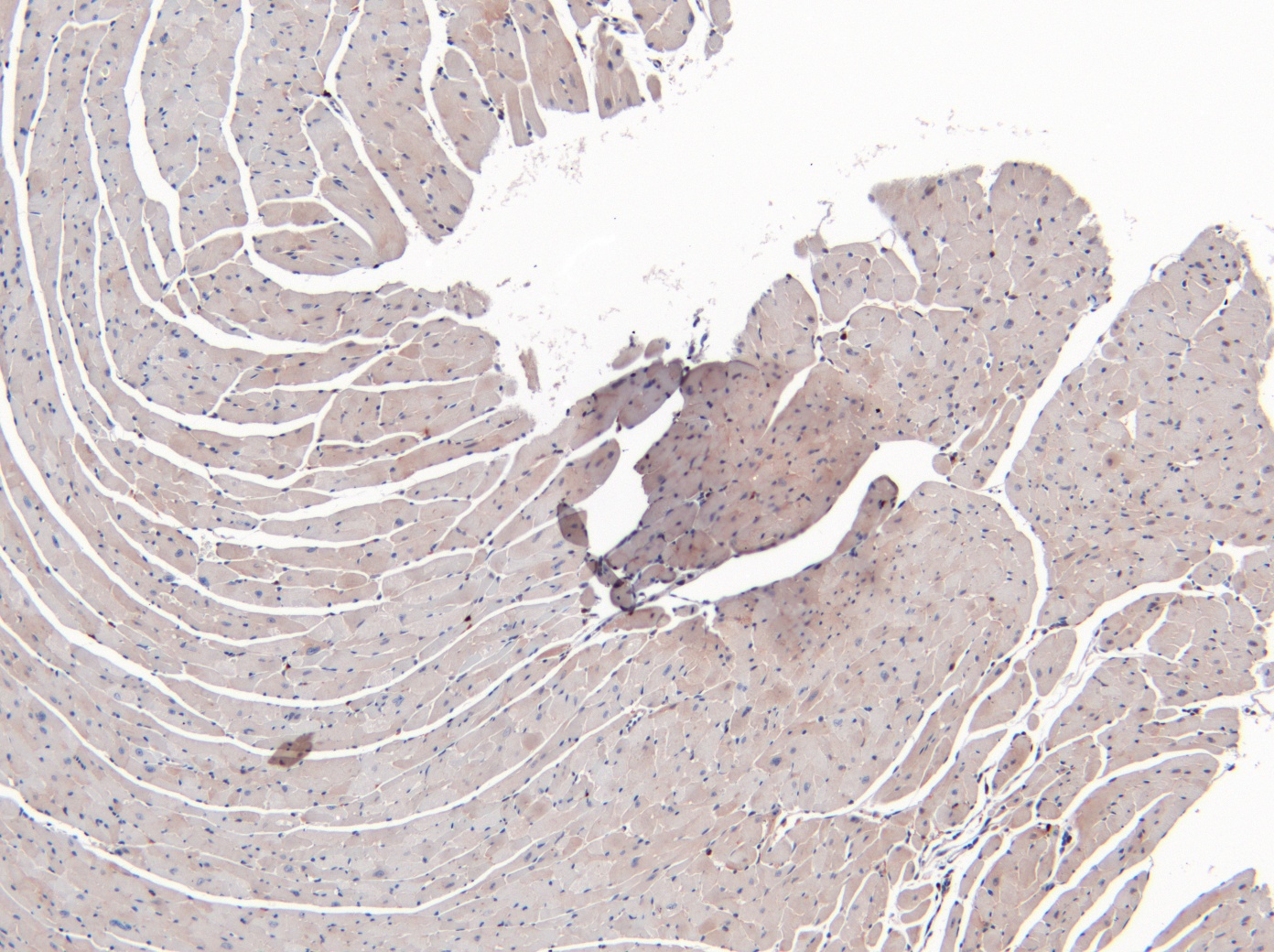

Supplement: Figure 4—source data 1. [file elife-55513-fig4-data1.zip › p16_images_for_eLife/p16_images_Ann_Chiao_for_eLife/Old Controls/OCL_3/MS_5_p16_10x_m_RGB.jpg]

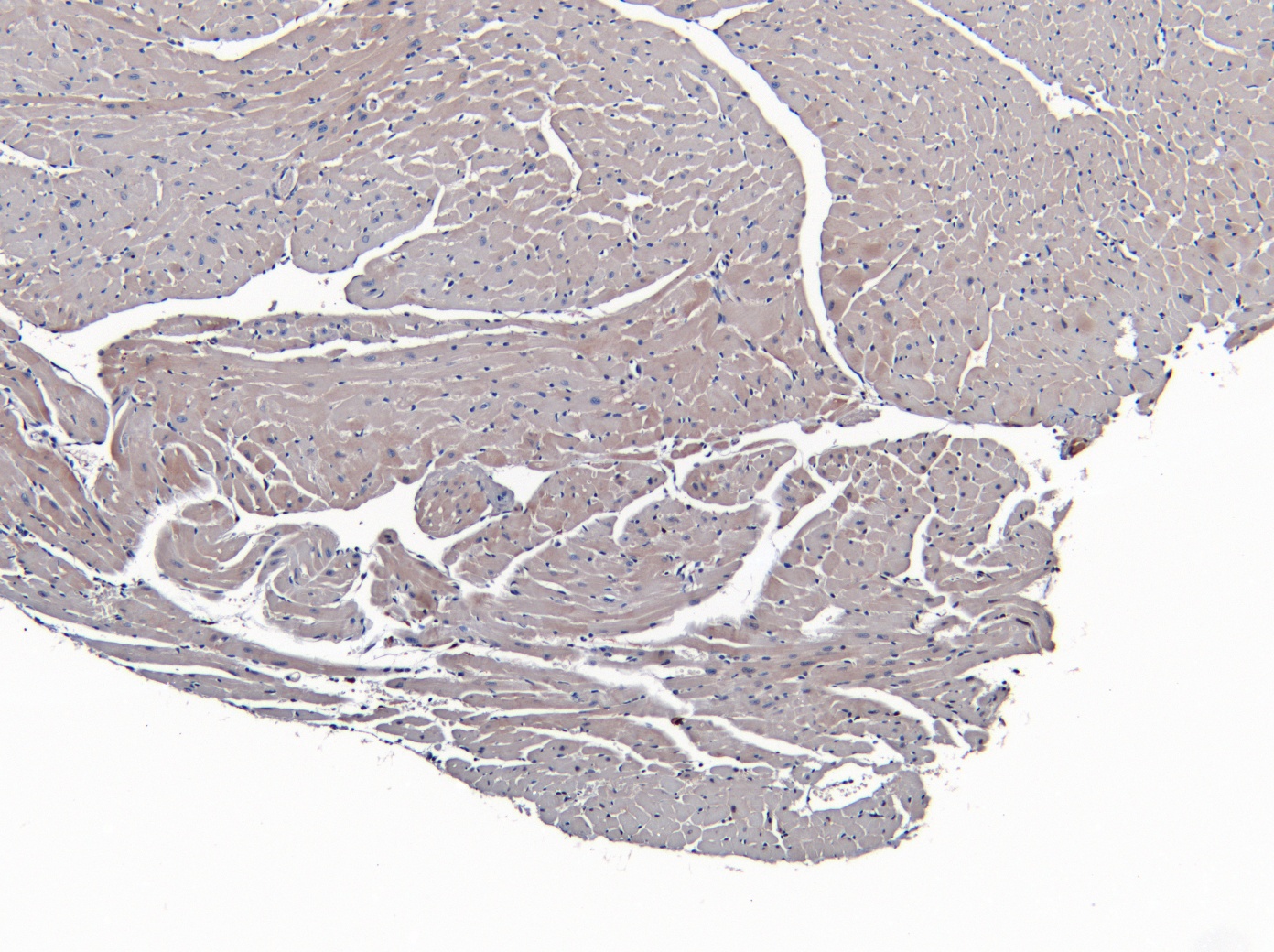

Supplement: Figure 4—source data 1. [file elife-55513-fig4-data1.zip › p16_images_for_eLife/p16_images_Ann_Chiao_for_eLife/Old Controls/OCL_4/MS_7_p16_10x_a_RGB.jpg]

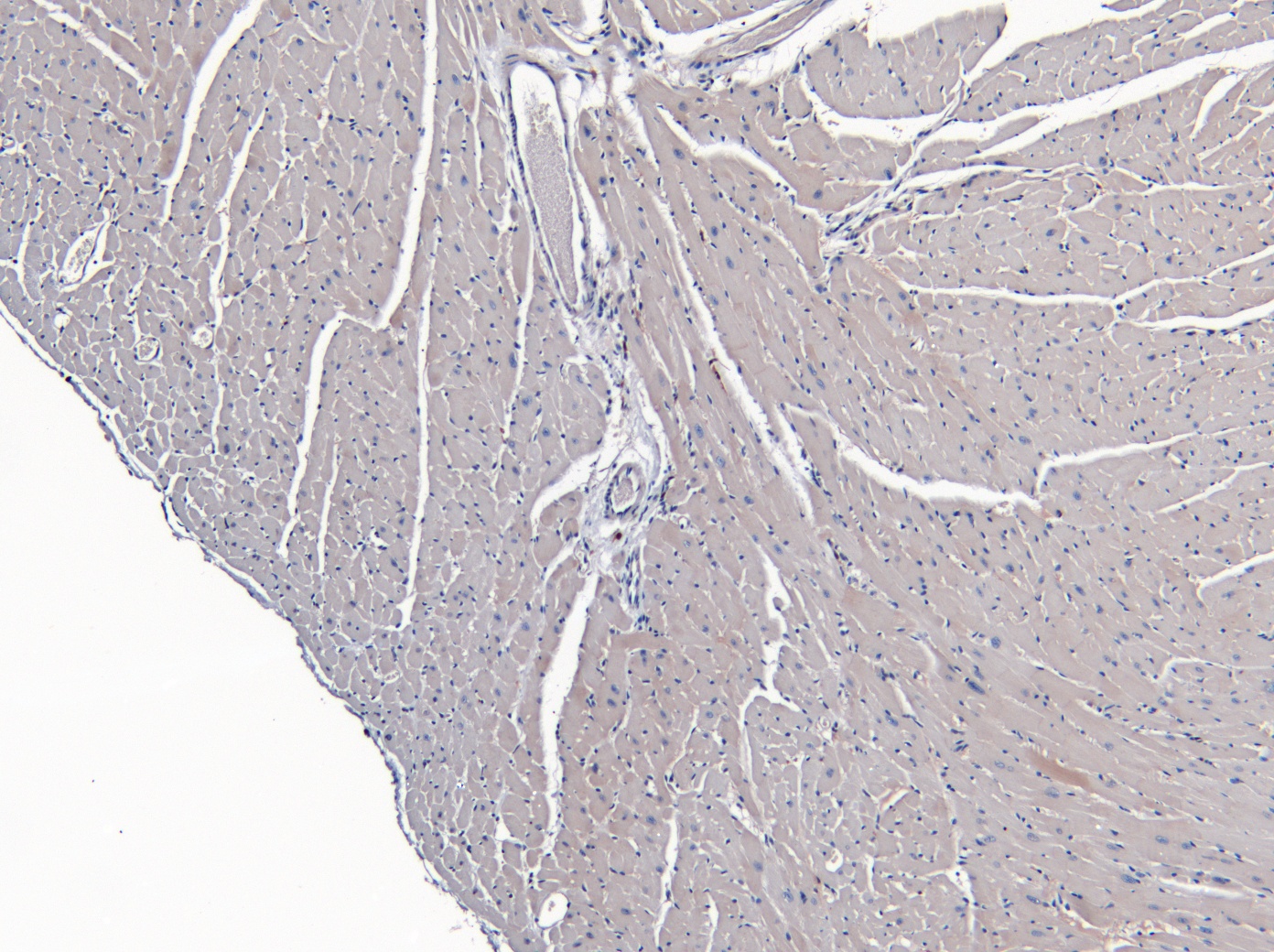

Supplement: Figure 4—source data 1. [file elife-55513-fig4-data1.zip › p16_images_for_eLife/p16_images_Ann_Chiao_for_eLife/Old Controls/OCL_4/MS_7_p16_10x_b_RGB.jpg]

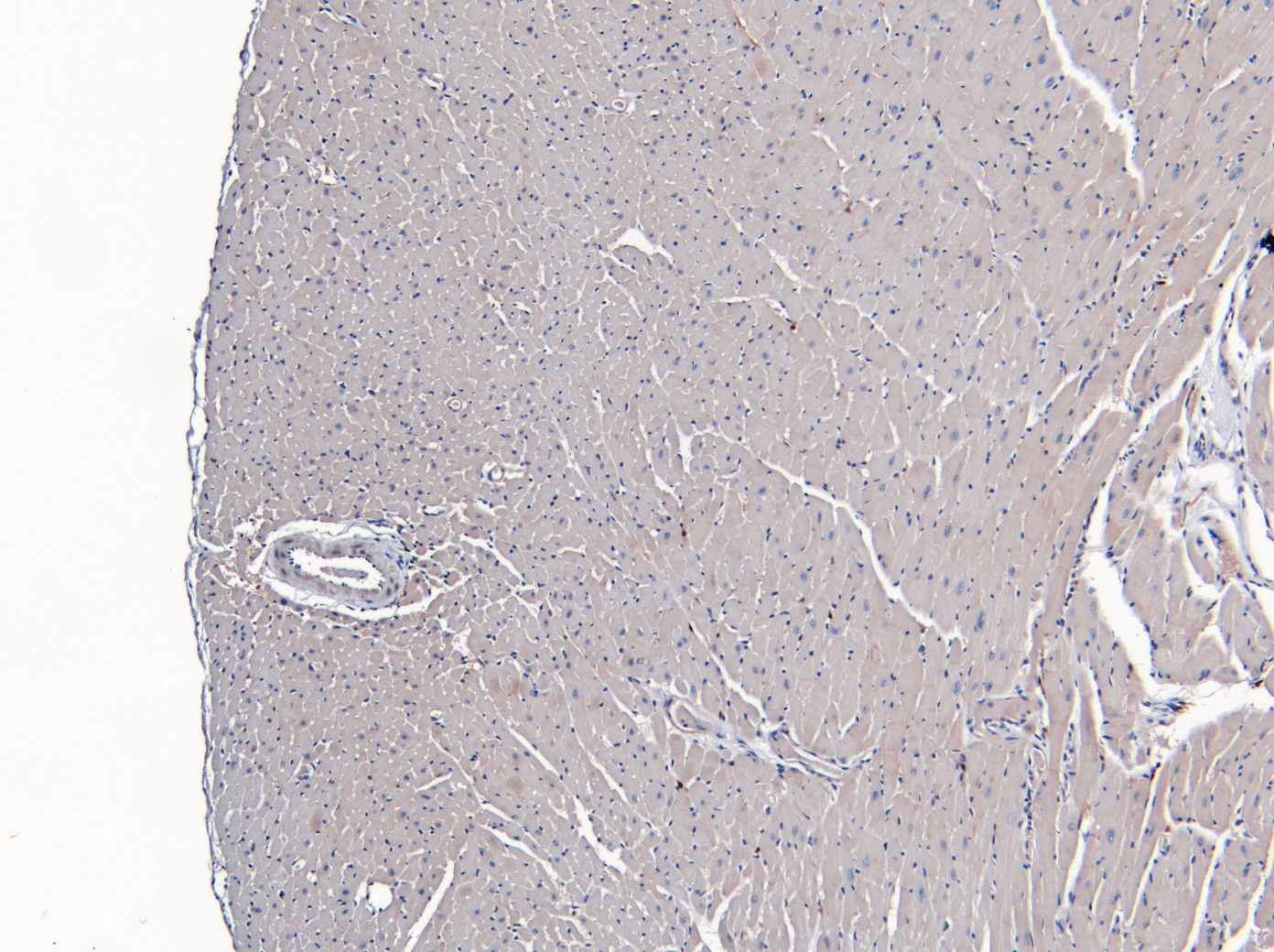

Supplement: Figure 4—source data 1. [file elife-55513-fig4-data1.zip › p16_images_for_eLife/p16_images_Ann_Chiao_for_eLife/Old Controls/OCL_4/MS_7_p16_10x_c_RGB.jpg]

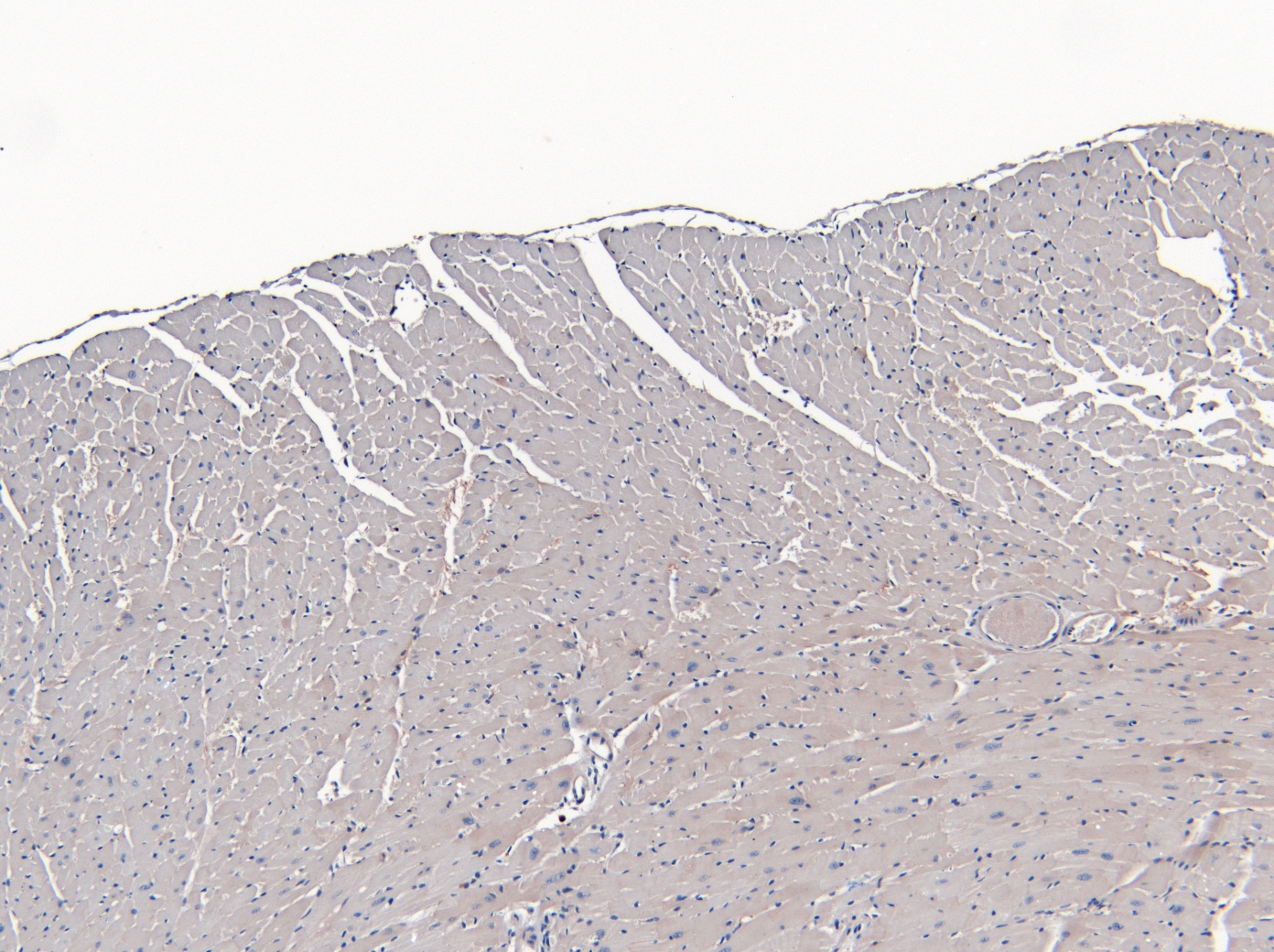

Supplement: Figure 4—source data 1. [file elife-55513-fig4-data1.zip › p16_images_for_eLife/p16_images_Ann_Chiao_for_eLife/Old Controls/OCL_4/MS_7_p16_10x_d_RGB.jpg]

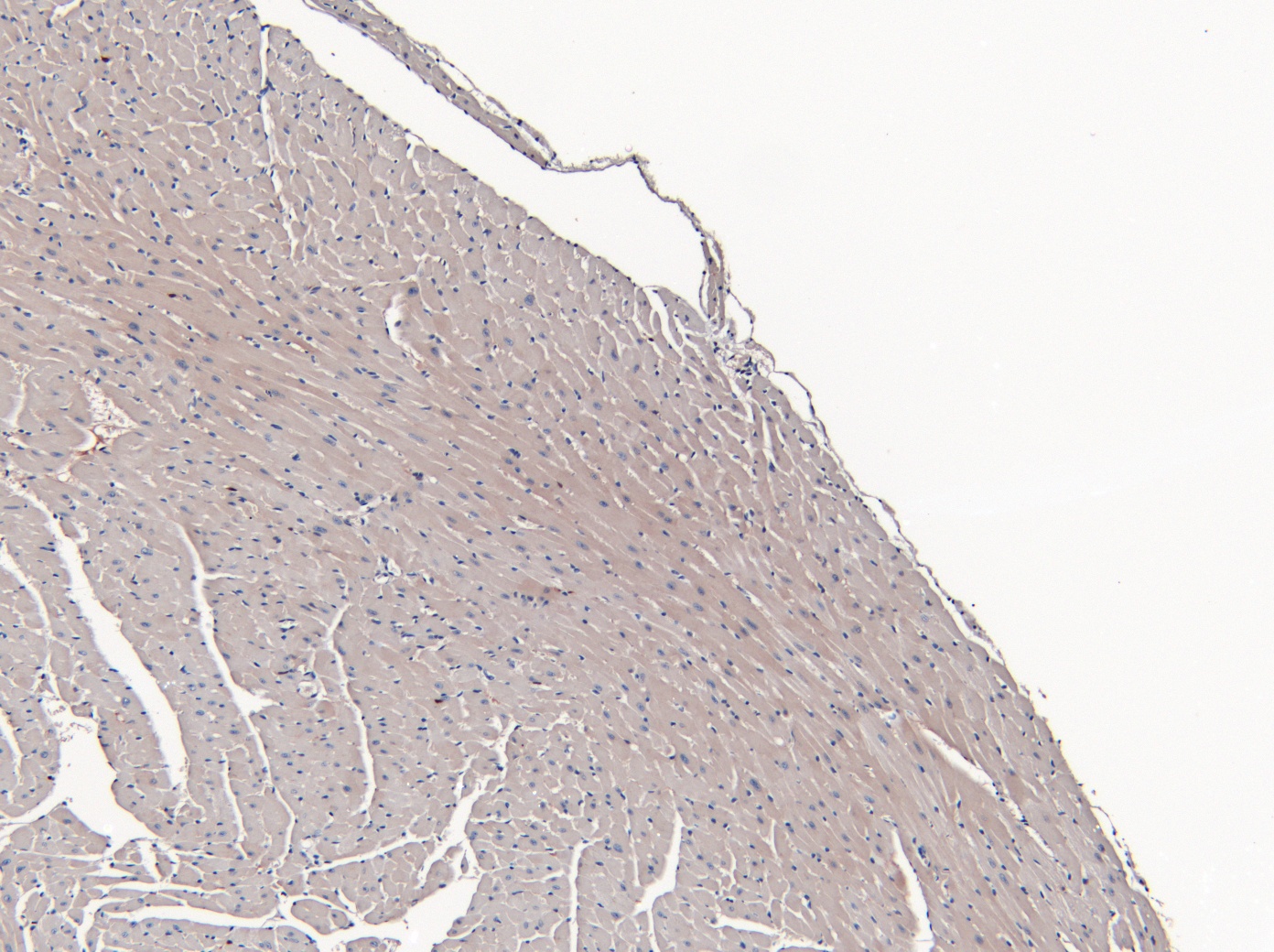

Supplement: Figure 4—source data 1. [file elife-55513-fig4-data1.zip › p16_images_for_eLife/p16_images_Ann_Chiao_for_eLife/Old Controls/OCL_4/MS_7_p16_10x_e_RGB.jpg]

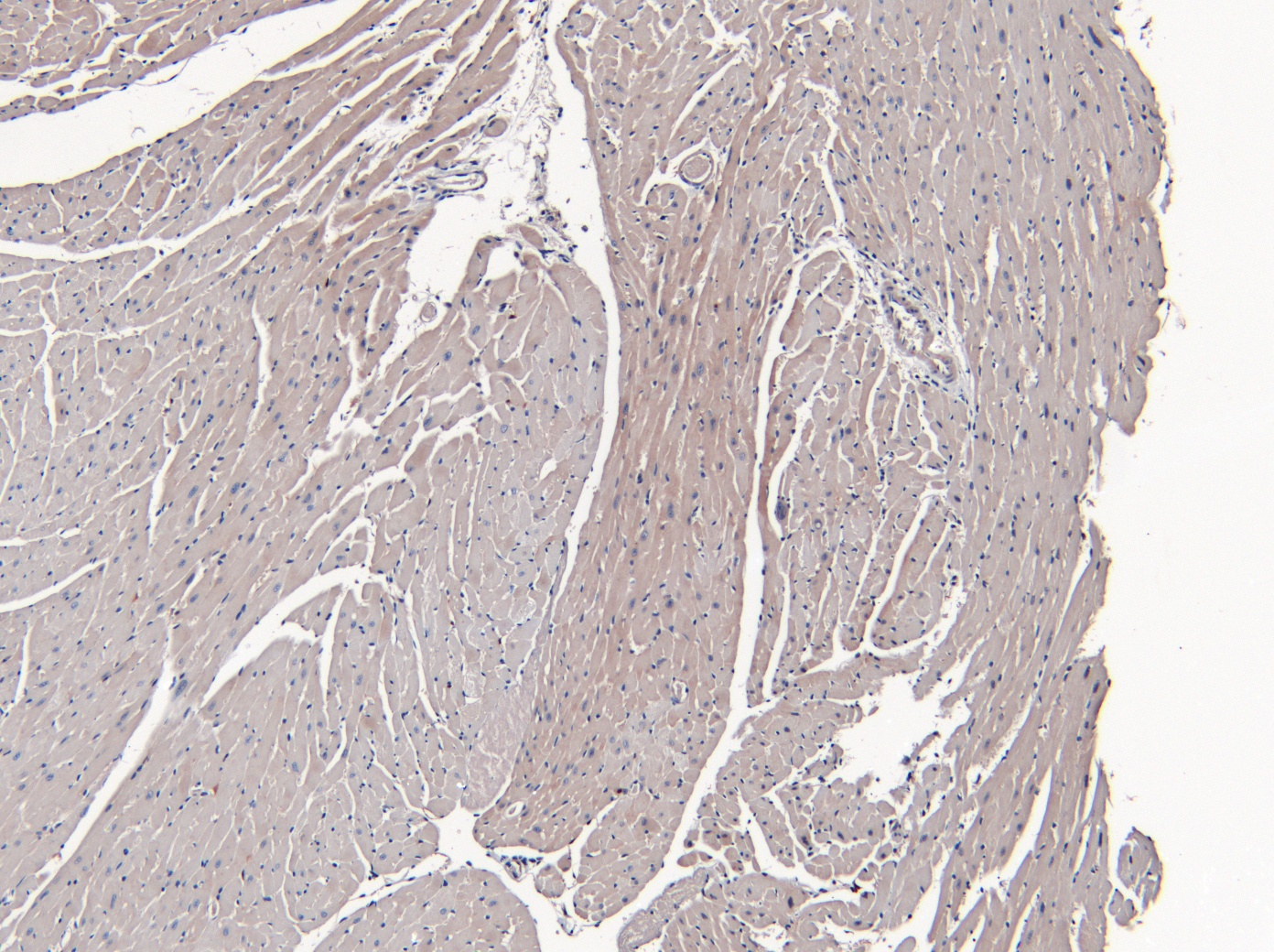

Supplement: Figure 4—source data 1. [file elife-55513-fig4-data1.zip › p16_images_for_eLife/p16_images_Ann_Chiao_for_eLife/Old Controls/OCL_4/MS_7_p16_10x_f_RGB.jpg]

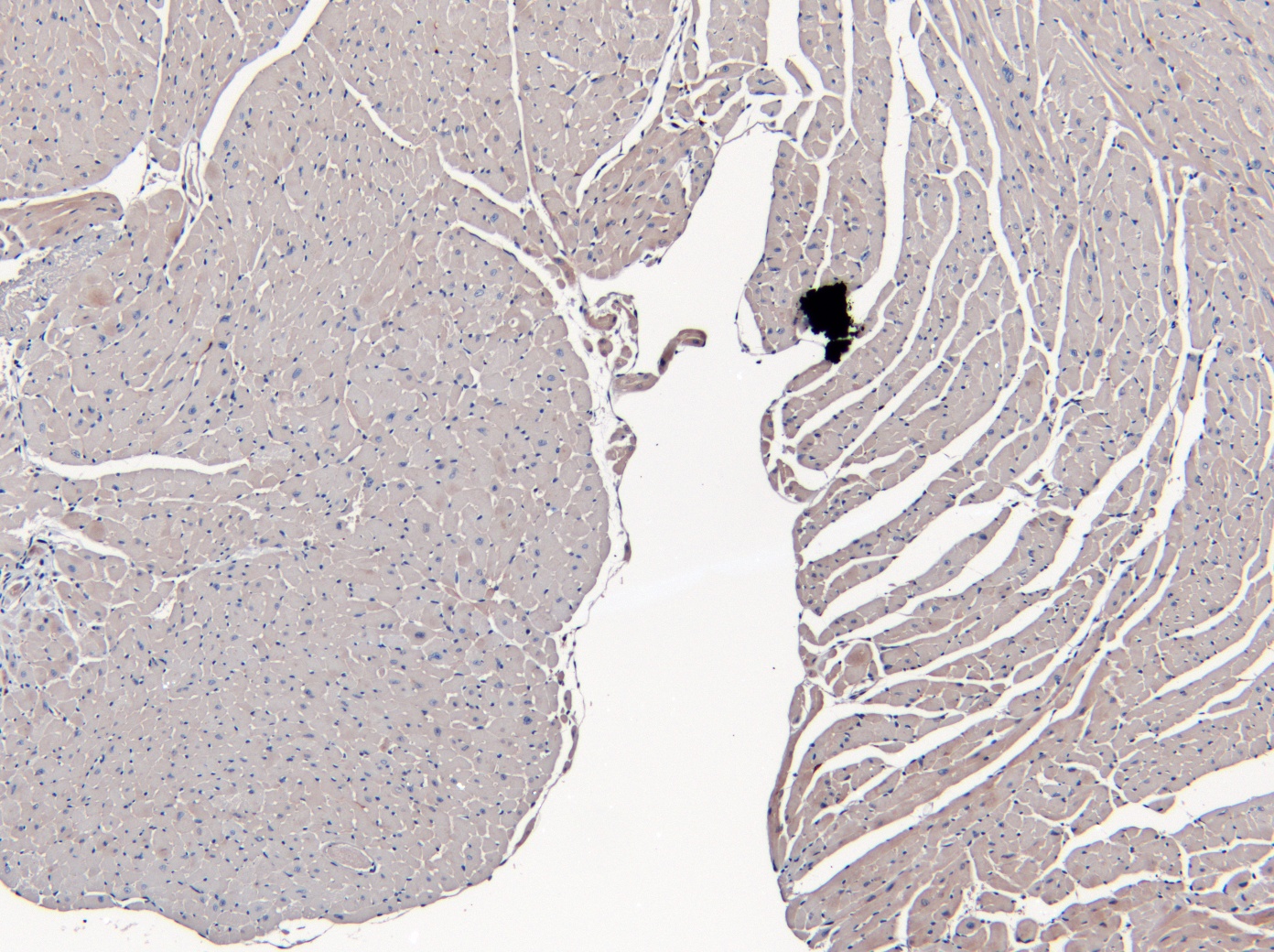

Supplement: Figure 4—source data 1. [file elife-55513-fig4-data1.zip › p16_images_for_eLife/p16_images_Ann_Chiao_for_eLife/Old Controls/OCL_4/MS_7_p16_10x_g_RGB.jpg]

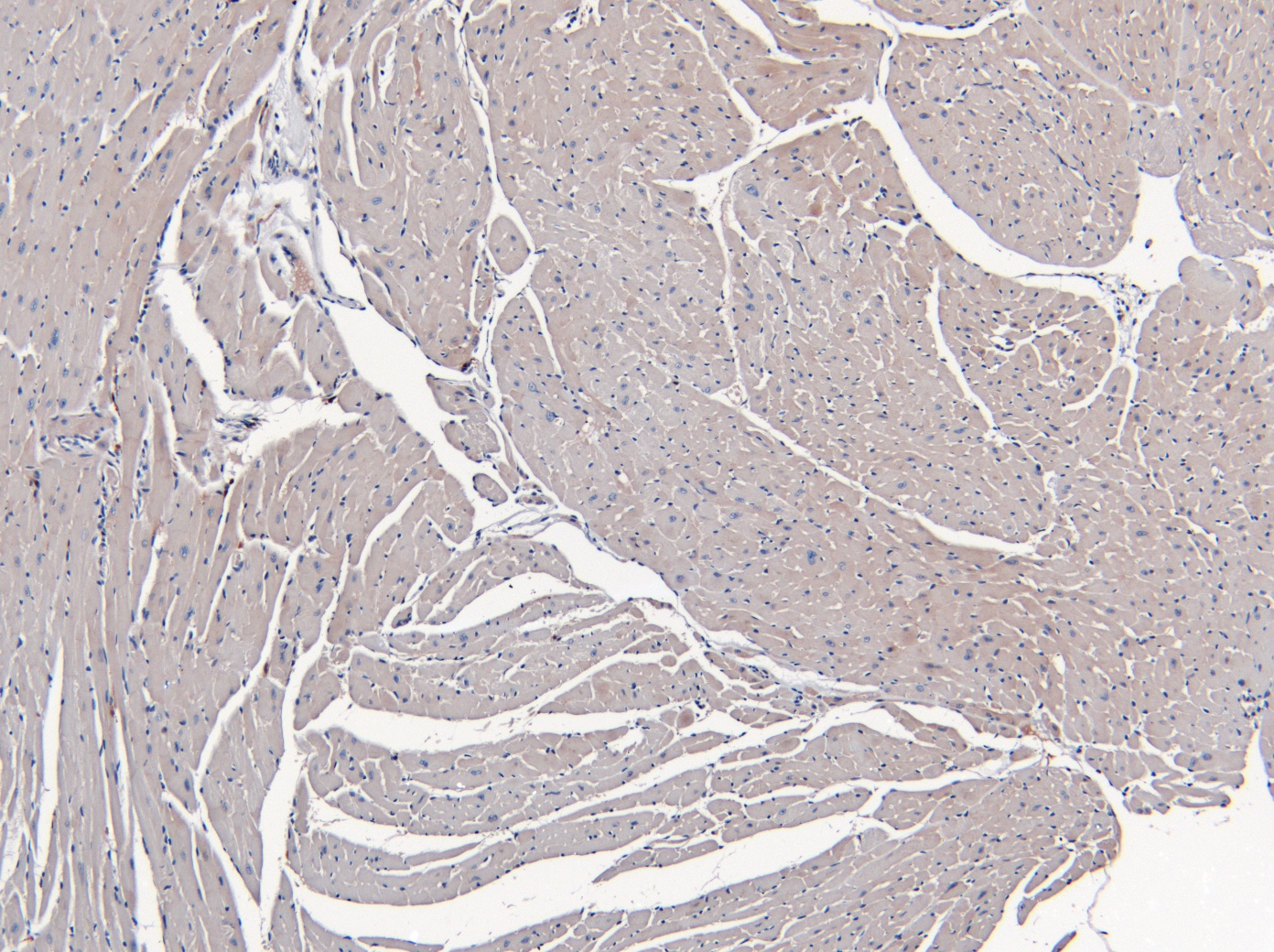

Supplement: Figure 4—source data 1. [file elife-55513-fig4-data1.zip › p16_images_for_eLife/p16_images_Ann_Chiao_for_eLife/Old Controls/OCL_4/MS_7_p16_10x_h_RGB.jpg]

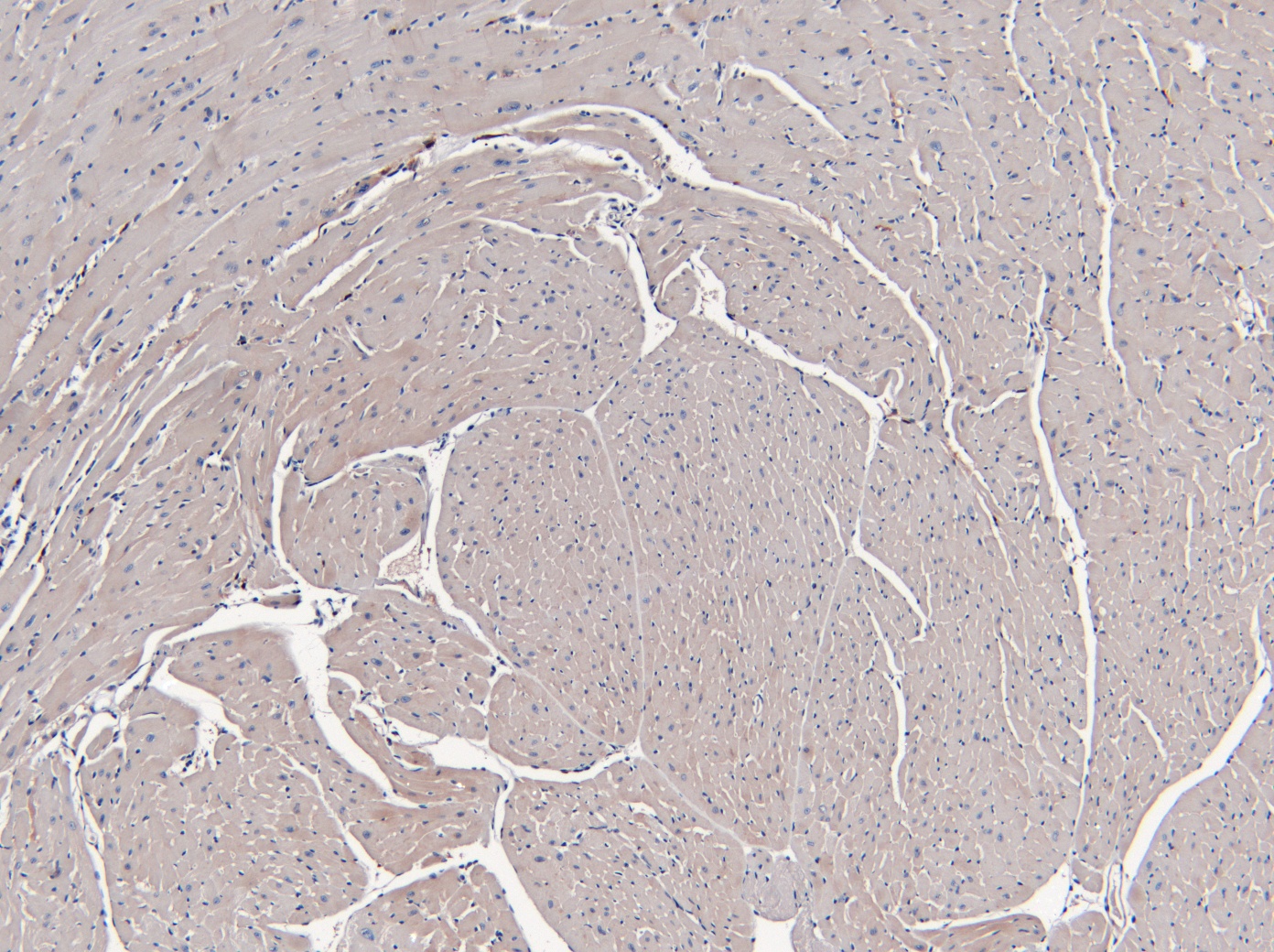

Supplement: Figure 4—source data 1. [file elife-55513-fig4-data1.zip › p16_images_for_eLife/p16_images_Ann_Chiao_for_eLife/Old Controls/OCL_4/MS_7_p16_10x_i_RGB.jpg]

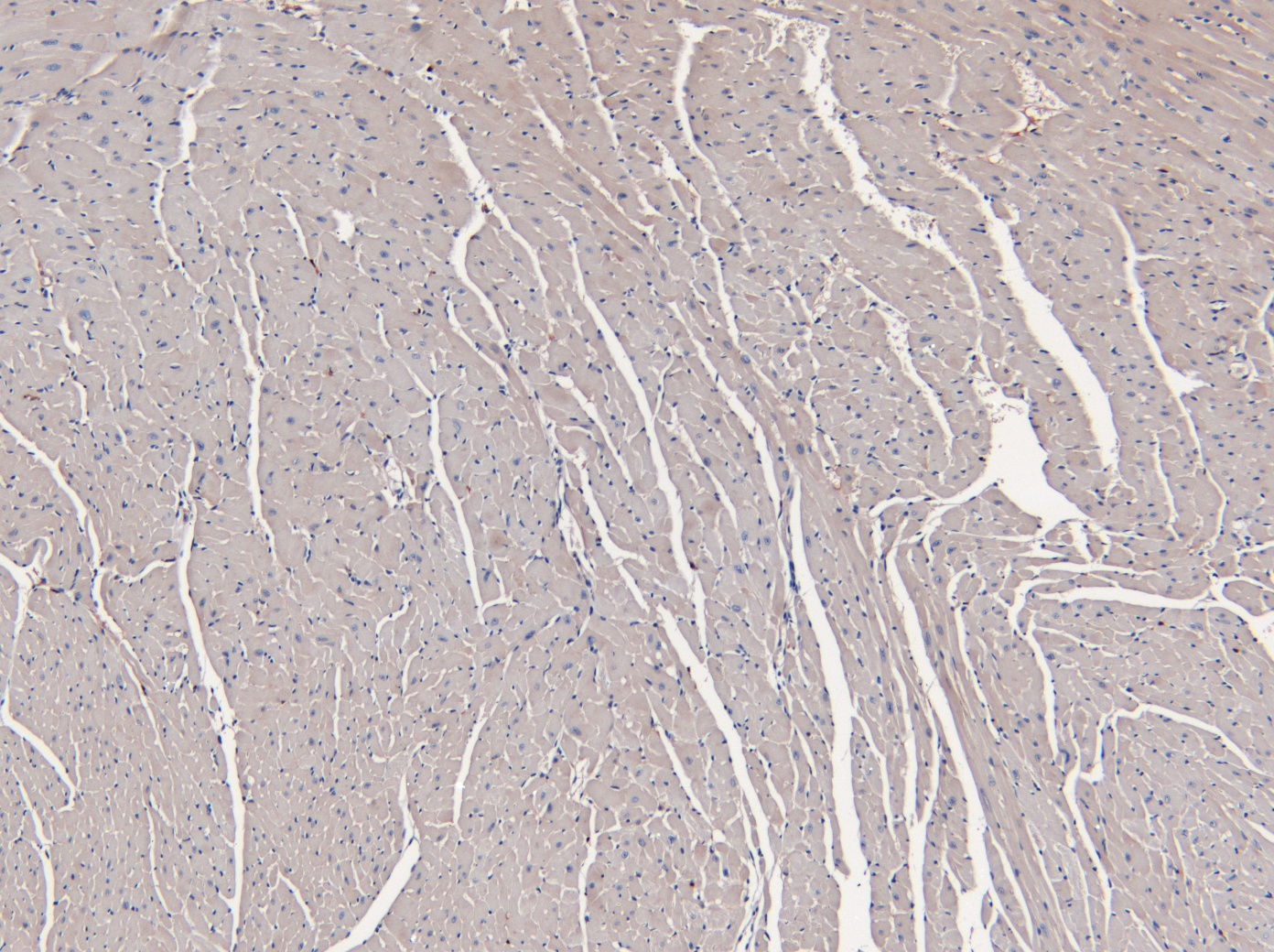

Supplement: Figure 4—source data 1. [file elife-55513-fig4-data1.zip › p16_images_for_eLife/p16_images_Ann_Chiao_for_eLife/Old Controls/OCL_4/MS_7_p16_10x_j_RGB.jpg]

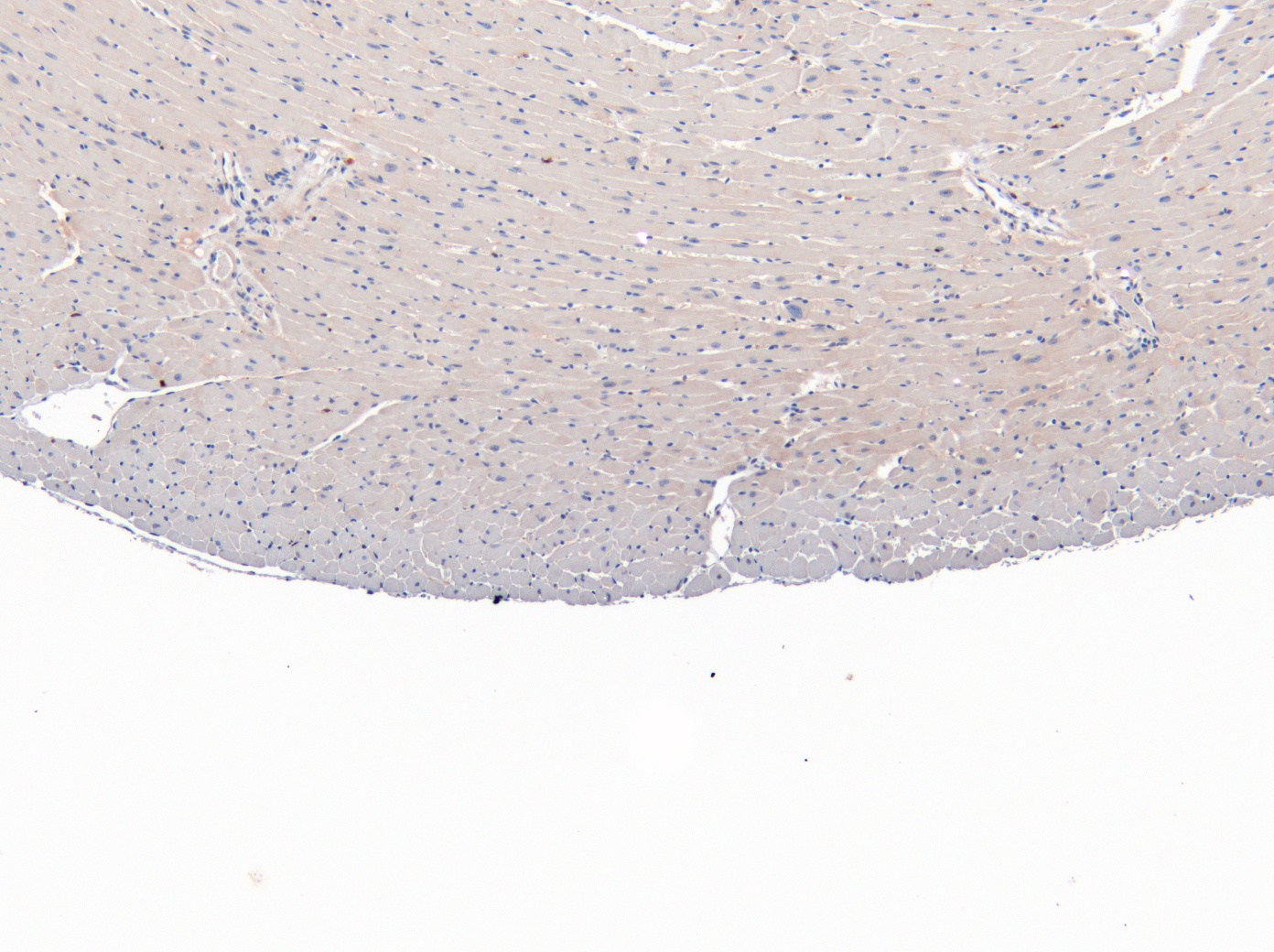

Supplement: Figure 4—source data 1. [file elife-55513-fig4-data1.zip › p16_images_for_eLife/p16_images_Ann_Chiao_for_eLife/Old Controls/OCL_5/MS_10_p16_10x_a_RGB.jpg]

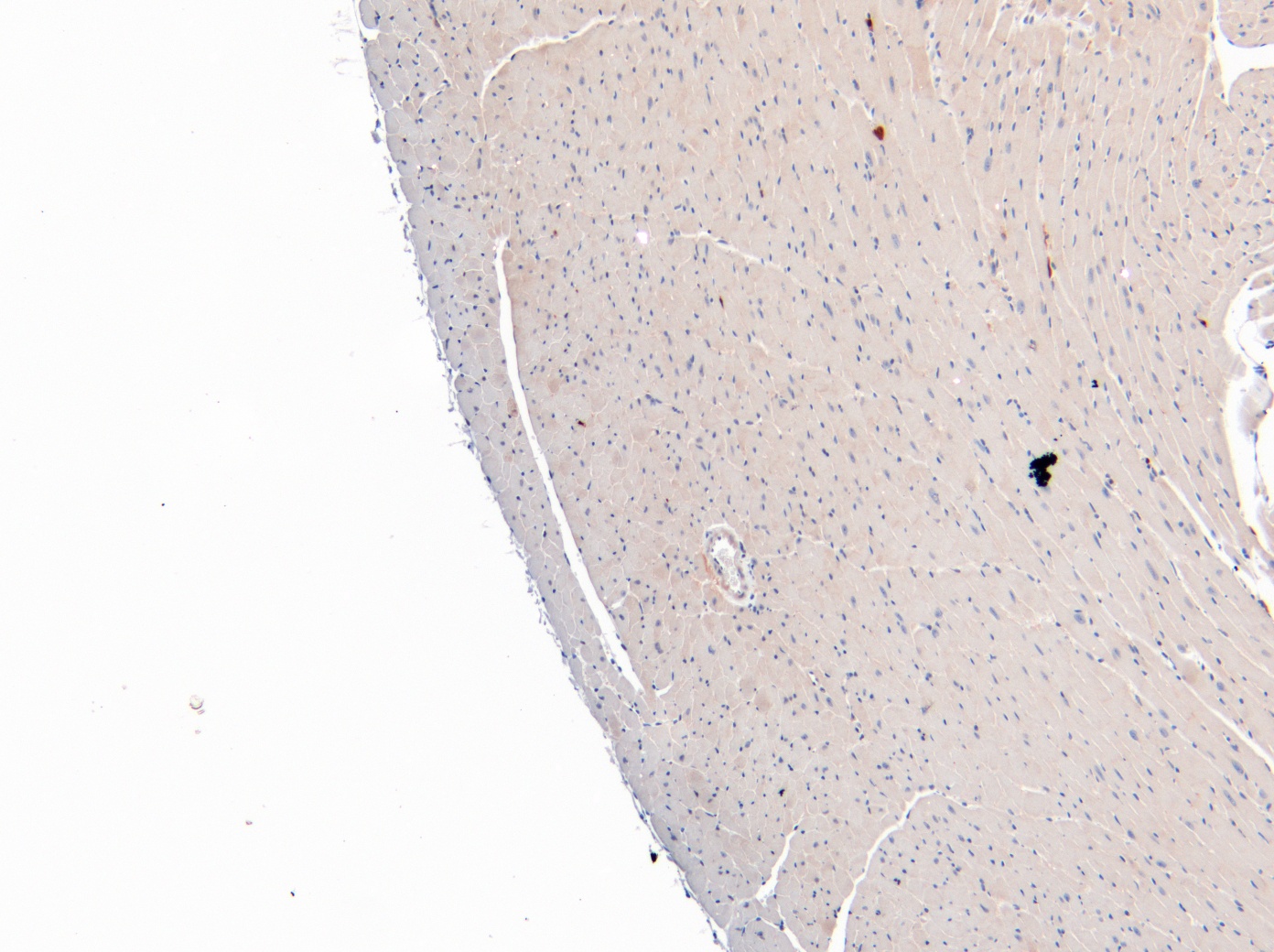

Supplement: Figure 4—source data 1. [file elife-55513-fig4-data1.zip › p16_images_for_eLife/p16_images_Ann_Chiao_for_eLife/Old Controls/OCL_5/MS_10_p16_10x_b_RGB.jpg]

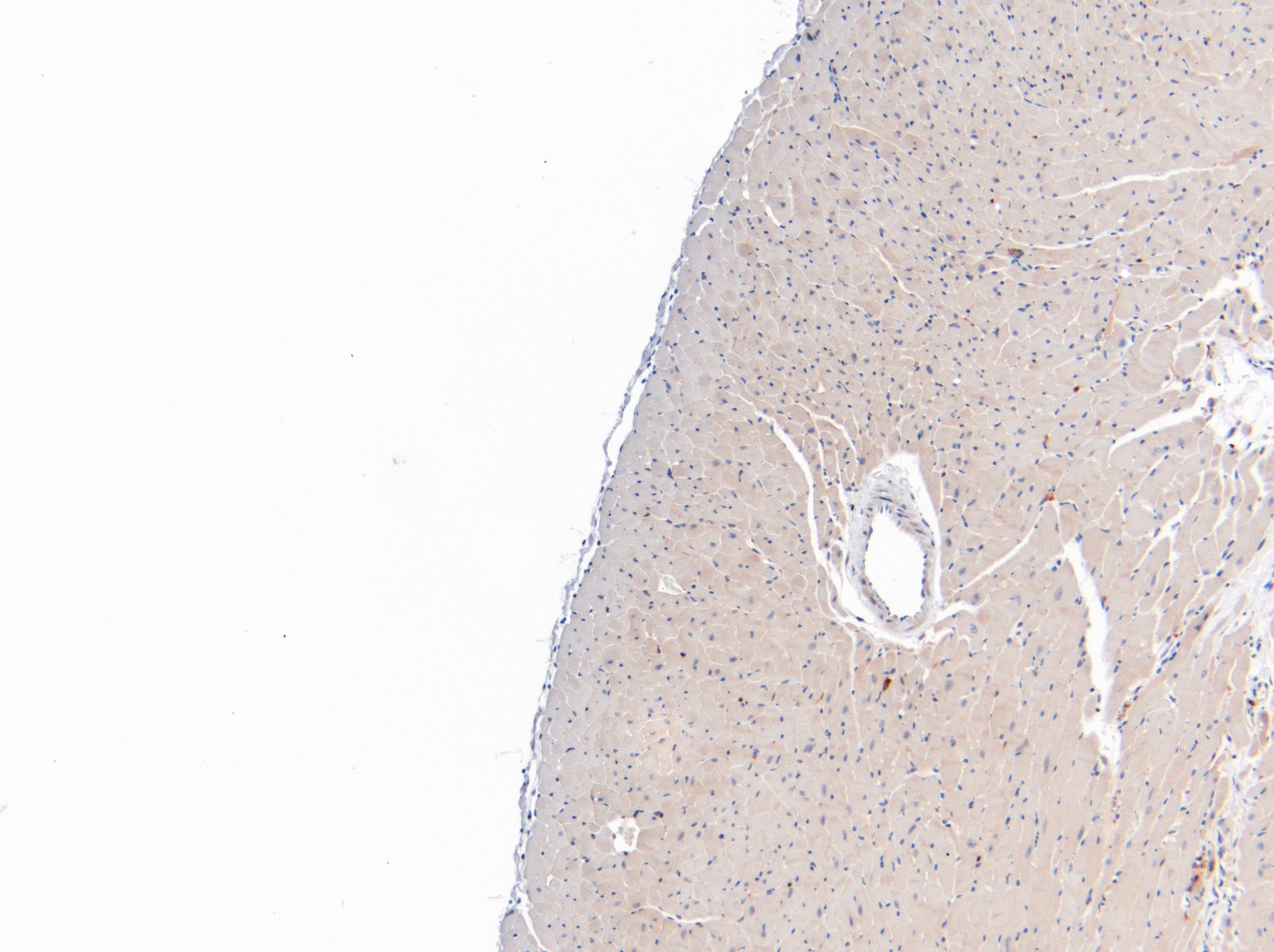

Supplement: Figure 4—source data 1. [file elife-55513-fig4-data1.zip › p16_images_for_eLife/p16_images_Ann_Chiao_for_eLife/Old Controls/OCL_5/MS_10_p16_10x_c_RGB.jpg]

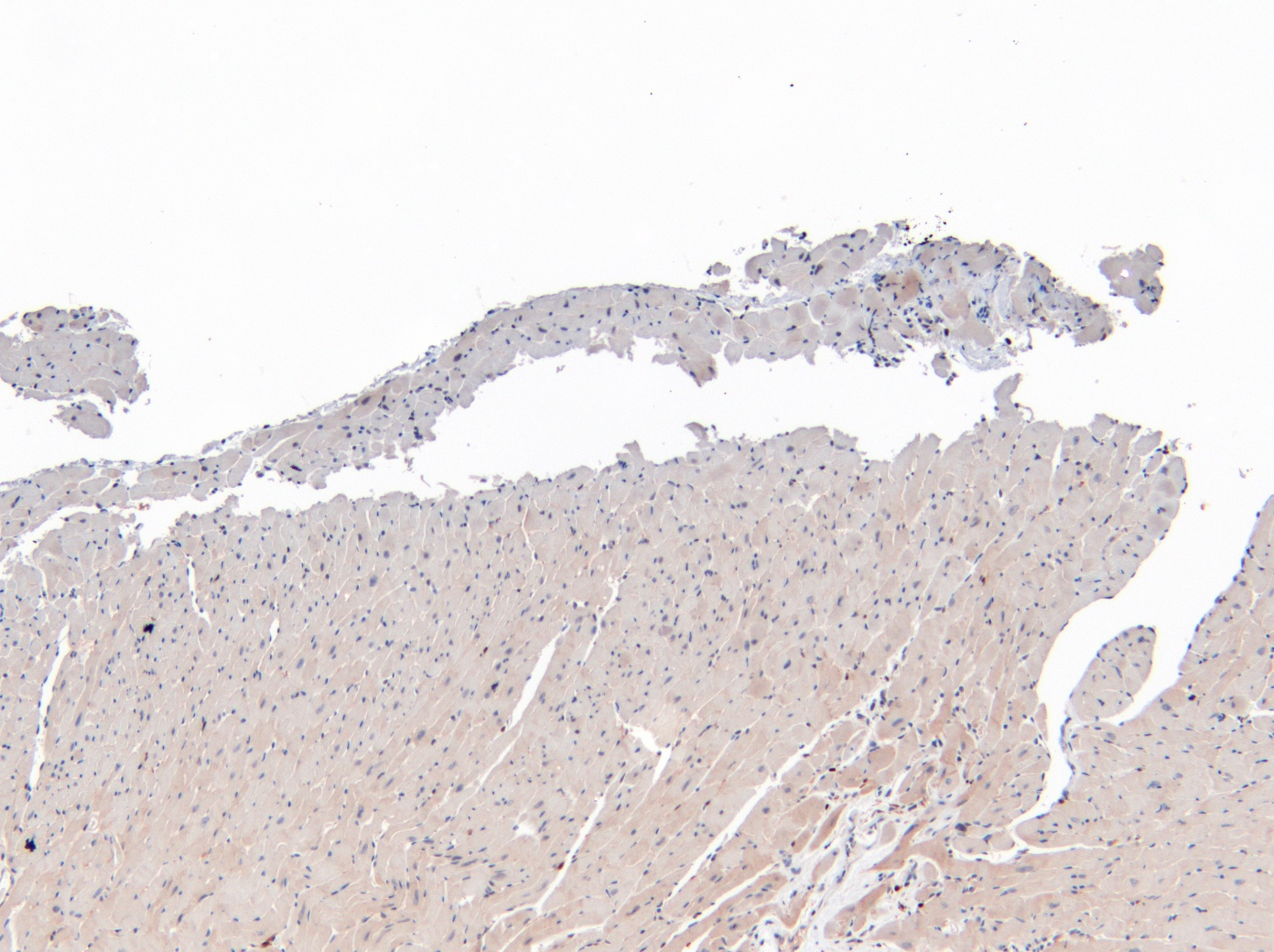

Supplement: Figure 4—source data 1. [file elife-55513-fig4-data1.zip › p16_images_for_eLife/p16_images_Ann_Chiao_for_eLife/Old Controls/OCL_5/MS_10_p16_10x_d_RGB (1).jpg]

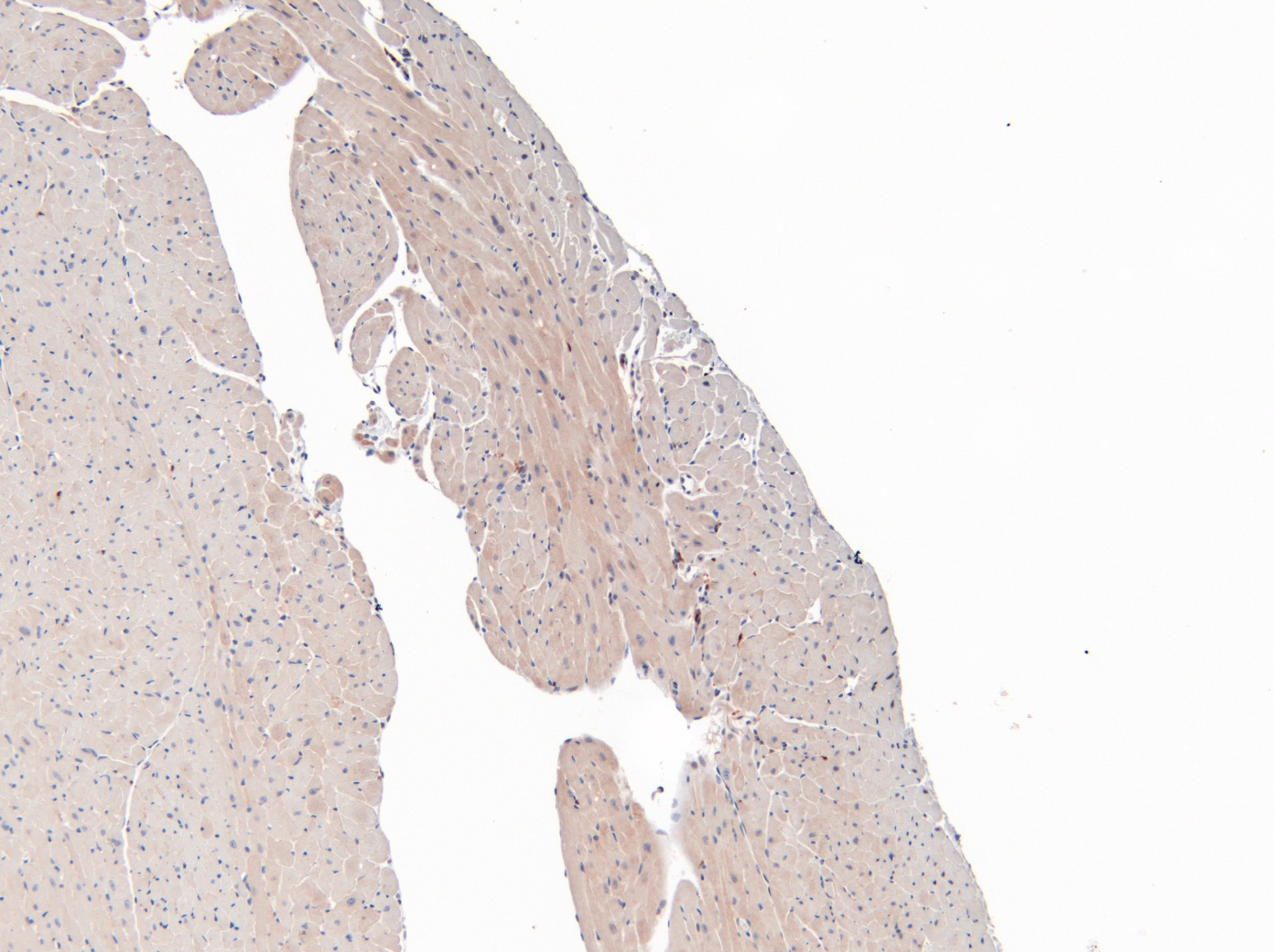

Supplement: Figure 4—source data 1. [file elife-55513-fig4-data1.zip › p16_images_for_eLife/p16_images_Ann_Chiao_for_eLife/Old Controls/OCL_5/MS_10_p16_10x_e_RGB (1).jpg]

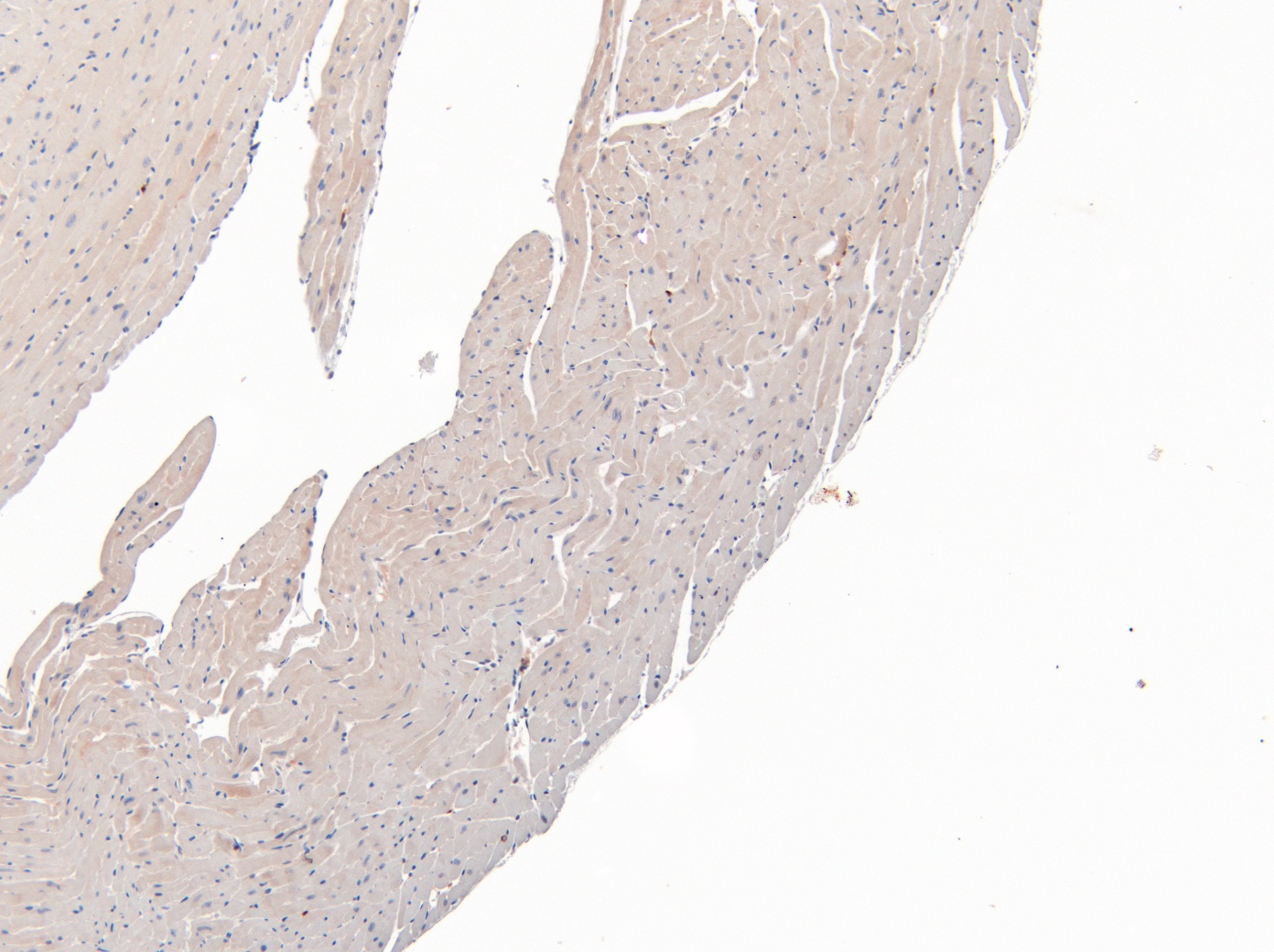

Supplement: Figure 4—source data 1. [file elife-55513-fig4-data1.zip › p16_images_for_eLife/p16_images_Ann_Chiao_for_eLife/Old Controls/OCL_5/MS_10_p16_10x_f_RGB.jpg]

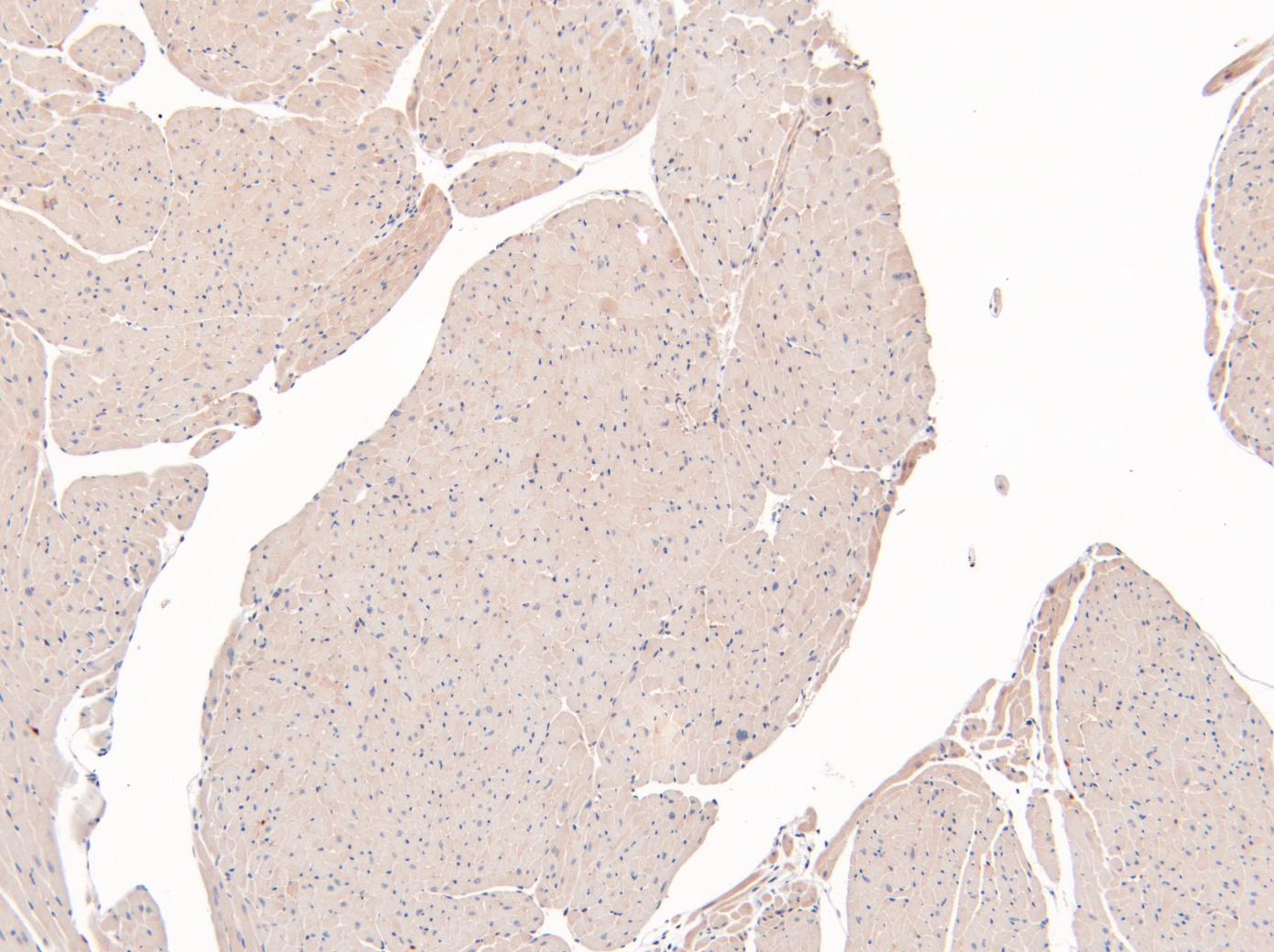

Supplement: Figure 4—source data 1. [file elife-55513-fig4-data1.zip › p16_images_for_eLife/p16_images_Ann_Chiao_for_eLife/Old Controls/OCL_5/MS_10_p16_10x_g_RGB.jpg]

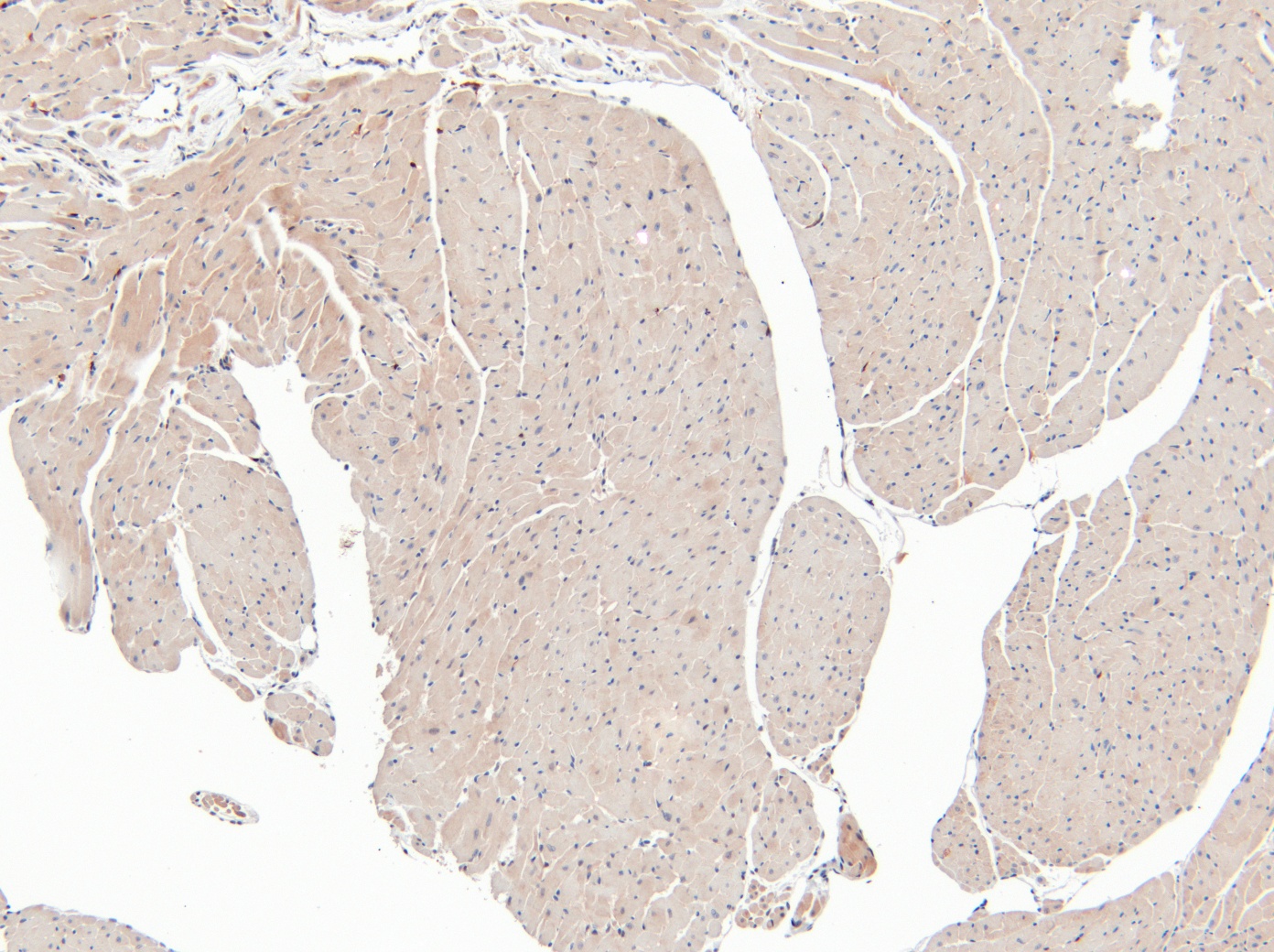

Supplement: Figure 4—source data 1. [file elife-55513-fig4-data1.zip › p16_images_for_eLife/p16_images_Ann_Chiao_for_eLife/Old Controls/OCL_5/MS_10_p16_10x_h_RGB.jpg]

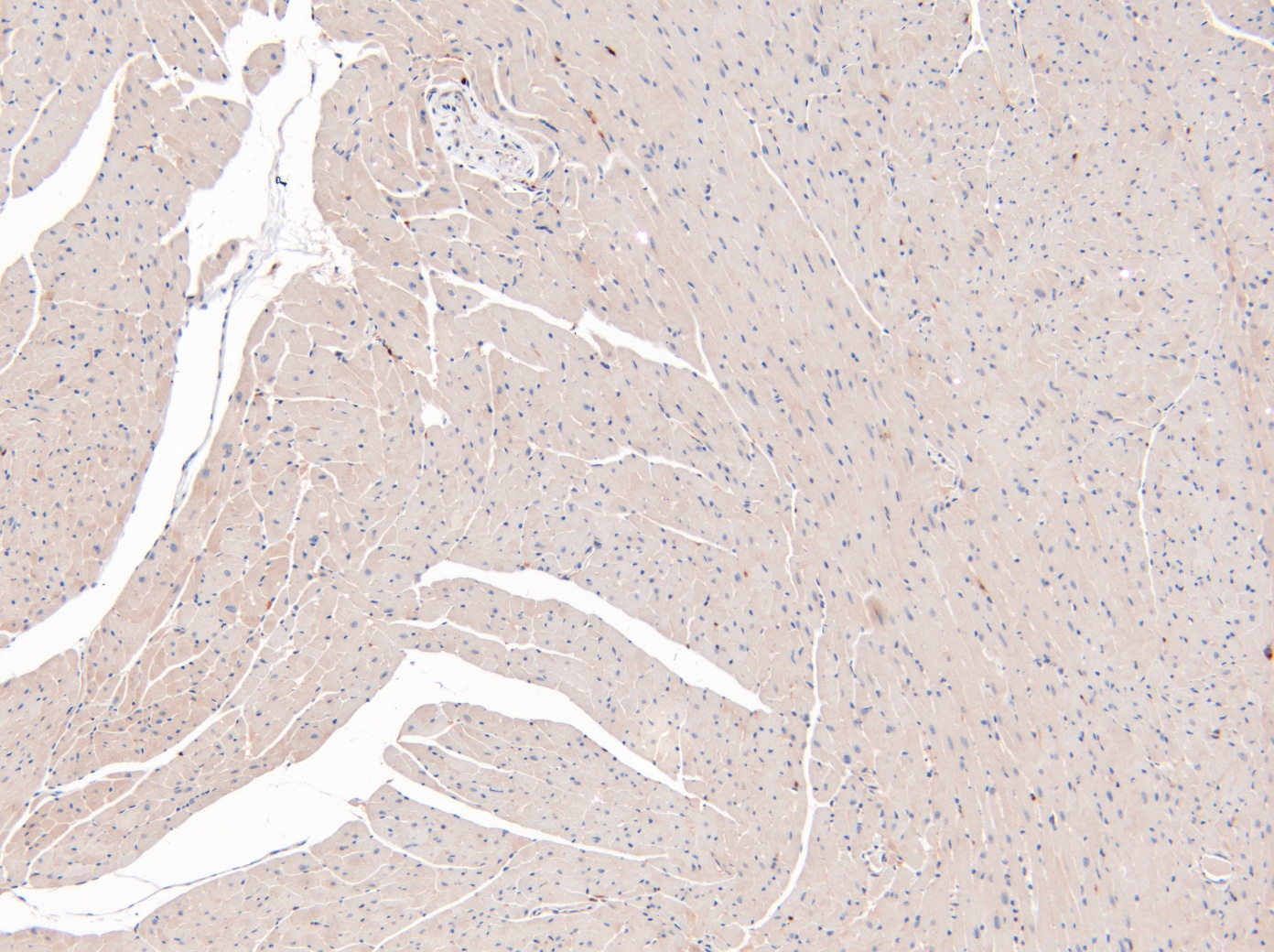

Supplement: Figure 4—source data 1. [file elife-55513-fig4-data1.zip › p16_images_for_eLife/p16_images_Ann_Chiao_for_eLife/Old Controls/OCL_5/MS_10_p16_10x_i_RGB.jpg]

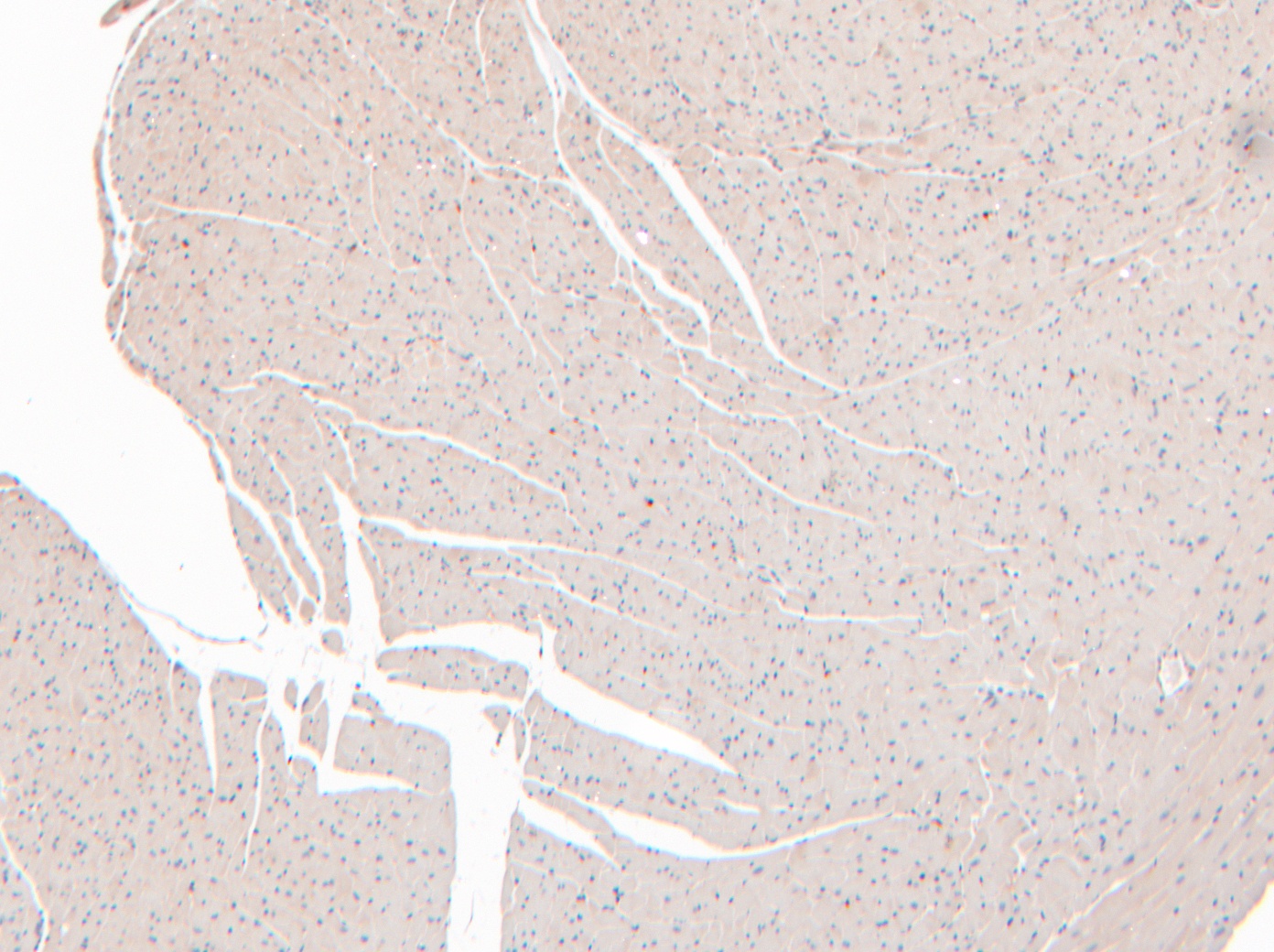

Supplement: Figure 4—source data 1. [file elife-55513-fig4-data1.zip › p16_images_for_eLife/p16_images_Ann_Chiao_for_eLife/Old Controls/OCL_5/MS_10_p16_10x_j_RGB.jpg]

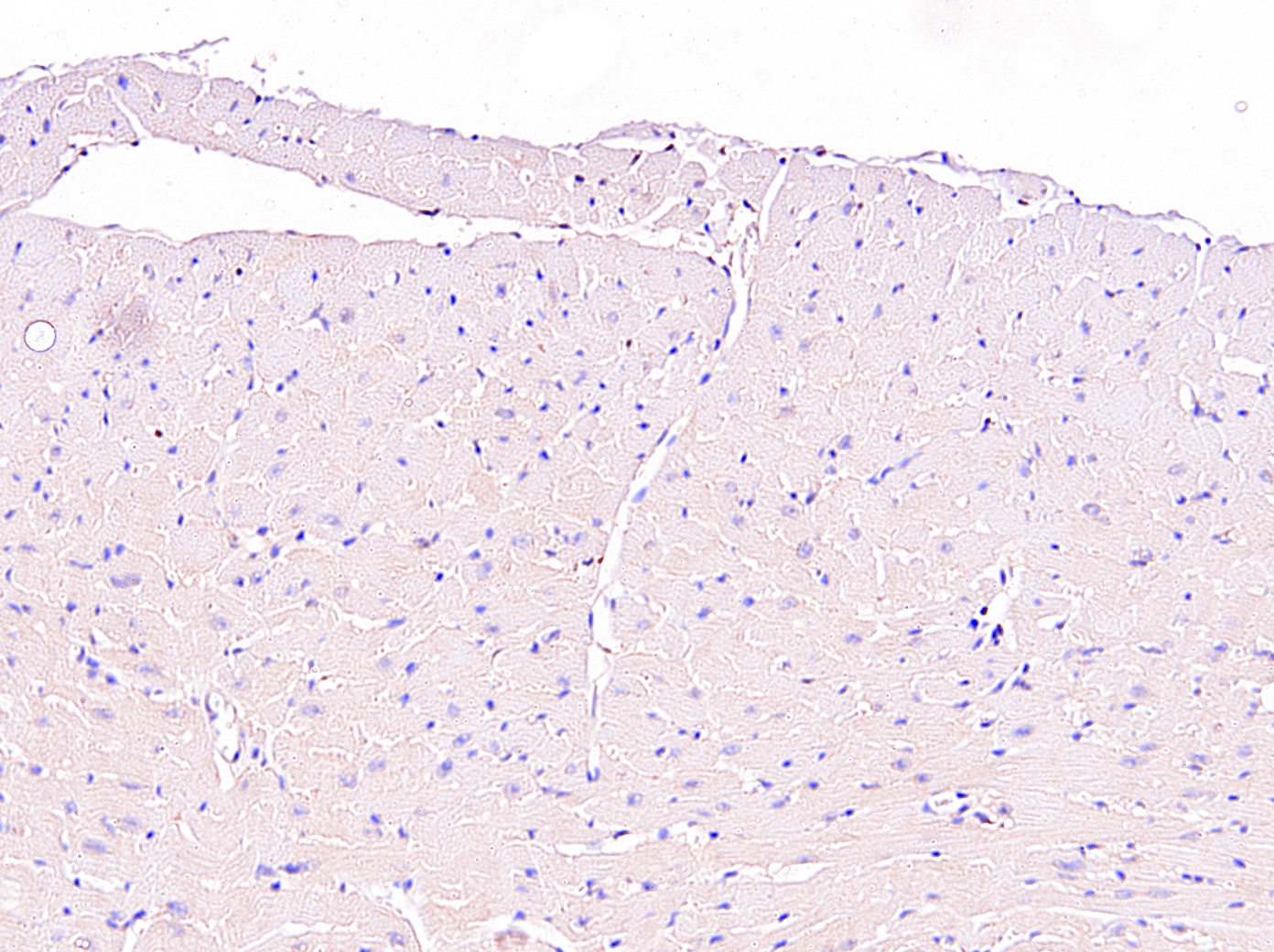

Supplement: Figure 4—source data 1. [file elife-55513-fig4-data1.zip › p16_images_for_eLife/p16_images_Ann_Chiao_for_eLife/Old Controls/OCL_6/2.1_a_unmixed_comp.jpg]

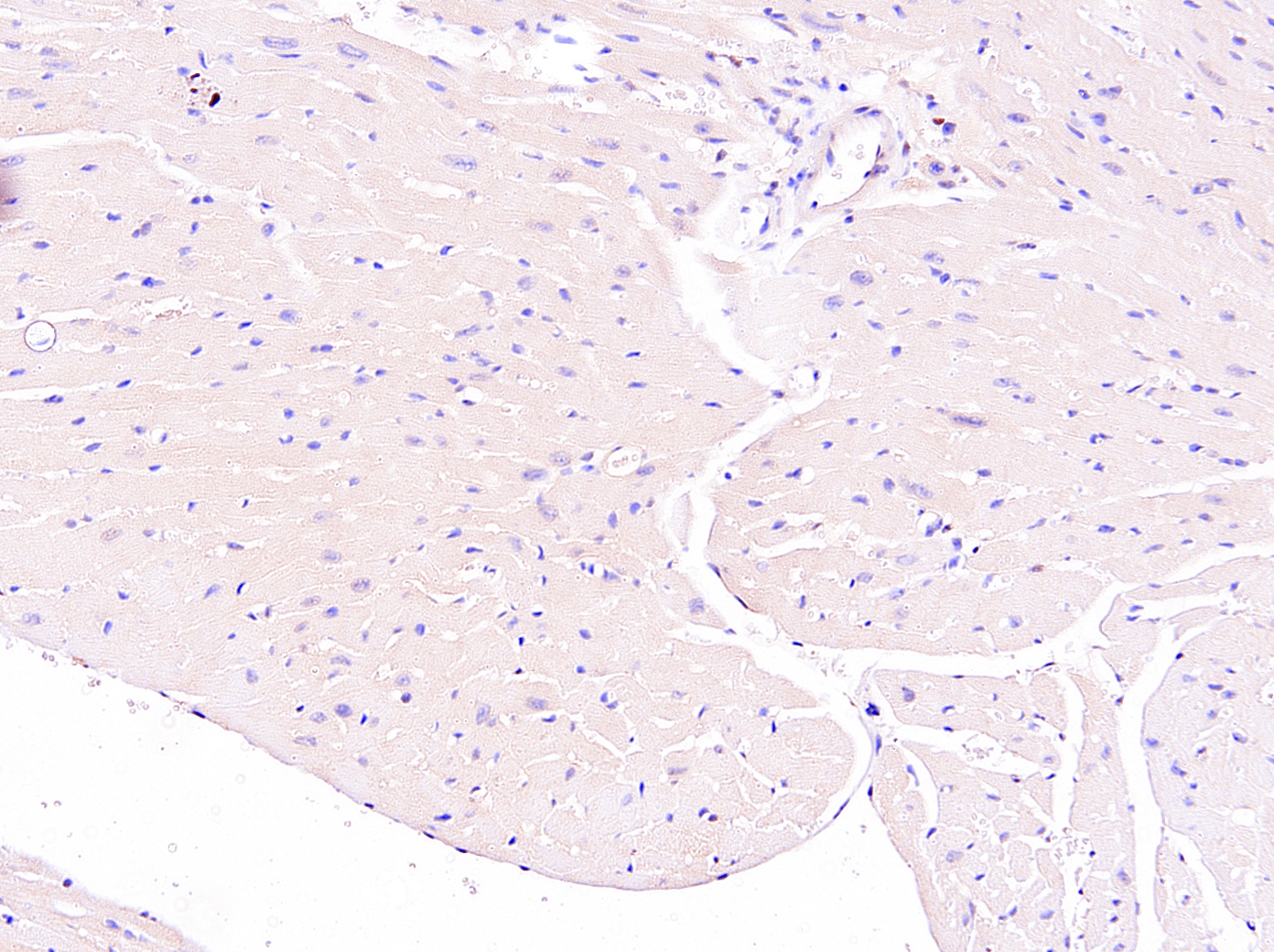

Supplement: Figure 4—source data 1. [file elife-55513-fig4-data1.zip › p16_images_for_eLife/p16_images_Ann_Chiao_for_eLife/Old Controls/OCL_6/2.1_b_unmixed_comp.jpg]

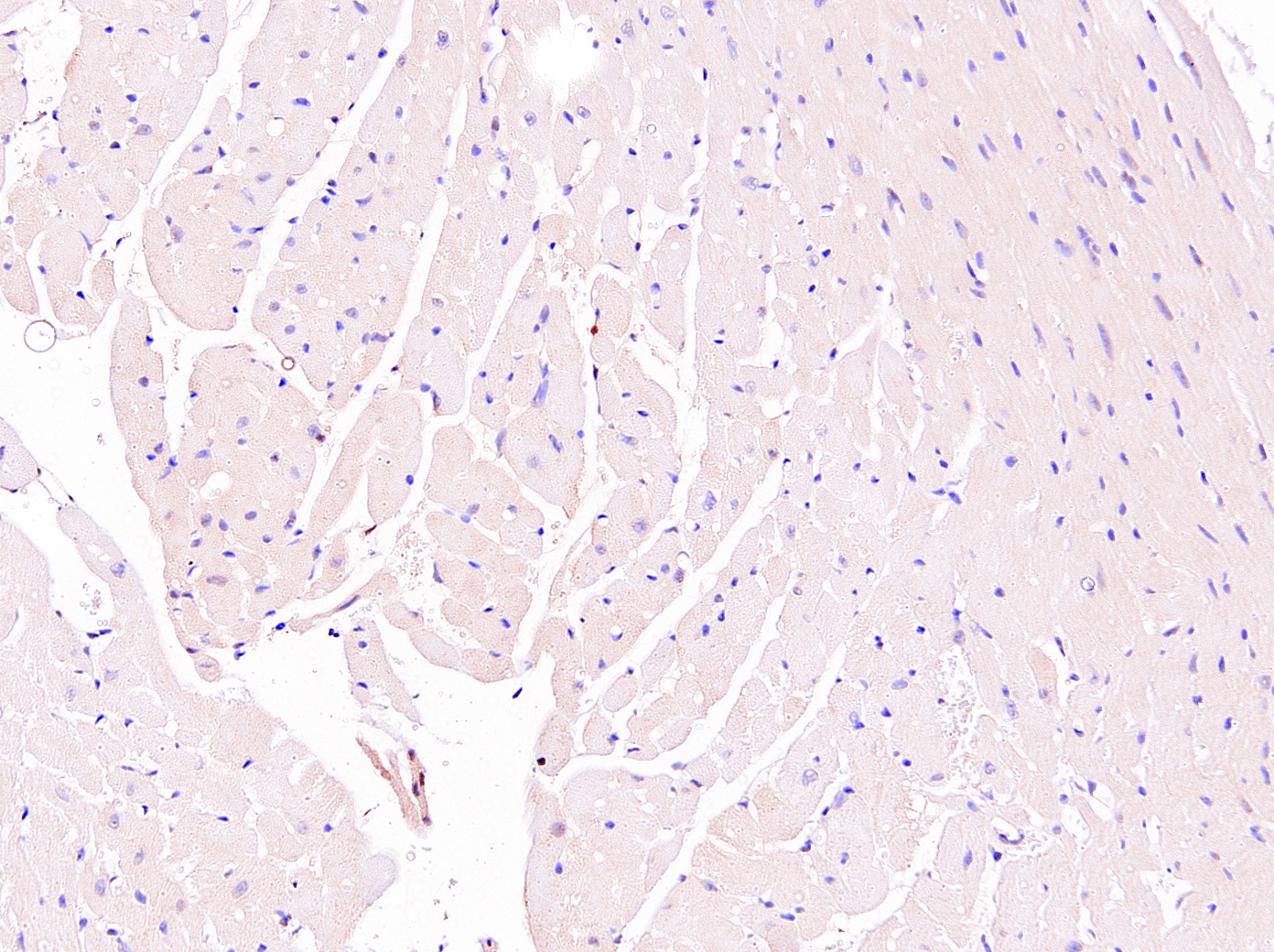

Supplement: Figure 4—source data 1. [file elife-55513-fig4-data1.zip › p16_images_for_eLife/p16_images_Ann_Chiao_for_eLife/Old Controls/OCL_6/2.1_c_unmixed_comp.jpg]

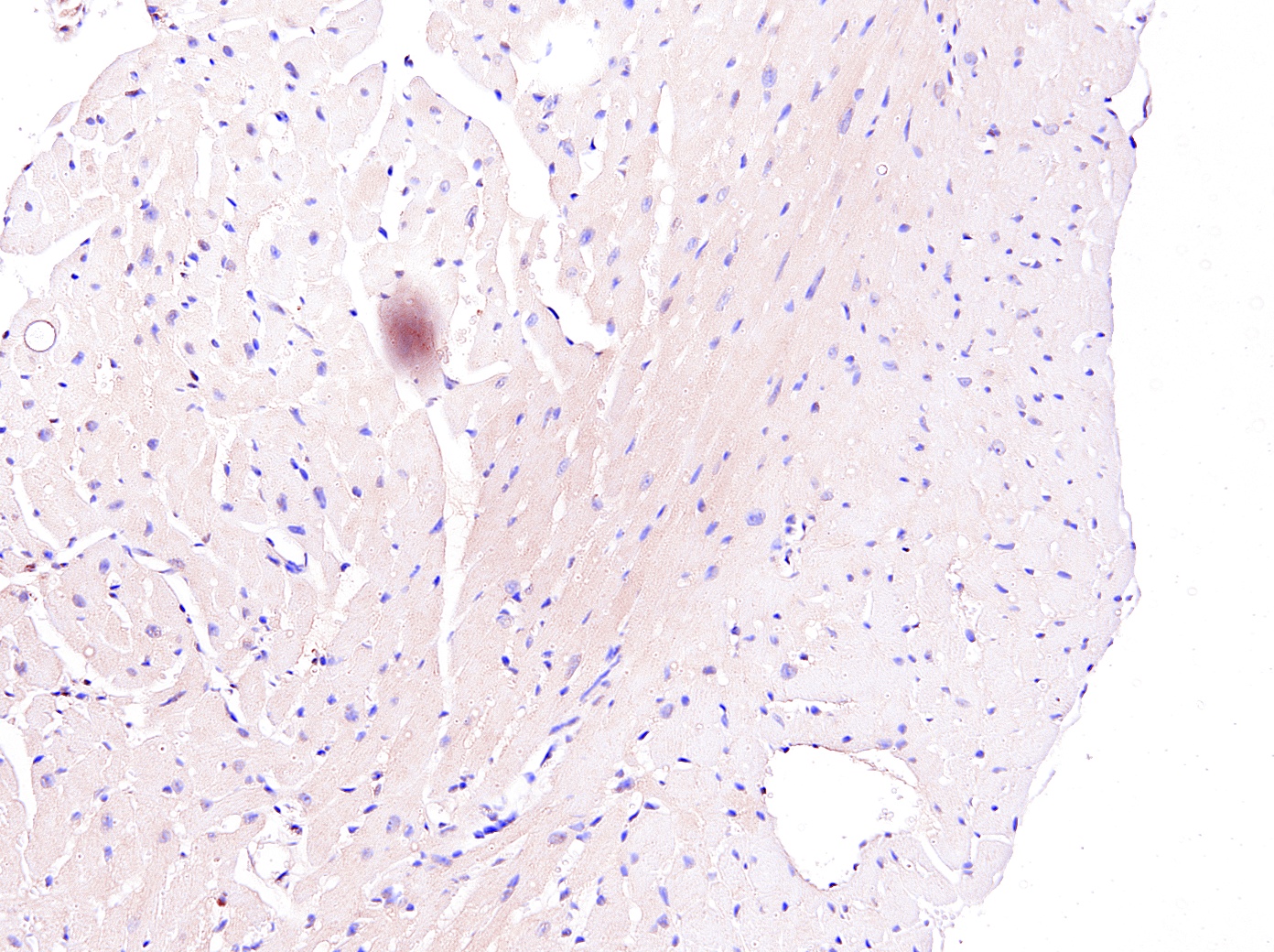

Supplement: Figure 4—source data 1. [file elife-55513-fig4-data1.zip › p16_images_for_eLife/p16_images_Ann_Chiao_for_eLife/Old Controls/OCL_6/2.1_d_unmixed_comp.jpg]

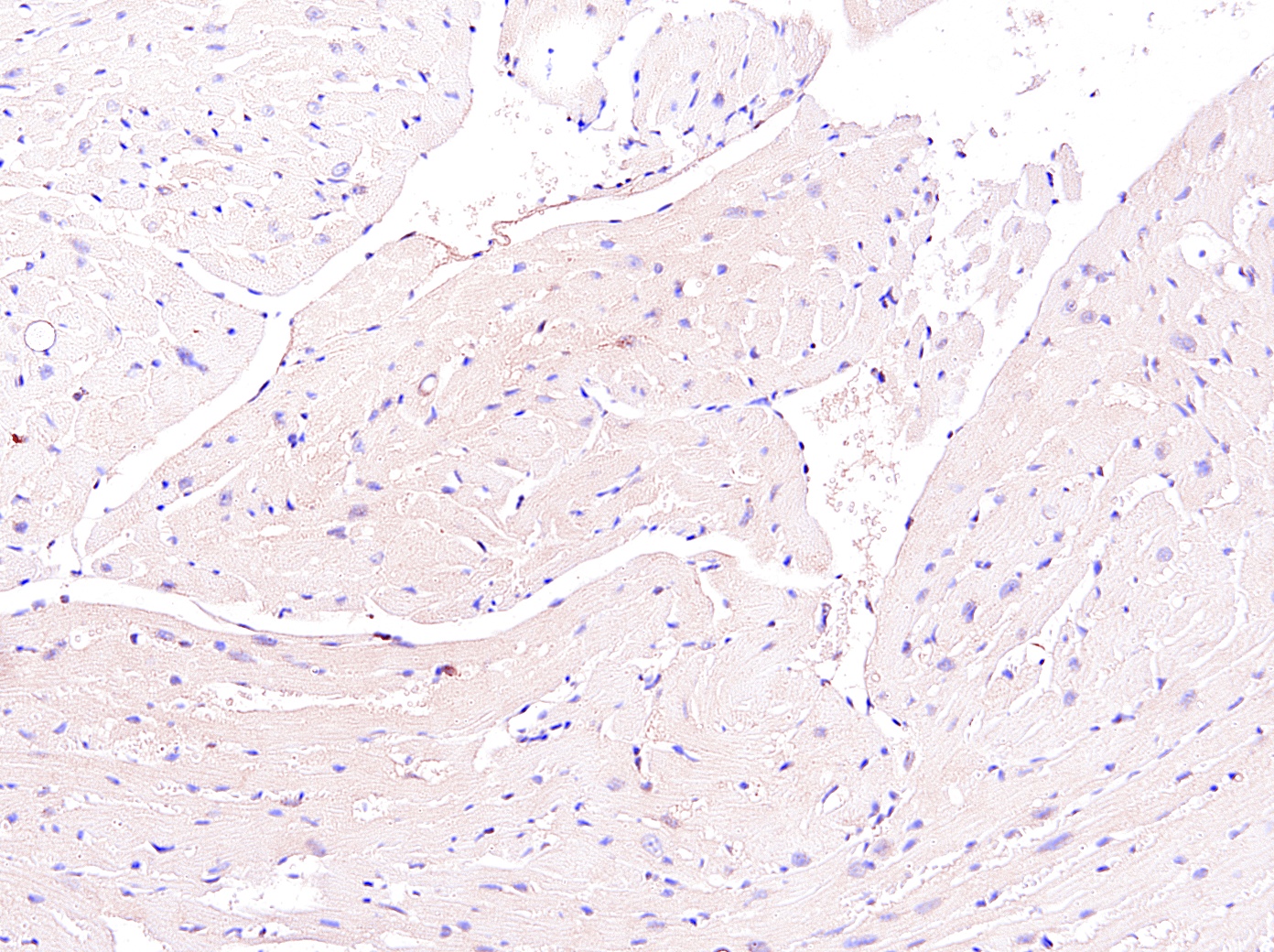

Supplement: Figure 4—source data 1. [file elife-55513-fig4-data1.zip › p16_images_for_eLife/p16_images_Ann_Chiao_for_eLife/Old Controls/OCL_6/2.1_e_unmixed_comp.jpg]

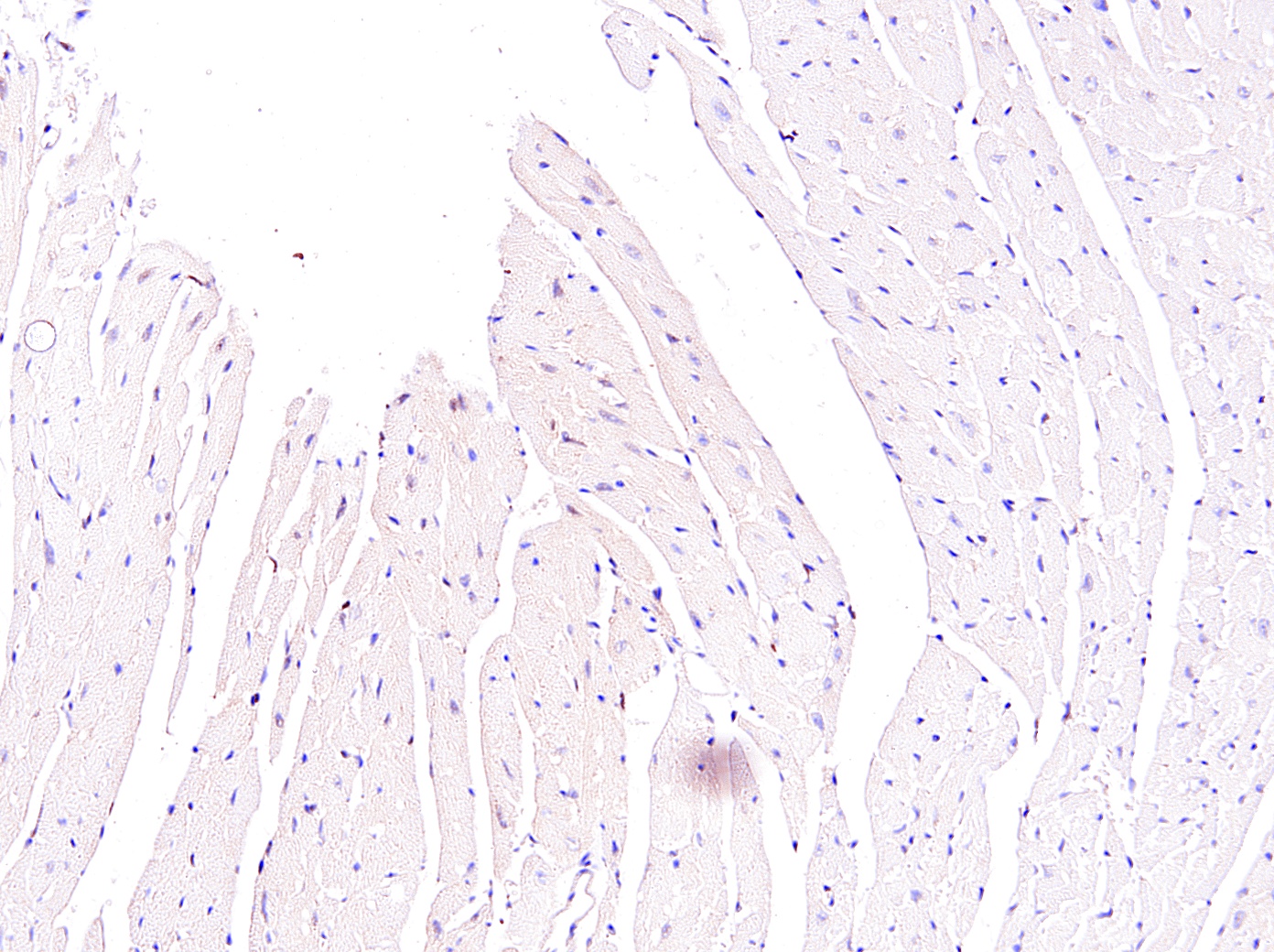

Supplement: Figure 4—source data 1. [file elife-55513-fig4-data1.zip › p16_images_for_eLife/p16_images_Ann_Chiao_for_eLife/Old Controls/OCL_6/2.1_f_unmixed_comp.jpg]

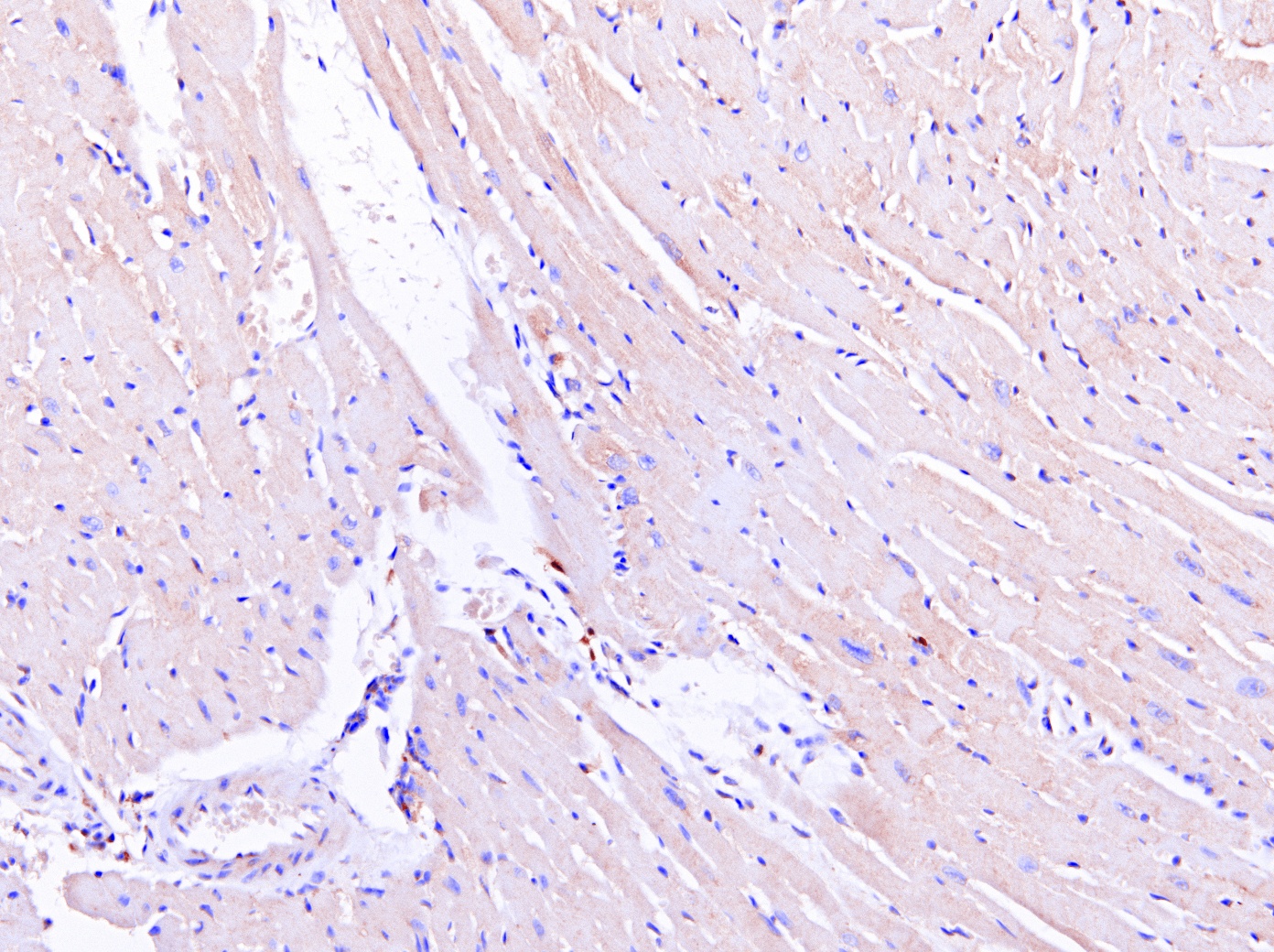

Supplement: Figure 4—source data 1. [file elife-55513-fig4-data1.zip › p16_images_for_eLife/p16_images_Ann_Chiao_for_eLife/Old Controls/OCL_6/2.1_g_unmixed_comp.jpg]

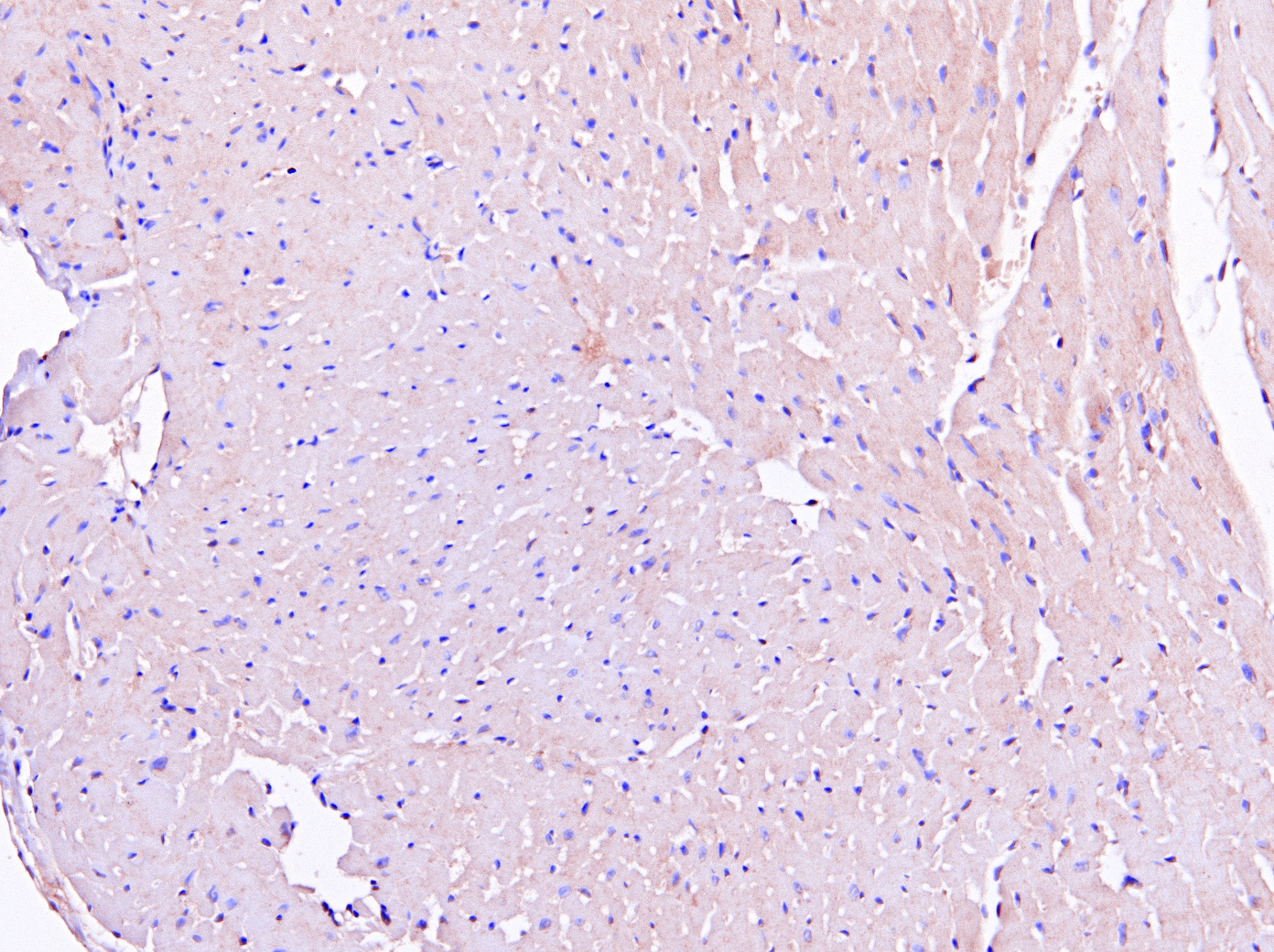

Supplement: Figure 4—source data 1. [file elife-55513-fig4-data1.zip › p16_images_for_eLife/p16_images_Ann_Chiao_for_eLife/Old Controls/OCL_6/2.1_h_unmixed_comp.jpg]

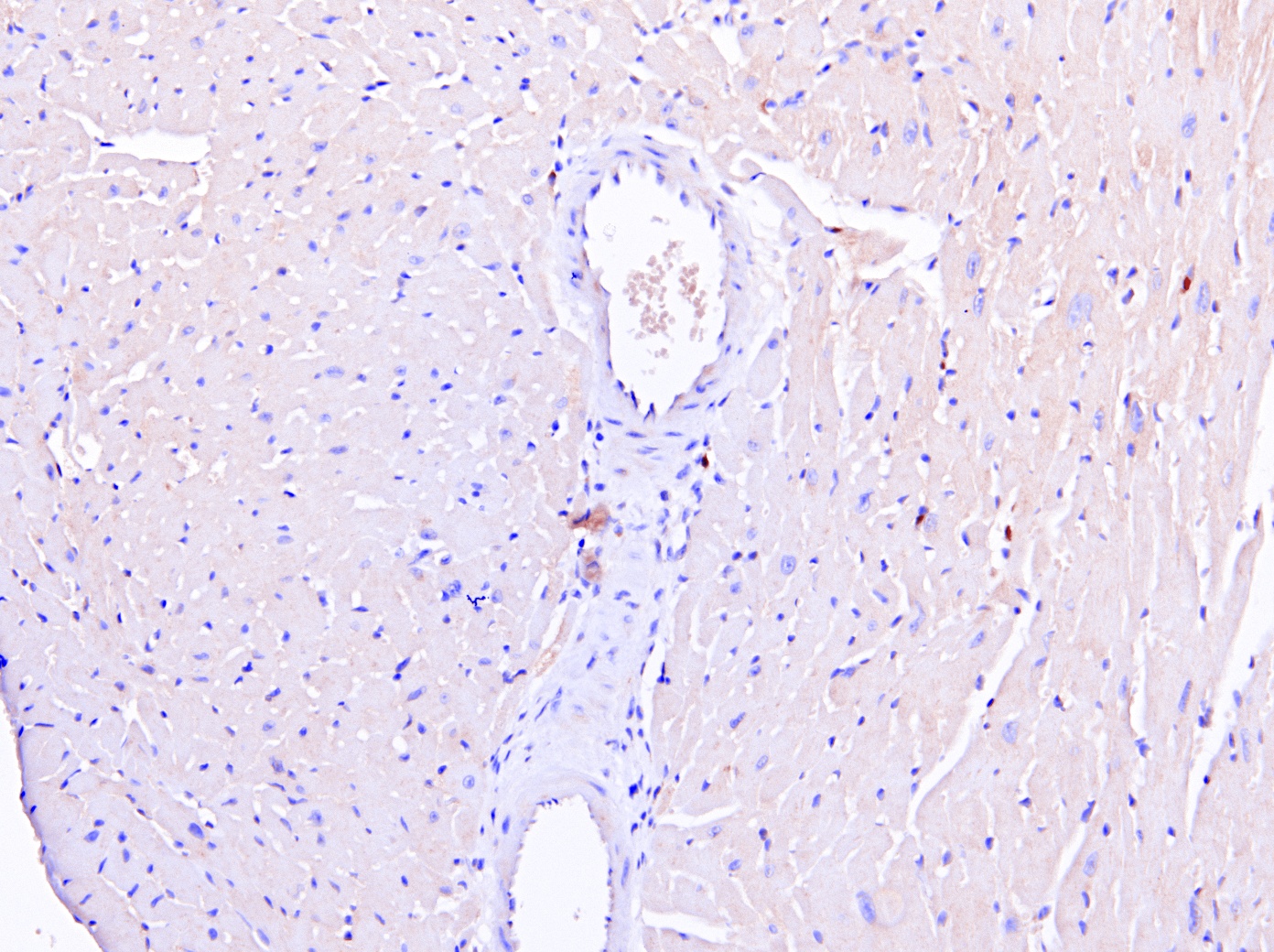

Supplement: Figure 4—source data 1. [file elife-55513-fig4-data1.zip › p16_images_for_eLife/p16_images_Ann_Chiao_for_eLife/Old Controls/OCL_6/2.1_i_unmixed_comp.jpg]

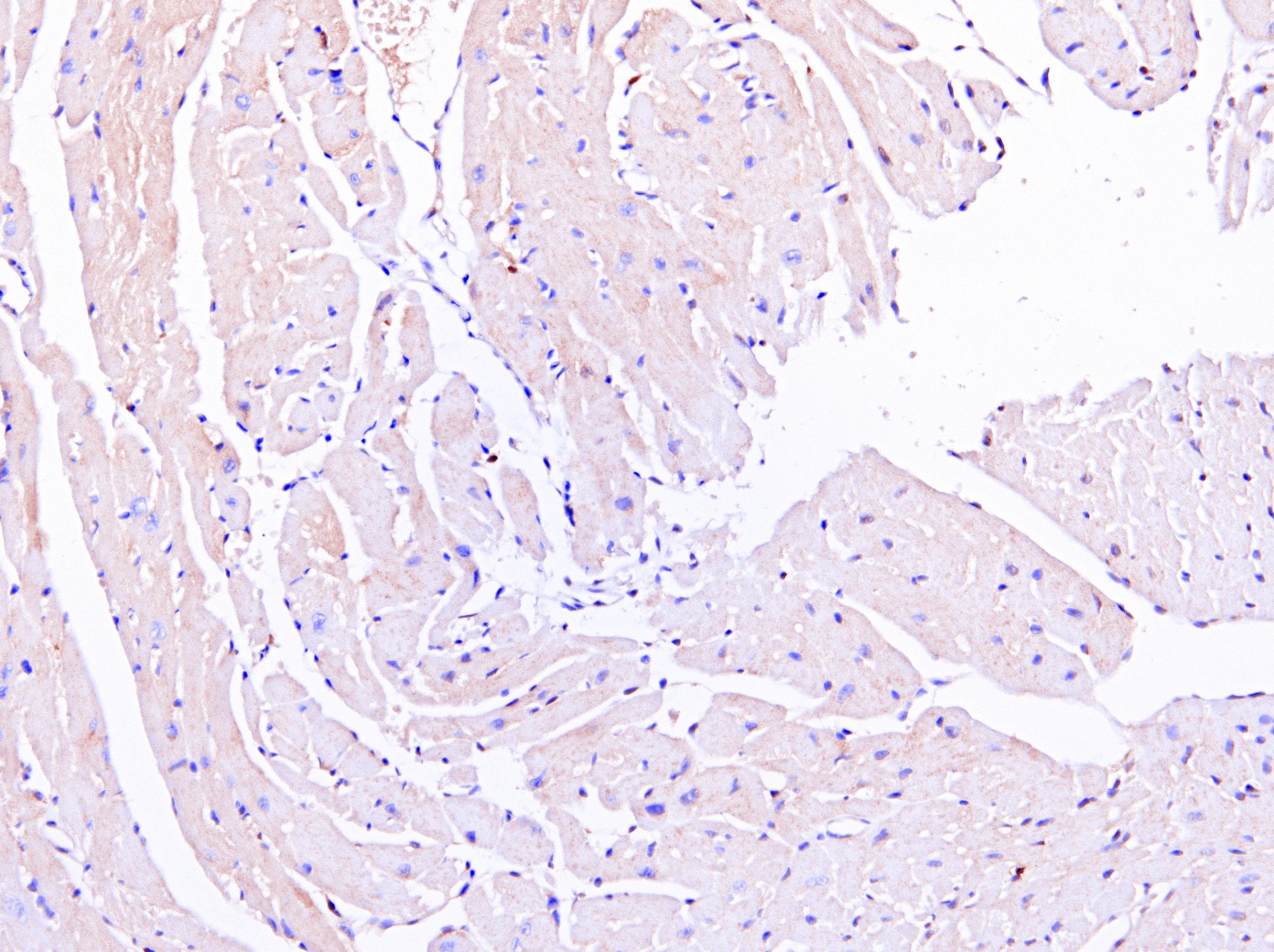

Supplement: Figure 4—source data 1. [file elife-55513-fig4-data1.zip › p16_images_for_eLife/p16_images_Ann_Chiao_for_eLife/Old Controls/OCL_6/2.1_j_unmixed_comp.jpg]

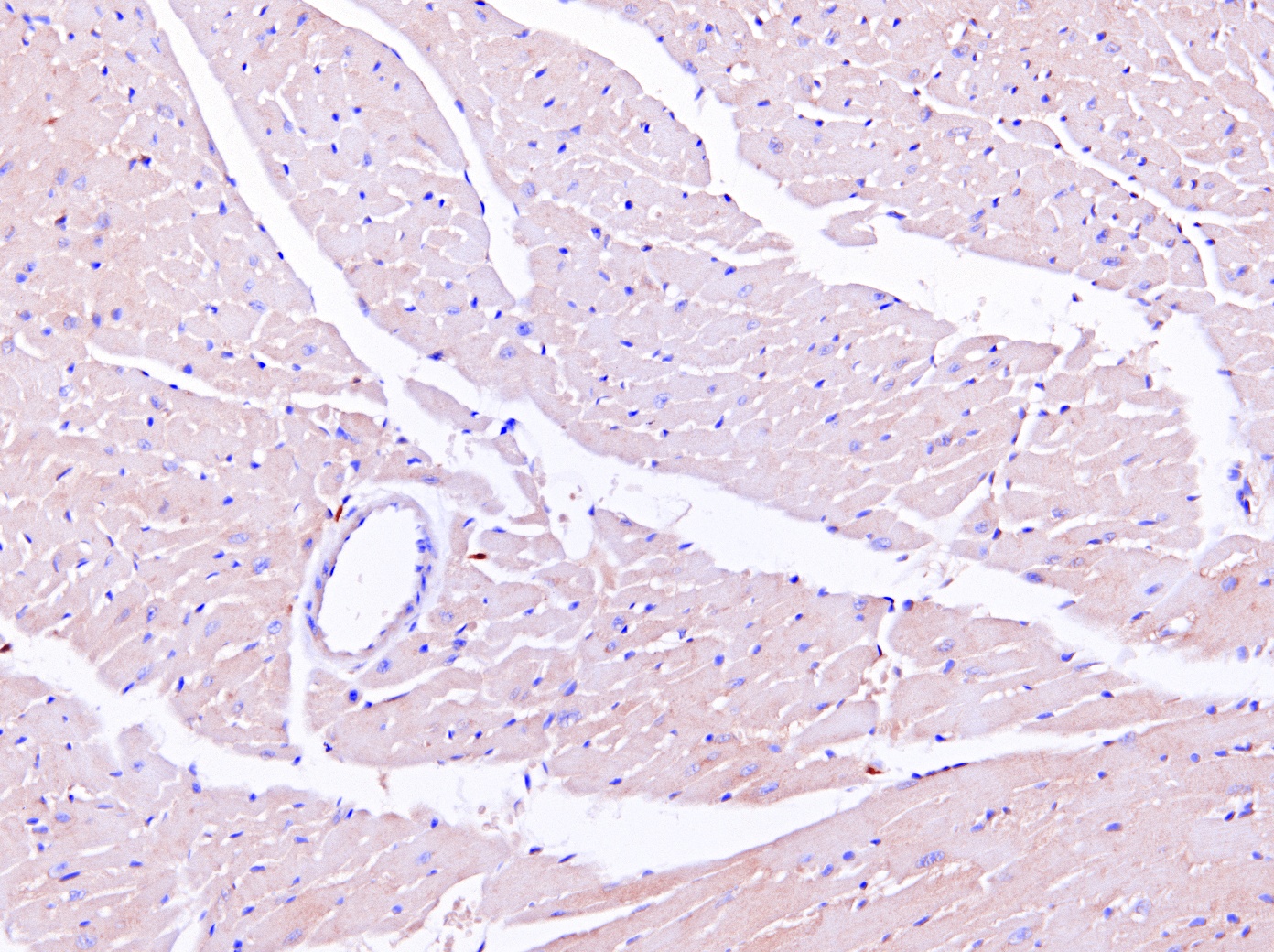

Supplement: Figure 4—source data 1. [file elife-55513-fig4-data1.zip › p16_images_for_eLife/p16_images_Ann_Chiao_for_eLife/Old Controls/OCL_7/3.2_a.jpg]

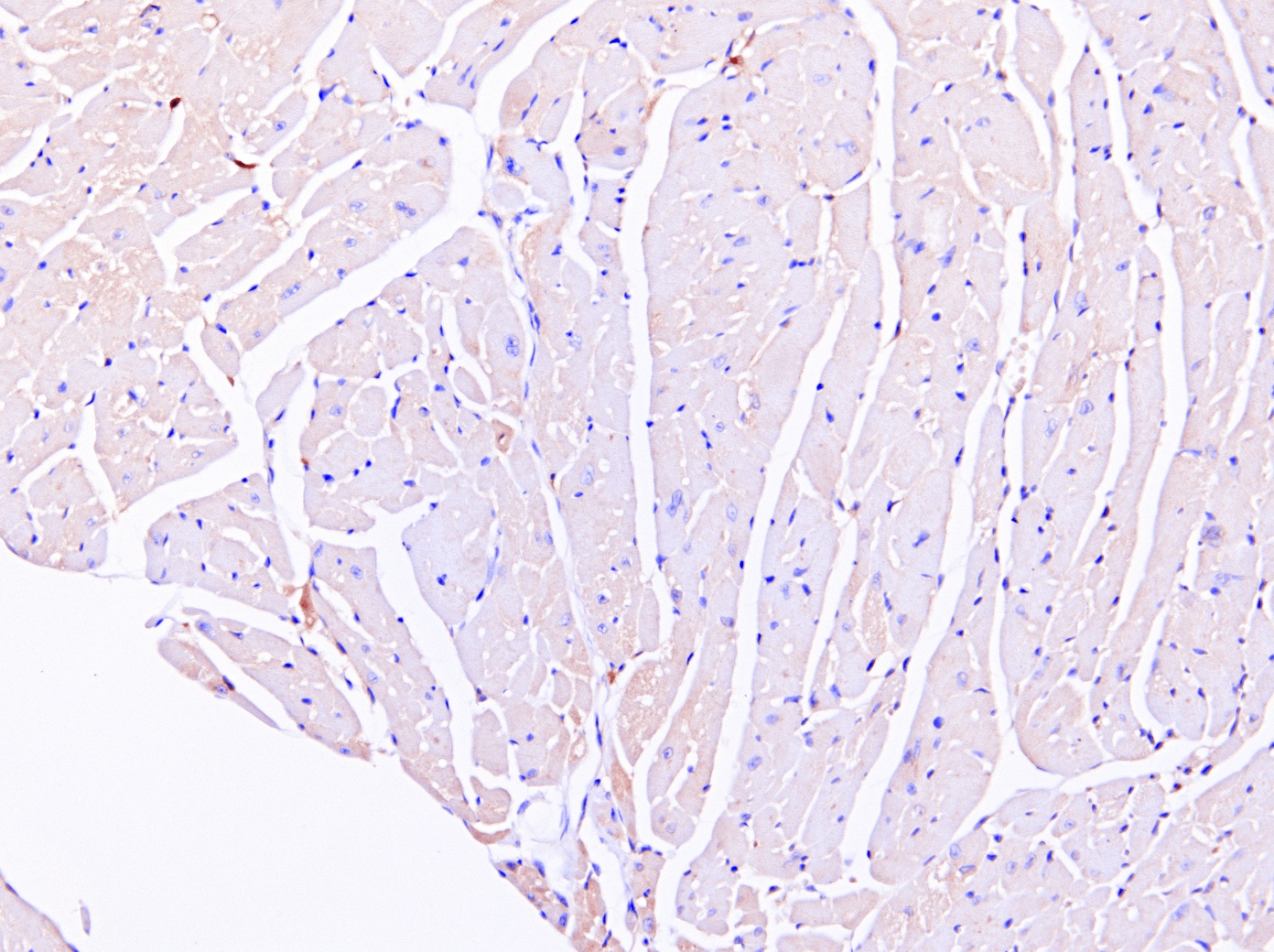

Supplement: Figure 4—source data 1. [file elife-55513-fig4-data1.zip › p16_images_for_eLife/p16_images_Ann_Chiao_for_eLife/Old Controls/OCL_7/3.2_b.jpg]

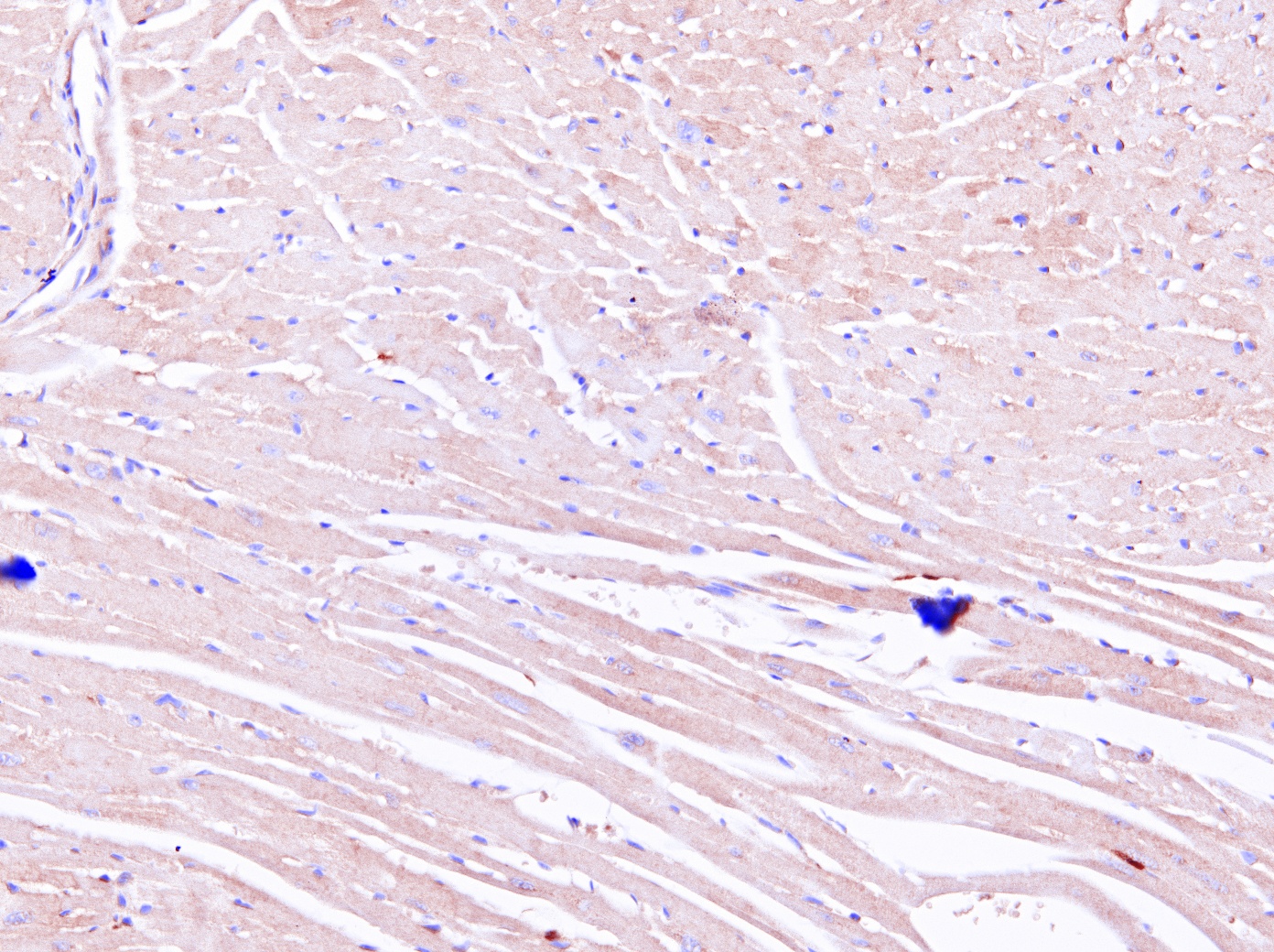

Supplement: Figure 4—source data 1. [file elife-55513-fig4-data1.zip › p16_images_for_eLife/p16_images_Ann_Chiao_for_eLife/Old Controls/OCL_7/3.2_c.jpg]

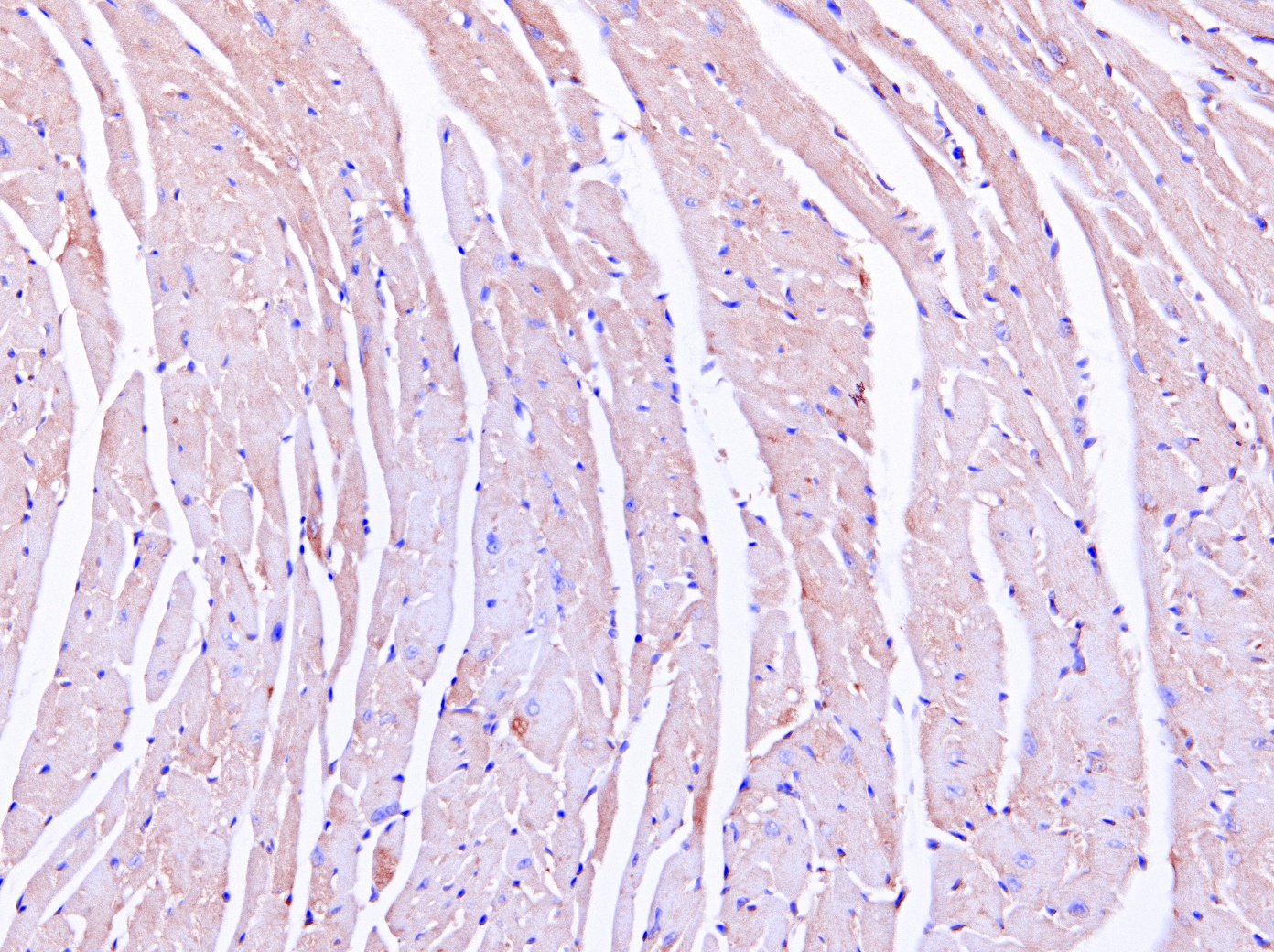

Supplement: Figure 4—source data 1. [file elife-55513-fig4-data1.zip › p16_images_for_eLife/p16_images_Ann_Chiao_for_eLife/Old Controls/OCL_7/3.2_d.jpg]

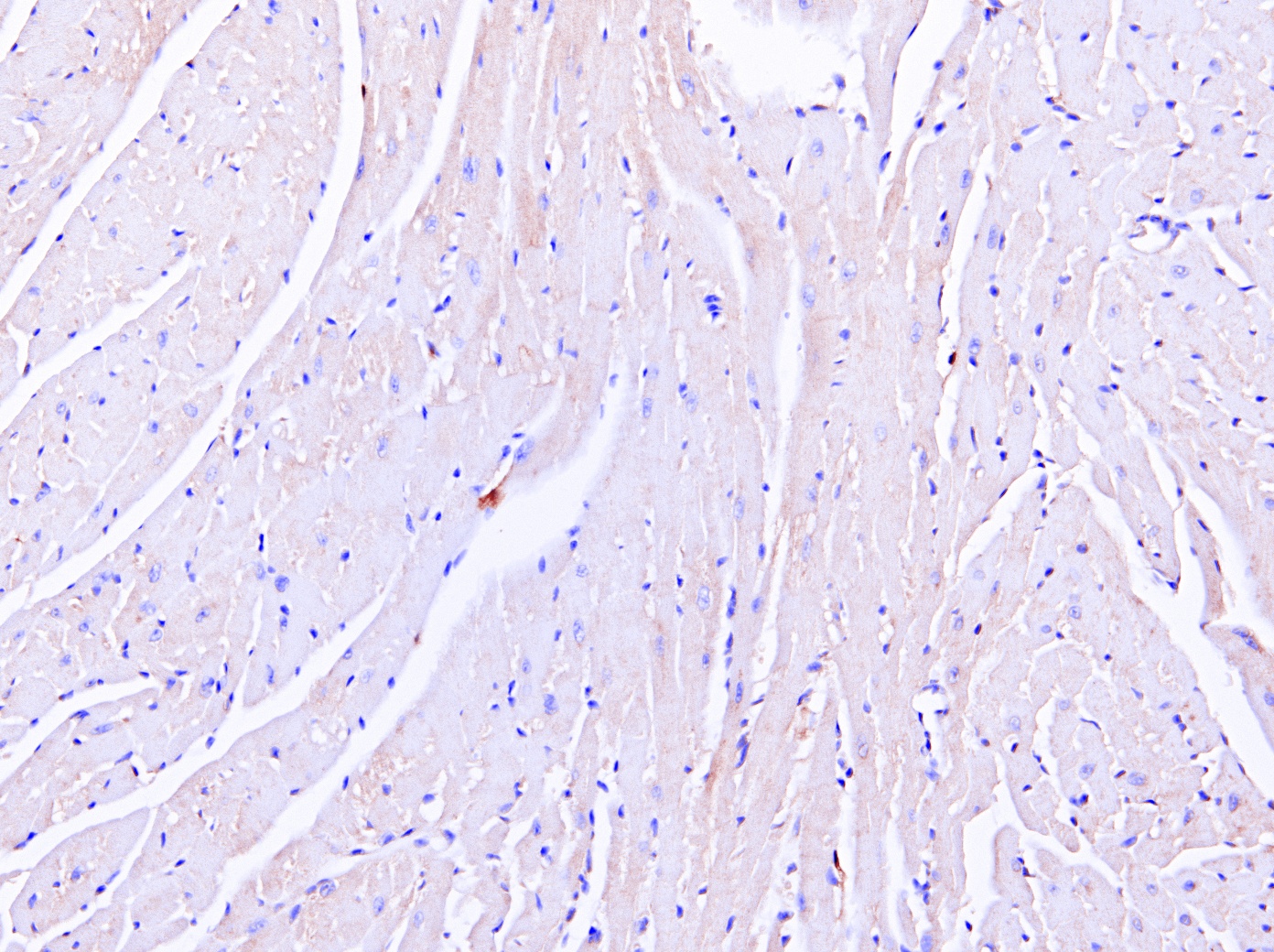

Supplement: Figure 4—source data 1. [file elife-55513-fig4-data1.zip › p16_images_for_eLife/p16_images_Ann_Chiao_for_eLife/Old Controls/OCL_7/3.2_e.jpg]

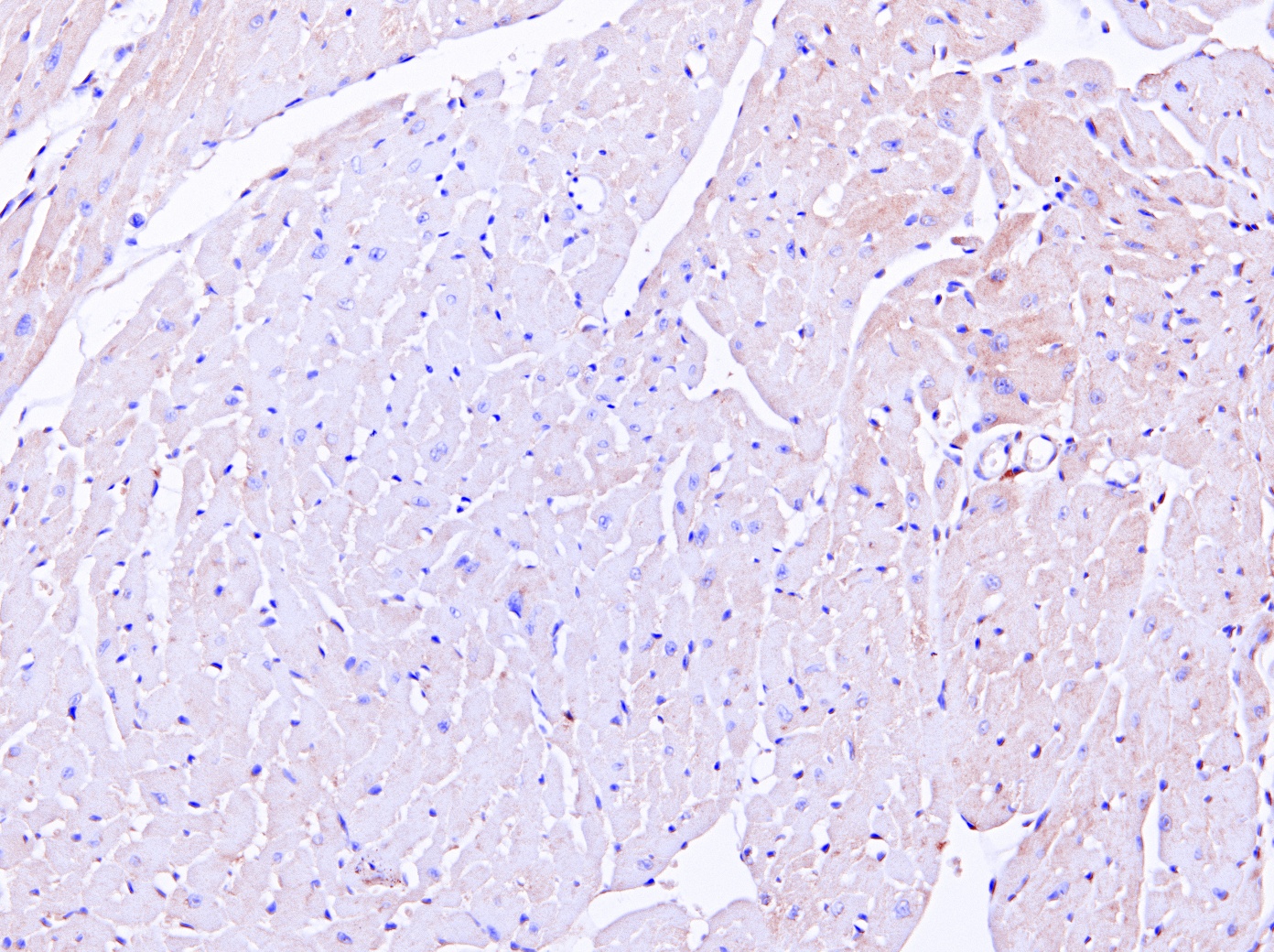

Supplement: Figure 4—source data 1. [file elife-55513-fig4-data1.zip › p16_images_for_eLife/p16_images_Ann_Chiao_for_eLife/Old Controls/OCL_7/3.2_f.jpg]

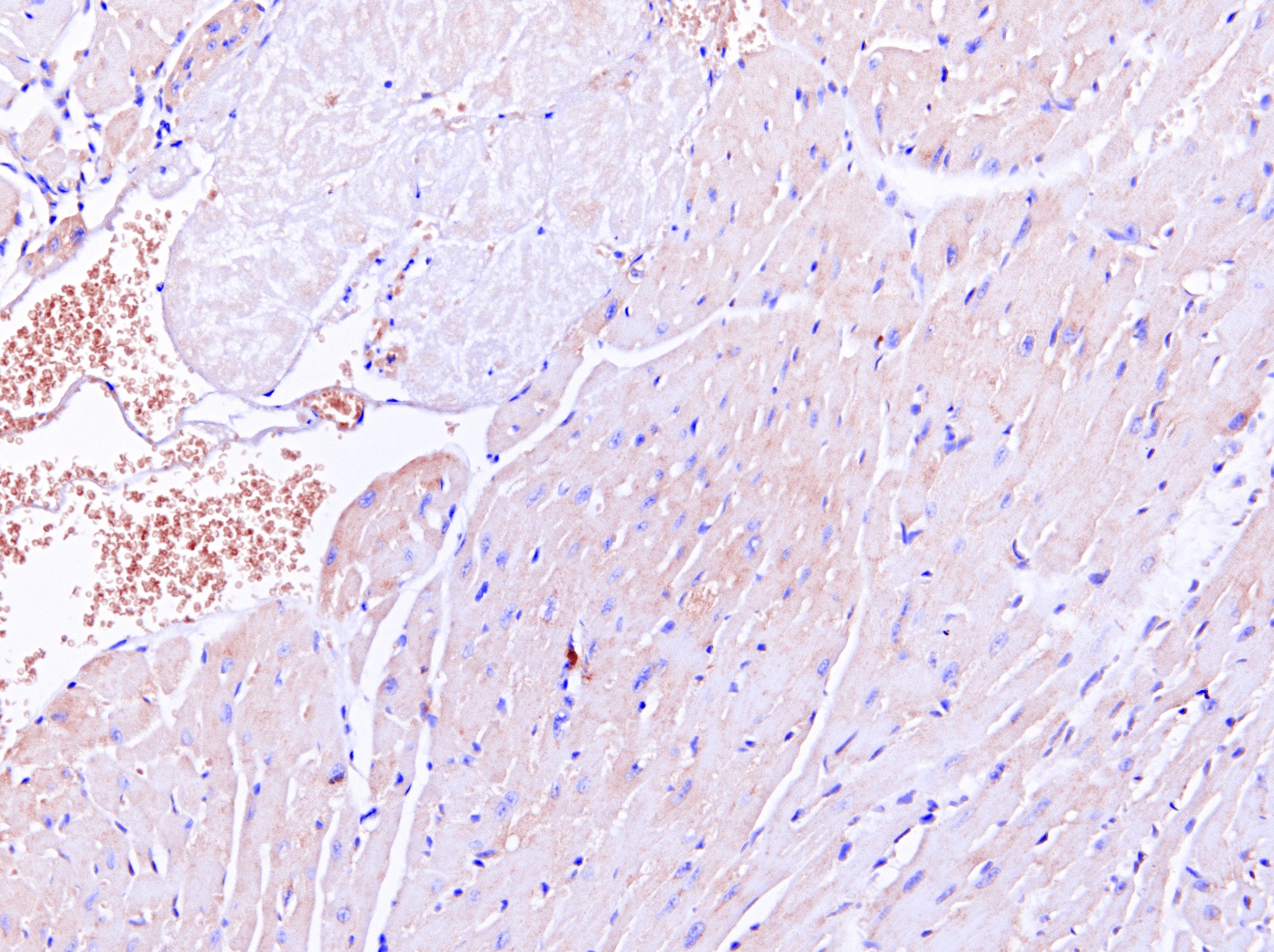

Supplement: Figure 4—source data 1. [file elife-55513-fig4-data1.zip › p16_images_for_eLife/p16_images_Ann_Chiao_for_eLife/Old Controls/OCL_7/3.2_g.jpg]

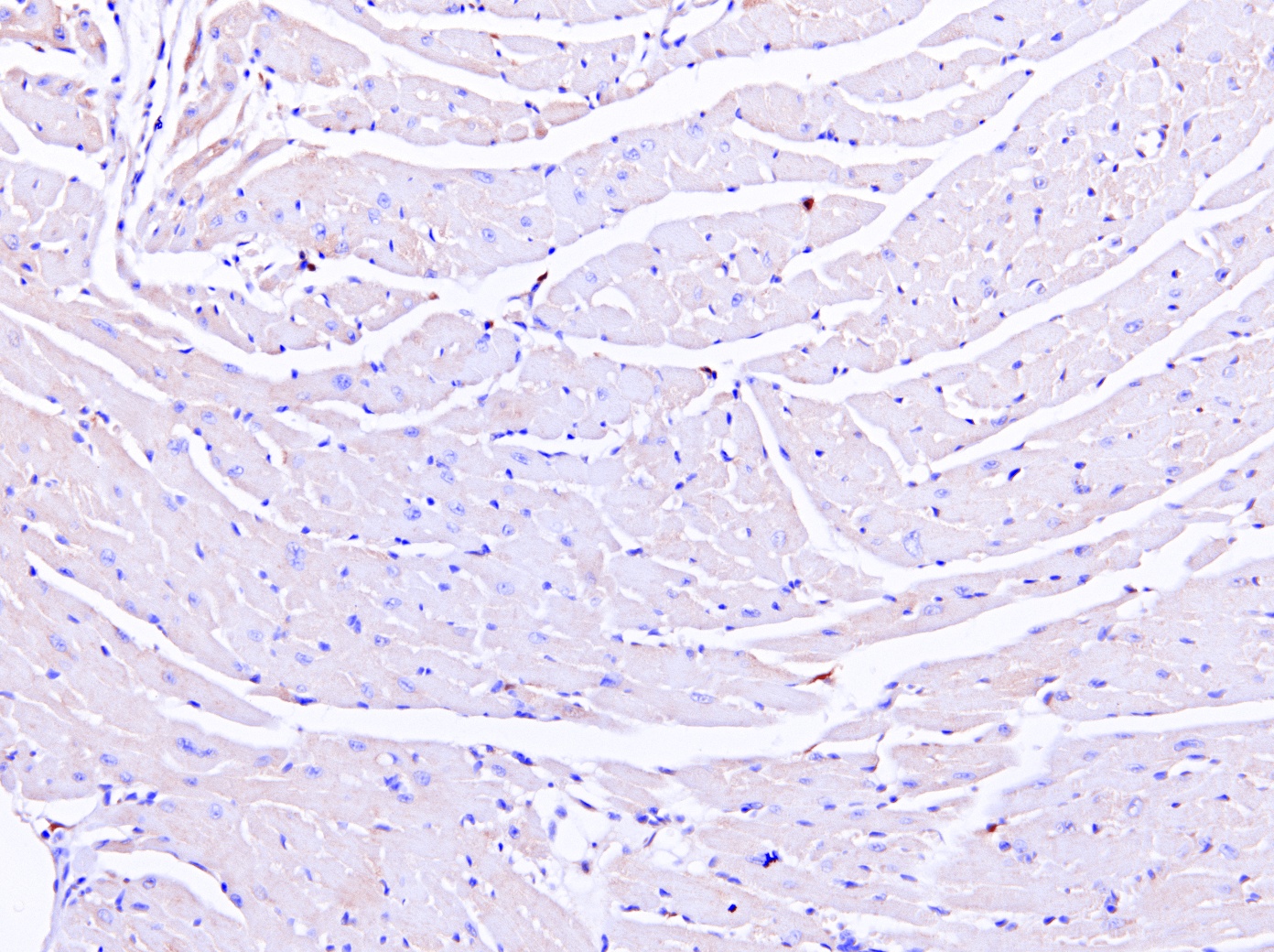

Supplement: Figure 4—source data 1. [file elife-55513-fig4-data1.zip › p16_images_for_eLife/p16_images_Ann_Chiao_for_eLife/Old Controls/OCL_7/3.2_h.jpg]

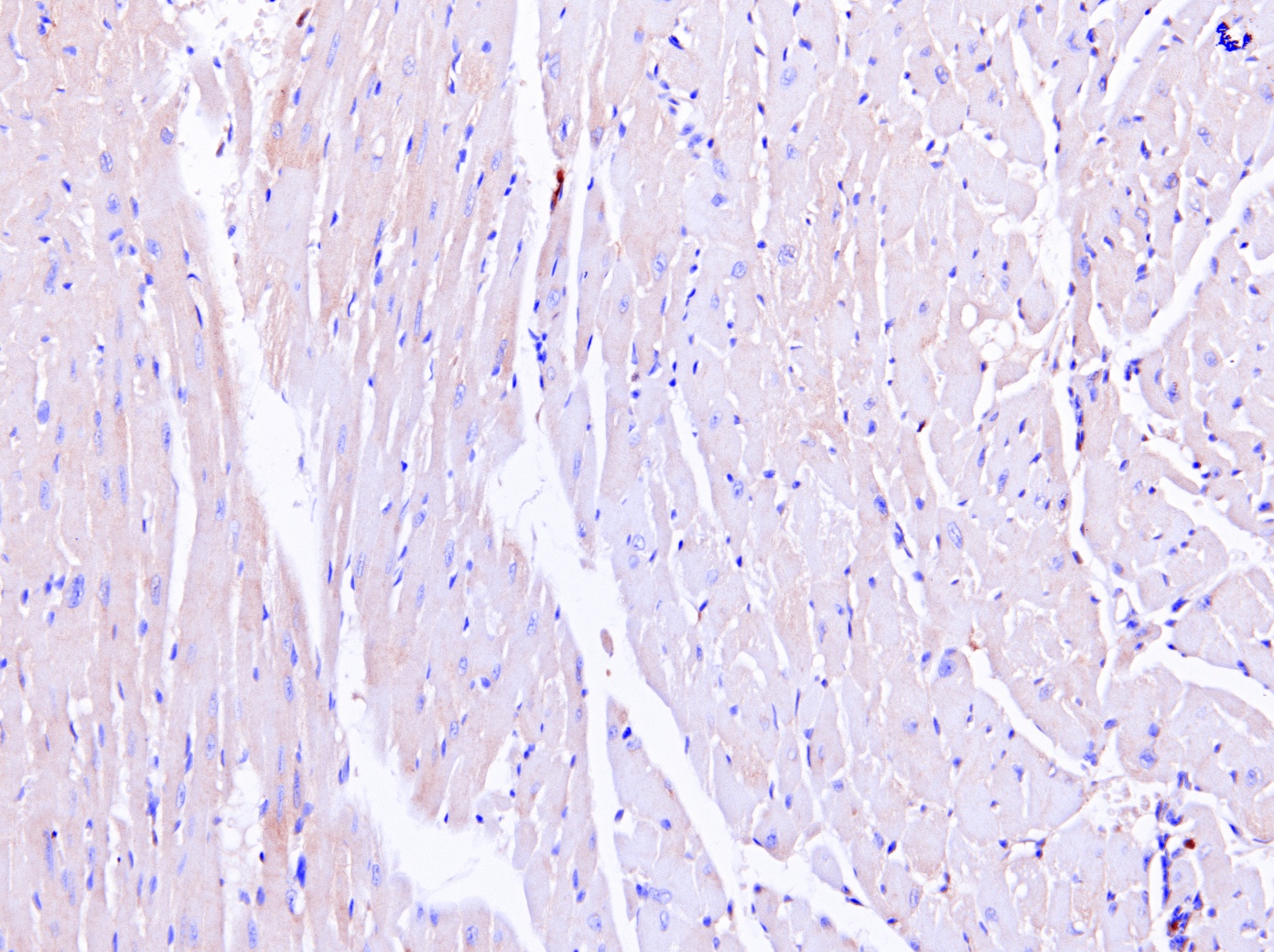

Supplement: Figure 4—source data 1. [file elife-55513-fig4-data1.zip › p16_images_for_eLife/p16_images_Ann_Chiao_for_eLife/Old Controls/OCL_7/3.2_i.jpg]

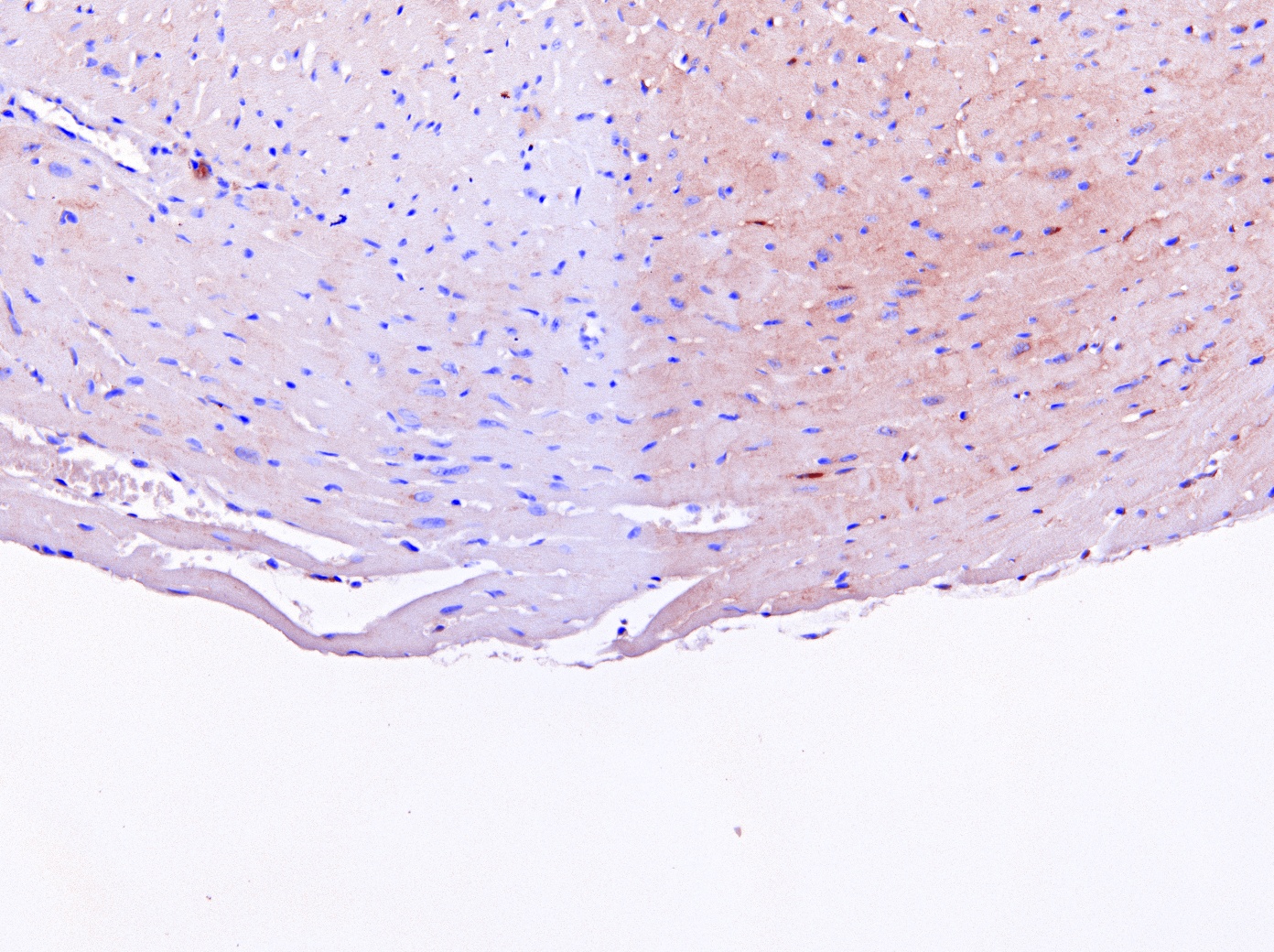

Supplement: Figure 4—source data 1. [file elife-55513-fig4-data1.zip › p16_images_for_eLife/p16_images_Ann_Chiao_for_eLife/Old Controls/OCL_8/4.2_Composite_a.jpg]

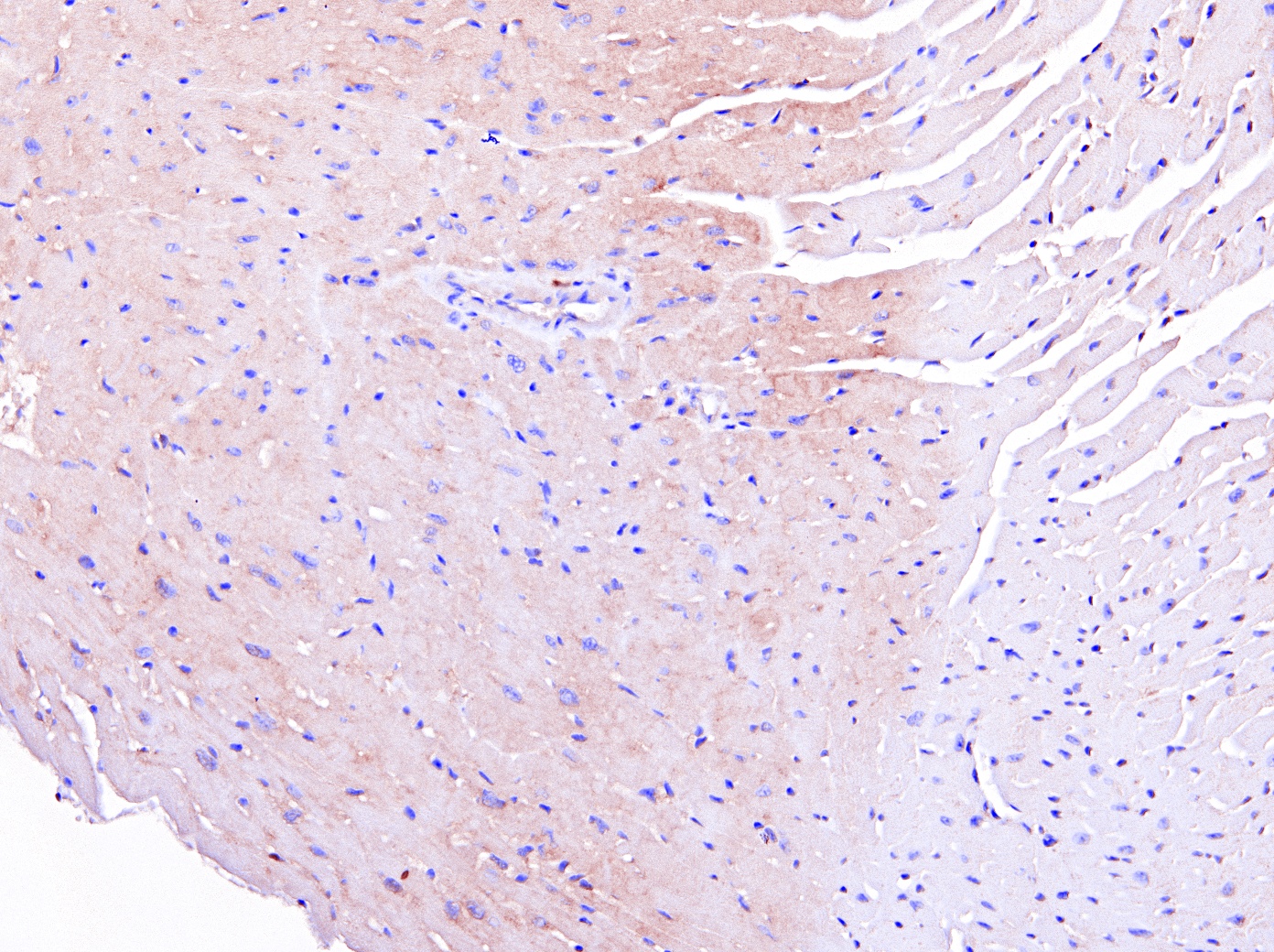

Supplement: Figure 4—source data 1. [file elife-55513-fig4-data1.zip › p16_images_for_eLife/p16_images_Ann_Chiao_for_eLife/Old Controls/OCL_8/4.2_Composite_b.jpg]

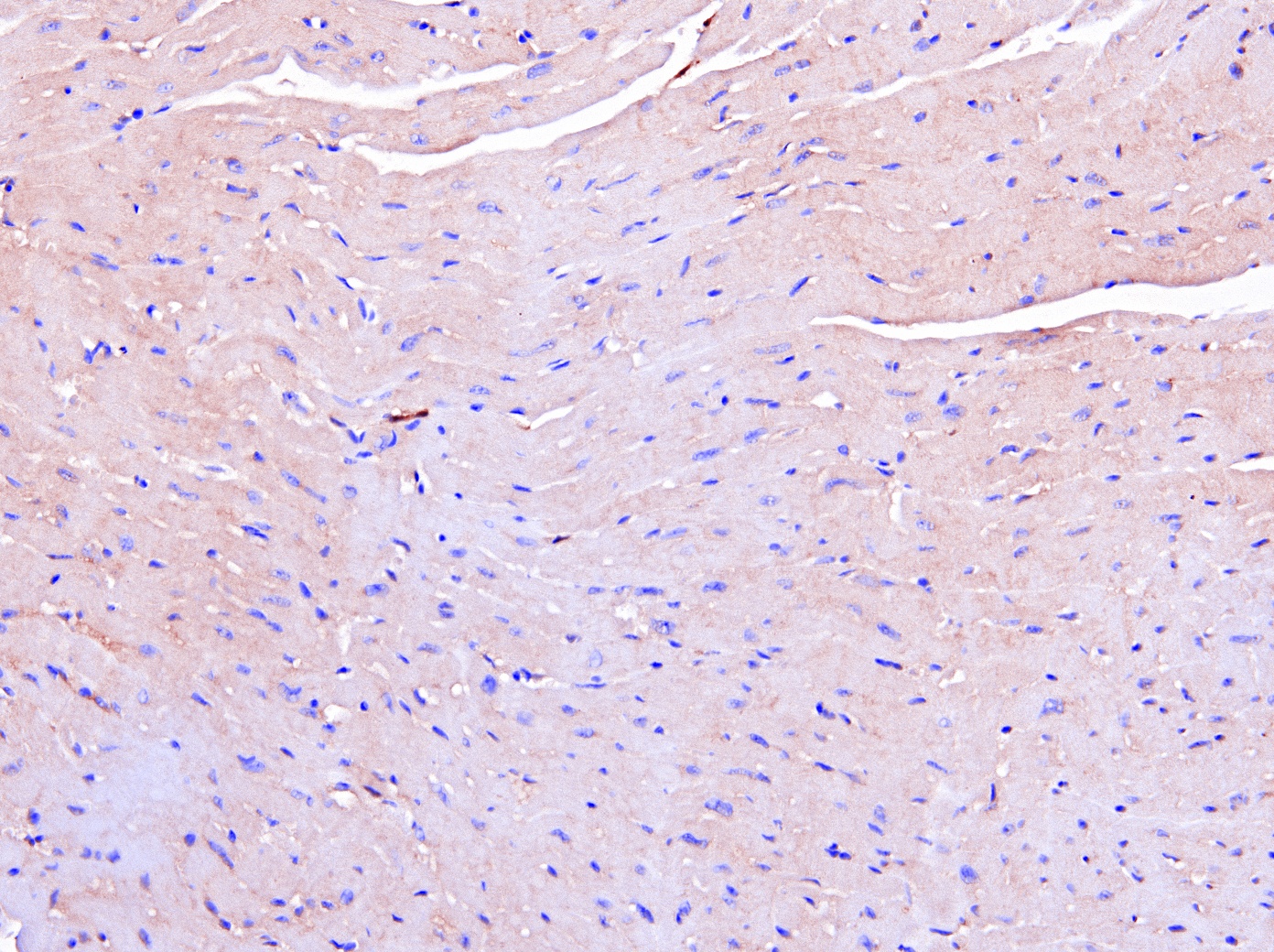

Supplement: Figure 4—source data 1. [file elife-55513-fig4-data1.zip › p16_images_for_eLife/p16_images_Ann_Chiao_for_eLife/Old Controls/OCL_8/4.2_Composite_c.jpg]

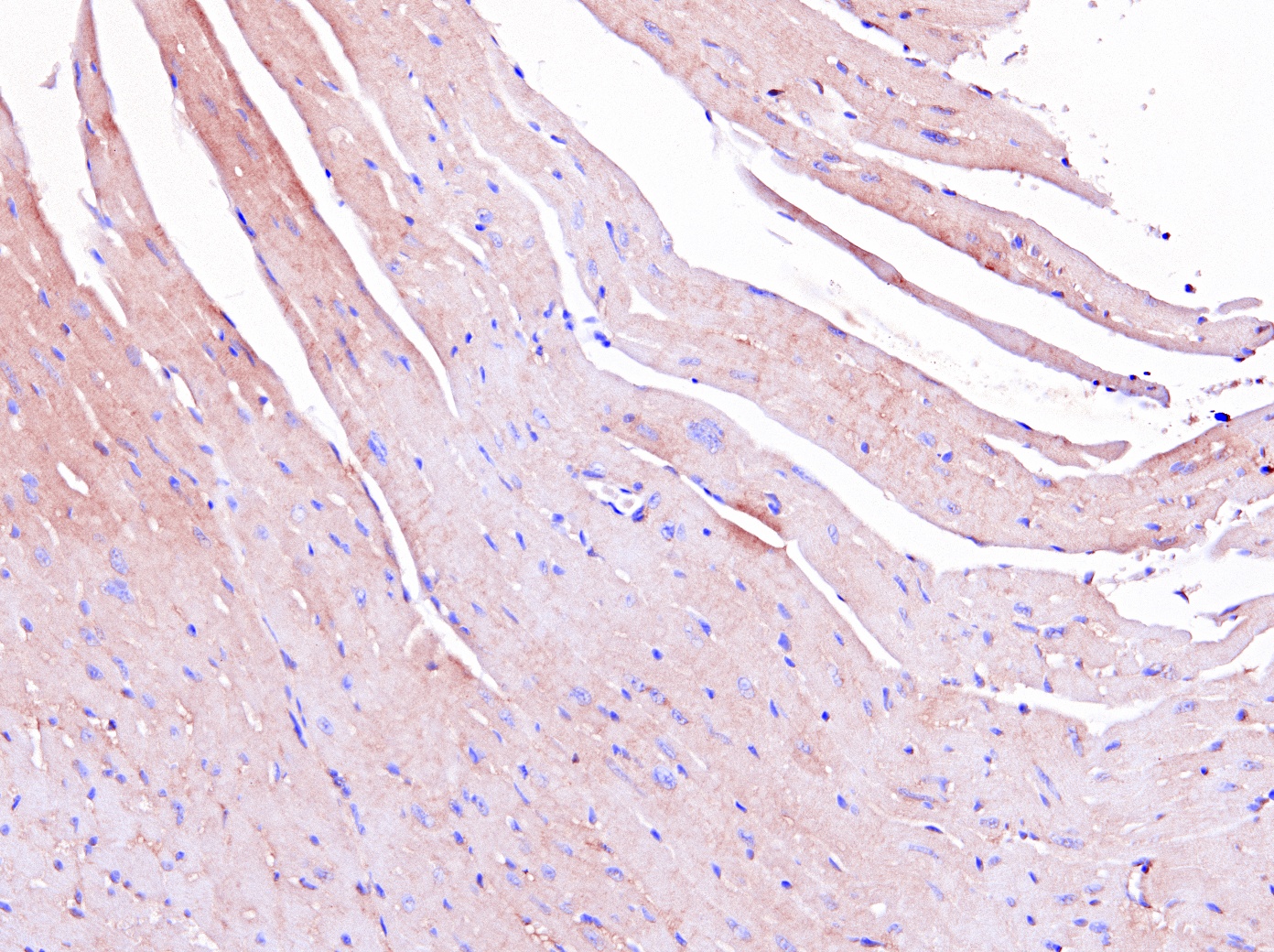

Supplement: Figure 4—source data 1. [file elife-55513-fig4-data1.zip › p16_images_for_eLife/p16_images_Ann_Chiao_for_eLife/Old Controls/OCL_8/4.2_Composite_d.jpg]

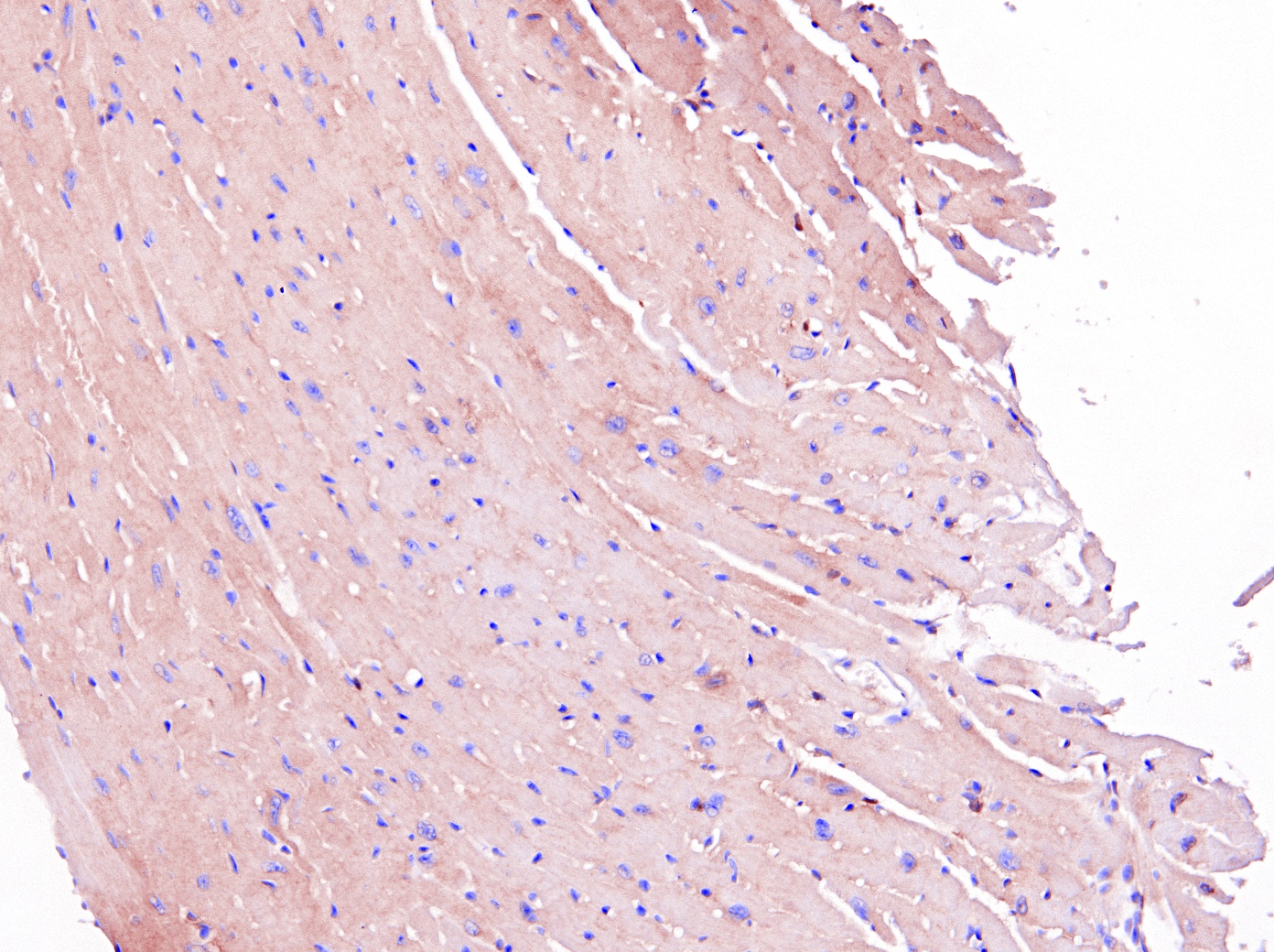

Supplement: Figure 4—source data 1. [file elife-55513-fig4-data1.zip › p16_images_for_eLife/p16_images_Ann_Chiao_for_eLife/Old Controls/OCL_8/4.2_Composite_e.jpg]

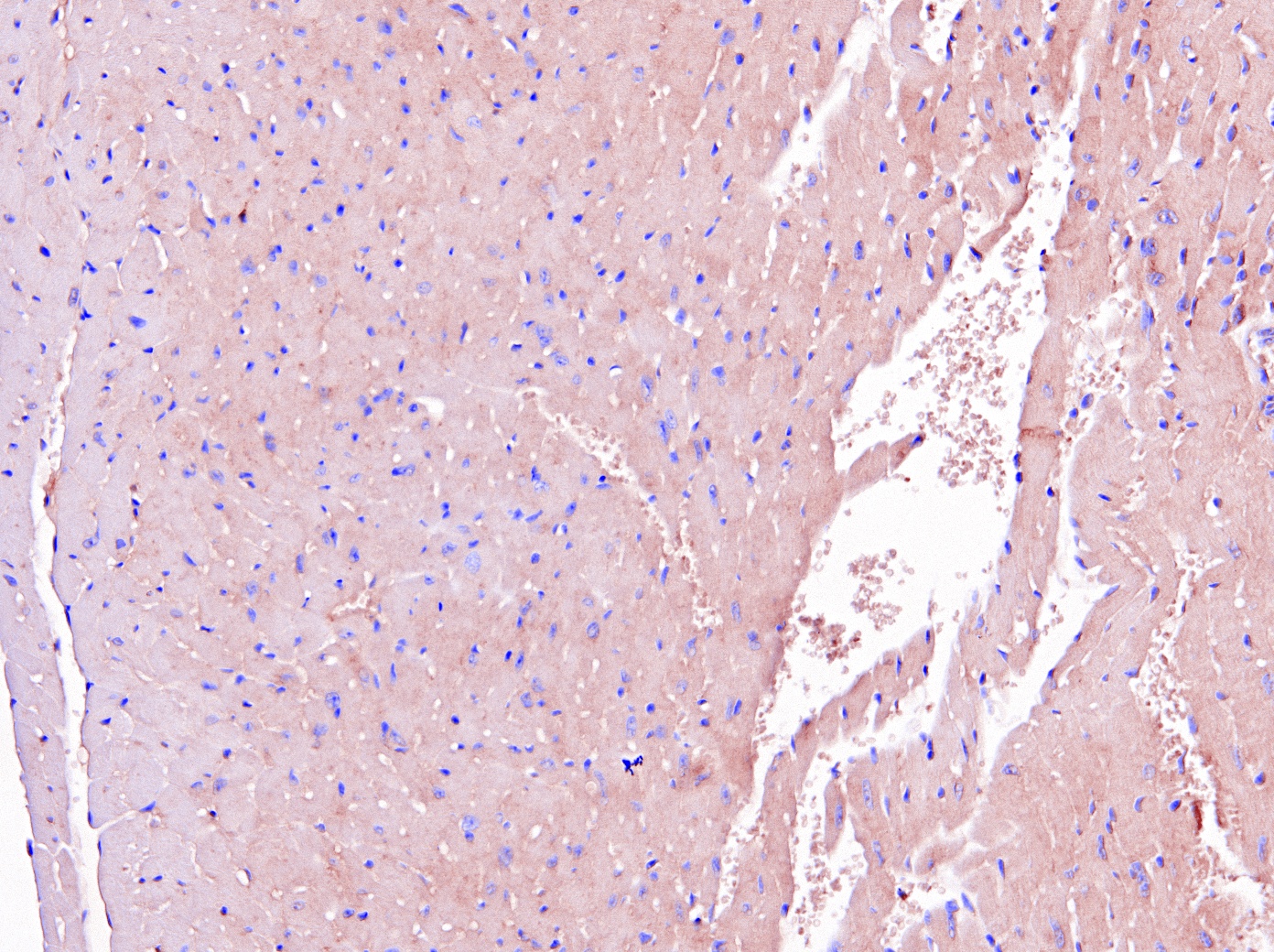

Supplement: Figure 4—source data 1. [file elife-55513-fig4-data1.zip › p16_images_for_eLife/p16_images_Ann_Chiao_for_eLife/Old Controls/OCL_8/4.2_Composite_f.jpg]

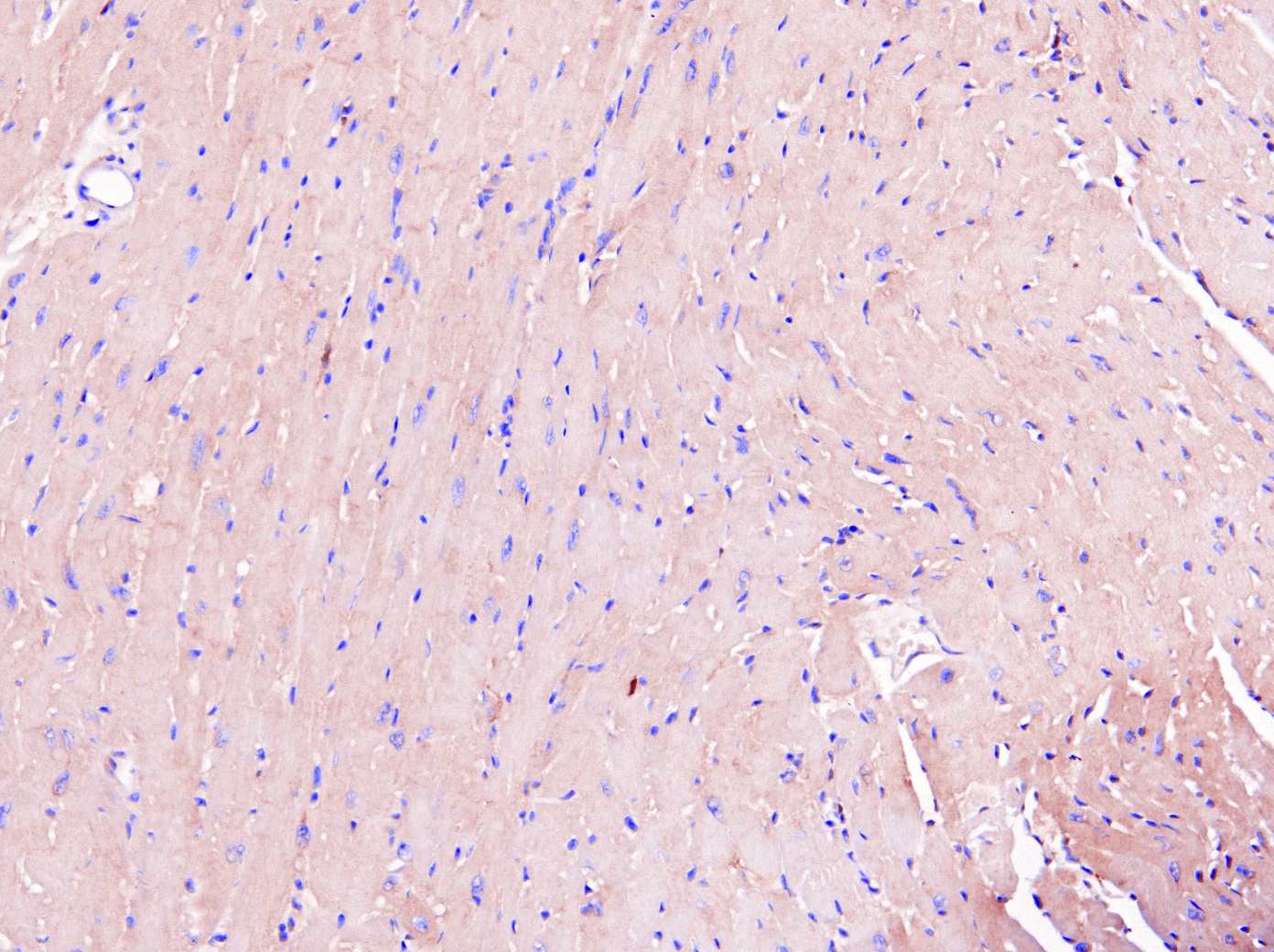

Supplement: Figure 4—source data 1. [file elife-55513-fig4-data1.zip › p16_images_for_eLife/p16_images_Ann_Chiao_for_eLife/Old Controls/OCL_8/4.2_Composite_g.jpg]

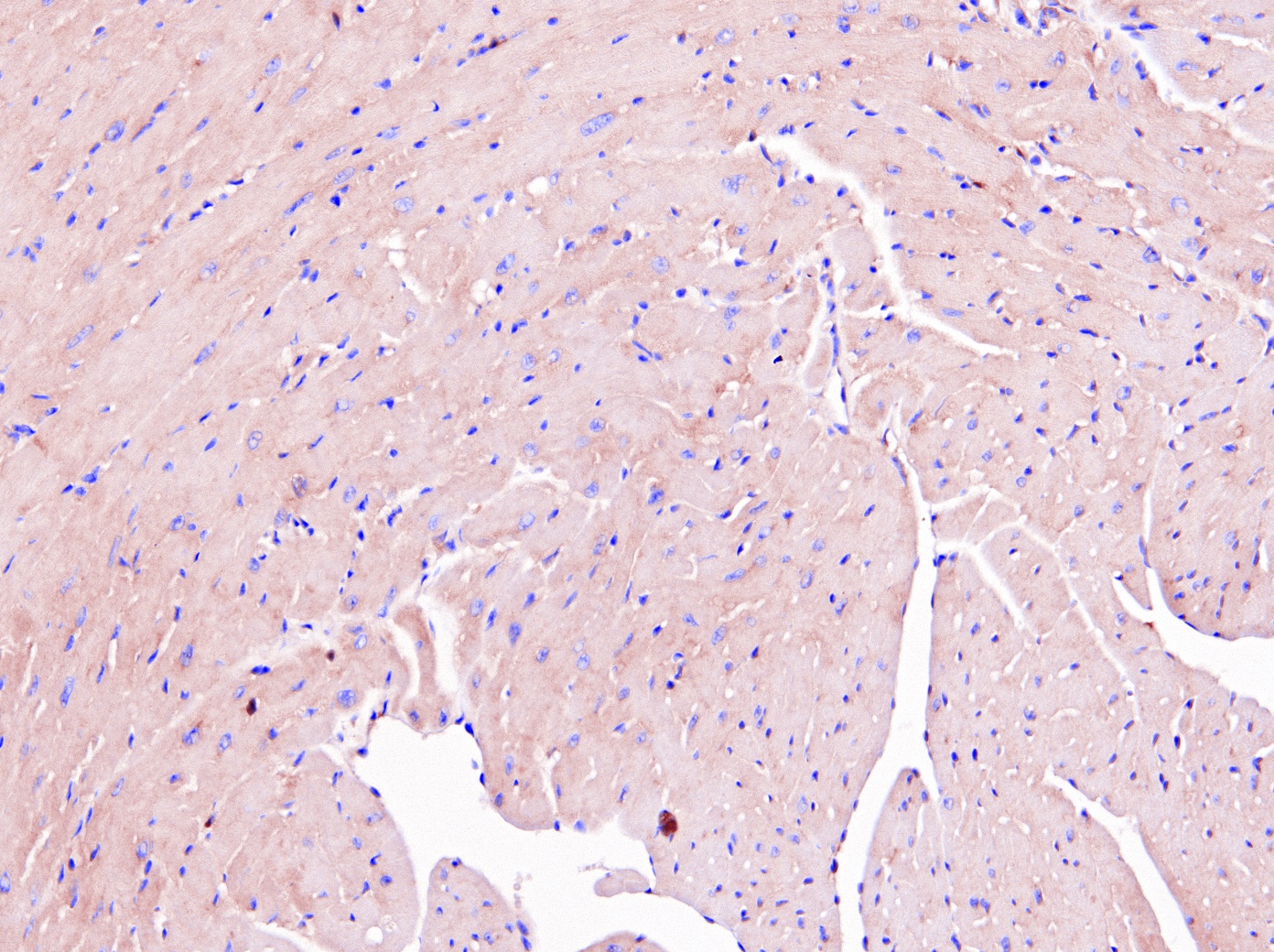

Supplement: Figure 4—source data 1. [file elife-55513-fig4-data1.zip › p16_images_for_eLife/p16_images_Ann_Chiao_for_eLife/Old Controls/OCL_8/4.2_Composite_h.jpg]

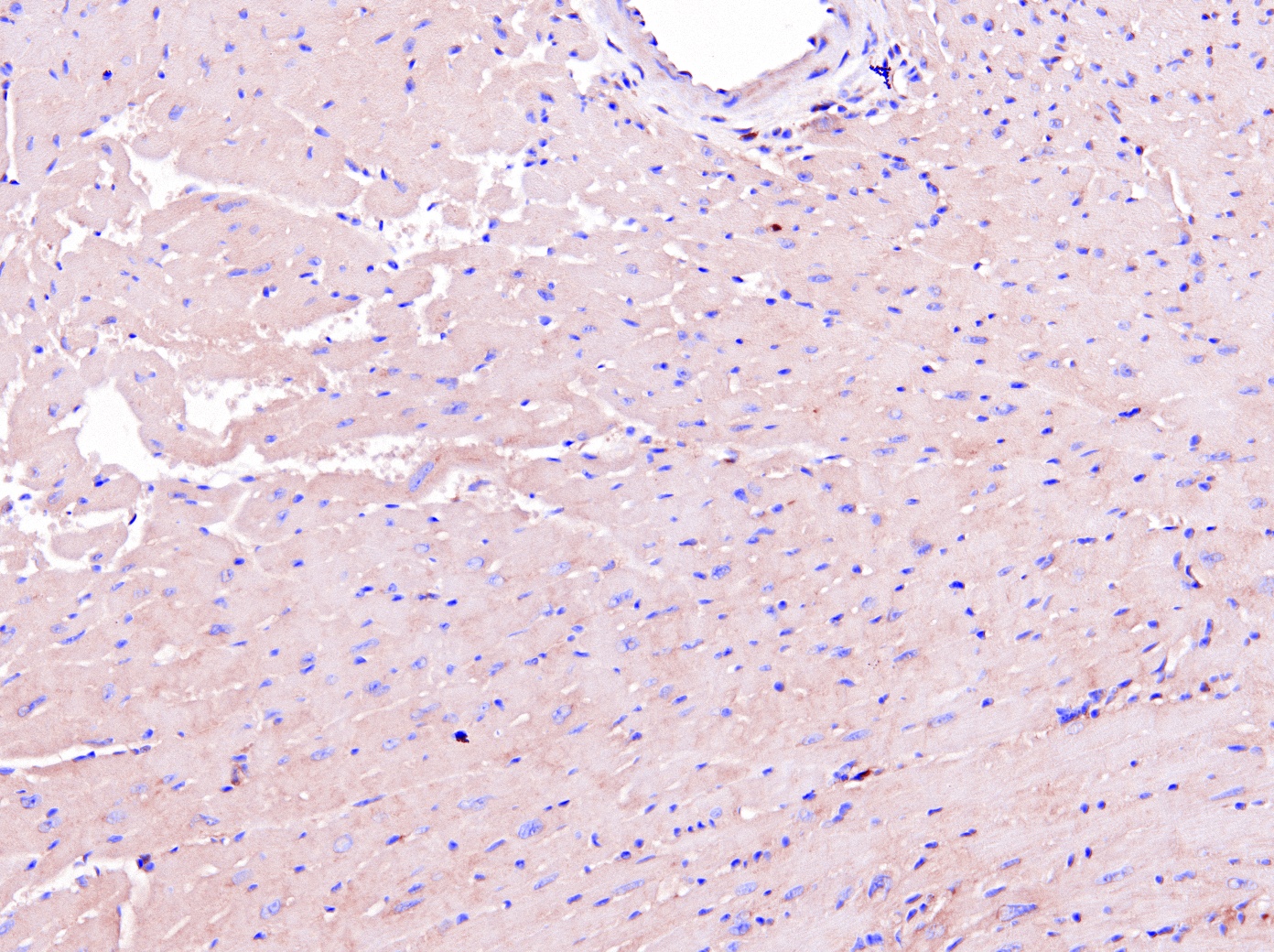

Supplement: Figure 4—source data 1. [file elife-55513-fig4-data1.zip › p16_images_for_eLife/p16_images_Ann_Chiao_for_eLife/Old Controls/OCL_8/4.2_Composite_i.jpg]

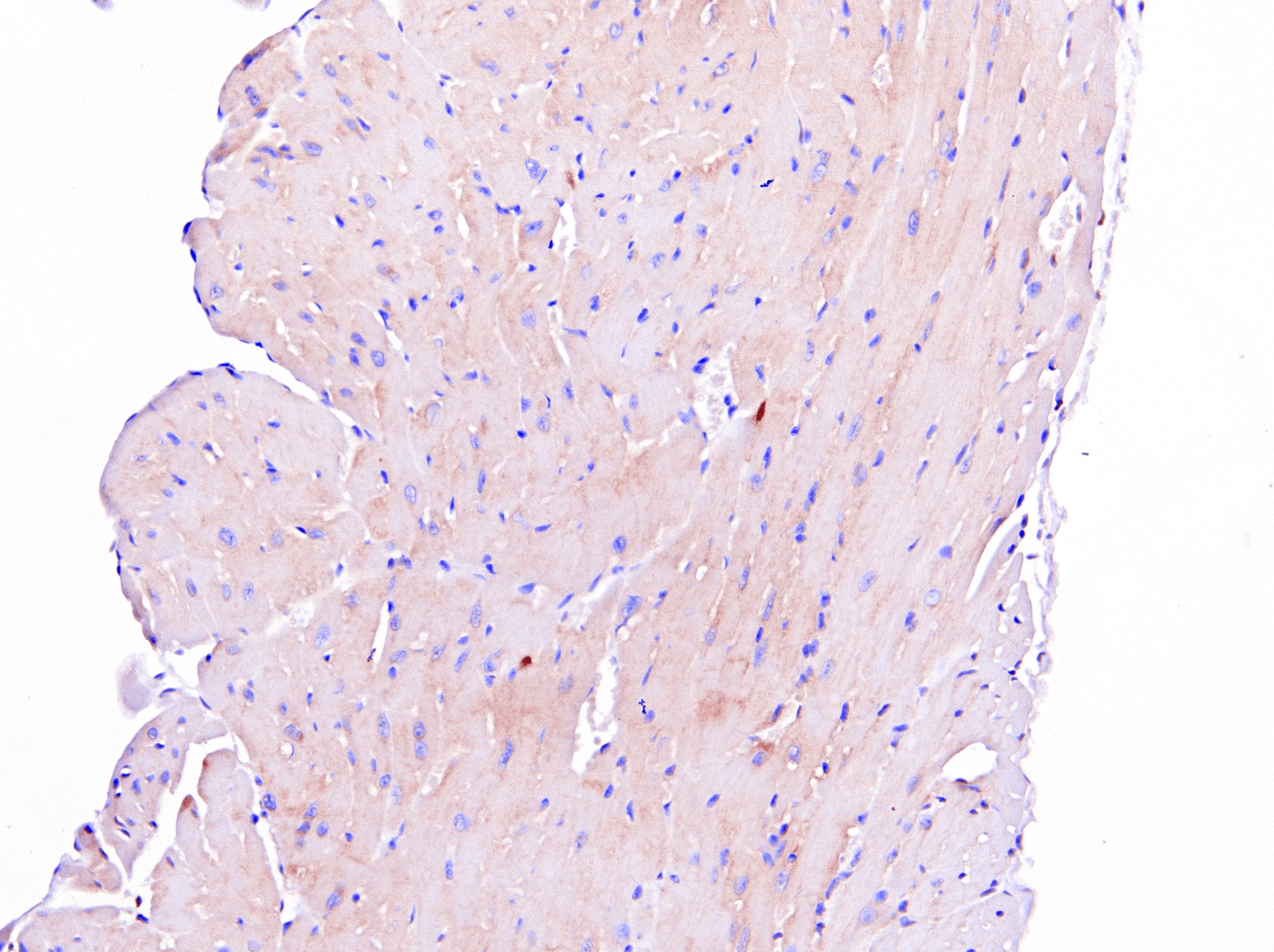

Supplement: Figure 4—source data 1. [file elife-55513-fig4-data1.zip › p16_images_for_eLife/p16_images_Ann_Chiao_for_eLife/Old Controls/OCL_8/4.2_Composite_j.jpg]

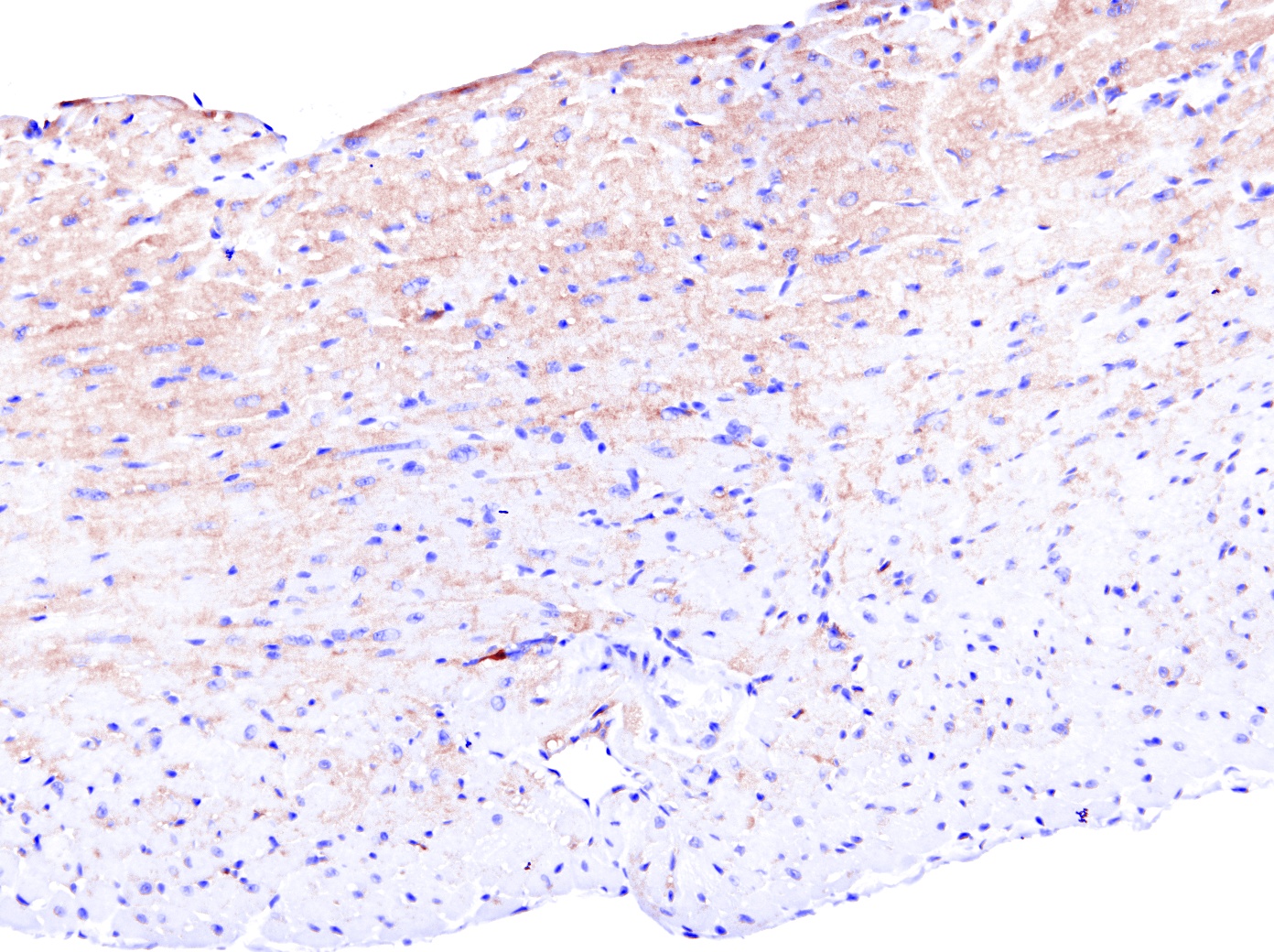

Supplement: Figure 4—source data 1. [file elife-55513-fig4-data1.zip › p16_images_for_eLife/p16_images_Ann_Chiao_for_eLife/Old Controls/OCL_9/Composite_7.3_a.jpg]

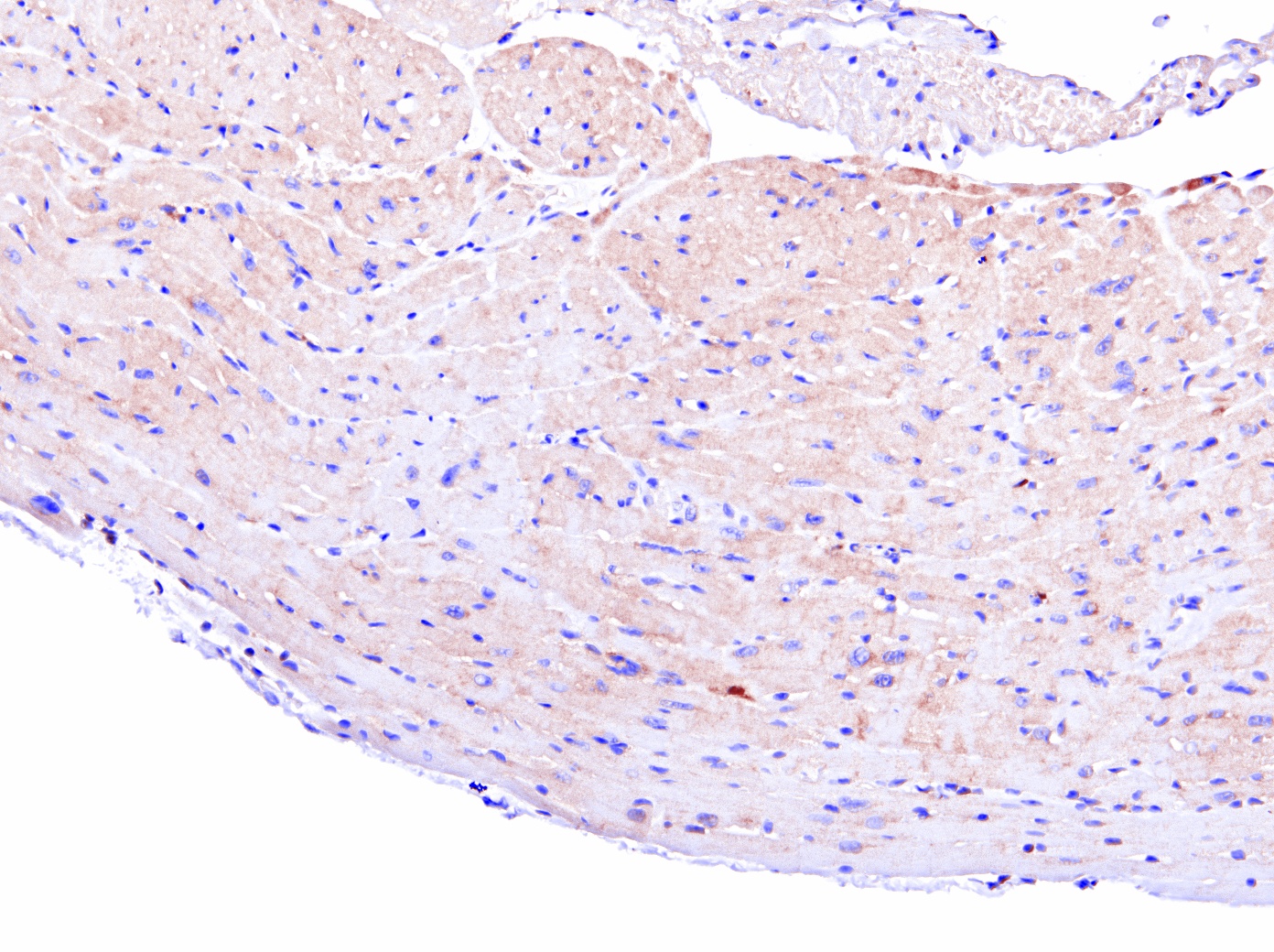

Supplement: Figure 4—source data 1. [file elife-55513-fig4-data1.zip › p16_images_for_eLife/p16_images_Ann_Chiao_for_eLife/Old Controls/OCL_9/Composite_7.3_b.jpg]

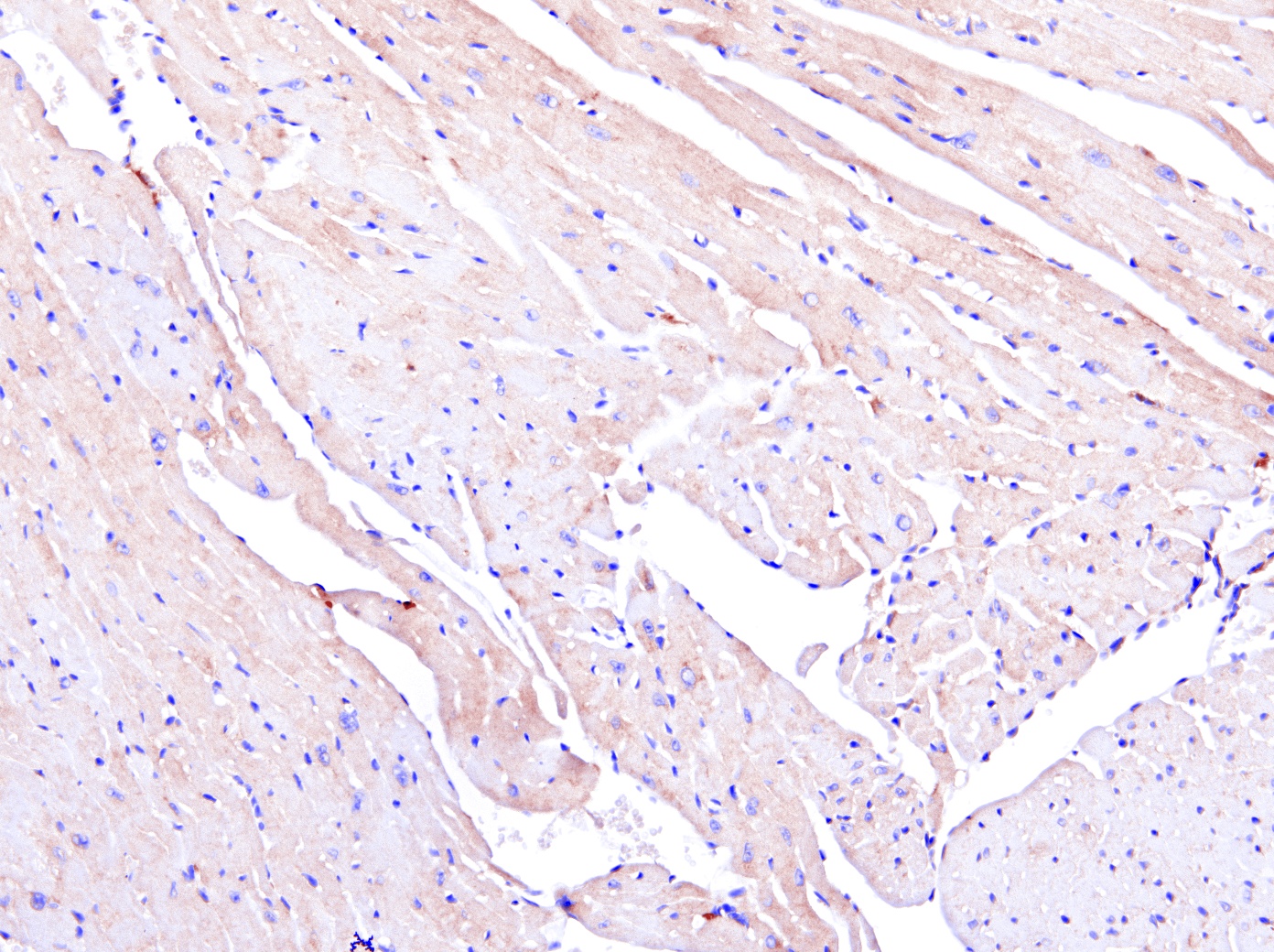

Supplement: Figure 4—source data 1. [file elife-55513-fig4-data1.zip › p16_images_for_eLife/p16_images_Ann_Chiao_for_eLife/Old Controls/OCL_9/Composite_7.3_c.jpg]
