# Supplementary figures and images for: Late-life restoration of mitochondrial function reverses cardiac dysfunction in old mice (part 3 of 3)
Source: eLife. 2020 Jul 10;9:e55513. doi: 10.7554/eLife.55513 (PMC7377906; doi:10.7554/eLife.55513)

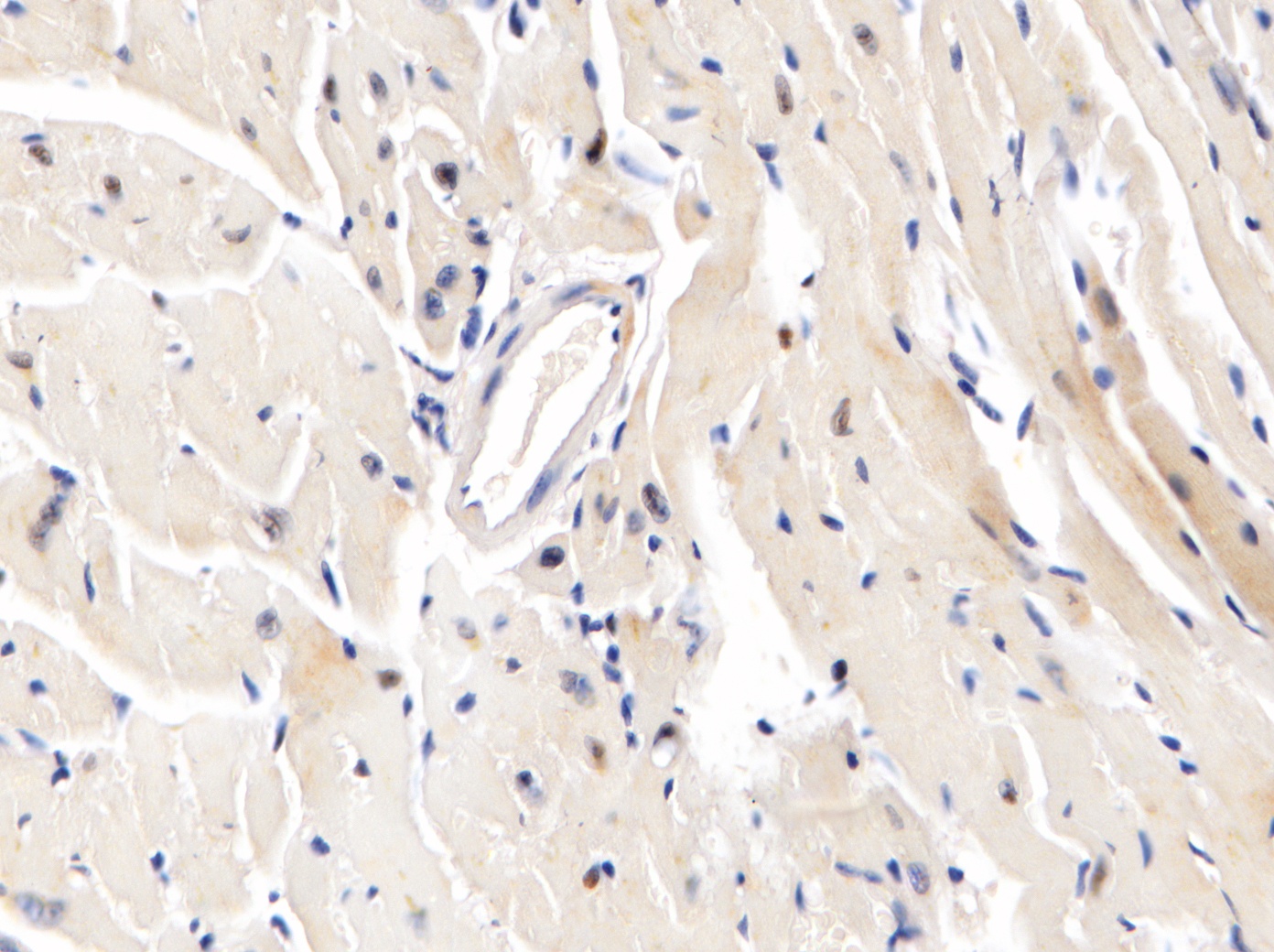

Supplement: Figure 4—source data 2. [file elife-55513-fig4-data2.zip › p19_images_for_eLife/p19_images_Ann_Chiao_for_eLife/Old SS-31 treated/OSS_3/Copy of MS6_2_40x_RGB.jpg]

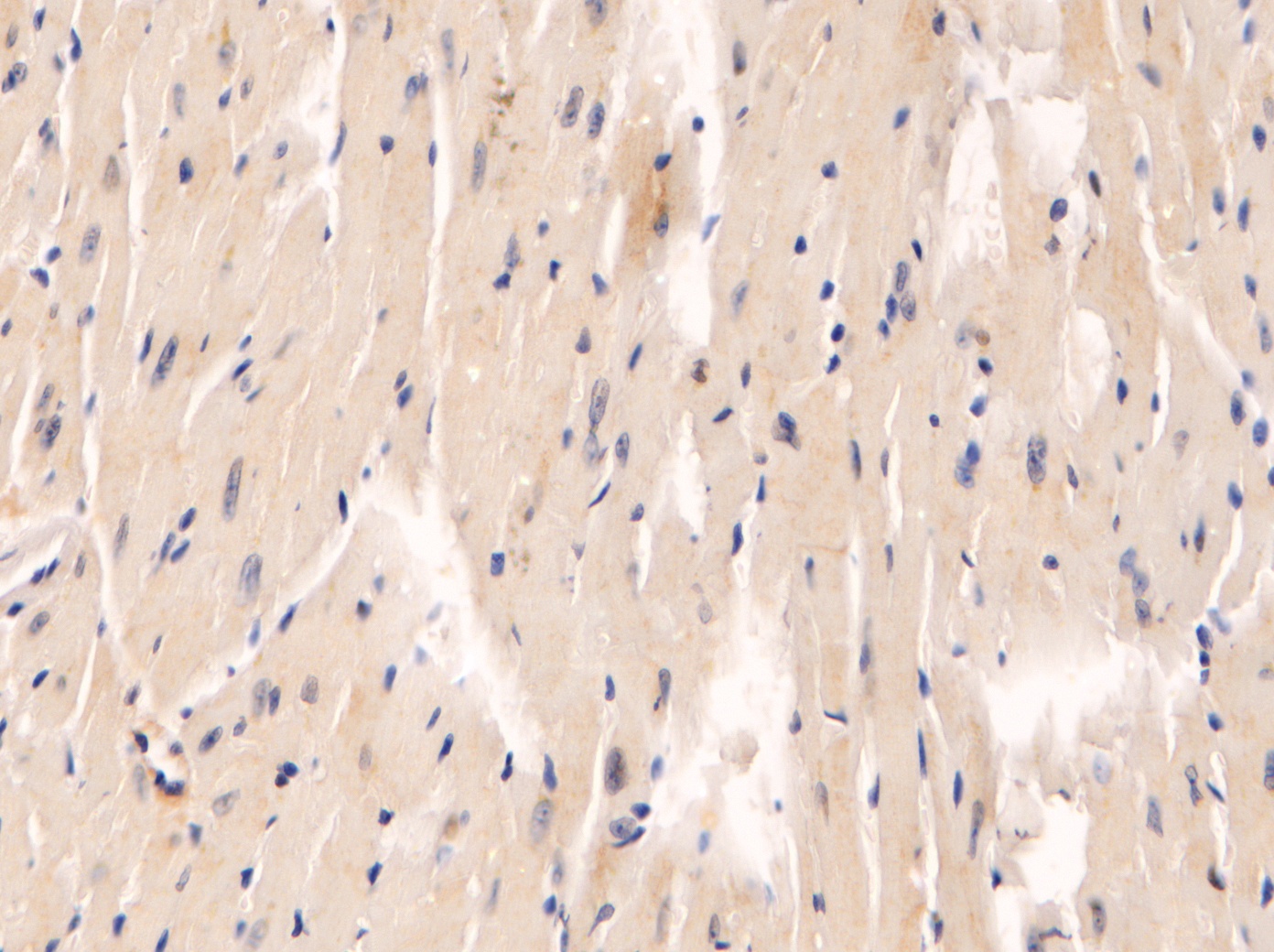

Supplement: Figure 4—source data 2. [file elife-55513-fig4-data2.zip › p19_images_for_eLife/p19_images_Ann_Chiao_for_eLife/Old SS-31 treated/OSS_3/Copy of MS6_3_40x_RGB.jpg]

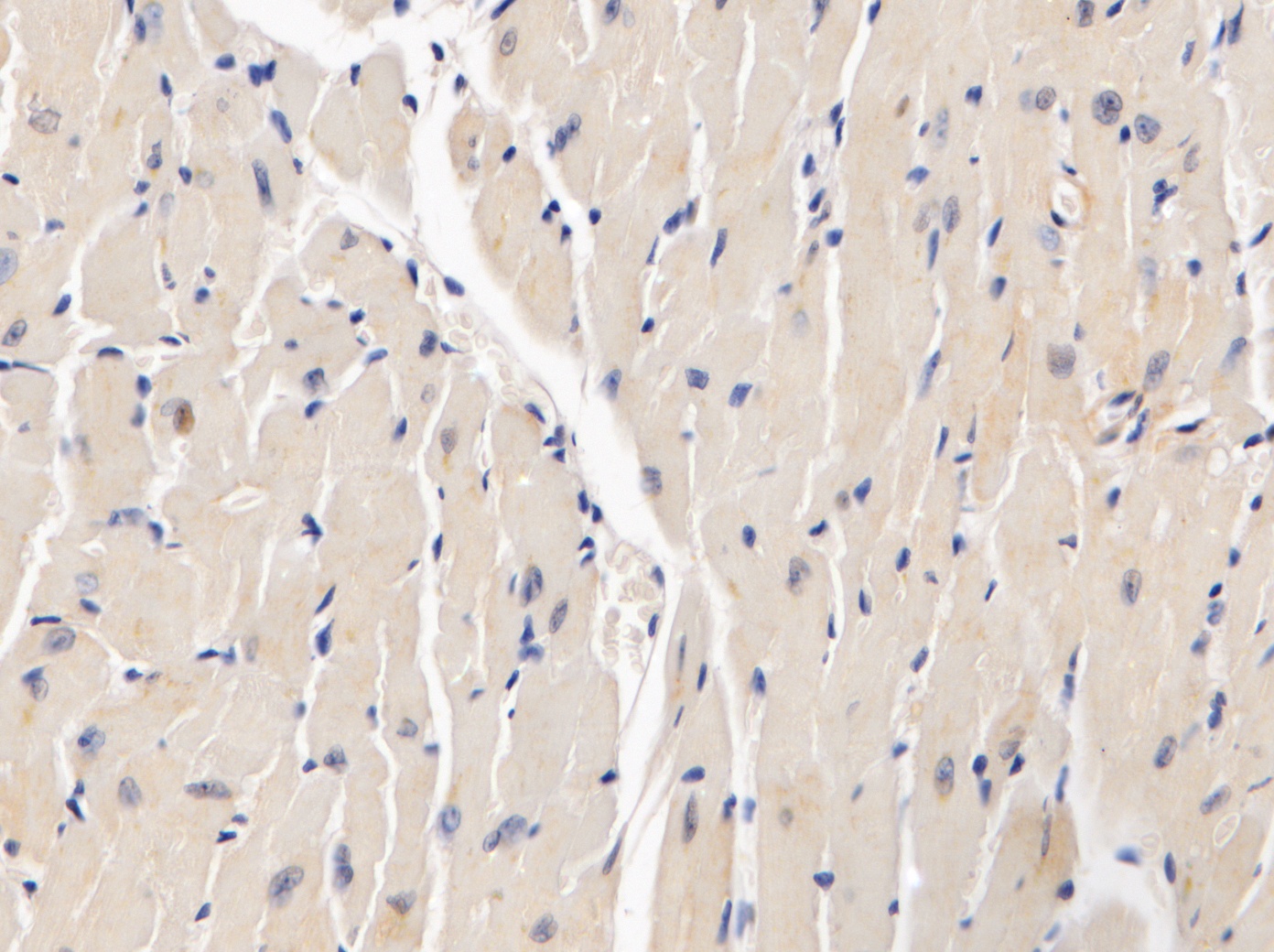

Supplement: Figure 4—source data 2. [file elife-55513-fig4-data2.zip › p19_images_for_eLife/p19_images_Ann_Chiao_for_eLife/Old SS-31 treated/OSS_3/Copy of MS6_4_40x_RGB.jpg]

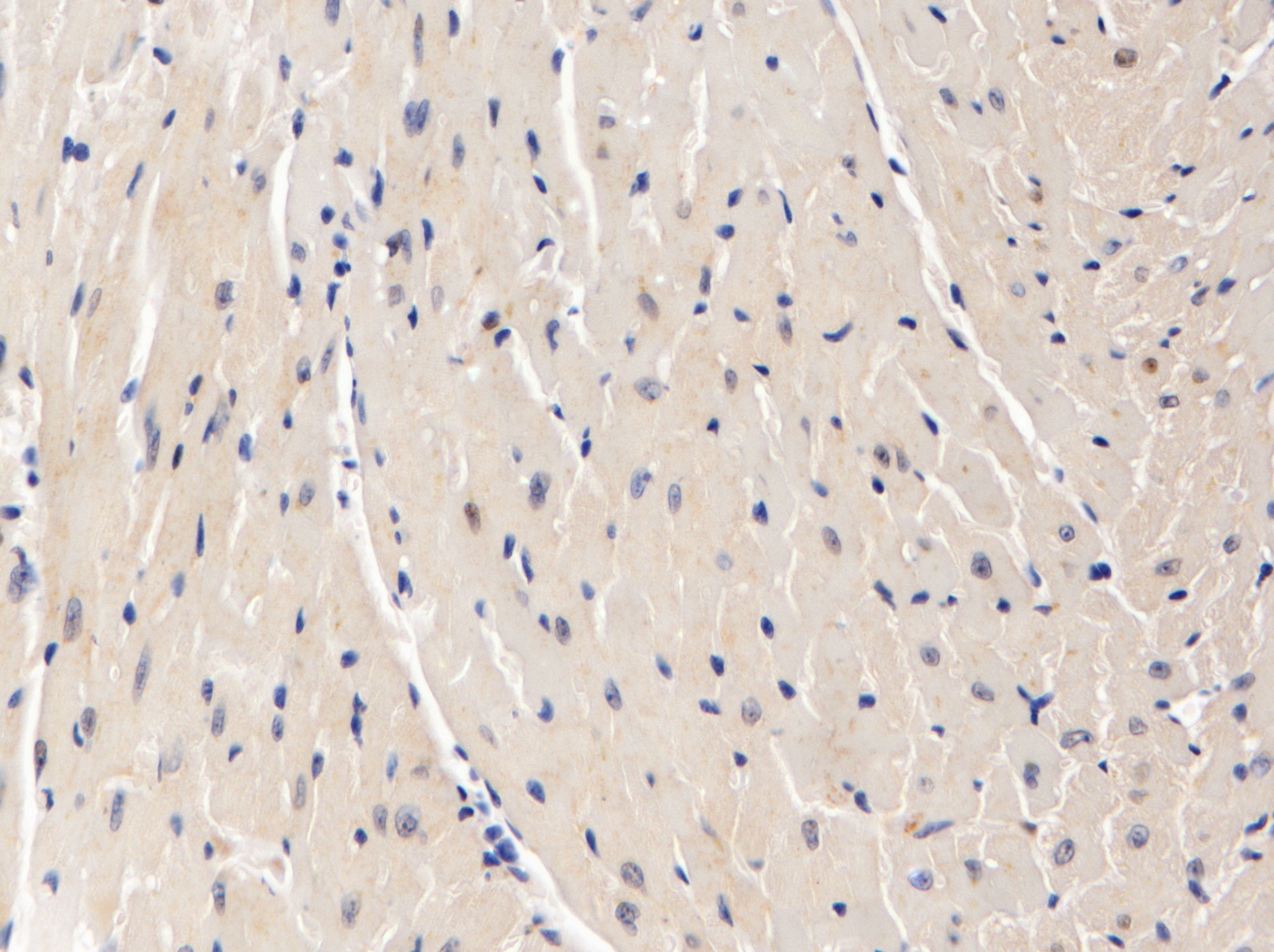

Supplement: Figure 4—source data 2. [file elife-55513-fig4-data2.zip › p19_images_for_eLife/p19_images_Ann_Chiao_for_eLife/Old SS-31 treated/OSS_3/Copy of MS6_5_40x_RGB.jpg]

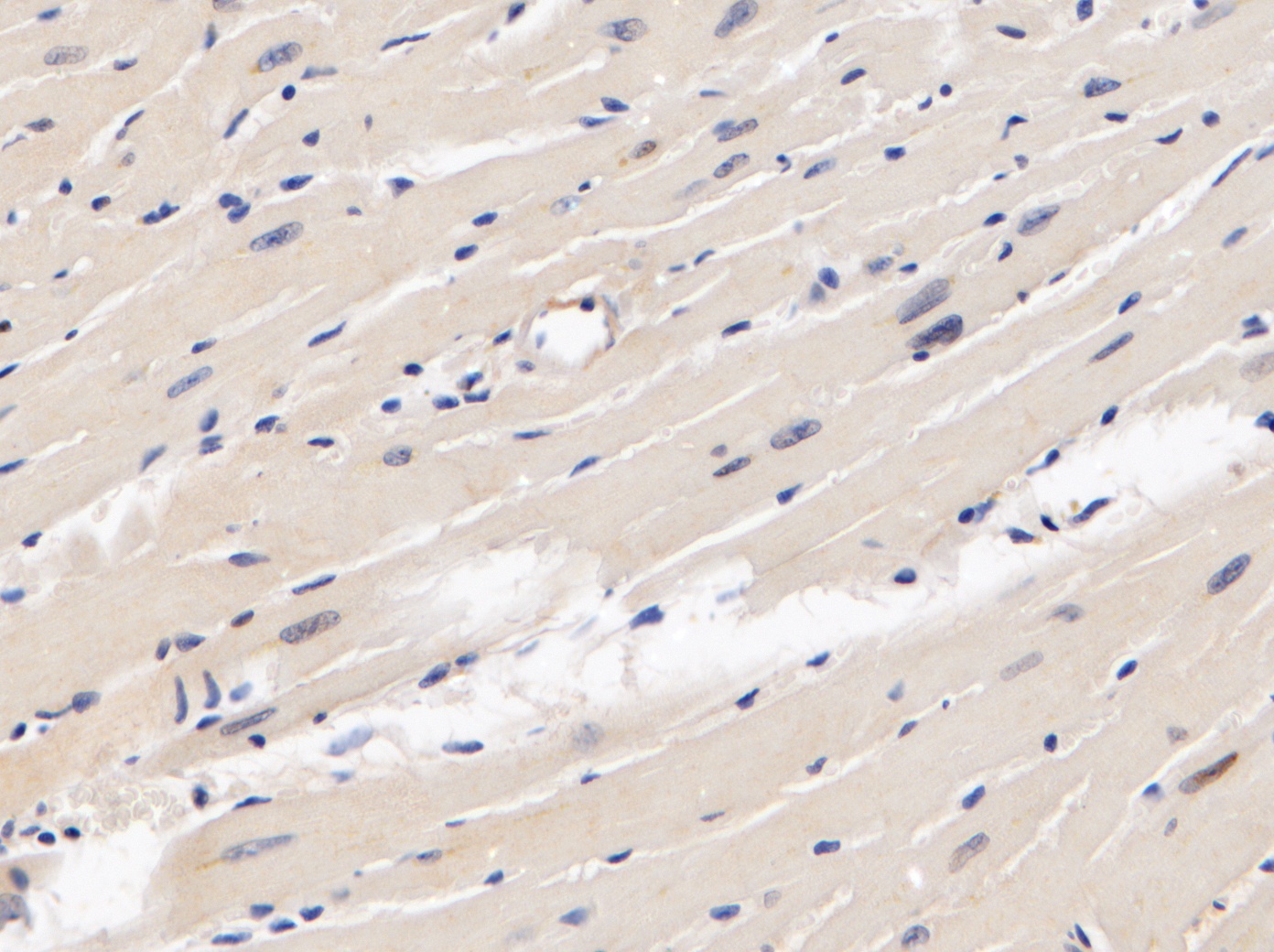

Supplement: Figure 4—source data 2. [file elife-55513-fig4-data2.zip › p19_images_for_eLife/p19_images_Ann_Chiao_for_eLife/Old SS-31 treated/OSS_3/Copy of MS6_6_40x_RGB.jpg]

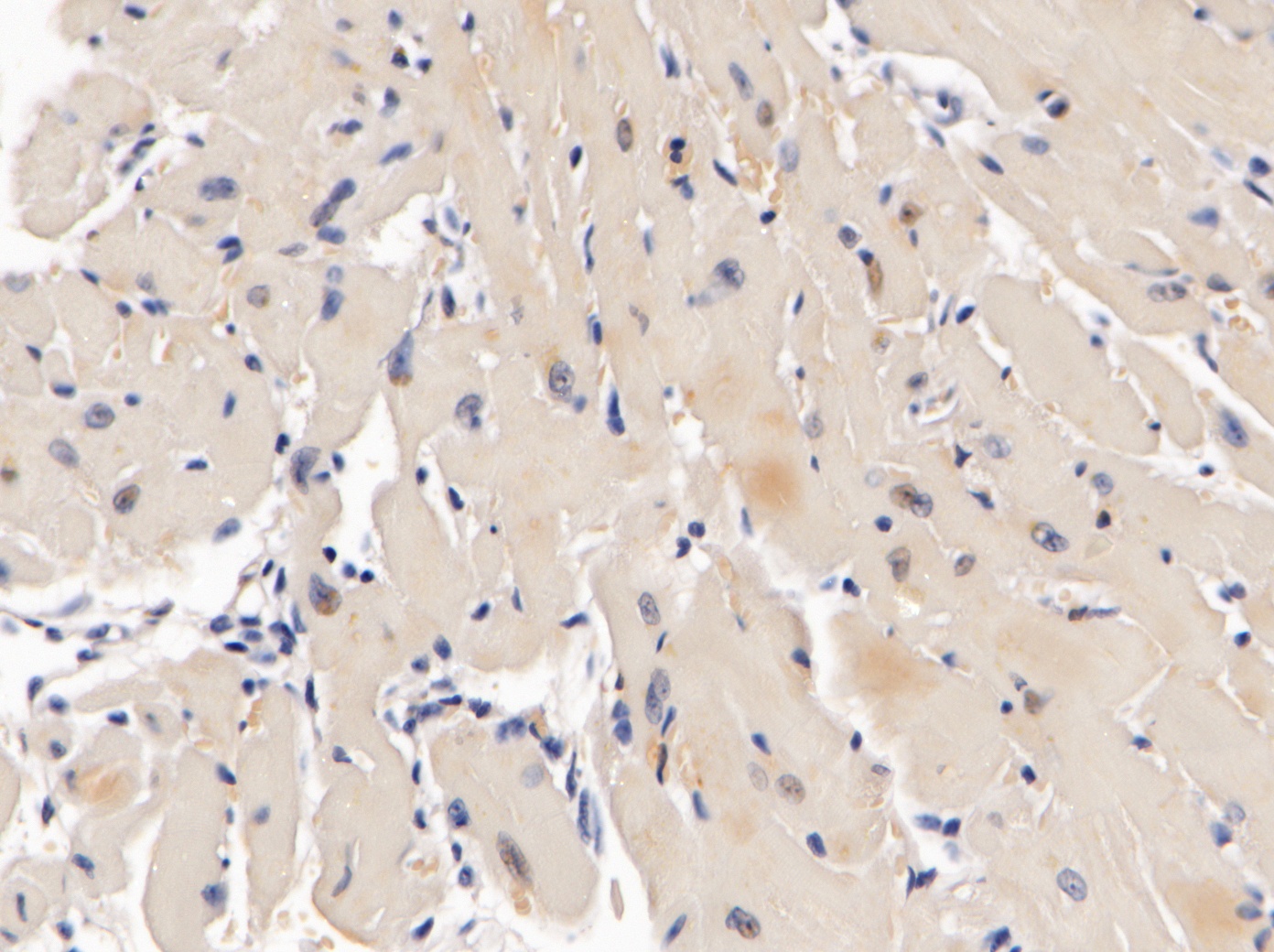

Supplement: Figure 4—source data 2. [file elife-55513-fig4-data2.zip › p19_images_for_eLife/p19_images_Ann_Chiao_for_eLife/Old SS-31 treated/OSS_4/Copy of MS8_1_40x_RGB.jpg]

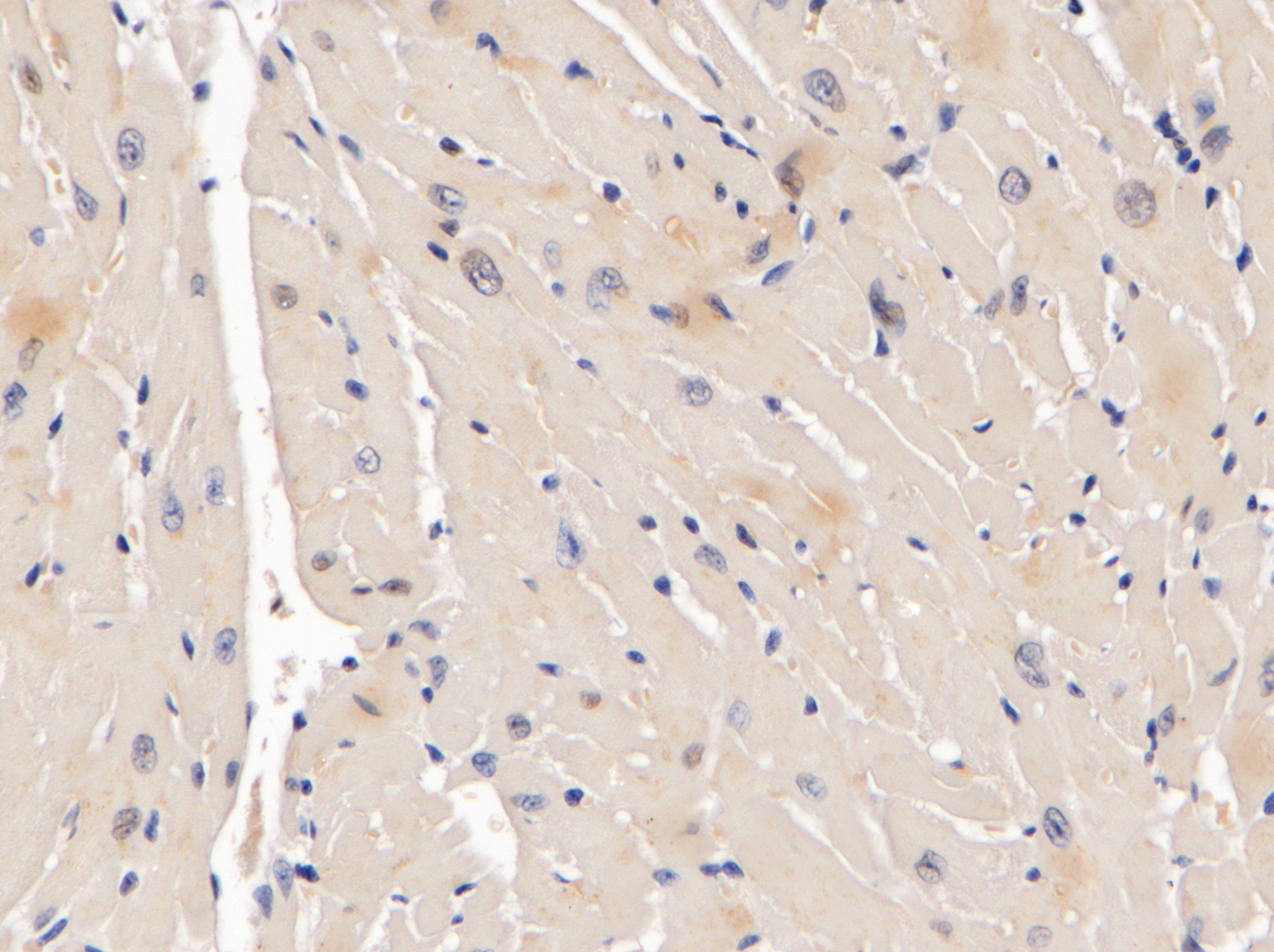

Supplement: Figure 4—source data 2. [file elife-55513-fig4-data2.zip › p19_images_for_eLife/p19_images_Ann_Chiao_for_eLife/Old SS-31 treated/OSS_4/Copy of MS8_2_40x_RGB.jpg]

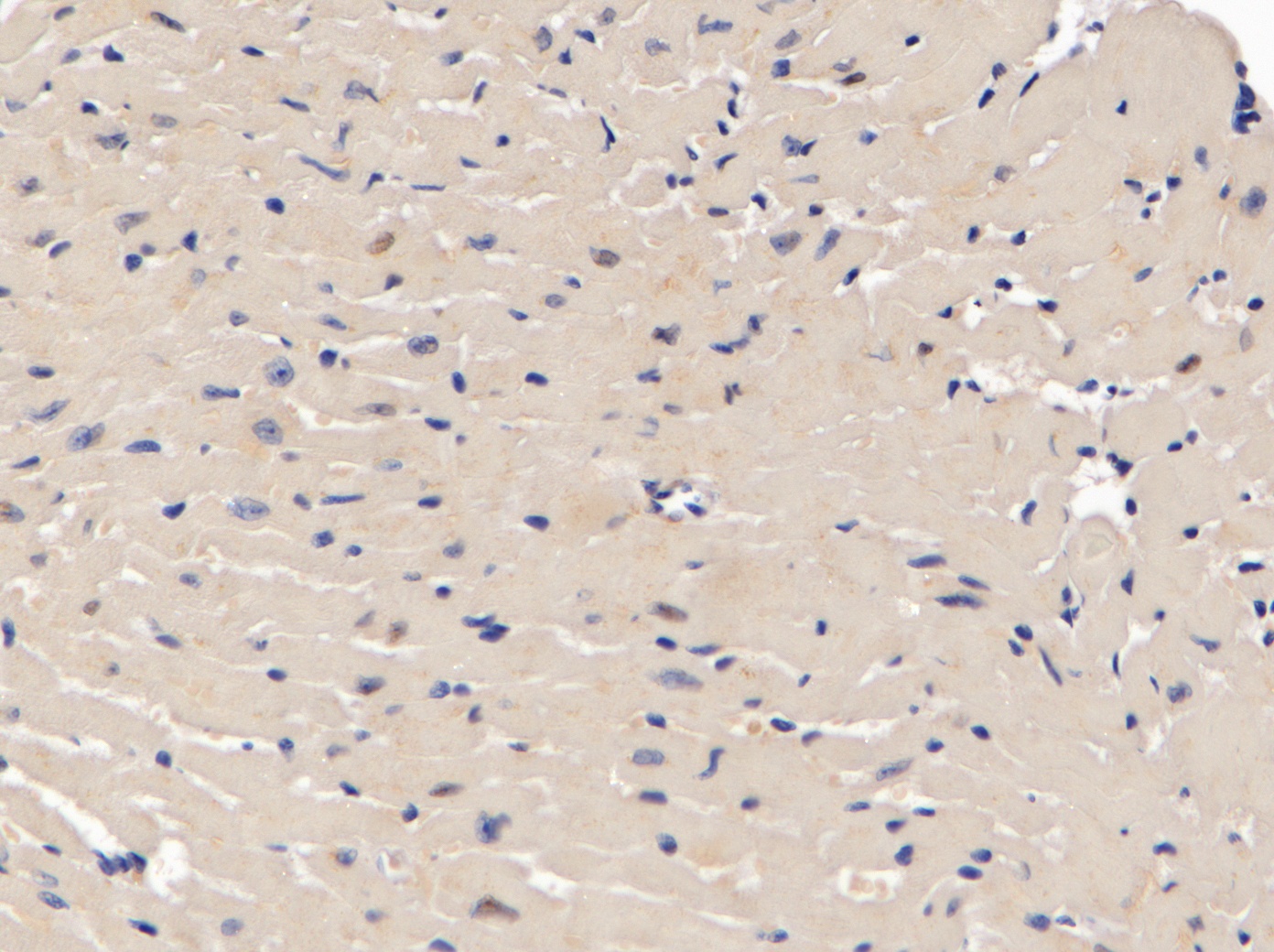

Supplement: Figure 4—source data 2. [file elife-55513-fig4-data2.zip › p19_images_for_eLife/p19_images_Ann_Chiao_for_eLife/Old SS-31 treated/OSS_4/Copy of MS8_3_40x_RGB.jpg]

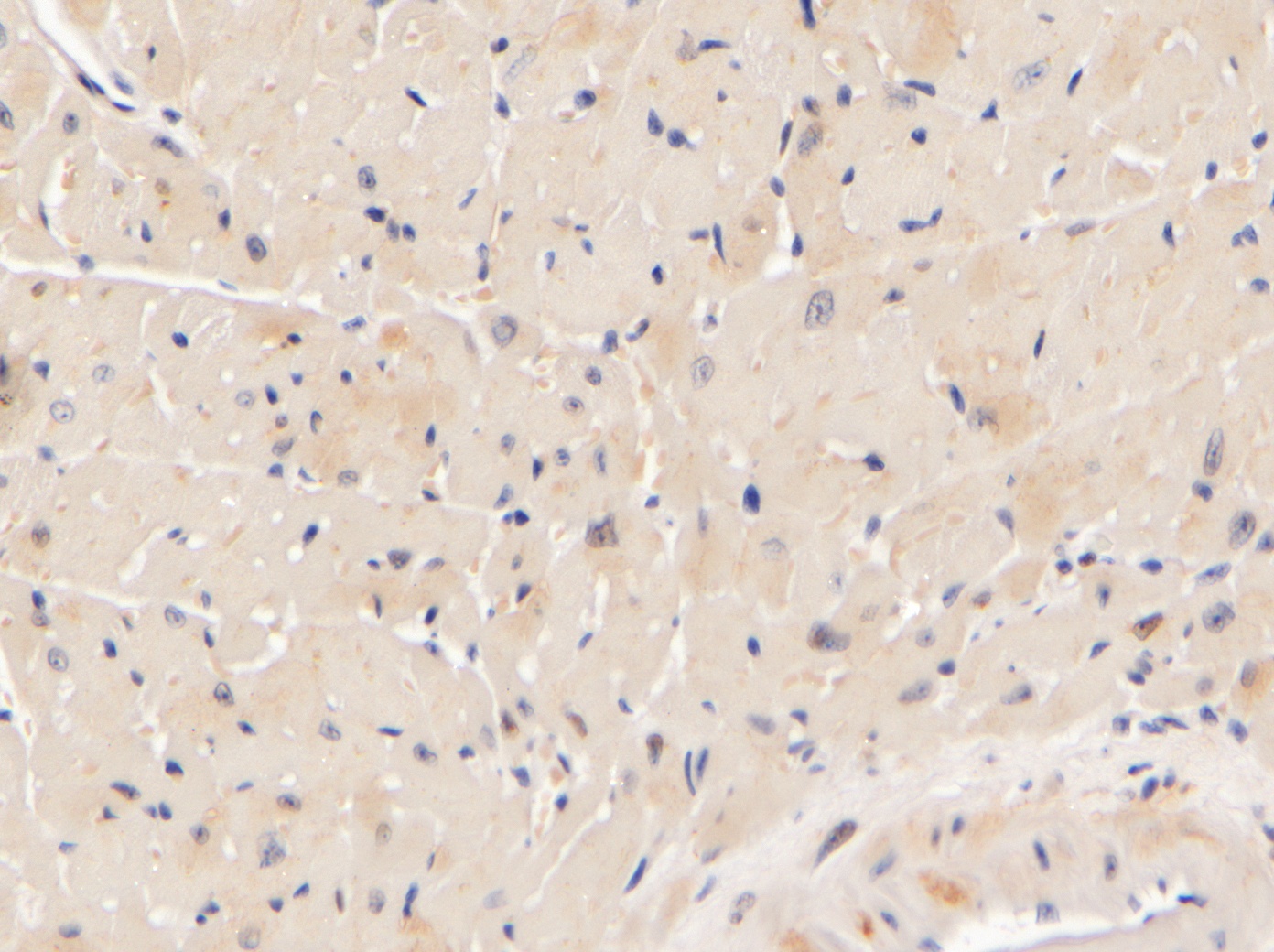

Supplement: Figure 4—source data 2. [file elife-55513-fig4-data2.zip › p19_images_for_eLife/p19_images_Ann_Chiao_for_eLife/Old SS-31 treated/OSS_4/Copy of MS8_4_40x_RGB.jpg]

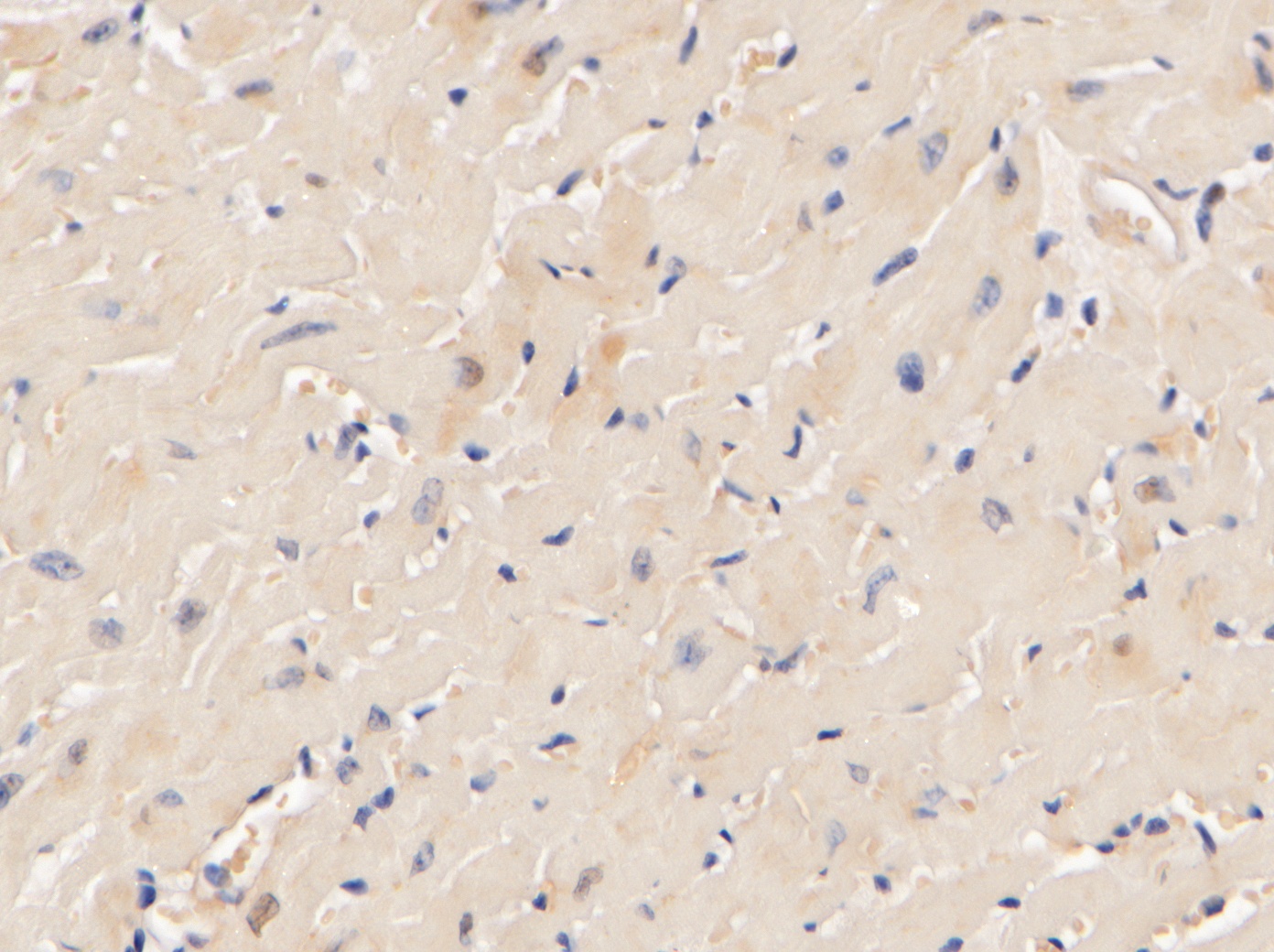

Supplement: Figure 4—source data 2. [file elife-55513-fig4-data2.zip › p19_images_for_eLife/p19_images_Ann_Chiao_for_eLife/Old SS-31 treated/OSS_4/Copy of MS8_5_40x_RGB.jpg]

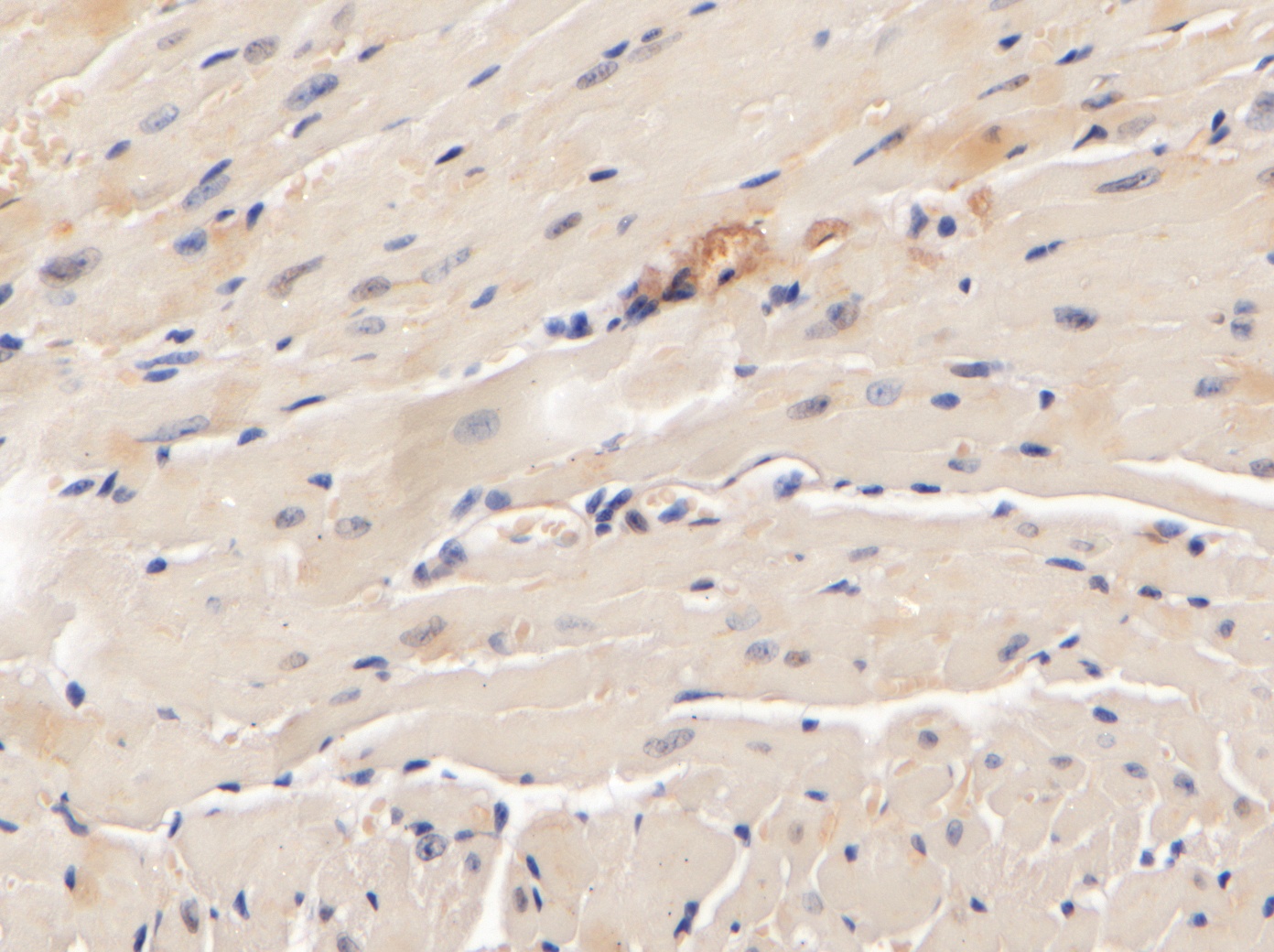

Supplement: Figure 4—source data 2. [file elife-55513-fig4-data2.zip › p19_images_for_eLife/p19_images_Ann_Chiao_for_eLife/Old SS-31 treated/OSS_4/Copy of MS8_6_40x_RGB.jpg]

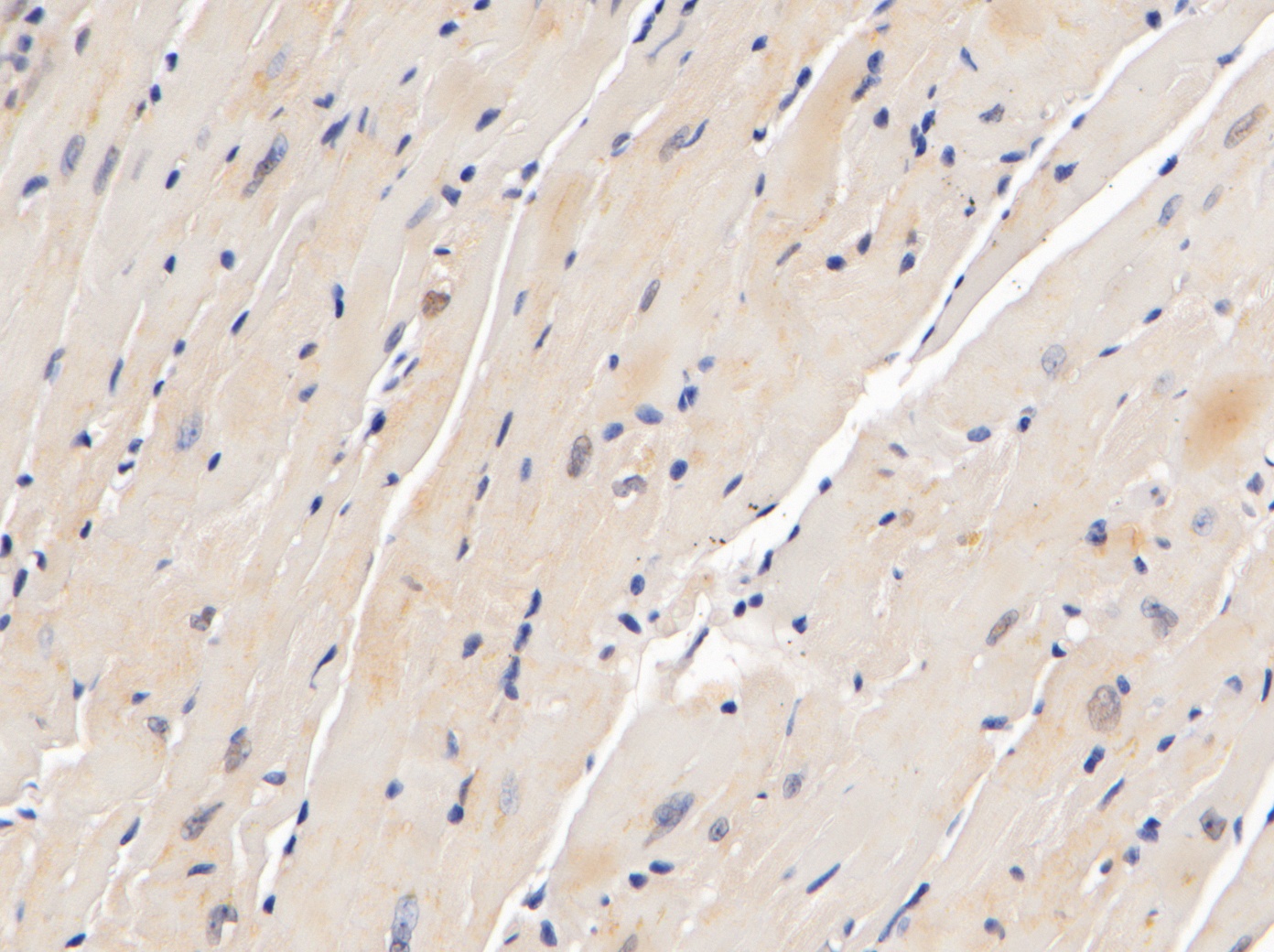

Supplement: Figure 4—source data 2. [file elife-55513-fig4-data2.zip › p19_images_for_eLife/p19_images_Ann_Chiao_for_eLife/Old SS-31 treated/OSS_5/Copy of MS9_1_40x_RGB.jpg]

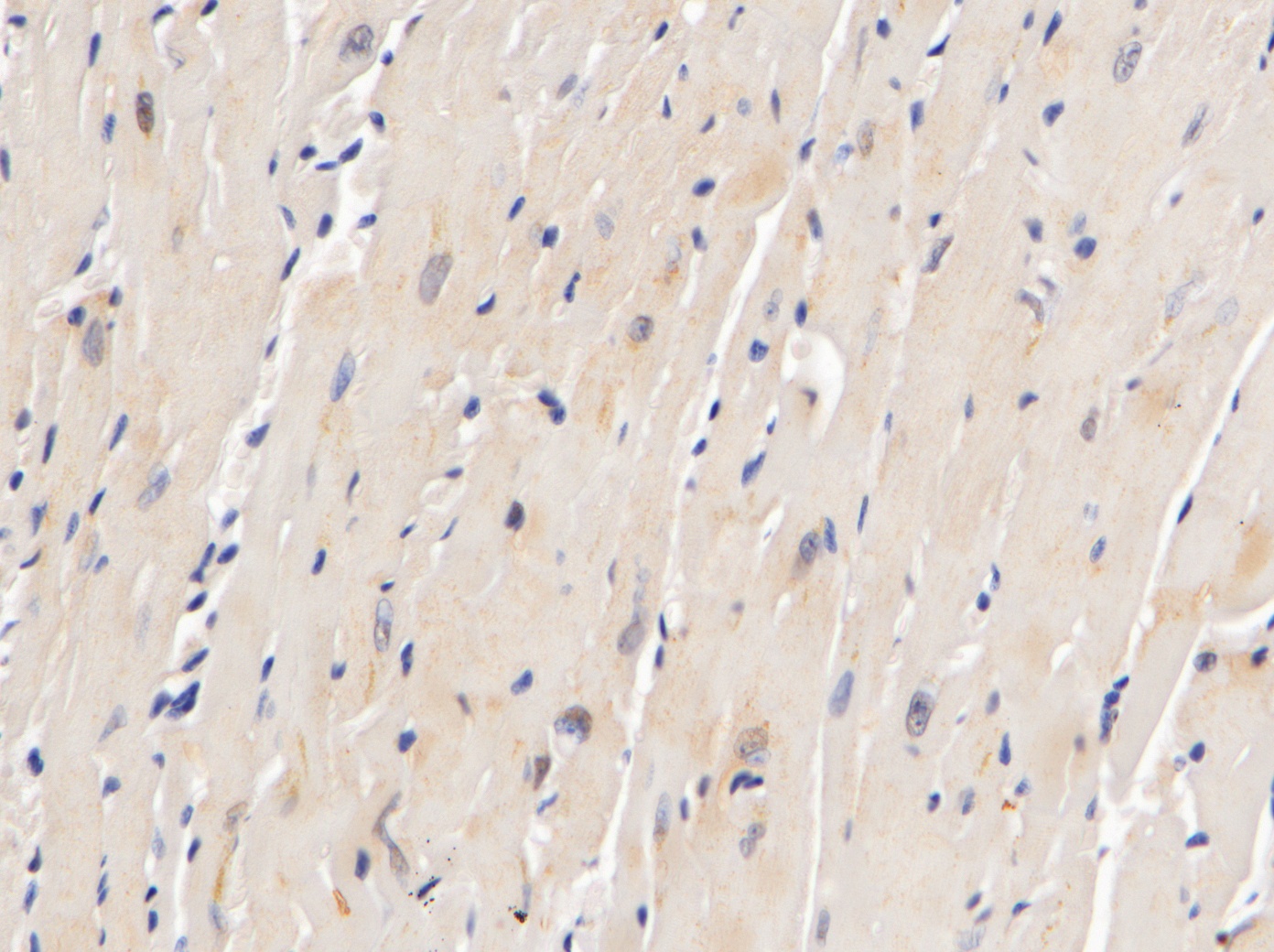

Supplement: Figure 4—source data 2. [file elife-55513-fig4-data2.zip › p19_images_for_eLife/p19_images_Ann_Chiao_for_eLife/Old SS-31 treated/OSS_5/Copy of MS9_2_40x_RGB.jpg]

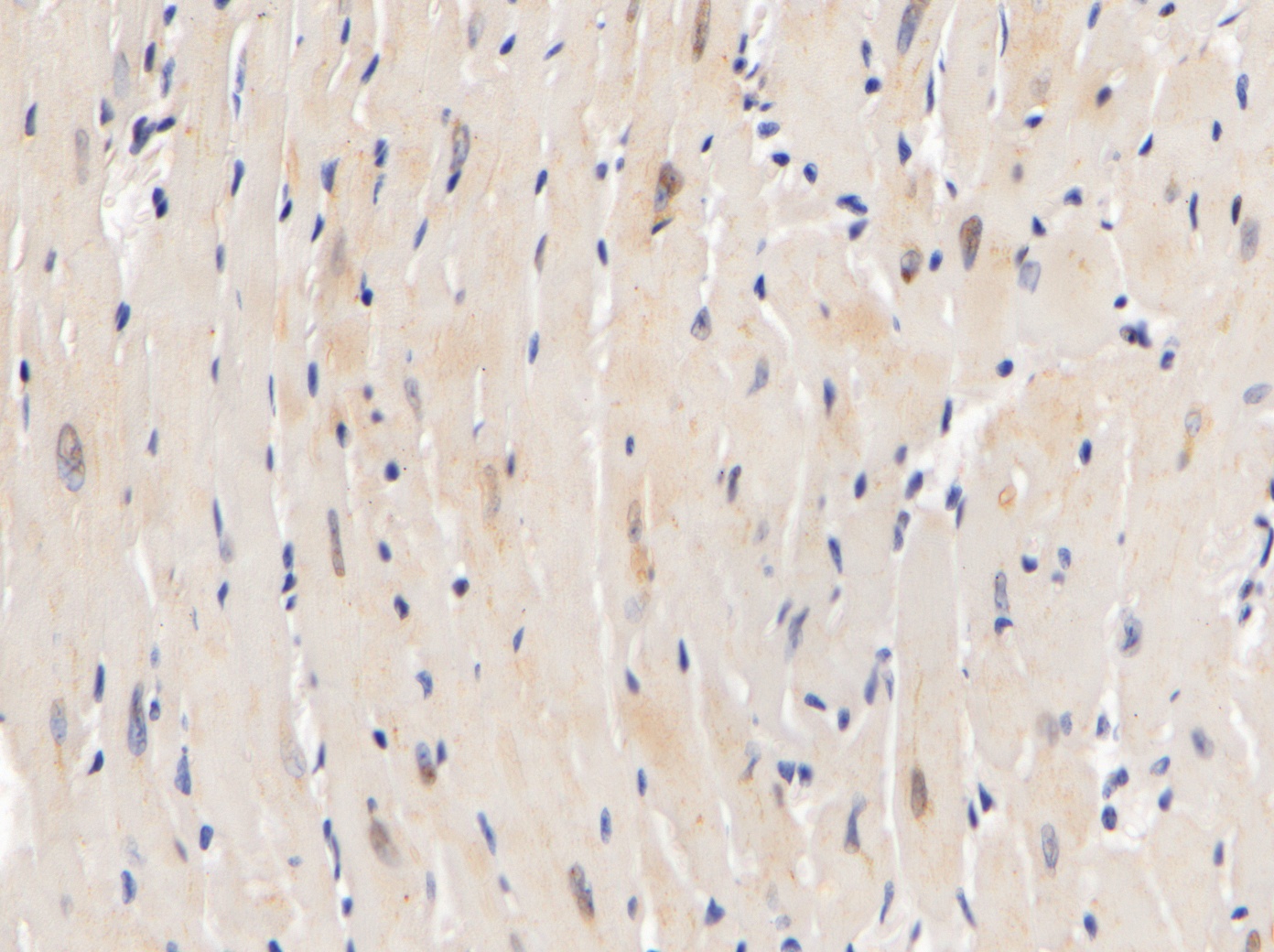

Supplement: Figure 4—source data 2. [file elife-55513-fig4-data2.zip › p19_images_for_eLife/p19_images_Ann_Chiao_for_eLife/Old SS-31 treated/OSS_5/Copy of MS9_3_40x_RGB.jpg]

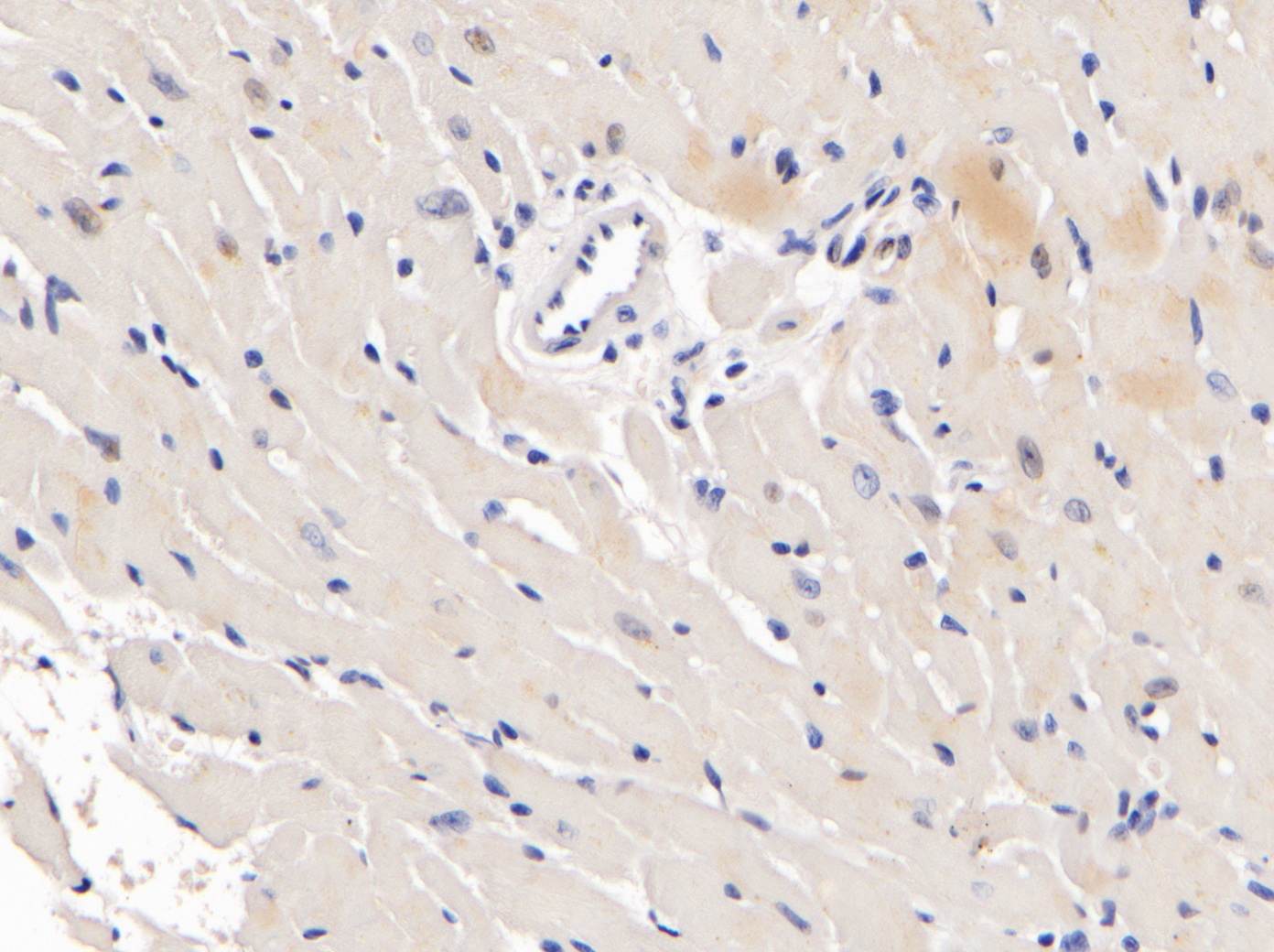

Supplement: Figure 4—source data 2. [file elife-55513-fig4-data2.zip › p19_images_for_eLife/p19_images_Ann_Chiao_for_eLife/Old SS-31 treated/OSS_5/Copy of MS9_4_40x_RGB.jpg]

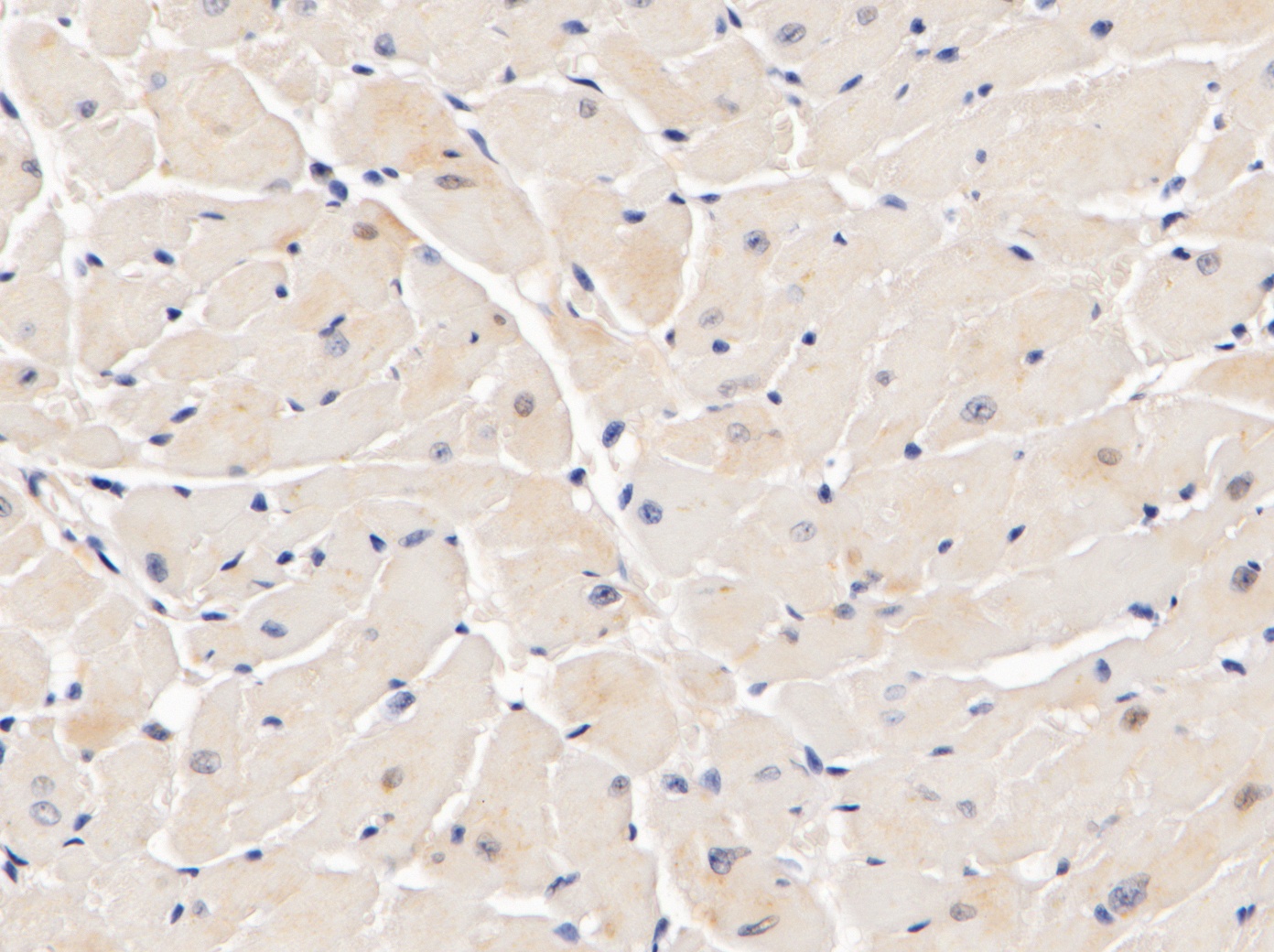

Supplement: Figure 4—source data 2. [file elife-55513-fig4-data2.zip › p19_images_for_eLife/p19_images_Ann_Chiao_for_eLife/Old SS-31 treated/OSS_5/Copy of MS9_5_40x_RGB.jpg]

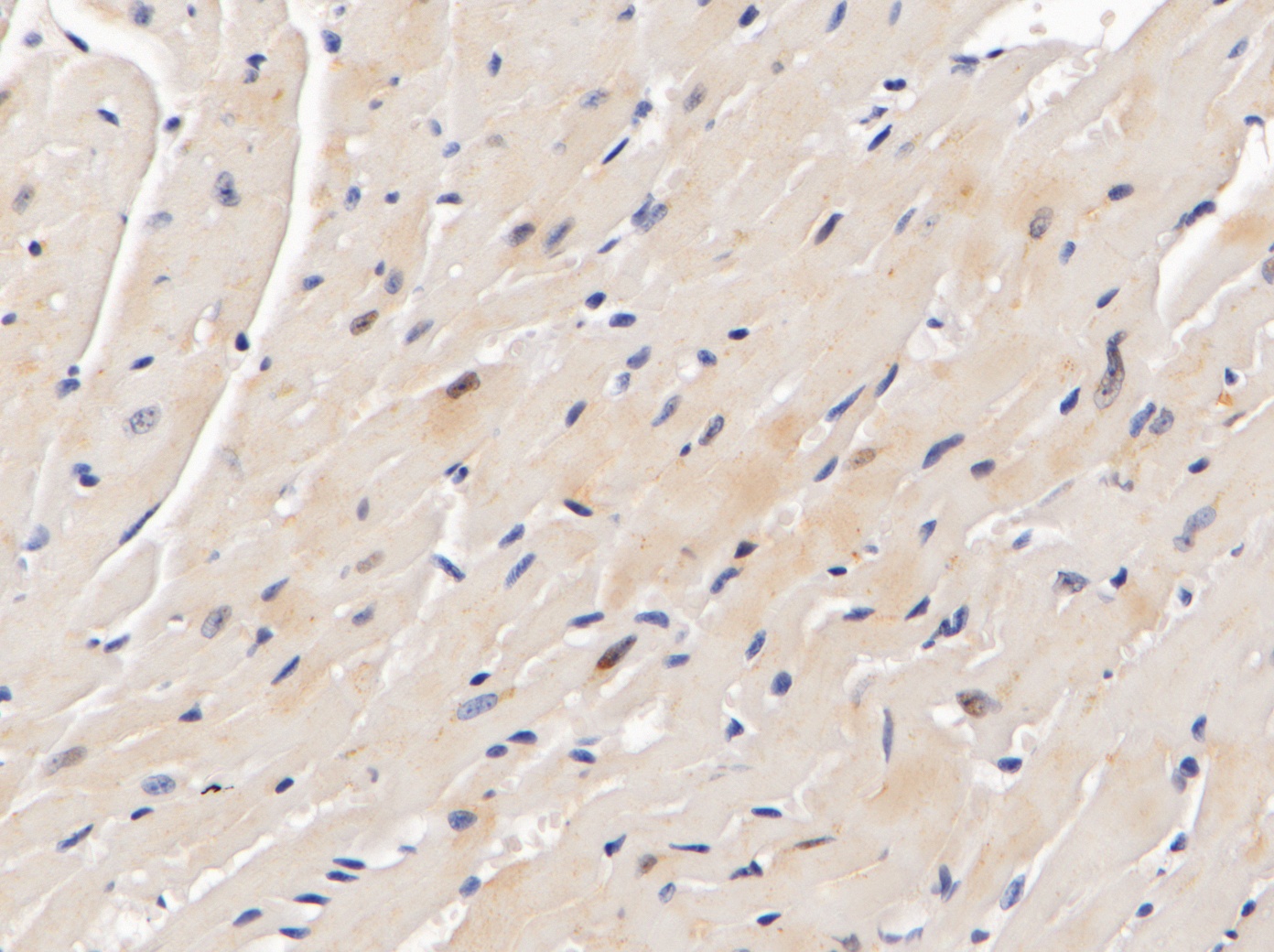

Supplement: Figure 4—source data 2. [file elife-55513-fig4-data2.zip › p19_images_for_eLife/p19_images_Ann_Chiao_for_eLife/Old SS-31 treated/OSS_5/Copy of MS9_6_40x_RGB.jpg]
