# Supplementary material for: Late-life restoration of mitochondrial function reverses cardiac dysfunction in old mice
Source: eLife. 2020 Jul 10;9:e55513. doi: 10.7554/eLife.55513 (PMC7377906; doi:10.7554/eLife.55513)
Supplement: Transparent reporting form [file elife-55513-transrepform.pdf]

eLIFE
1st Floor
24 Hills Road
Cambridge CB2 1JP, UK
P 01223 855340
W elifesciences.org
T @elife

eLife's transparent reporting form

We encourage authors to provide detailed information *within their submission* to facilitate the interpretation and replication of experiments. Authors can upload supporting documentation to indicate the use of appropriate reporting guidelines for health-related research (see EQUATOR Network), life science research (see the BioSharing Information Resource), or the ARRIVE guidelines for reporting work involving animal research. Where applicable, authors should refer to any relevant reporting standards documents in this form.

If you have any questions, please consult our Journal Policies and/or contact us: editorial@elifesciences.org.

Sample-size estimation

- You should state whether an appropriate sample size was computed when the study was being designed
- You should state the statistical method of sample size computation and any required assumptions
- If no explicit power analysis was used, you should describe how you decided what sample (replicate) size (number) to use

Please outline where this information can be found within the submission (e.g., sections or figure legends), or explain why this information doesn't apply to your submission:

Sample sizes for animal experiments were decided based on previously published studies from our group. Due to the mortality of old mice during the study, the final sample sizes varies between experiments.

Replicates

- You should report how often each experiment was performed
- You should include a definition of biological versus technical replication
- The data obtained should be provided and sufficient information should be provided to indicate the number of independent biological and/or technical replicates
- If you encountered any outliers, you should describe how these were handled
- Criteria for exclusion/inclusion of data should be clearly stated
- High-throughput sequence data should be uploaded before submission, with a private link for reviewers provided (these are available from both GEO and ArrayExpress)

Please outline where this information can be found within the submission (e.g., sections or figure legends), or explain why this information doesn't apply to your submission:

The information on biological and technical replication can be found in figure legends. Data from mice that died before designed endpoints were excluded from study as stated in the method section.

Statistical reporting

- Statistical analysis methods should be described and justified
- Raw data should be presented in figures whenever informative to do so (typically when N per group is less than 10)
- For each experiment, you should identify the statistical tests used, exact values of N, definitions of center, methods of multiple test correction, and dispersion and precision measures (e.g., mean, median, SD, SEM, confidence intervals; and, for the major substantive results, a measure of effect size (e.g., Pearson's r, Cohen's d)
- Report exact p-values wherever possible alongside the summary statistics and 95% confidence intervals. These should be reported for all key questions and not only when the p-value is less than 0.05.

Please outline where this information can be found within the submission (e.g., sections or figure legends), or explain why this information doesn't apply to your submission:

Statistical analysis methods were described in method section and sample sizes for each experiment were described in figure legends. Images for immunoblots were included in figures, and individual data points were included in graphs when sample sizes were less than 10 per group.

(For large datasets, or papers with a very large number of statistical tests, you may upload a single table file with tests, Ns, etc., with reference to sections in the manuscript.)

Group allocation

- Indicate how samples were allocated into experimental groups (in the case of clinical studies, please specify allocation to treatment method); if randomization was used, please also state if restricted randomization was applied
- Indicate if masking was used during group allocation, data collection and/or data analysis

Please outline where this information can be found within the submission (e.g., sections or figure legends), or explain why this information doesn't apply to your submission:

Mice were randomly assigned to control or treatment group as described in method section.

Additional data files ("source data")

- We encourage you to upload relevant additional data files, such as numerical data that are represented as a graph in a figure, or as a summary table
- Where provided, these should be in the most useful format, and they can be uploaded as "Source data" files linked to a main figure or table
- Include model definition files including the full list of parameters used
- Include code used for data analysis (e.g., R, MatLab)
- Avoid stating that data files are "available upon request"

Please indicate the figures or tables for which source data files have been provided:

Data file for metabolomic analysis (Table S1) and statistics of all proteins identified by proteomic analysis (Table S3) have been provided as supplementary materials.
The raw mass spec files for proteomics analysis of S-glutathionylation were uploaded to MassIVE and can be accessed via the following link
ftp://massive.ucsd.edu/MSV000085329/
The raw mass spectrometry files for global proteomic analysis were uploaded to MassIVE and can be accessed via the following link
ftp://massive.ucsd.edu/MSV000084961/

The image files for ROS (Fig 2a and b) and senescence (Fig 4b and c) analyses can be accessed via the following link and were also included as supporting zip documents.
https://www.dropbox.com/sh/0r50ew6xnpunc3t/AAA_HbJ0fQwhOUpDbI9Oq07va?dl=0
